# Supplementary material for: A high‐throughput method to identify trans‐activation domains within transcription factor sequences
Source: EMBO J. 2018 Jul 13;37(16):e98896. doi: 10.15252/embj.201798896 (PMC6092621; doi:10.15252/embj.201798896)
Supplement: Supplementary file 1 — Appendix [file EMBJ-37-e98896-s001.pdf]

Appendix
for

A high-throughput method to identify trans-activation domains within transcription factor sequences

Cosmas D. Arnold*, Filip Nemčko*, Ashley R. Woodfin*, Sebastian Wienerroither*, Anna Vlasova*, Alexander Schleiffer, Michaela Pagani, Martina Rath, Alexander Stark

* shared first authors

Table of contents:

Appendix Figures S1-S4

Appendix Figure S1. Short-fragment library native (+1) reading-frame tADs
UCSC genome browser screenshots for 53 tADs (see Table EV2 for details).
One tAD per page – order as in Table EV2. 2

Appendix Figure S2. Short-fragment library non-native frame tADs
UCSC genome browser screenshots for 103 tADs (see Table EV4 for details). One tAD per page.
One tAD per page – order as in Table EV4. 56

Appendix Figure S3. Long-fragment library native (+1) reading-frame tADs
UCSC genome browser screenshots for 18 tADs (see Table EV5 for details).
One tAD per page – order as in Table EV5. 160

Appendix Figure S4. Long-fragment library non-native frame tADs
UCSC genome browser screenshots for 13 tADs (see Table EV6 for details). One tAD per page.
One tAD per page – order as in Table EV6. 179

Appendix Figure S1

53 short-fragment library native (+1) reading-frame tADs

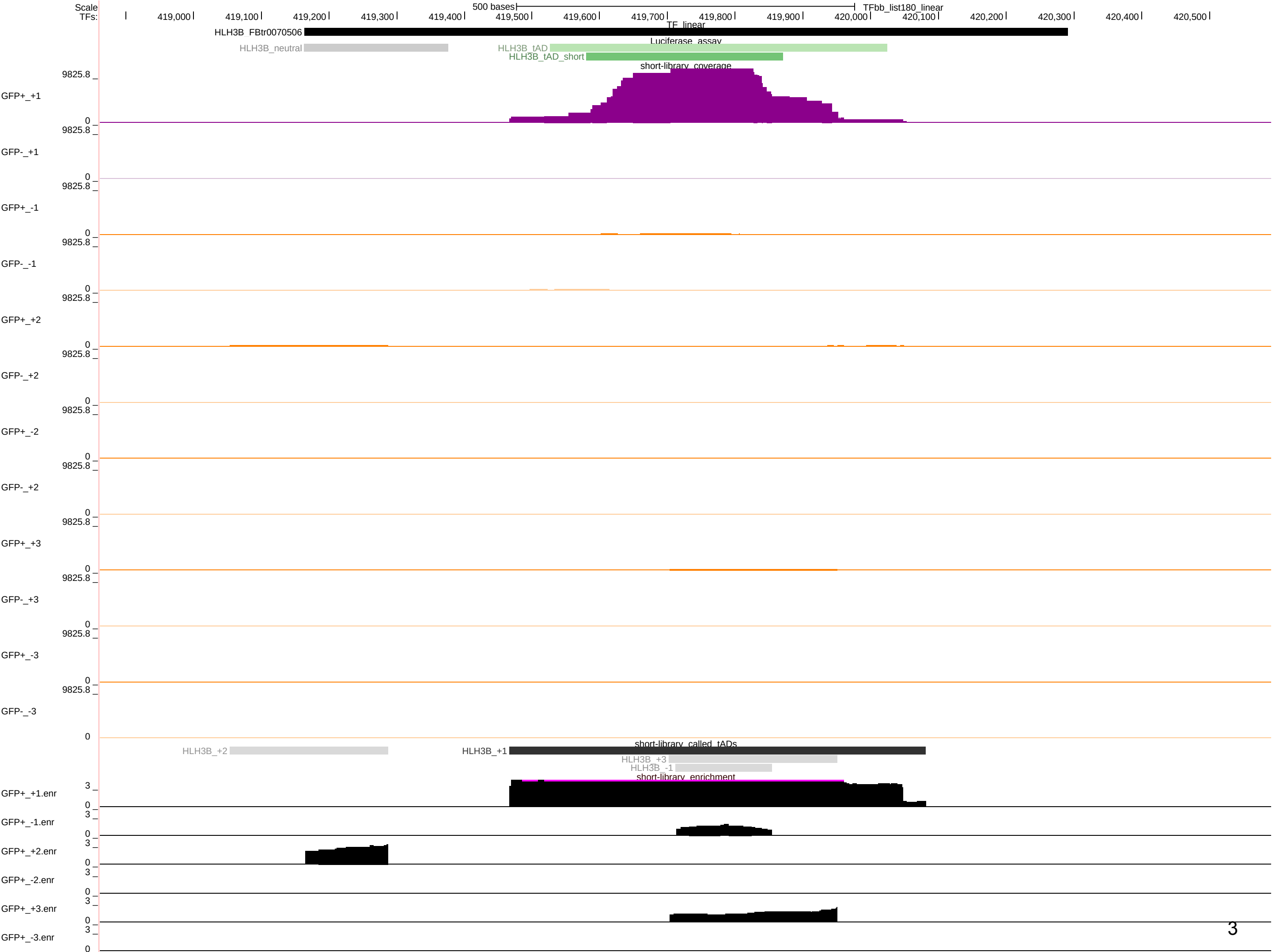
Scale
TFs:
500 bases
TFbb_list180_linear
419,000
419,100
419,200
419,300
419,400
419,500
419,600
419,700
419,800
419,900
420,000
420,100
420,200
420,300
420,400
420,500
TF_linear
HLH3B_FBtr0070506
Luciferase_assay
HLH3B_neutral
HLH3B_tAD
HLH3B_tAD_short
short-library_coverage
9825.8 _
GFP+_+1
0 _
GFP-_+1
GFP+_-1
GFP-_-1
GFP+_+2
GFP-_+2
GFP+_-2
GFP-_+2
GFP+_+3
GFP-_+3
GFP+_-3
GFP-_-3
short-library_called_tADs
HLH3B_+2
HLH3B_+1
HLH3B_+3
HLH3B_-1
short-library_enrichment
3 _
GFP+_+1.enr
GFP+_-1.enr
GFP+_+2.enr
GFP+_-2.enr
GFP+_+3.enr
GFP+_-3.enr

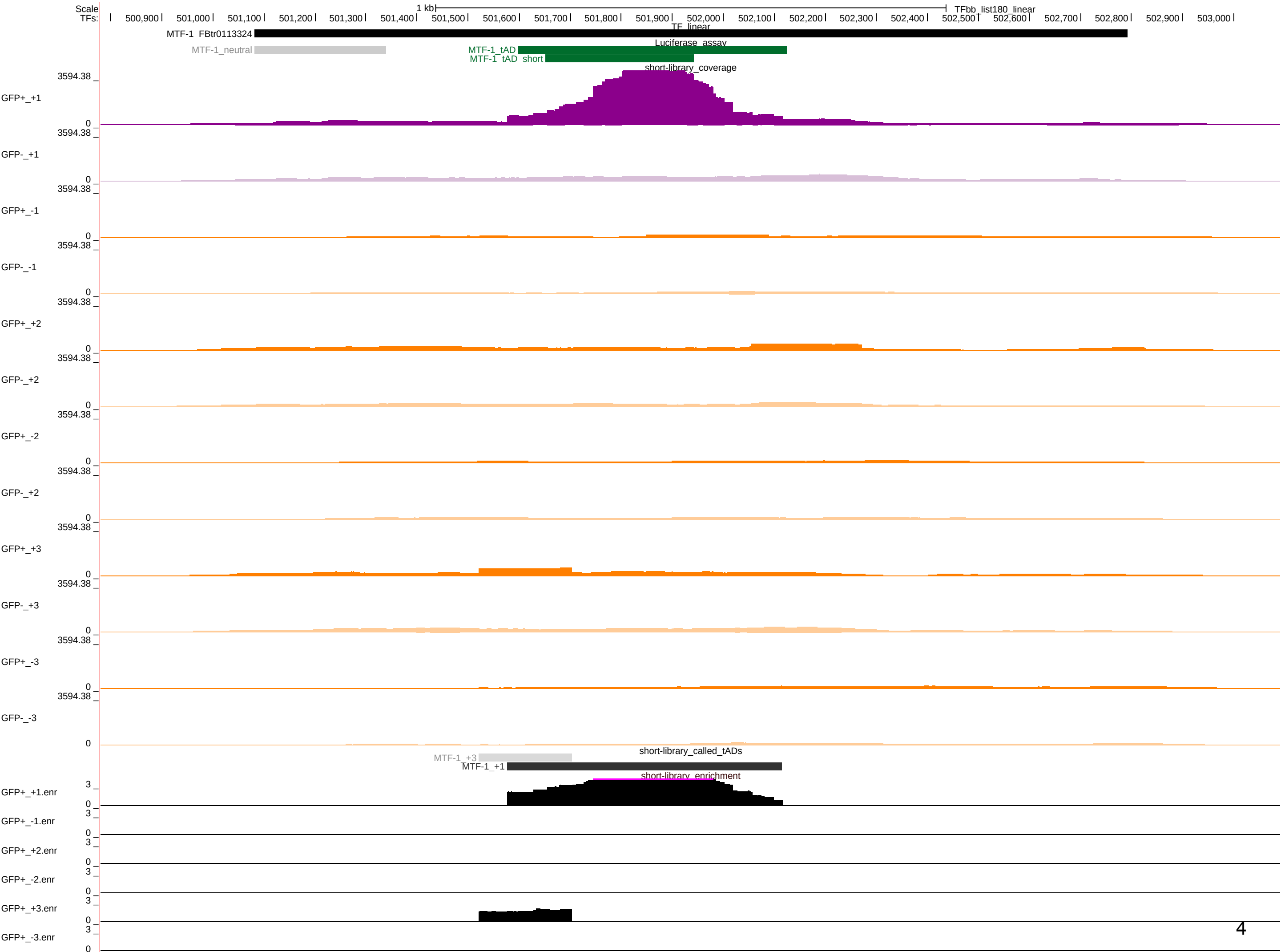

Scale
1 kb
TFs:
500,900
501,000
501,100
501,200
501,300
501,400
501,500
501,600
501,700
501,800
501,900
502,000
502,100
502,200
502,300
502,400
502,500
502,600
502,700
502,800
502,900
503,000
TFbb_list180_linear
TF_linear
MTF-1_FBtr0113324
Luciferase_assay
MTF-1_neutral
MTF-1_tAD
MTF-1_tAD_short
short-library_coverage
GFP+_+1
GFP-_+1
GFP+_-1
GFP-_-1
GFP+_+2
GFP-_+2
GFP+_-2
GFP-_+2
GFP+_+3
GFP-_+3
GFP+_-3
GFP-_-3
3594.38
0
short-library_called_tADs
MTF-1_+3
MTF-1_+1
short-library_enrichment
GFP+_+1.enr
GFP+_-1.enr
GFP+_+2.enr
GFP+_-2.enr
GFP+_+3.enr
GFP+_-3.enr
3
0

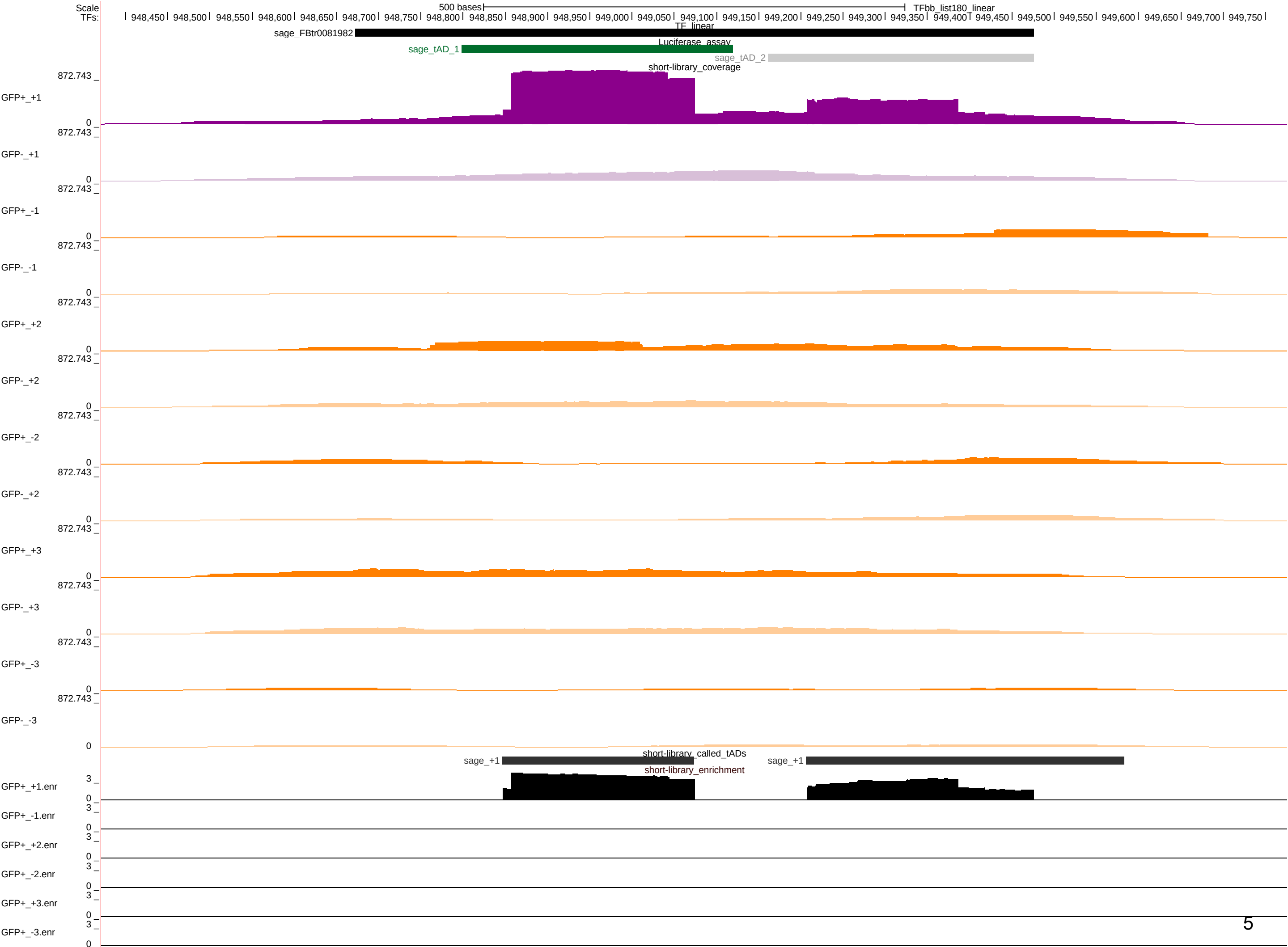
Scale
500 bases
TFs:
948,450
948,500
948,550
948,600
948,650
948,700
948,750
948,800
948,850
948,900
948,950
949,000
949,050
949,100
949,150
949,200
949,250
949,300
949,350
949,400
949,450
949,500
949,550
949,600
949,650
949,700
949,750
TFbb_list180_linear
TF_linear
sage FBtr0081982
Luciferase_assay
sage_tAD_1
sage_tAD_2
short-library_coverage
872.743
0
GFP+_+1
GFP-_+1
GFP+_-1
GFP-_-1
GFP+_+2
GFP-_+2
GFP+_-2
GFP-_+2
GFP+_+3
GFP-_+3
GFP+_-3
GFP-_-3
short-library_called_tADs
sage_+1
sage_+1
short-library_enrichment
3
GFP+_+1.enr
GFP+_-1.enr
GFP+_+2.enr
GFP+_-2.enr
GFP+_+3.enr
GFP+_-3.enr

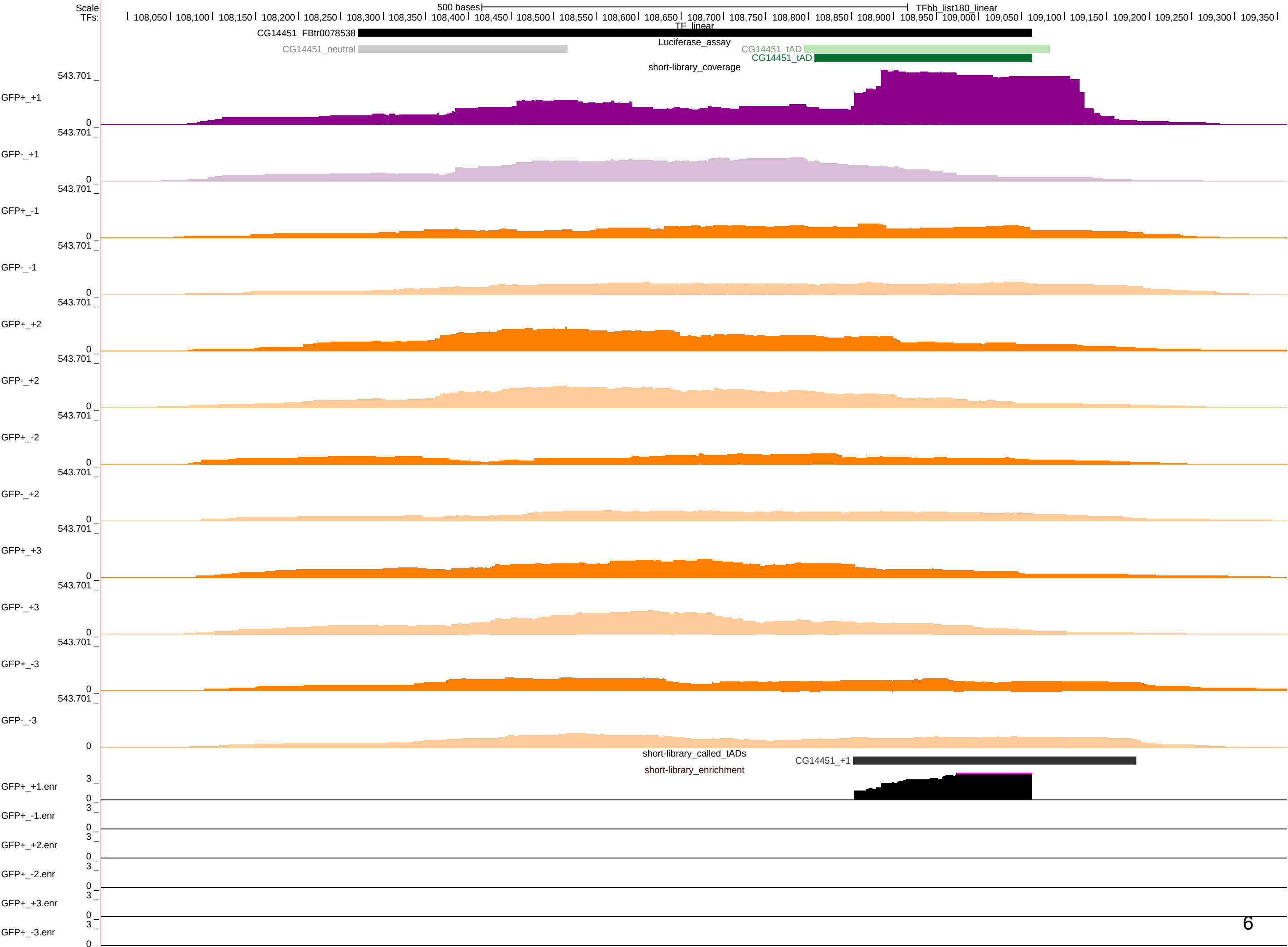

Scale
500 bases
TFbb_list180_linear
TFs:
108,050
108,100
108,150
108,200
108,250
108,300
108,350
108,400
108,450
108,500
108,550
108,600
108,650
108,700
108,750
108,800
108,850
108,900
108,950
109,000
109,050
109,100
109,150
109,200
109,250
109,300
109,350
TF_linear
CG14451 FBtr0078538
Luciferase_assay
CG14451_neutral
CG14451_tAD
CG14451_tAD
short-library_coverage
543.701
GFP+_+1
GFP-_+1
GFP+_-1
GFP-_-1
GFP+_+2
GFP-_+2
GFP+_-2
GFP-_+2
GFP+_+3
GFP-_+3
GFP+_-3
GFP-_-3
0
short-library_called_tADs
CG14451_+1
short-library_enrichment
3
GFP+_+1.enr
GFP+_-1.enr
GFP+_+2.enr
GFP+_-2.enr
GFP+_+3.enr
GFP+_-3.enr

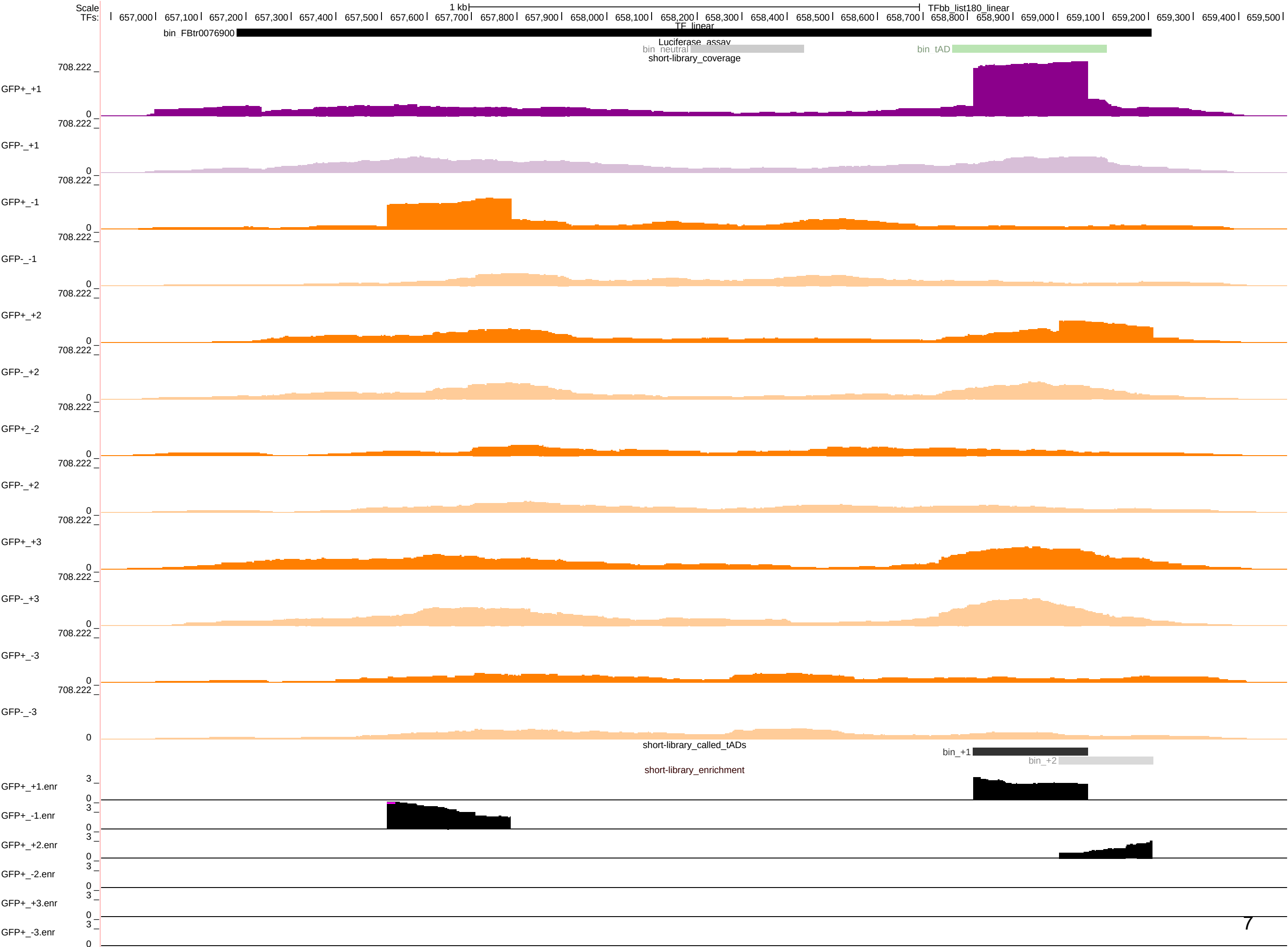

Scale
1 kb
TFbb_list180_linear
TFs:
657,000
657,100
657,200
657,300
657,400
657,500
657,600
657,700
657,800
657,900
658,000
658,100
658,200
658,300
658,400
658,500
658,600
658,700
658,800
658,900
659,000
659,100
659,200
659,300
659,400
659,500
TF_linear
bin_FBtr0076900
Luciferase_assay
bin_neutral
bin_tAD
short-library_coverage
GFP+_+1
GFP-_+1
GFP+_-1
GFP-_-1
GFP+_+2
GFP-_+2
GFP+_-2
GFP-_+2
GFP+_+3
GFP-_+3
GFP+_-3
GFP-_-3
708.222
0
short-library_called_tADs
bin_+1
bin_+2
short-library_enrichment
GFP+_+1.enr
GFP+_-1.enr
GFP+_+2.enr
GFP+_-2.enr
GFP+_+3.enr
GFP+_-3.enr
3
0

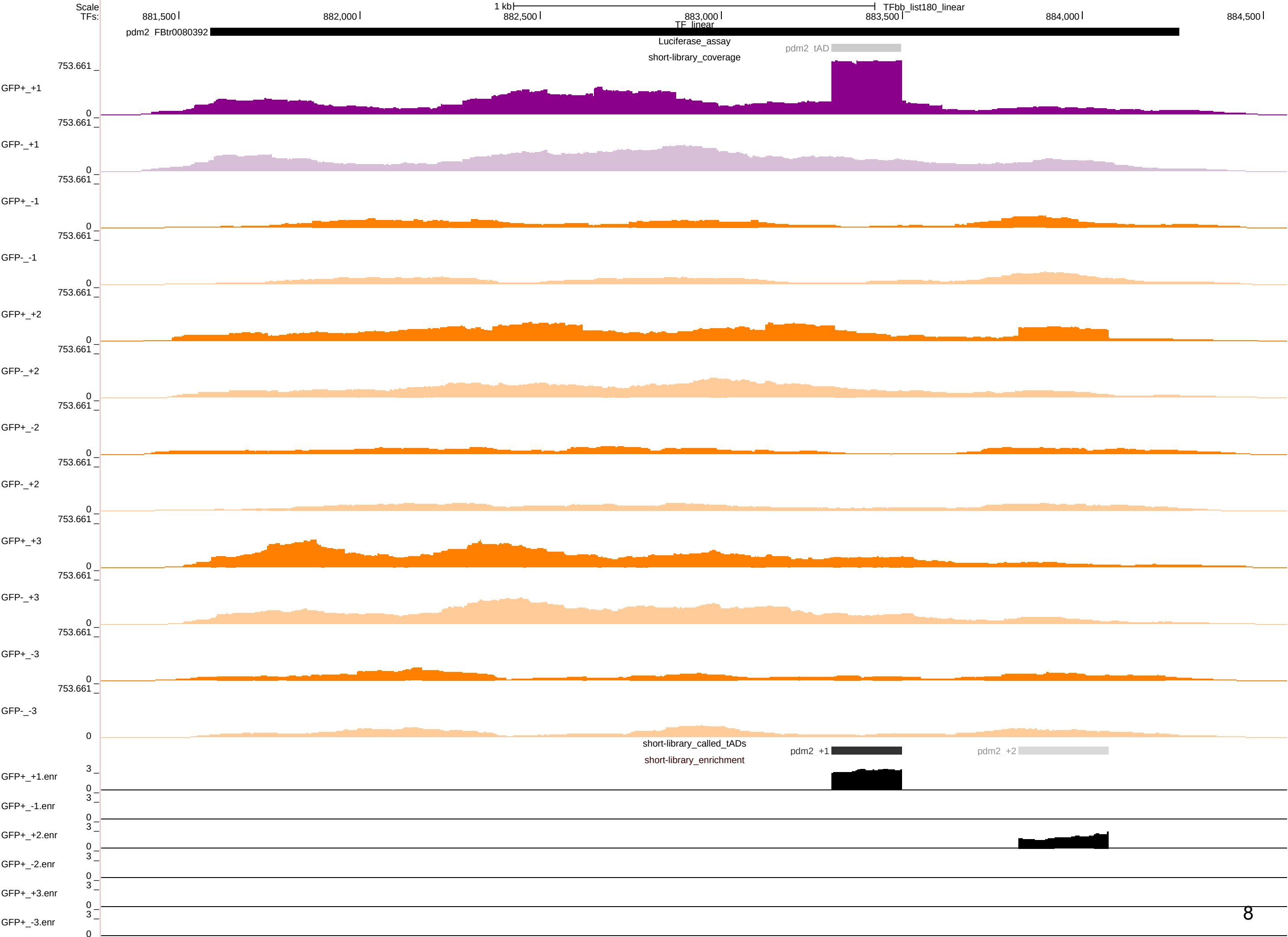
Scale
TFs:
1 kb
881,500
882,000
882,500
883,000
883,500
884,000
884,500
TFbb_list180_linear
TF_linear
pdm2_FBtr0080392
Luciferase_assay
pdm2_tAD
short-library_coverage
753.661
0
GFP+_+1
GFP-_+1
GFP+_-1
GFP-_-1
GFP+_+2
GFP-_+2
GFP+_-2
GFP-_+2
GFP+_+3
GFP-_+3
GFP+_-3
GFP-_-3
short-library_called_tADs
pdm2_+1
pdm2_+2
short-library_enrichment
3
GFP+_+1.enr
GFP+_-1.enr
GFP+_+2.enr
GFP+_-2.enr
GFP+_+3.enr
GFP+_-3.enr

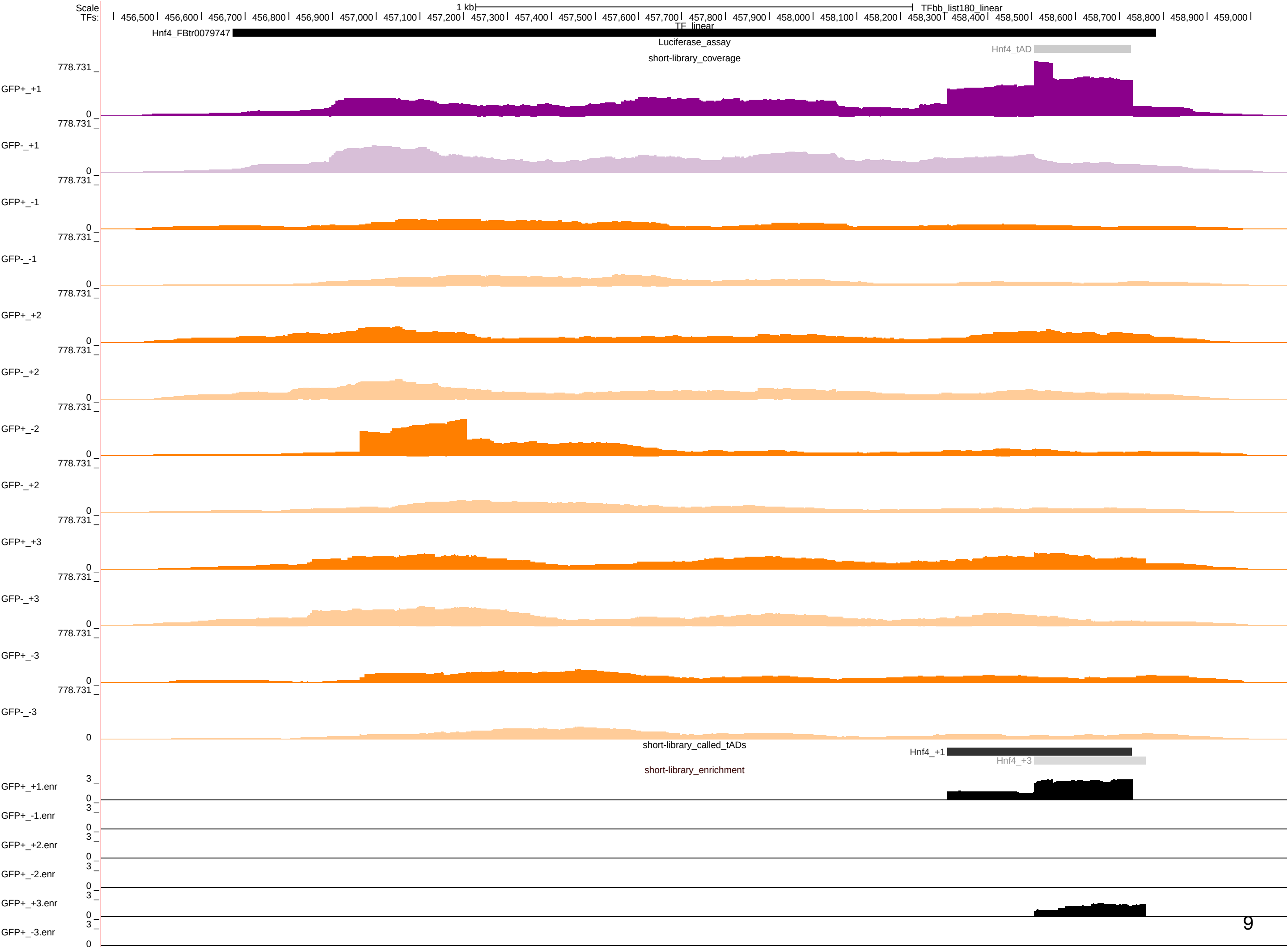

Scale
TFs:
1 kb
TFbb_list180_linear
456,500
456,600
456,700
456,800
456,900
457,000
457,100
457,200
457,300
457,400
457,500
457,600
457,700
457,800
457,900
458,000
458,100
458,200
458,300
458,400
458,500
458,600
458,700
458,800
458,900
459,000
TF_linear
Hnf4 FBtr0079747
Luciferase_assay
Hnf4 tAD
short-library_coverage
778.731
0
GFP+_+1
GFP-_+1
GFP+_-1
GFP-_-1
GFP+_+2
GFP-_+2
GFP+_-2
GFP-_+2
GFP+_+3
GFP-_+3
GFP+_-3
GFP-_-3
short-library_called_tADs
Hnf4_+1
Hnf4_+3
short-library_enrichment
3
GFP+_+1.enr
GFP+_-1.enr
GFP+_+2.enr
GFP+_-2.enr
GFP+_+3.enr
GFP+_-3.enr

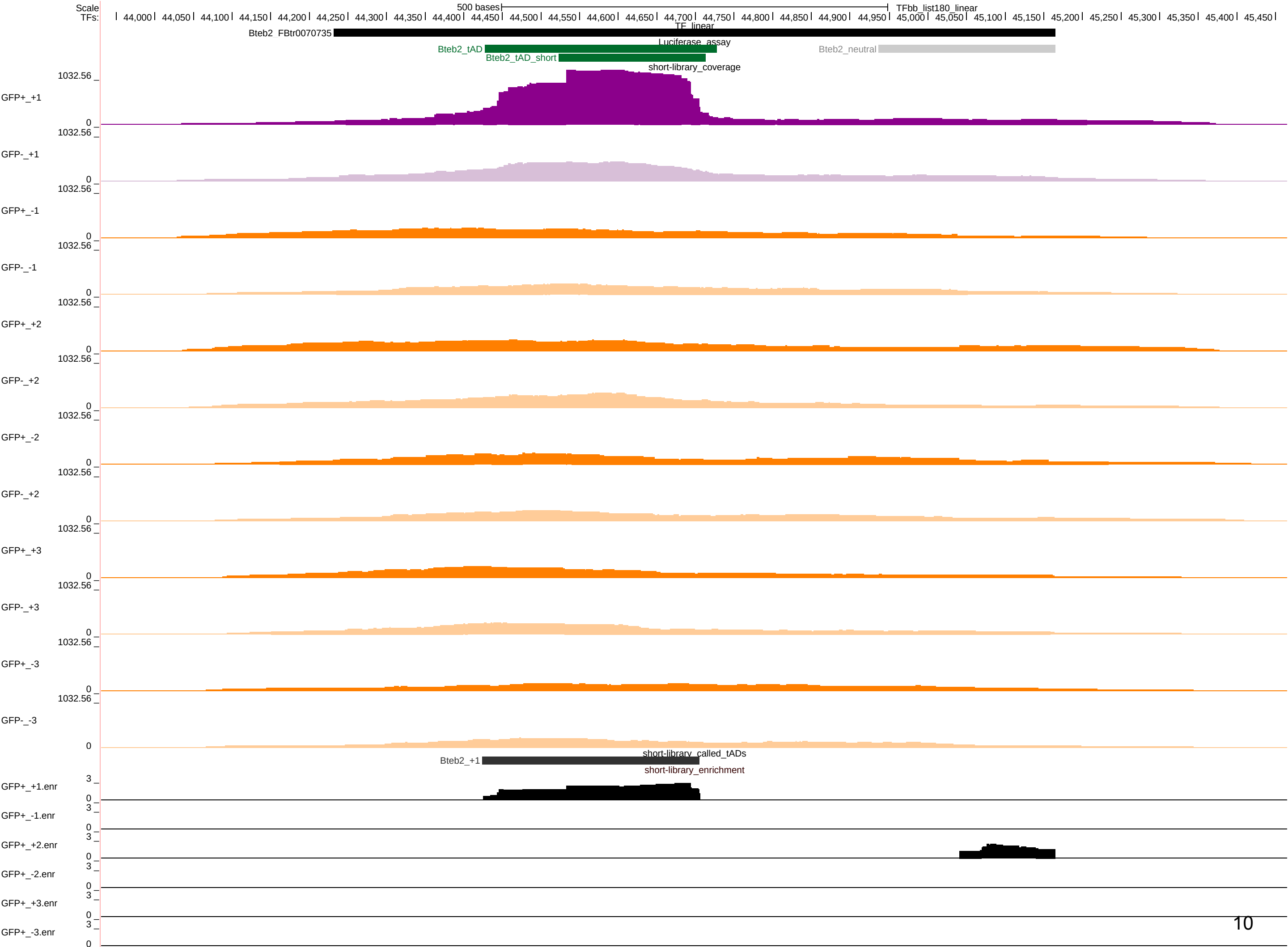

Scale
TFs:
500 bases
TFbb_list180_linear
44,000 44,050 44,100 44,150 44,200 44,250 44,300 44,350 44,400 44,450 44,500 44,550 44,600 44,650 44,700 44,750 44,800 44,850 44,900 44,950 45,000 45,050 45,100 45,150 45,200 45,250 45,300 45,350 45,400 45,450
TF_linear
Bteb2 FBtr0070735
Luciferase_assay
Bteb2_tAD
Bteb2_tAD_short
Bteb2_neutral
short-library_coverage
GFP+_+1
GFP-_+1
GFP+_-1
GFP-_-1
GFP+_+2
GFP-_+2
GFP+_-2
GFP-_+2
GFP+_+3
GFP-_+3
GFP+_-3
GFP-_-3
1032.56
0
short-library_called_tADs
Bteb2_+1
short-library_enrichment
GFP+_+1.enr
GFP+_-1.enr
GFP+_+2.enr
GFP+_-2.enr
GFP+_+3.enr
GFP+_-3.enr
3
0

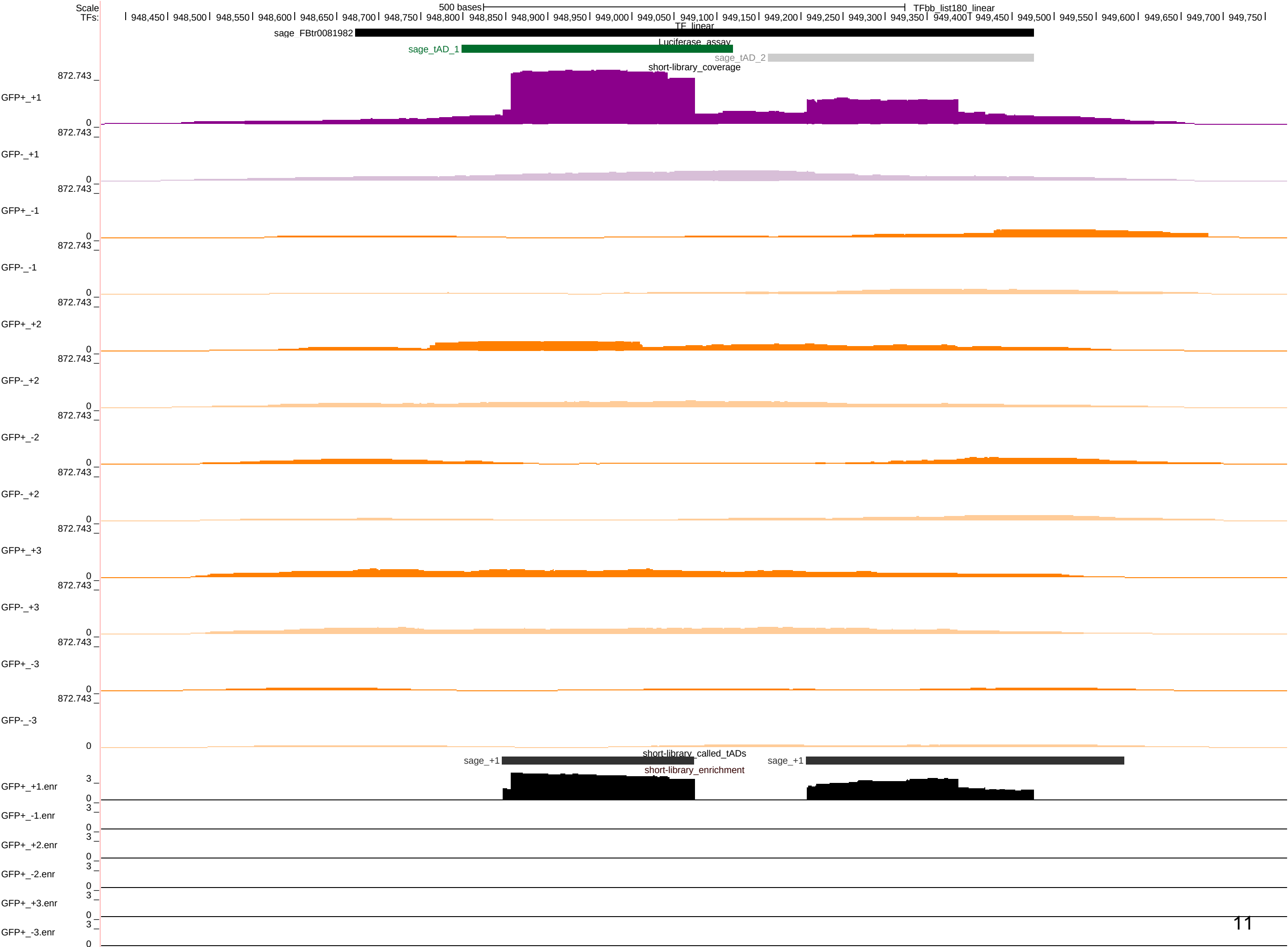

Scale
500 bases
TFbb_list180_linear
TFs:
948,450 948,500 948,550 948,600 948,650 948,700 948,750 948,800 948,850 948,900 948,950 949,000 949,050 949,100 949,150 949,200 949,250 949,300 949,350 949,400 949,450 949,500 949,550 949,600 949,650 949,700 949,750
TF_linear
sage FBtr0081982
Luciferase_assay
sage_tAD_1
sage_tAD_2
short-library_coverage
GFP+_+1
GFP-_+1
GFP+_-1
GFP-_-1
GFP+_+2
GFP-_+2
GFP+_-2
GFP-_+2
GFP+_+3
GFP-_+3
GFP+_-3
GFP-_-3
872.743
0
short-library_called_tADs
sage_+1
sage_+1
short-library_enrichment
GFP+_+1.enr
GFP+_-1.enr
GFP+_+2.enr
GFP+_-2.enr
GFP+_+3.enr
GFP+_-3.enr
3
0

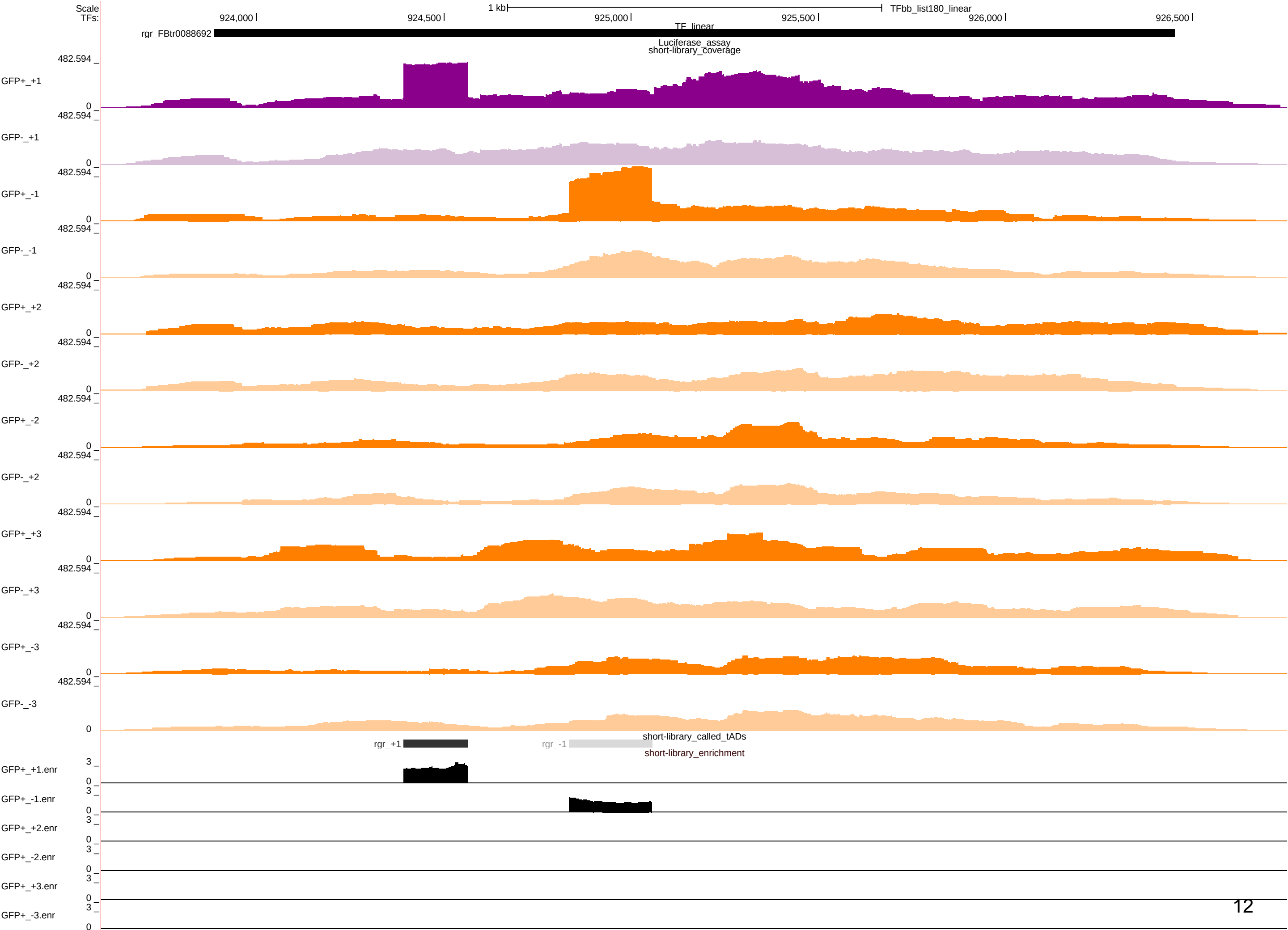

Scale
TFs:
1 kb
TFbb_list180_linear
924,000
924,500
925,000
925,500
926,000
926,500
TF_linear
rgr_FBtr0088692
Luciferase_assay
short-library_coverage
482.594
0
GFP+_+1
GFP-_+1
GFP+_-1
GFP-_-1
GFP+_+2
GFP-_+2
GFP+_-2
GFP-_+2
GFP+_+3
GFP-_+3
GFP+_-3
GFP-_-3
short-library_called_tADs
rgr_+1
rgr_-1
short-library_enrichment
3
GFP+_+1.enr
GFP+_-1.enr
GFP+_+2.enr
GFP+_-2.enr
GFP+_+3.enr
GFP+_-3.enr

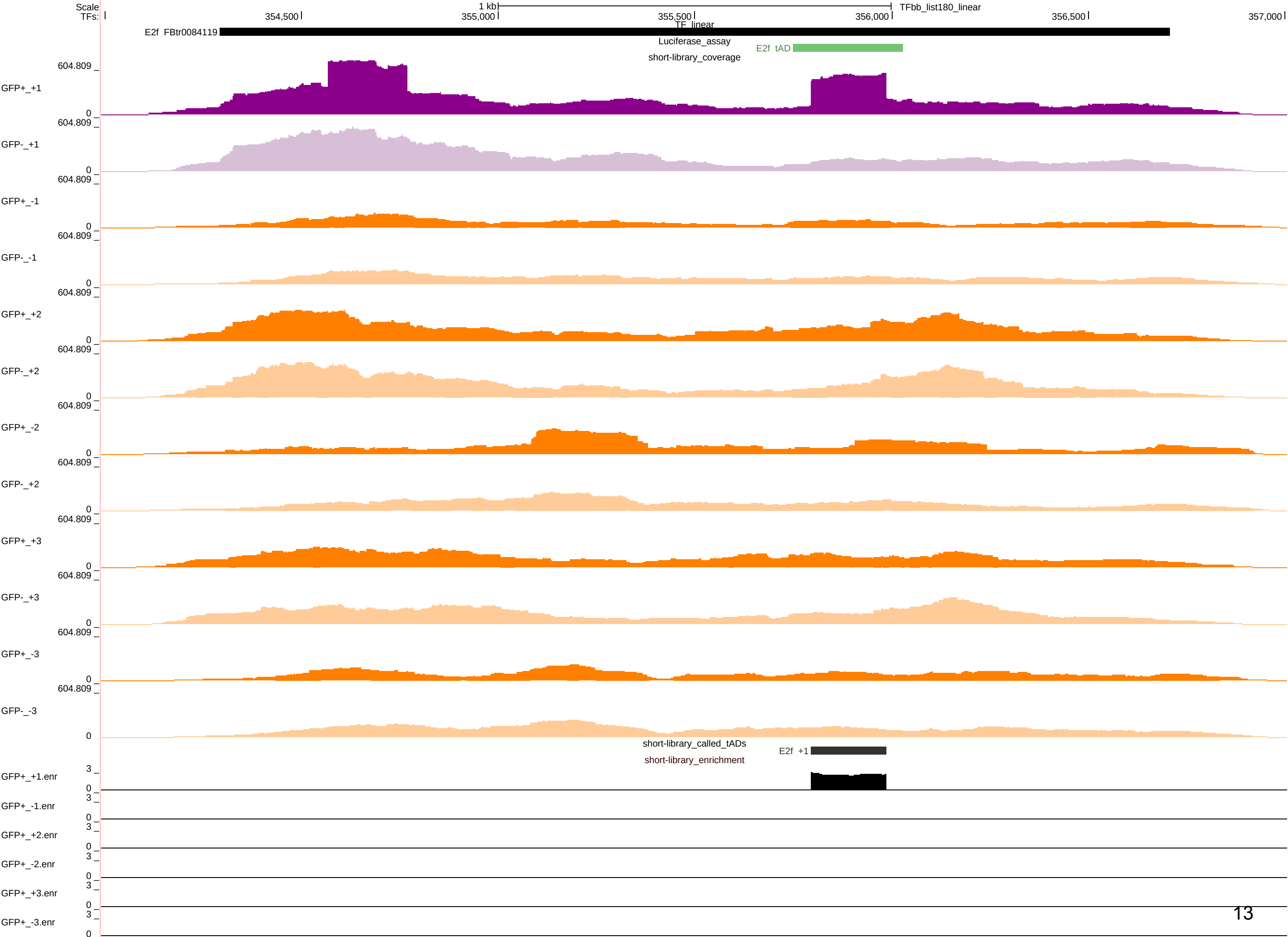
Scale
1 kb
TFs:
354,500
355,000
355,500
356,000
356,500
357,000
TFbb_list180_linear
TF_linear
E2f_FBtr0084119
Luciferase_assay
E2f_tAD
short-library_coverage
604.809
0
GFP+_+1
GFP-_+1
GFP+_-1
GFP-_-1
GFP+_+2
GFP-_+2
GFP+_-2
GFP-_+2
GFP+_+3
GFP-_+3
GFP+_-3
GFP-_-3
short-library_called_tADs
E2f_+1
short-library_enrichment
3
GFP+_+1.enr
GFP+_-1.enr
GFP+_+2.enr
GFP+_-2.enr
GFP+_+3.enr
GFP+_-3.enr

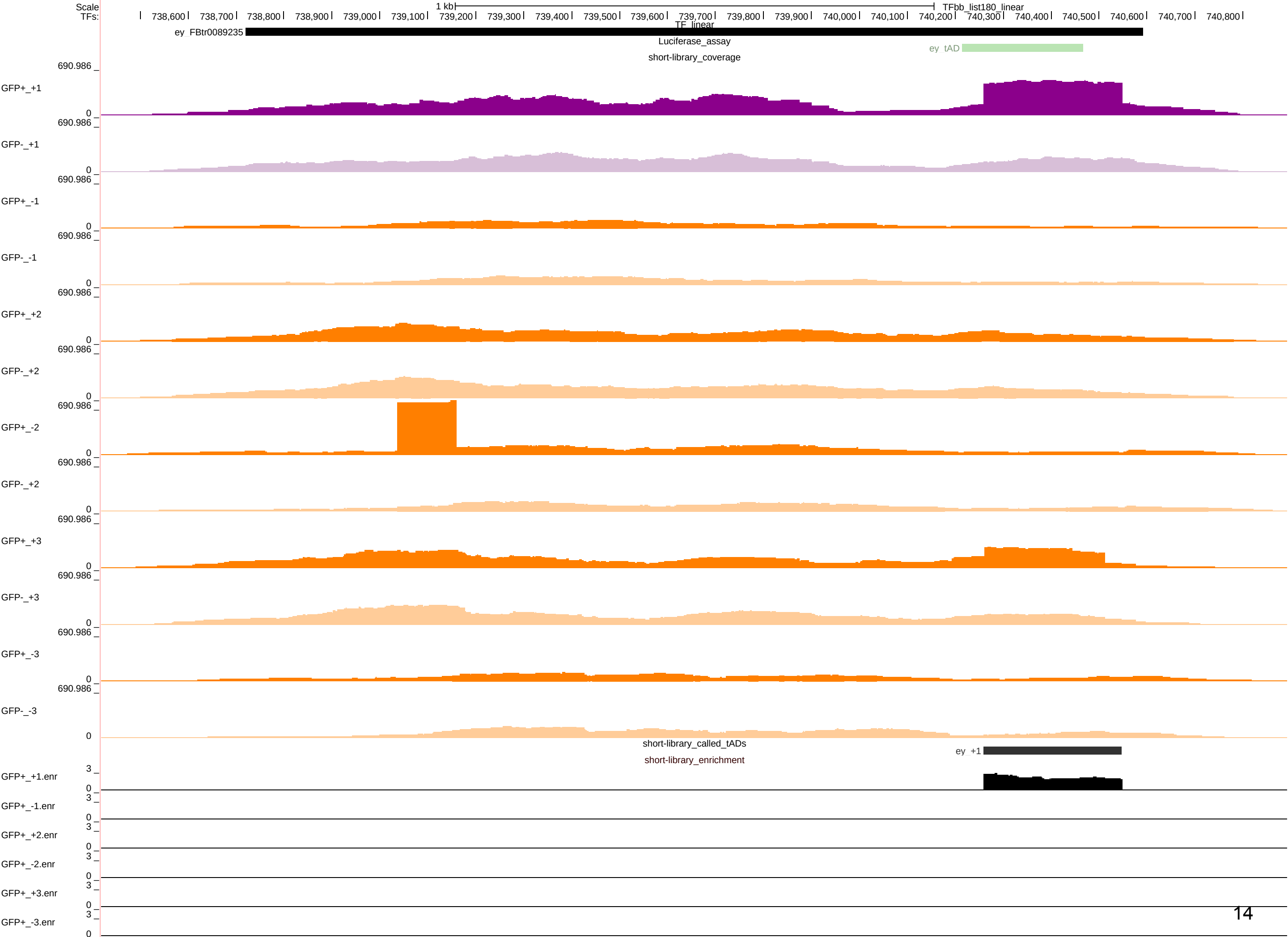
Scale
TFs:
1 kb
738,600
738,700
738,800
738,900
739,000
739,100
739,200
739,300
739,400
739,500
739,600
739,700
739,800
739,900
740,000
740,100
740,200
740,300
740,400
740,500
740,600
740,700
740,800
TFbb_list180_linear
TF_linear
ey_FBtr0089235
Luciferase_assay
ey_tAD
short-library_coverage
GFP+_+1
GFP-_+1
GFP+_-1
GFP-_-1
GFP+_+2
GFP-_+2
GFP+_-2
GFP-_+2
GFP+_+3
GFP-_+3
GFP+_-3
GFP-_-3
690.986
0
short-library_called_tADs
ey_+1
short-library_enrichment
GFP+_+1.enr
GFP+_-1.enr
GFP+_+2.enr
GFP+_-2.enr
GFP+_+3.enr
GFP+_-3.enr
3
0

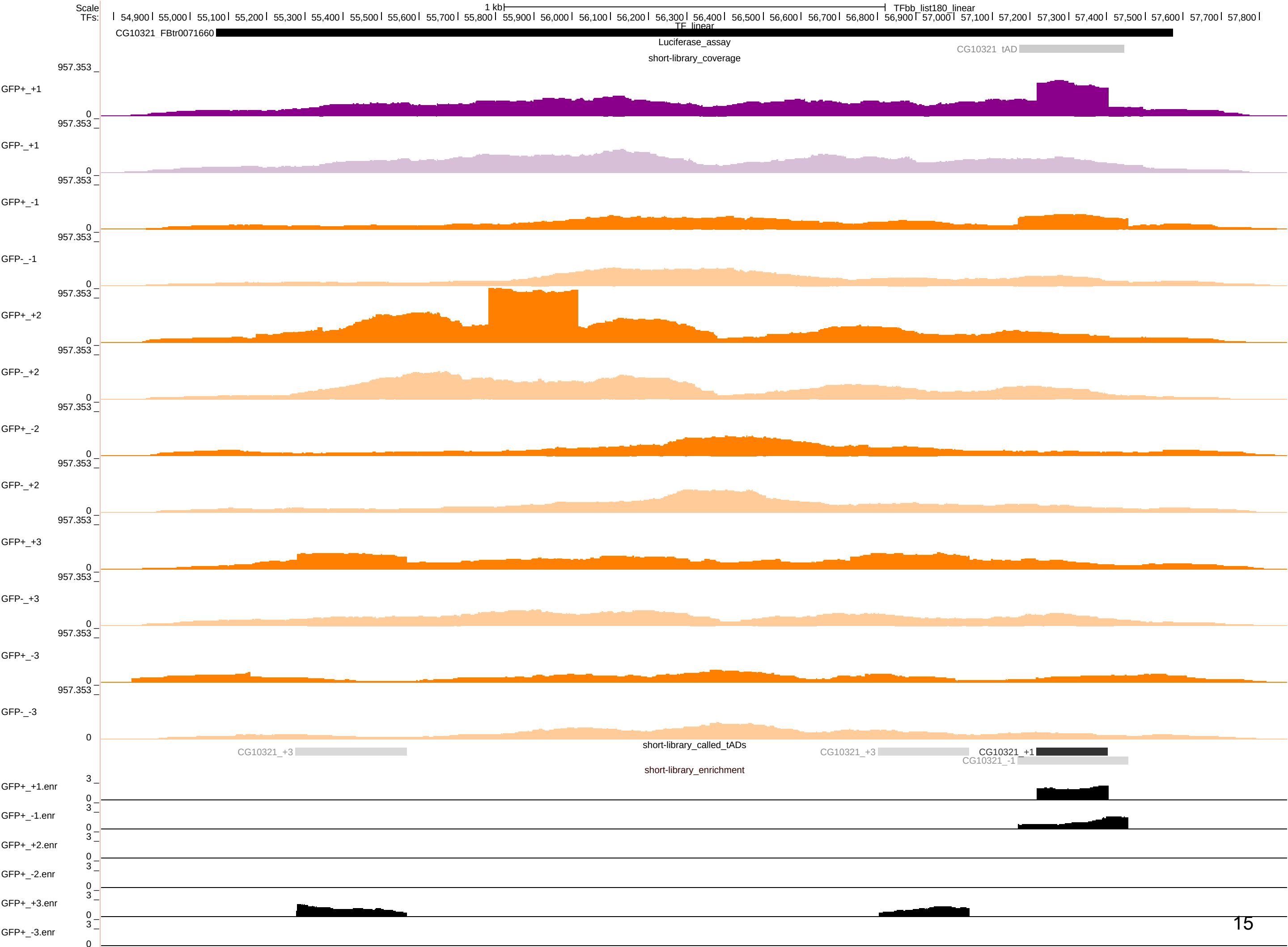
Scale
1 kb
TFbb_list180_linear
TFs:
54,900
55,000
55,100
55,200
55,300
55,400
55,500
55,600
55,700
55,800
55,900
56,000
56,100
56,200
56,300
56,400
56,500
56,600
56,700
56,800
56,900
57,000
57,100
57,200
57,300
57,400
57,500
57,600
57,700
57,800
TF_linear
CG10321 FBtr0071660
Luciferase_assay
CG10321 tAD
short-library_coverage
957.353
0
GFP+_+1
GFP-_+1
GFP+_-1
GFP-_-1
GFP+_+2
GFP-_+2
GFP+_-2
GFP-_+2
GFP+_+3
GFP-_+3
GFP+_-3
GFP-_-3
short-library_called_tADs
CG10321_+3
CG10321_+3
CG10321_+1
CG10321_-1
short-library_enrichment
3
0
GFP+_+1.enr
GFP+_-1.enr
GFP+_+2.enr
GFP+_-2.enr
GFP+_+3.enr
GFP+_-3.enr

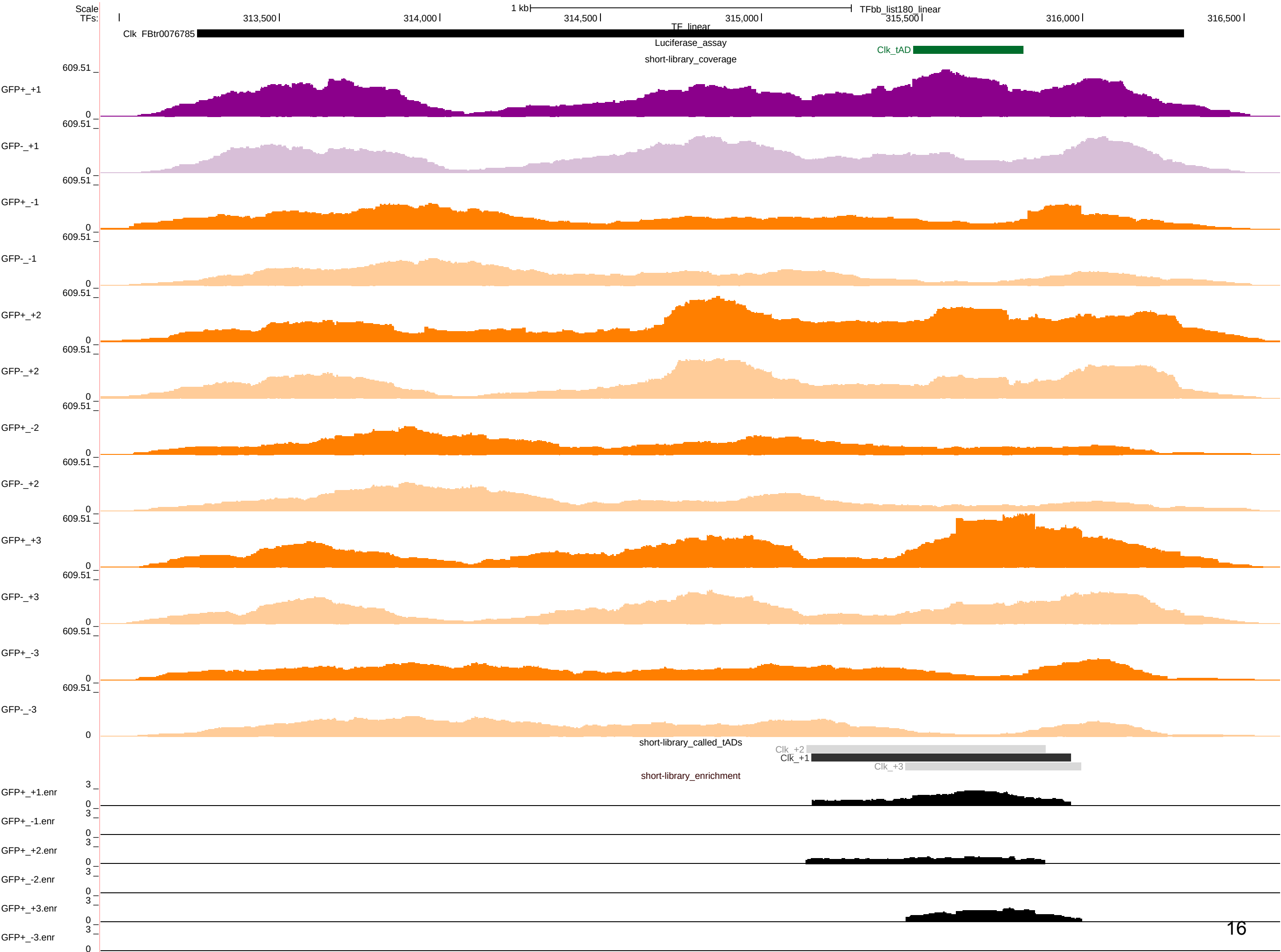
Scale
TFs:
1 kb
313,500
314,000
314,500
315,000
315,500
316,000
316,500
TFbb_list180_linear
TF_linear
Clk FBtr0076785
Luciferase_assay
Clk_tAD
short-library_coverage
609.51
0
GFP+_+1
GFP-_+1
GFP+_-1
GFP-_-1
GFP+_+2
GFP-_+2
GFP+_-2
GFP-_+2
GFP+_+3
GFP-_+3
GFP+_-3
GFP-_-3
short-library_called_tADs
Clk_+2
Clk_+1
Clk_+3
short-library_enrichment
3
GFP+_+1.enr
GFP+_-1.enr
GFP+_+2.enr
GFP+_-2.enr
GFP+_+3.enr
GFP+_-3.enr

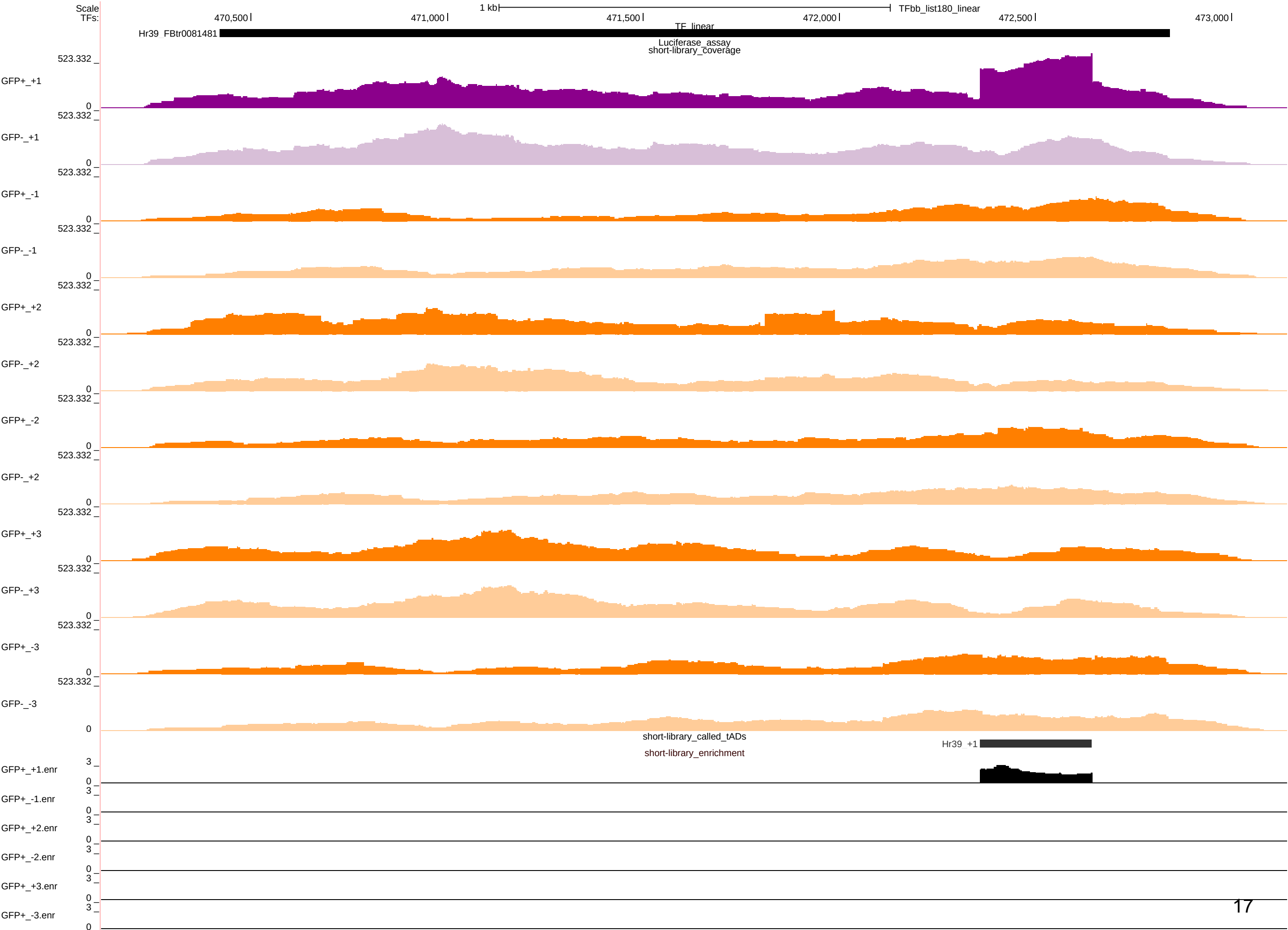
Scale
TFs:
1 kb
TFbb_list180_linear
470,500
471,000
471,500
472,000
472,500
473,000
TF_linear
Hr39_FBtr0081481
Luciferase_assay
short-library_coverage
523.332
0
GFP+_+1
GFP-_+1
GFP+_-1
GFP-_-1
GFP+_+2
GFP-_+2
GFP+_-2
GFP-_+2
GFP+_+3
GFP-_+3
GFP+_-3
GFP-_-3
short-library_called_tADs
Hr39_+1
short-library_enrichment
3
GFP+_+1.enr
GFP+_-1.enr
GFP+_+2.enr
GFP+_-2.enr
GFP+_+3.enr
GFP+_-3.enr

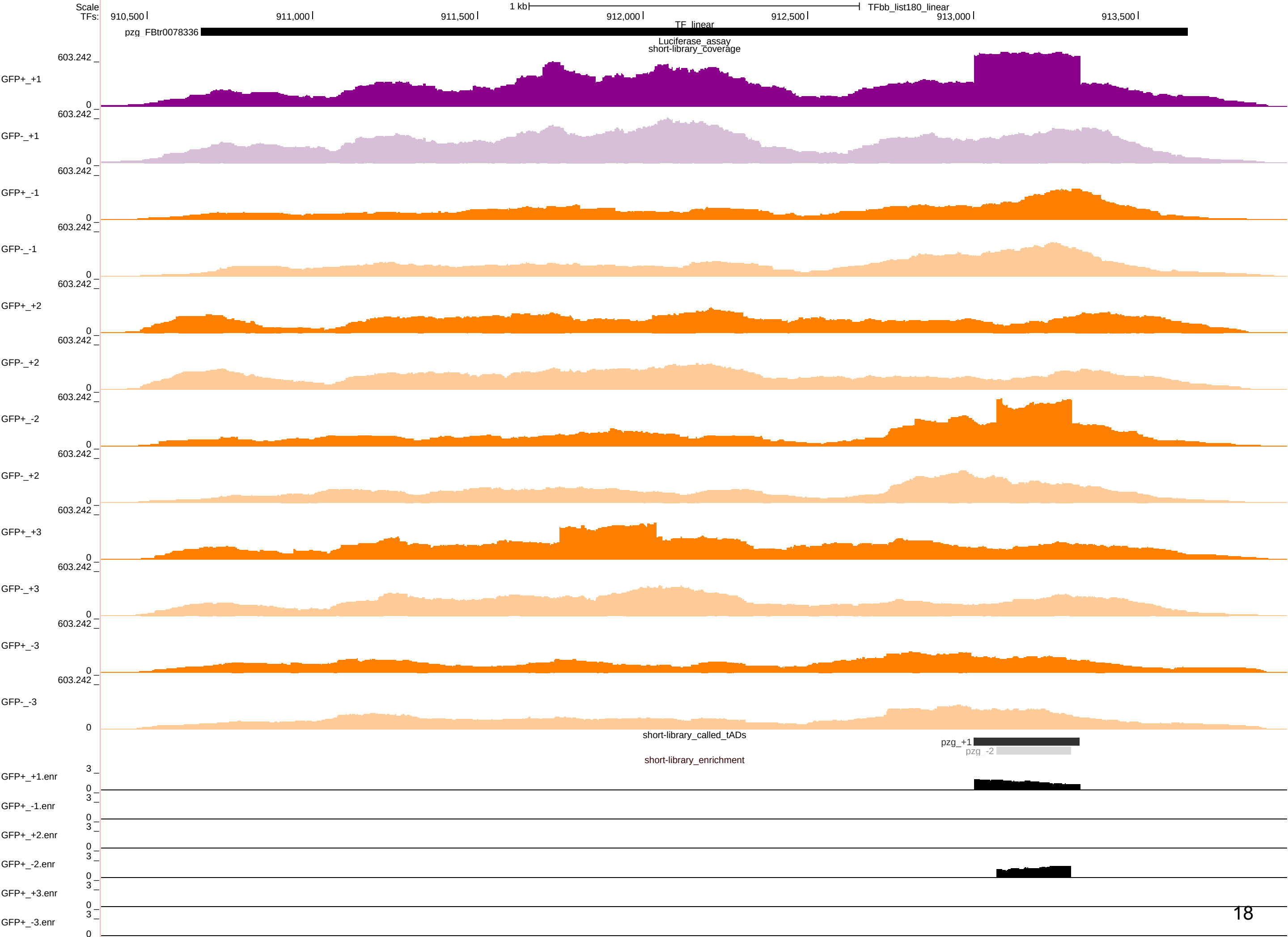

Scale
TFs:
1 kb
TFbb_list180_linear
910,500
911,000
911,500
912,000
912,500
913,000
913,500
TF_linear
pzg_FBtr0078336
Luciferase_assay
short-library_coverage
603.242 _
GFP+_+1
0 _
603.242 _
GFP-_+1
0 _
603.242 _
GFP+_-1
0 _
603.242 _
GFP-_-1
0 _
603.242 _
GFP+_+2
0 _
603.242 _
GFP-_+2
0 _
603.242 _
GFP+_-2
0 _
603.242 _
GFP-_+2
0 _
603.242 _
GFP+_+3
0 _
603.242 _
GFP-_+3
0 _
603.242 _
GFP+_-3
0 _
603.242 _
GFP-_-3
0
short-library_called_tADs
pzg_+1
pzg_-2
short-library_enrichment
3 _
GFP+_+1.enr
0 _
3 _
GFP+_-1.enr
0 _
3 _
GFP+_+2.enr
0 _
3 _
GFP+_-2.enr
0 _
3 _
GFP+_+3.enr
0 _
3 _
GFP+_-3.enr
0

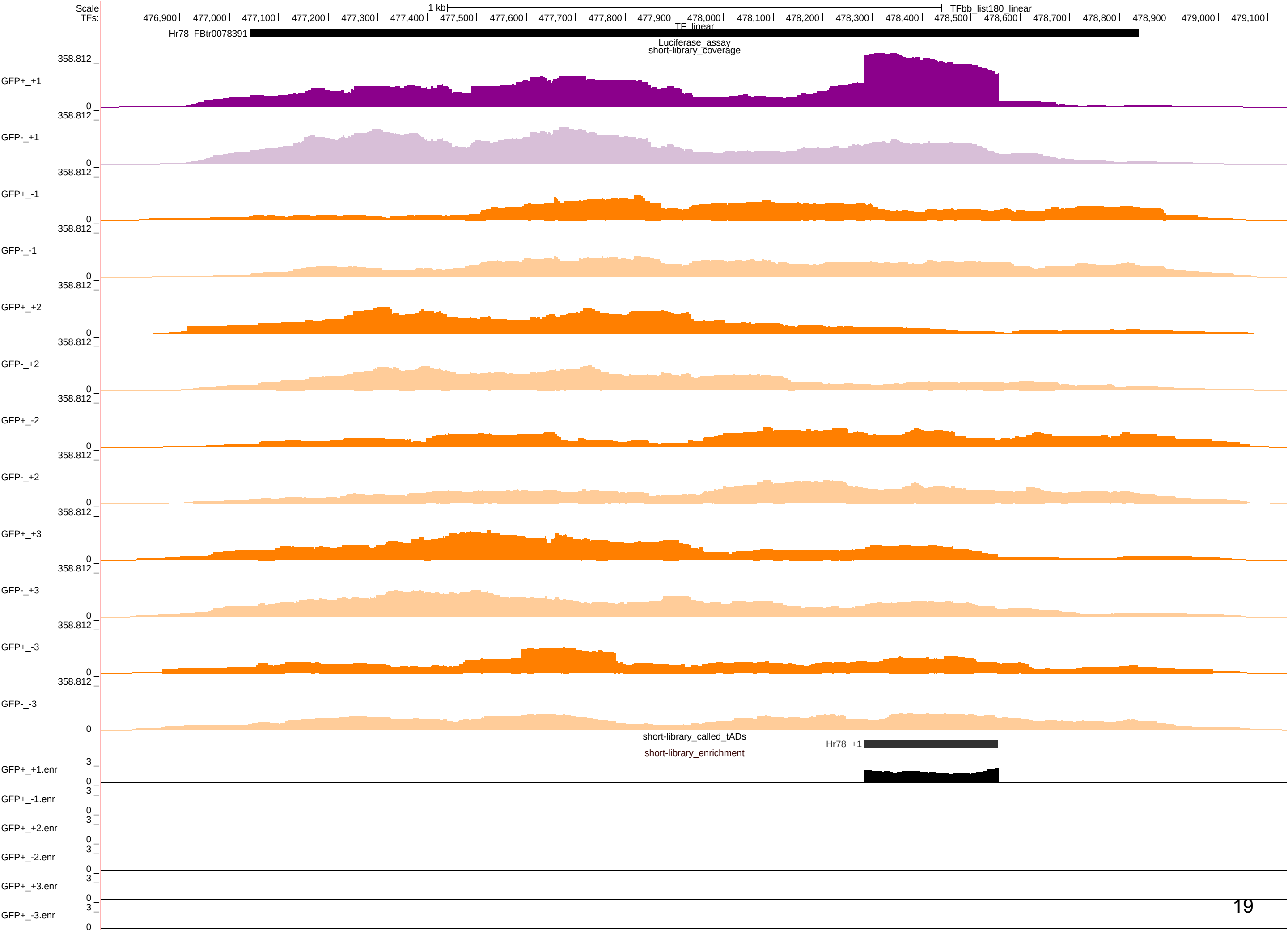
Scale
TFs:
1 kb
TFbb_list180_linear
476,900 477,000 477,100 477,200 477,300 477,400 477,500 477,600 477,700 477,800 477,900 478,000 478,100 478,200 478,300 478,400 478,500 478,600 478,700 478,800 478,900 479,000 479,100
TF_linear
Hr78 FBtr0078391
Luciferase_assay
short-library_coverage
358.812
0
GFP+_+1
GFP-_+1
GFP+_-1
GFP-_-1
GFP+_+2
GFP-_+2
GFP+_-2
GFP-_+2
GFP+_+3
GFP-_+3
GFP+_-3
GFP-_-3
short-library_called_tADs
Hr78_+1
short-library_enrichment
3
GFP+_+1.enr
GFP+_-1.enr
GFP+_+2.enr
GFP+_-2.enr
GFP+_+3.enr
GFP+_-3.enr

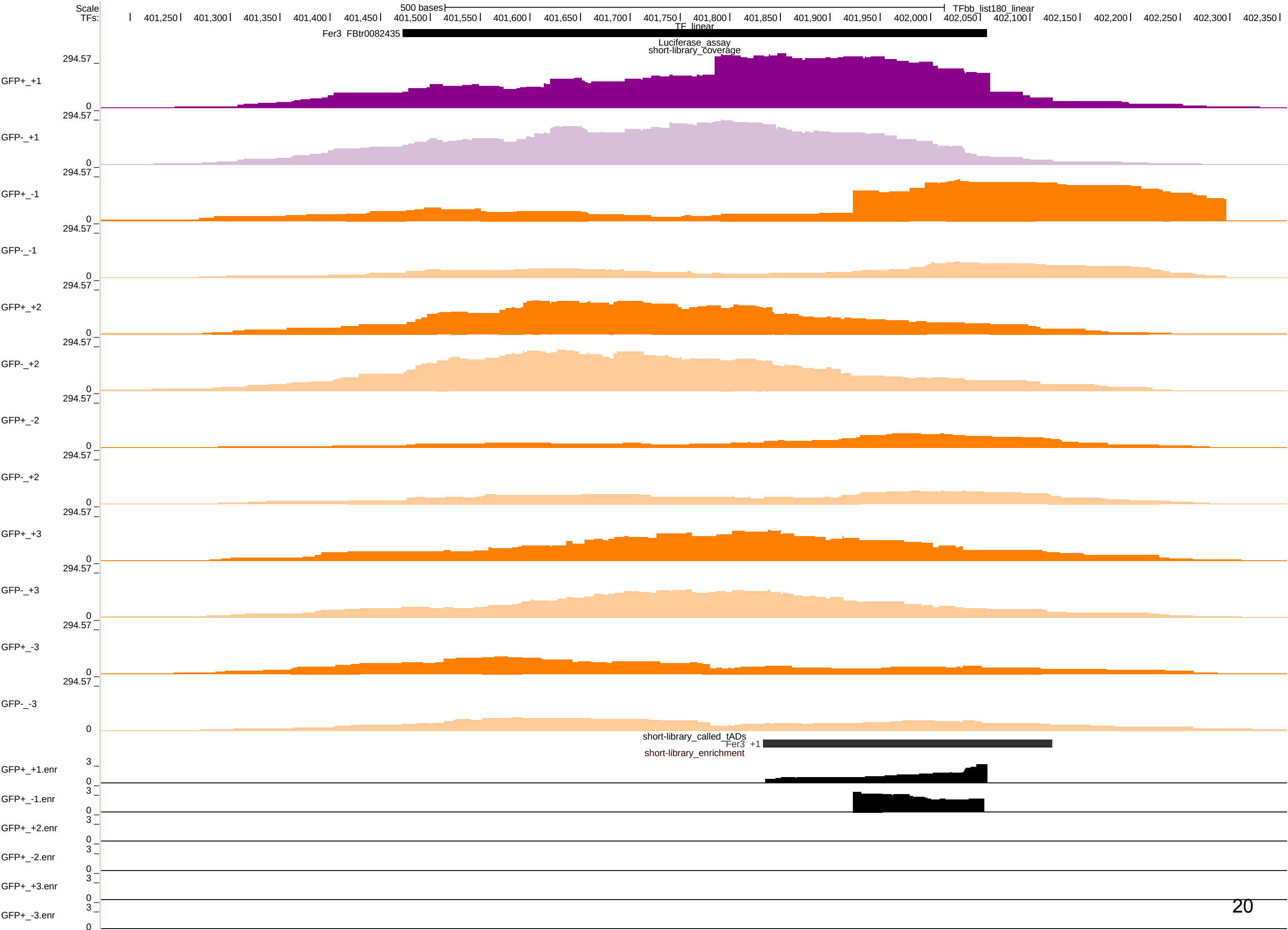

Scale
500 bases
TFbb_list180_linear
TFs:
401,250
401,300
401,350
401,400
401,450
401,500
401,550
401,600
401,650
401,700
401,750
401,800
401,850
401,900
401,950
402,000
402,050
402,100
402,150
402,200
402,250
402,300
402,350
TF_linear
Fer3 FBtr0082435
Luciferase_assay
short-library_coverage
GFP+_+1
GFP-_+1
GFP+_-1
GFP-_-1
GFP+_+2
GFP-_+2
GFP+_-2
GFP-_+2
GFP+_+3
GFP-_+3
GFP+_-3
GFP-_-3
294.57
0
short-library_called_tADs
Fer3 +1
short-library_enrichment
GFP+_+1.enr
GFP+_-1.enr
GFP+_+2.enr
GFP+_-2.enr
GFP+_+3.enr
GFP+_-3.enr
3
0

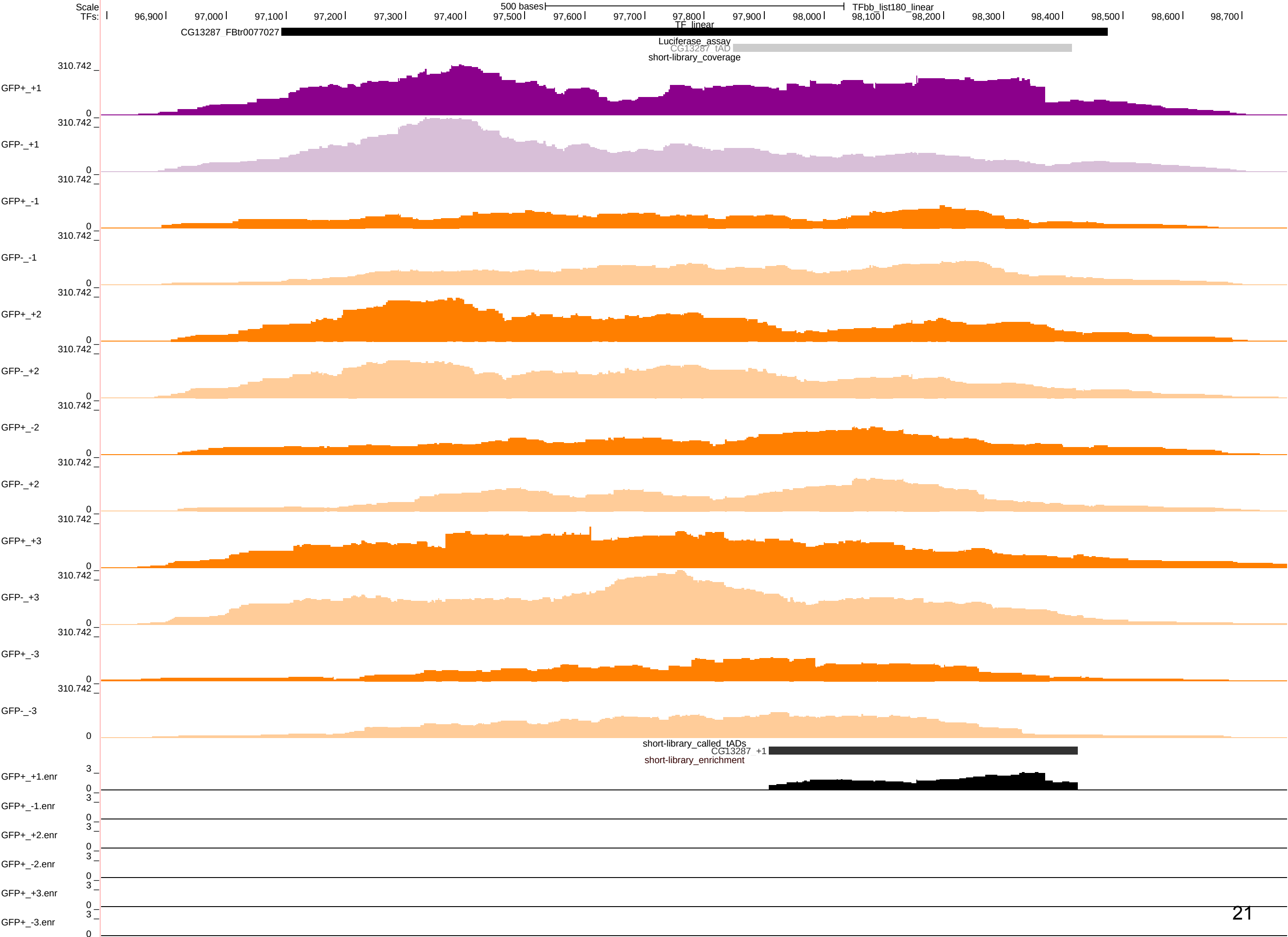

Scale
TFs:
500 bases
96,900
97,000
97,100
97,200
97,300
97,400
97,500
97,600
97,700
97,800
97,900
98,000
98,100
98,200
98,300
98,400
98,500
98,600
98,700
TFbb_list180_linear
TF_linear
CG13287_FBtr0077027
Luciferase_assay
CG13287_tAD
short-library_coverage
GFP+_+1
GFP-_+1
GFP+_-1
GFP-_-1
GFP+_+2
GFP-_+2
GFP+_-2
GFP-_+2
GFP+_+3
GFP-_+3
GFP+_-3
GFP-_-3
310.742
0
short-library_called_tADs
CG13287_+1
short-library_enrichment
GFP+_+1.enr
GFP+_-1.enr
GFP+_+2.enr
GFP+_-2.enr
GFP+_+3.enr
GFP+_-3.enr
3
0

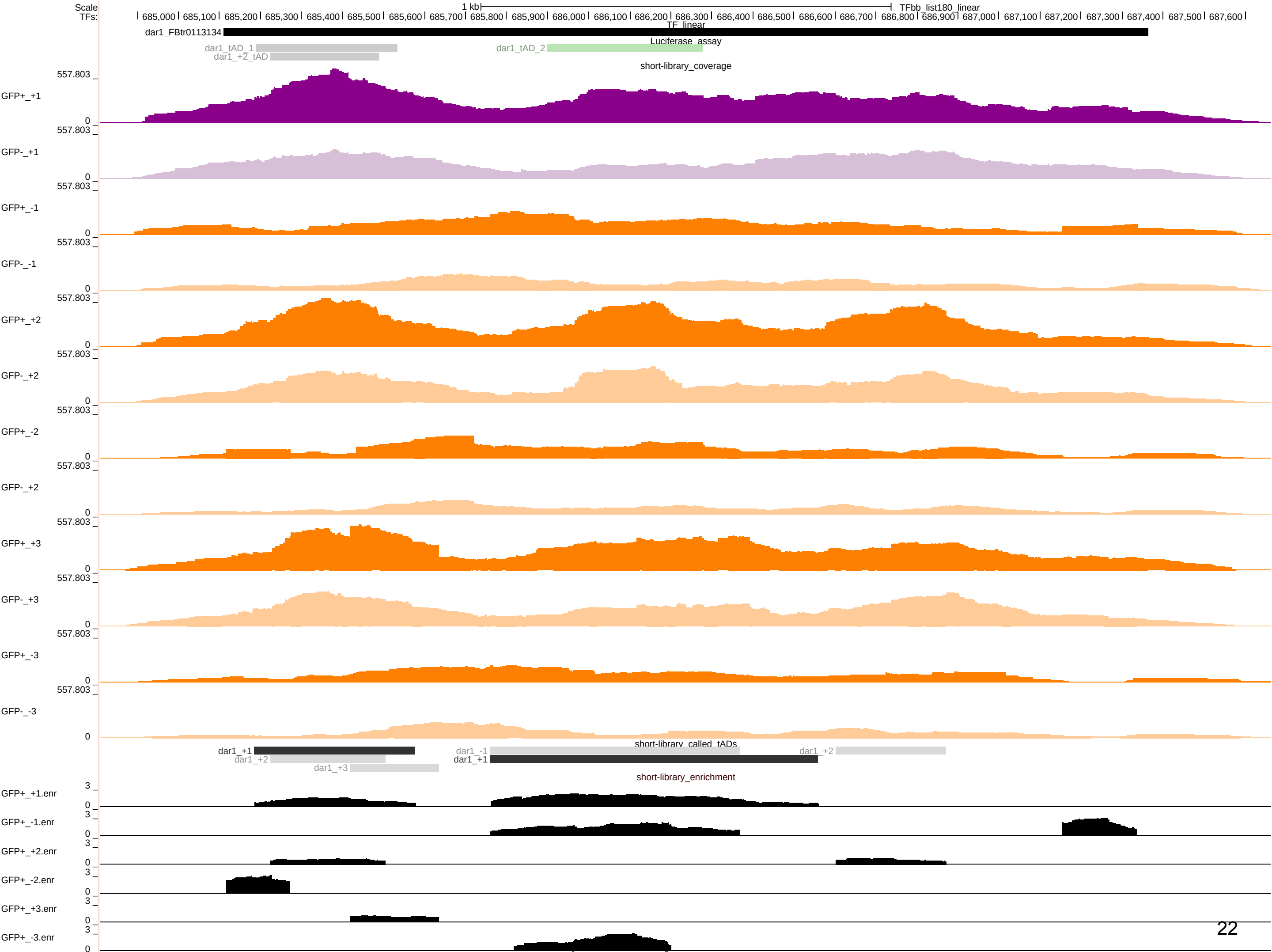

Scale
TFs:
1 kb
TFbb_list180_linear
685,000 685,100 685,200 685,300 685,400 685,500 685,600 685,700 685,800 685,900 686,000 686,100 686,200 686,300 686,400 686,500 686,600 686,700 686,800 686,900 687,000 687,100 687,200 687,300 687,400 687,500 687,600
TF_linear
dar1 FBtr0113134
Luciferase_assay
dar1_tAD_1
dar1_+2_tAD
dar1_tAD_2
short-library_coverage
GFP+_+1
GFP-_+1
GFP+_-1
GFP-_-1
GFP+_+2
GFP-_+2
GFP+_-2
GFP-_+2
GFP+_+3
GFP-_+3
GFP+_-3
GFP-_-3
557.803
0
short-library_called_tADs
dar1_+1
dar1_+2
dar1_+3
dar1_-1
dar1_+1
dar1_+2
short-library_enrichment
GFP+_+1.enr
GFP+_-1.enr
GFP+_+2.enr
GFP+_-2.enr
GFP+_+3.enr
GFP+_-3.enr
3
0

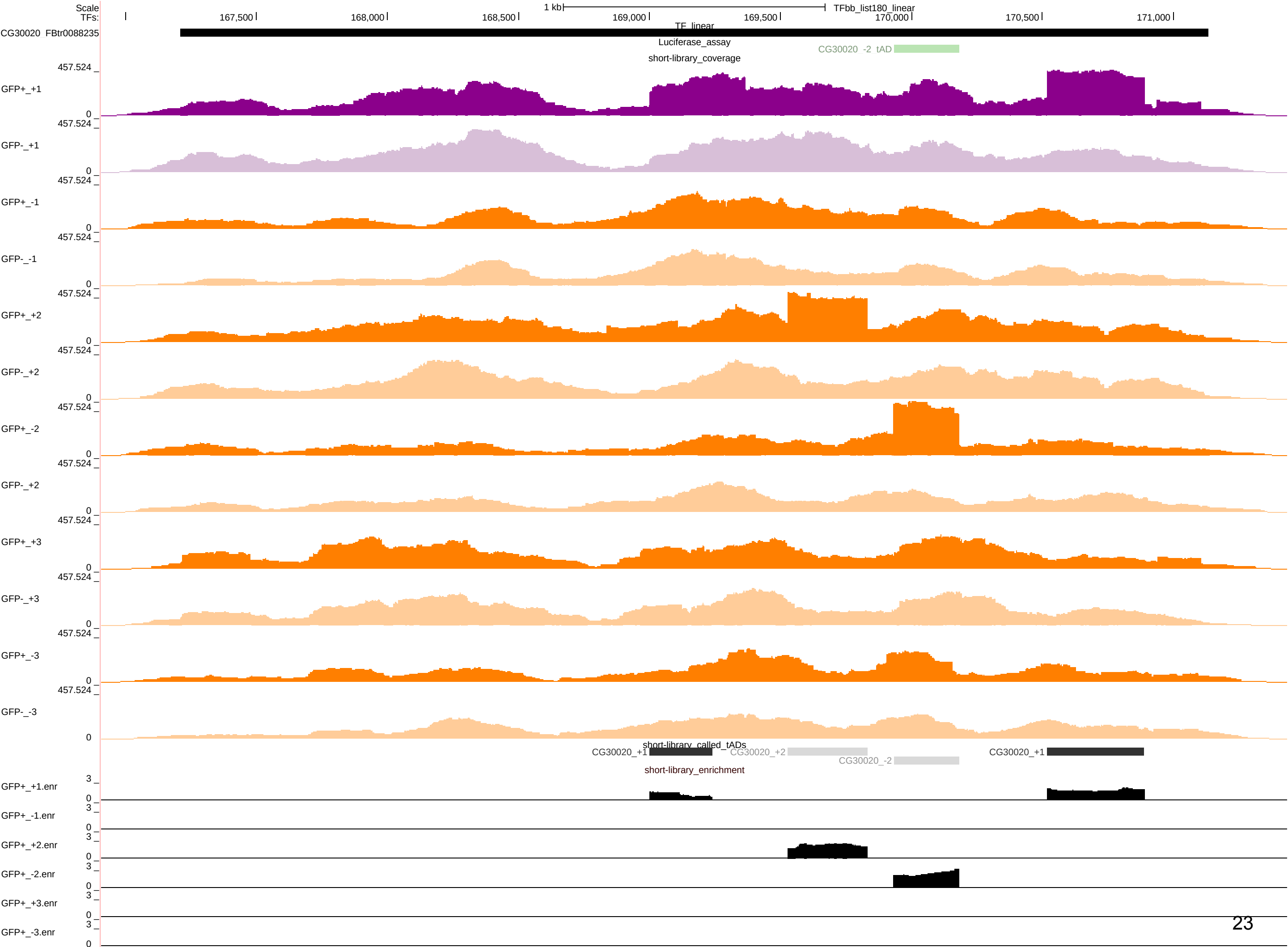
Scale
TFs:
1 kb
TFbb_list180_linear
167,500
168,000
168,500
169,000
169,500
170,000
170,500
171,000
TF_linear
CG30020 FBtr0088235
Luciferase_assay
CG30020 -2 tAD
short-library_coverage
457.524
0
GFP+_+1
GFP-_+1
GFP+_-1
GFP-_-1
GFP+_+2
GFP-_+2
GFP+_-2
GFP-_+2
GFP+_+3
GFP-_+3
GFP+_-3
GFP-_-3
short-library_called_tADs
CG30020_+1
CG30020_+2
CG30020_-2
CG30020_+1
short-library_enrichment
3
GFP+_+1.enr
GFP+_-1.enr
GFP+_+2.enr
GFP+_-2.enr
GFP+_+3.enr
GFP+_-3.enr

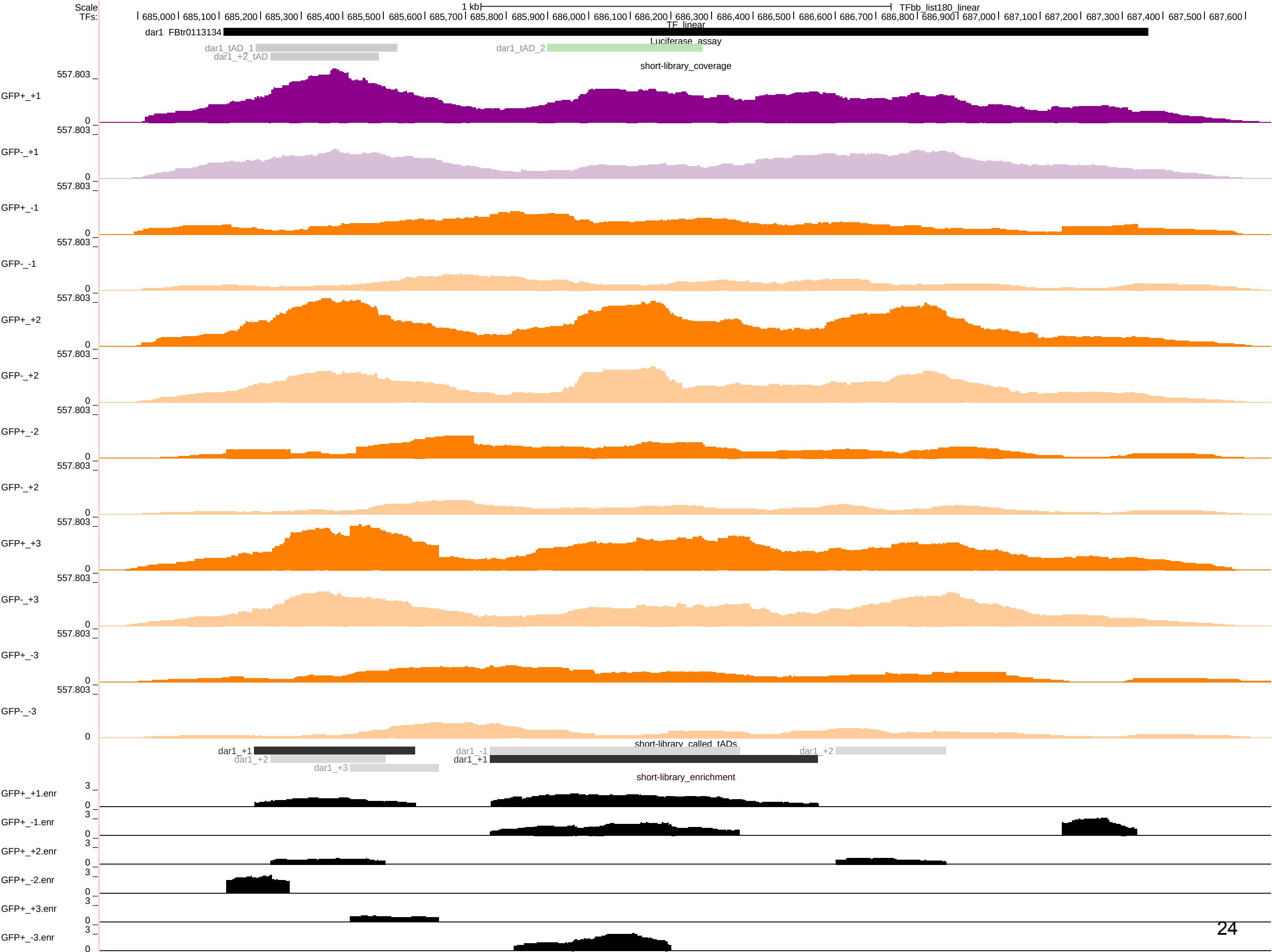
Scale
TFs:
1 kb
TFbb_list180_linear
685,000 685,100 685,200 685,300 685,400 685,500 685,600 685,700 685,800 685,900 686,000 686,100 686,200 686,300 686,400 686,500 686,600 686,700 686,800 686,900 687,000 687,100 687,200 687,300 687,400 687,500 687,600
TF_linear
dar1 FBtr0113134
Luciferase_assay
dar1_tAD_1
dar1_+2_tAD
dar1_tAD_2
short-library_coverage
GFP+_+1
GFP-_+1
GFP+_-1
GFP-_-1
GFP+_+2
GFP-_+2
GFP+_-2
GFP-_+2
GFP+_+3
GFP-_+3
GFP+_-3
GFP-_-3
557.803
0
short-library_called_tADs
dar1_+1
dar1_+2
dar1_+3
dar1_-1
dar1_+1
dar1_+2
short-library_enrichment
GFP+_+1.enr
GFP+_-1.enr
GFP+_+2.enr
GFP+_-2.enr
GFP+_+3.enr
GFP+_-3.enr
3
0

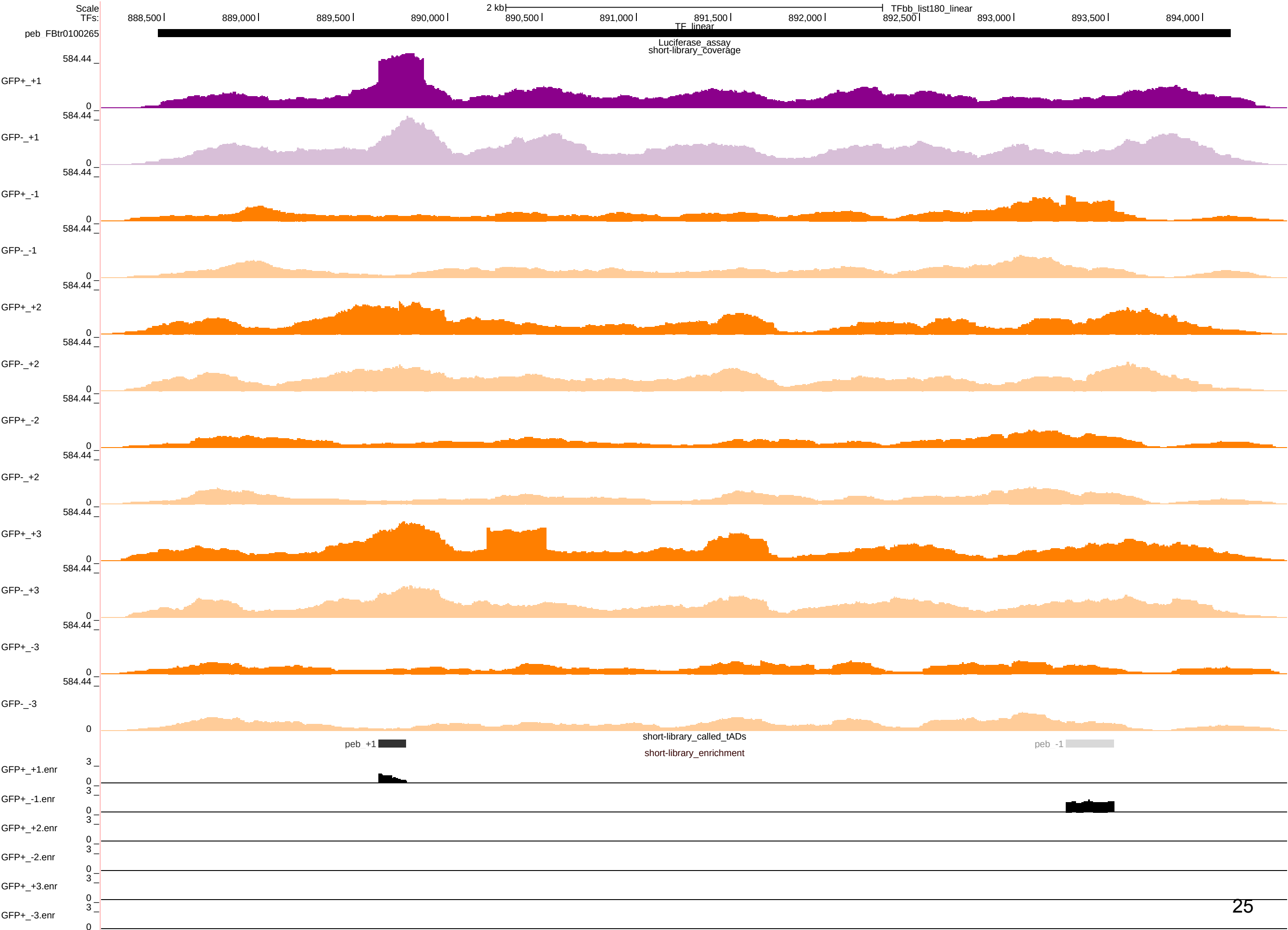

Scale
TFs:
2 kb
TFbb_list180_linear
888,500
889,000
889,500
890,000
890,500
891,000
891,500
892,000
892,500
893,000
893,500
894,000
TF_linear
peb_FBtr0100265
Luciferase_assay
short-library_coverage
584.44
0
GFP+_+1
GFP-_+1
GFP+_-1
GFP-_-1
GFP+_+2
GFP-_+2
GFP+_-2
GFP-_+2
GFP+_+3
GFP-_+3
GFP+_-3
GFP-_-3
short-library_called_tADs
peb_+1
peb_-1
short-library_enrichment
3
GFP+_+1.enr
GFP+_-1.enr
GFP+_+2.enr
GFP+_-2.enr
GFP+_+3.enr
GFP+_-3.enr

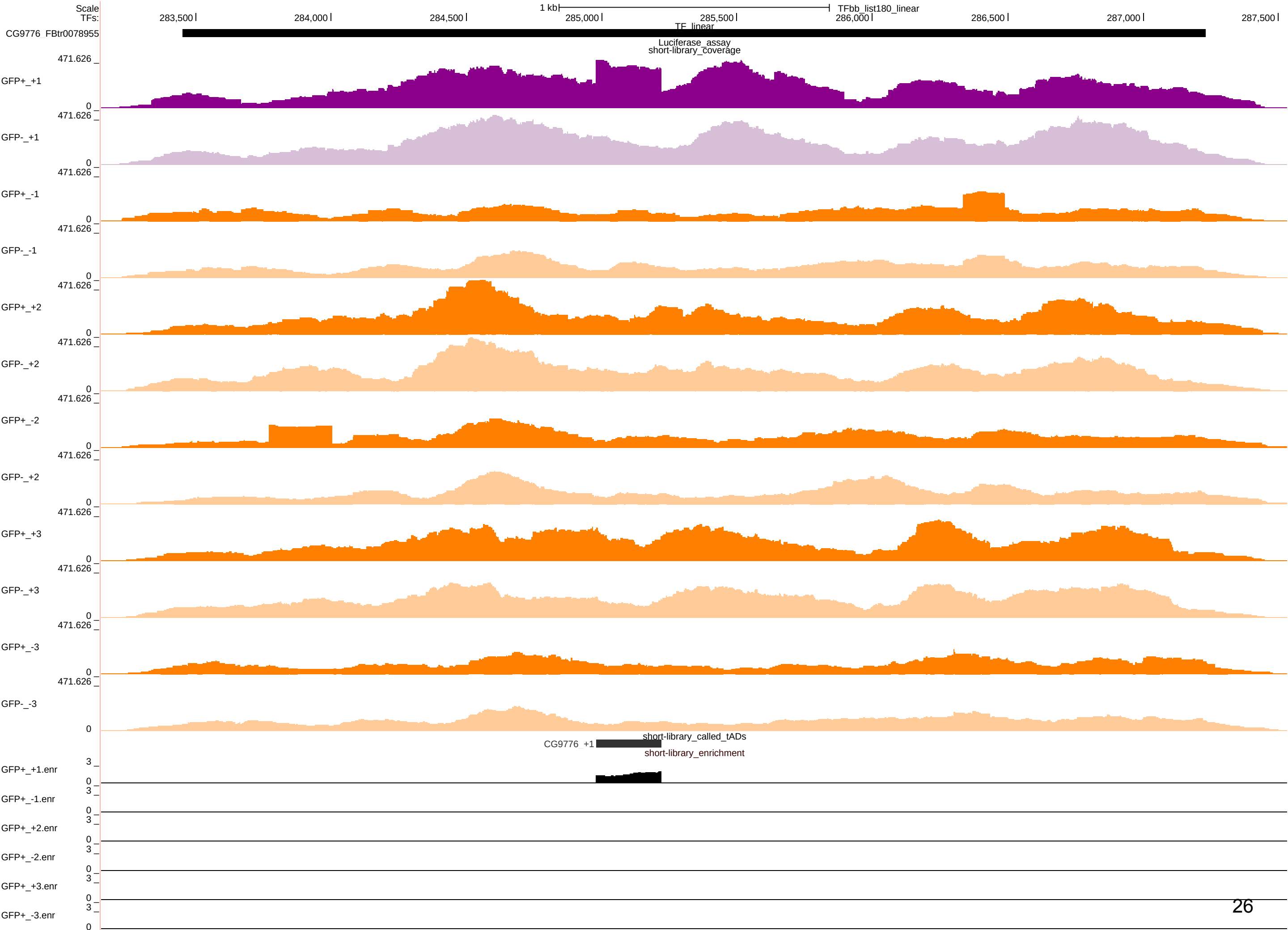
Scale
1 kb
TFs:
TFbb_list180_linear
283,500
284,000
284,500
285,000
285,500
286,000
286,500
287,000
287,500
TF_linear
CG9776 FBtr0078955
Luciferase_assay
short-library_coverage
GFP+_+1
GFP-_+1
GFP+_-1
GFP-_-1
GFP+_+2
GFP-_+2
GFP+_-2
GFP-_+2
GFP+_+3
GFP-_+3
GFP+_-3
GFP-_-3
471.626
0
short-library_called_tADs
CG9776_+1
short-library_enrichment
GFP+_+1.enr
GFP+_-1.enr
GFP+_+2.enr
GFP+_-2.enr
GFP+_+3.enr
GFP+_-3.enr
3
0

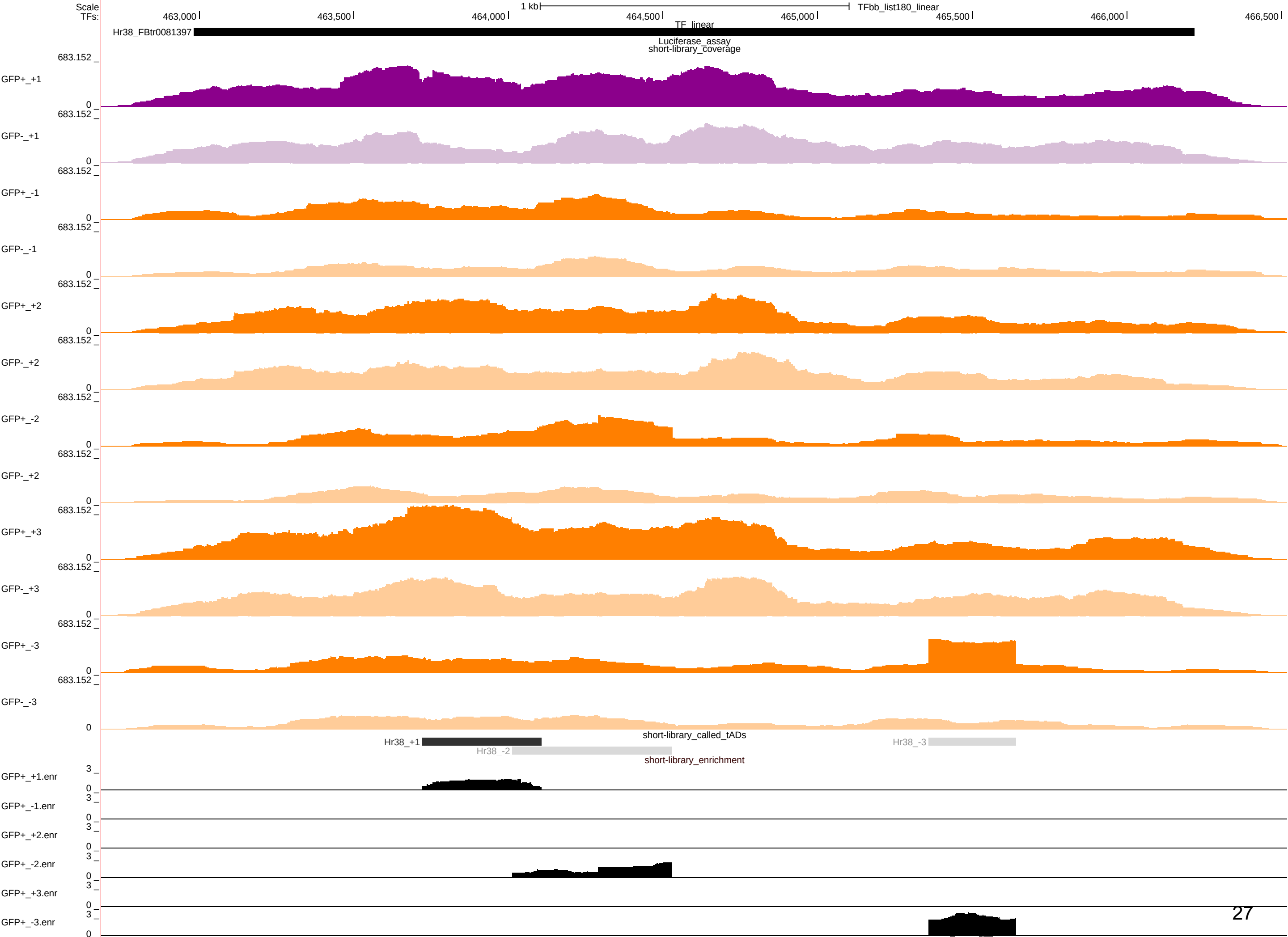
Scale
TFs:
1 kb
TFbb_list180_linear
463,000
463,500
464,000
464,500
465,000
465,500
466,000
466,500
Hr38 FBtr0081397
TF_linear
Luciferase_assay
short-library_coverage
683.152
0
GFP+_+1
GFP-_+1
GFP+_-1
GFP-_-1
GFP+_+2
GFP-_+2
GFP+_-2
GFP-_+2
GFP+_+3
GFP-_+3
GFP+_-3
GFP-_-3
short-library_called_tADs
Hr38_+1
Hr38_-2
Hr38_-3
short-library_enrichment
3
0
GFP+_+1.enr
GFP+_-1.enr
GFP+_+2.enr
GFP+_-2.enr
GFP+_+3.enr
GFP+_-3.enr

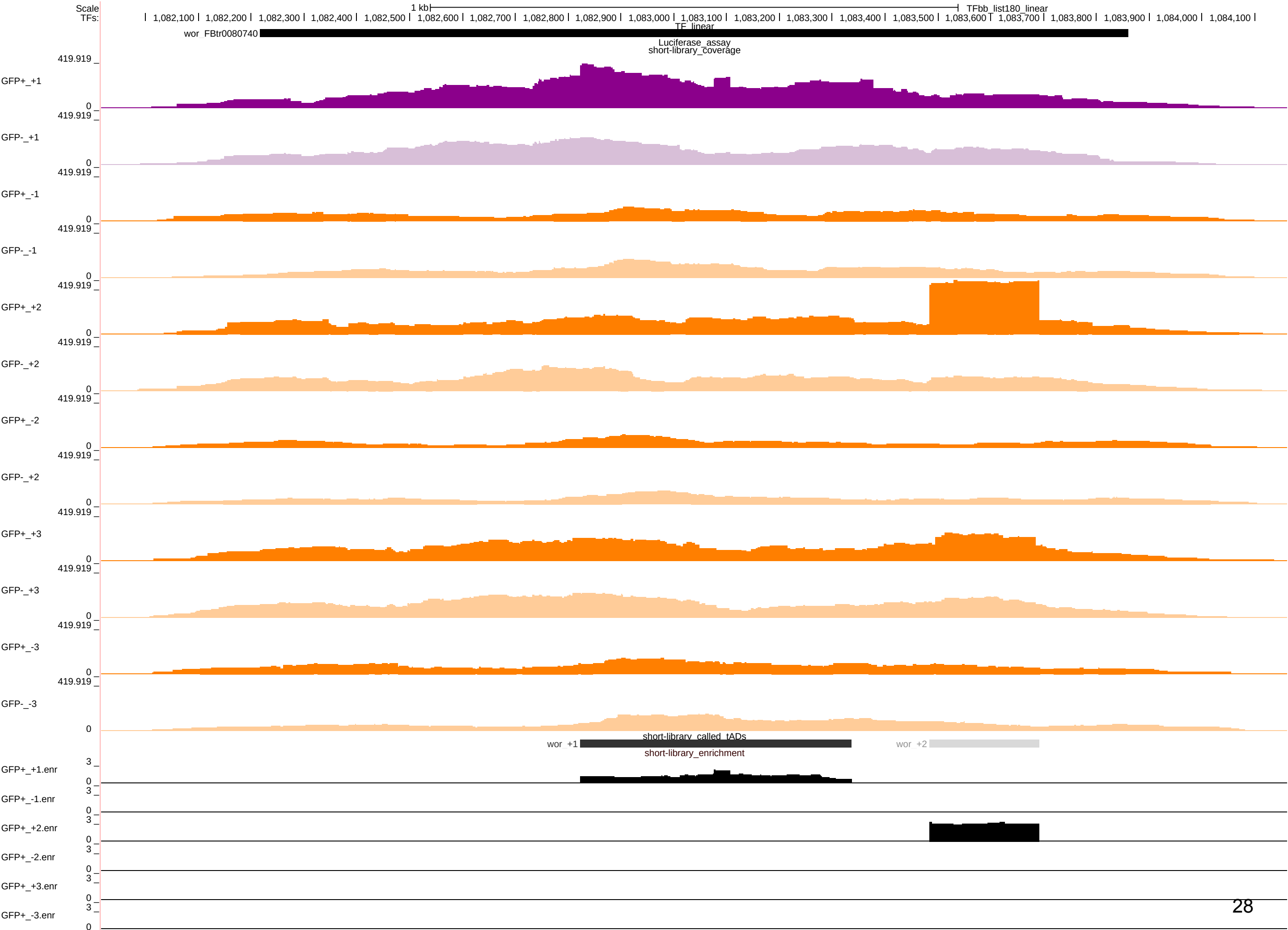

Scale
1 kb
TFbb_list180_linear
TFs:
1,082,100
1,082,200
1,082,300
1,082,400
1,082,500
1,082,600
1,082,700
1,082,800
1,082,900
1,083,000
1,083,100
1,083,200
1,083,300
1,083,400
1,083,500
1,083,600
1,083,700
1,083,800
1,083,900
1,084,000
1,084,100
TF_linear
wor FBtr0080740
Luciferase_assay
short-library_coverage
419.919
GFP+_+1
GFP-_+1
GFP+_-1
GFP-_-1
GFP+_+2
GFP-_+2
GFP+_-2
GFP-_+2
GFP+_+3
GFP-_+3
GFP+_-3
GFP-_-3
0
short-library_called_tADs
wor_+1
wor_+2
short-library_enrichment
3
GFP+_+1.enr
GFP+_-1.enr
GFP+_+2.enr
GFP+_-2.enr
GFP+_+3.enr
GFP+_-3.enr

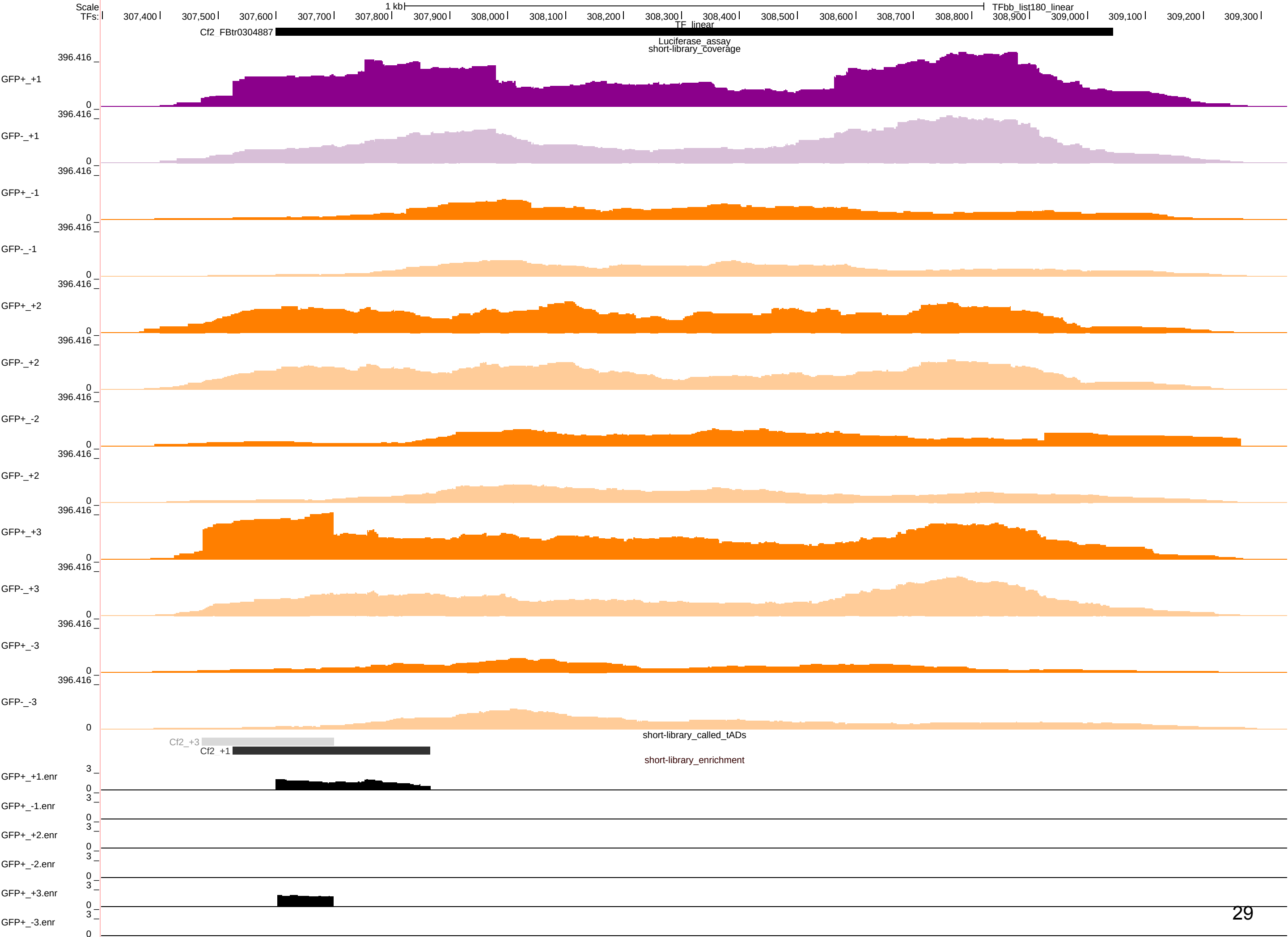

Scale
TFs:
1 kb
TFbb_list180_linear
307,400
307,500
307,600
307,700
307,800
307,900
308,000
308,100
308,200
308,300
308,400
308,500
308,600
308,700
308,800
308,900
309,000
309,100
309,200
309,300
TF_linear
Cf2_FBtr0304887
Luciferase_assay
short-library_coverage
GFP+_+1
GFP-_+1
GFP+_-1
GFP-_-1
GFP+_+2
GFP-_+2
GFP+_-2
GFP-_+2
GFP+_+3
GFP-_+3
GFP+_-3
GFP-_-3
396.416
0
short-library_called_tADs
Cf2_+3
Cf2_+1
short-library_enrichment
GFP+_+1.enr
GFP+_-1.enr
GFP+_+2.enr
GFP+_-2.enr
GFP+_+3.enr
GFP+_-3.enr
3
0

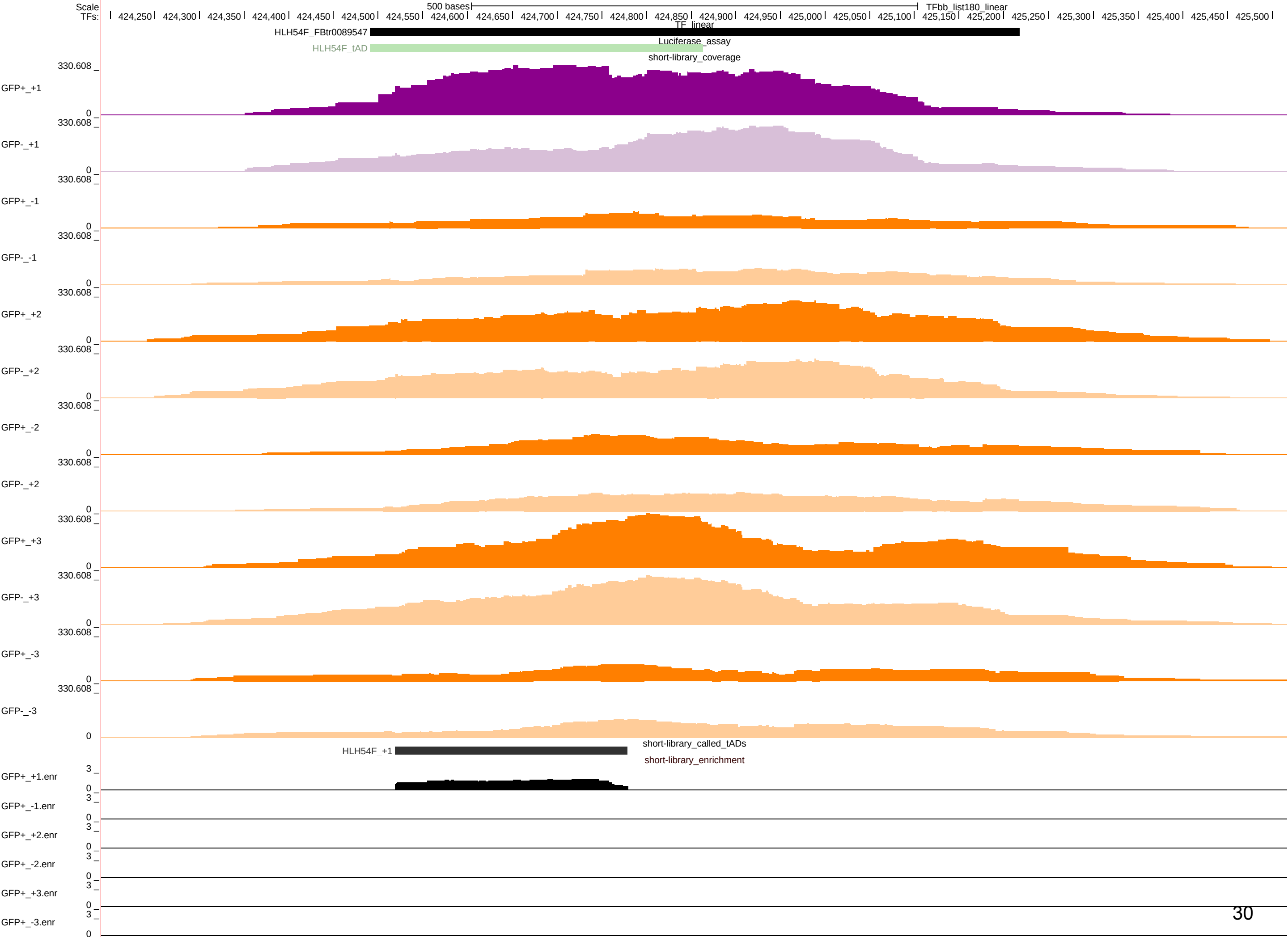

Scale
500 bases
TFs:
424,250 424,300 424,350 424,400 424,450 424,500 424,550 424,600 424,650 424,700 424,750 424,800 424,850 424,900 424,950 425,000 425,050 425,100 425,150 425,200 425,250 425,300 425,350 425,400 425,450 425,500
TFbb_list180_linear
TF_linear
HLH54F_FBtr0089547
Luciferase_assay
HLH54F_tAD
short-library_coverage
GFP+_+1
GFP-_+1
GFP+_-1
GFP-_-1
GFP+_+2
GFP-_+2
GFP+_-2
GFP-_+2
GFP+_+3
GFP-_+3
GFP+_-3
GFP-_-3
330.608
0
short-library_called_tADs
HLH54F_+1
short-library_enrichment
GFP+_+1.enr
GFP+_-1.enr
GFP+_+2.enr
GFP+_-2.enr
GFP+_+3.enr
GFP+_-3.enr
3
0

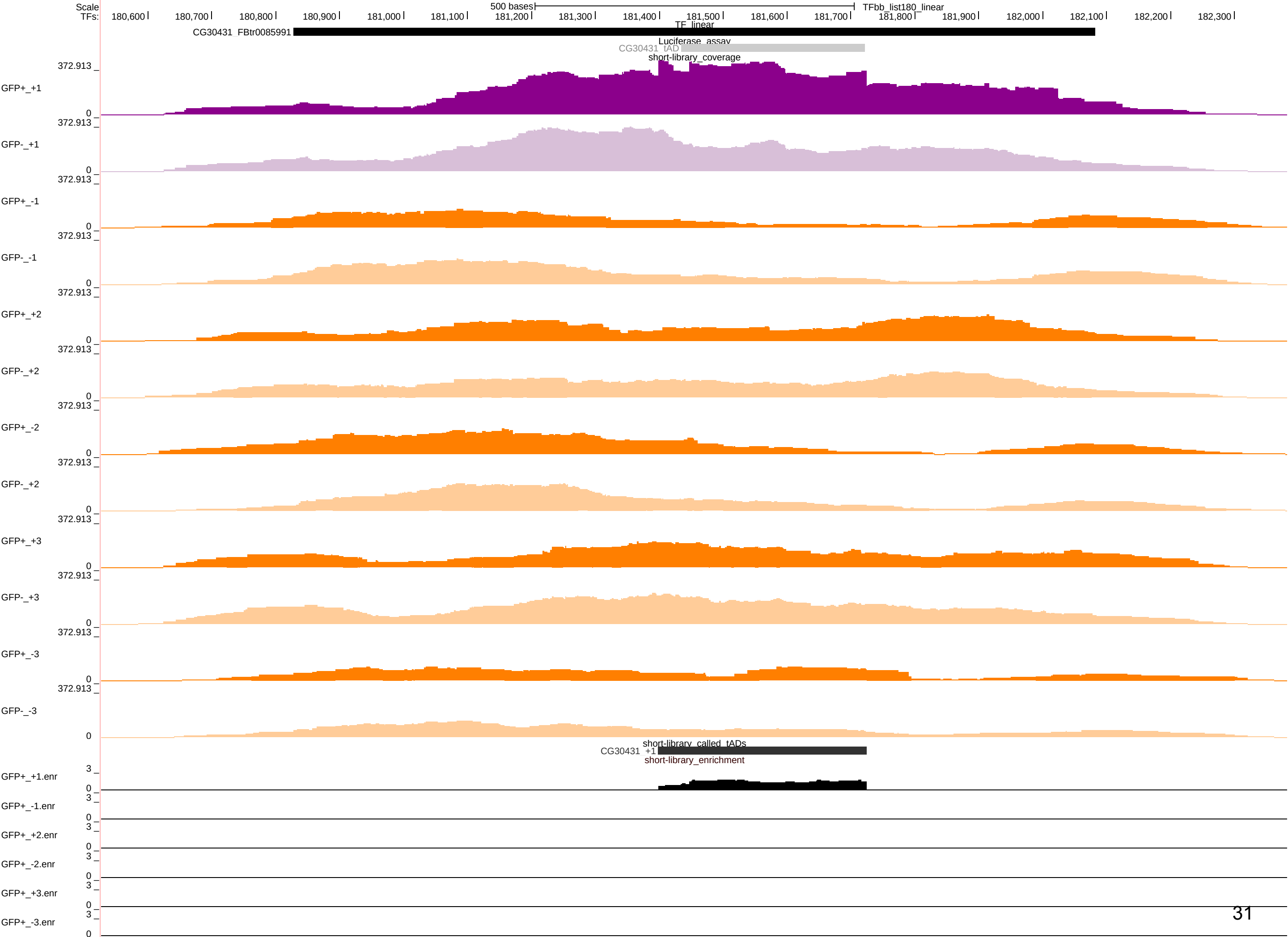

Scale
TFs:
500 bases
180,600
180,700
180,800
180,900
181,000
181,100
181,200
181,300
181,400
181,500
181,600
181,700
181,800
181,900
182,000
182,100
182,200
182,300
TFbb_list180_linear
TF_linear
CG30431_FBtr0085991
Luciferase_assay
CG30431_tAD
short-library_coverage
GFP+_+1
GFP-_+1
GFP+_-1
GFP-_-1
GFP+_+2
GFP-_+2
GFP+_-2
GFP-_+2
GFP+_+3
GFP-_+3
GFP+_-3
GFP-_-3
372.913
0
short-library_called_tADs
CG30431_+1
short-library_enrichment
GFP+_+1.enr
GFP+_-1.enr
GFP+_+2.enr
GFP+_-2.enr
GFP+_+3.enr
GFP+_-3.enr
3
0

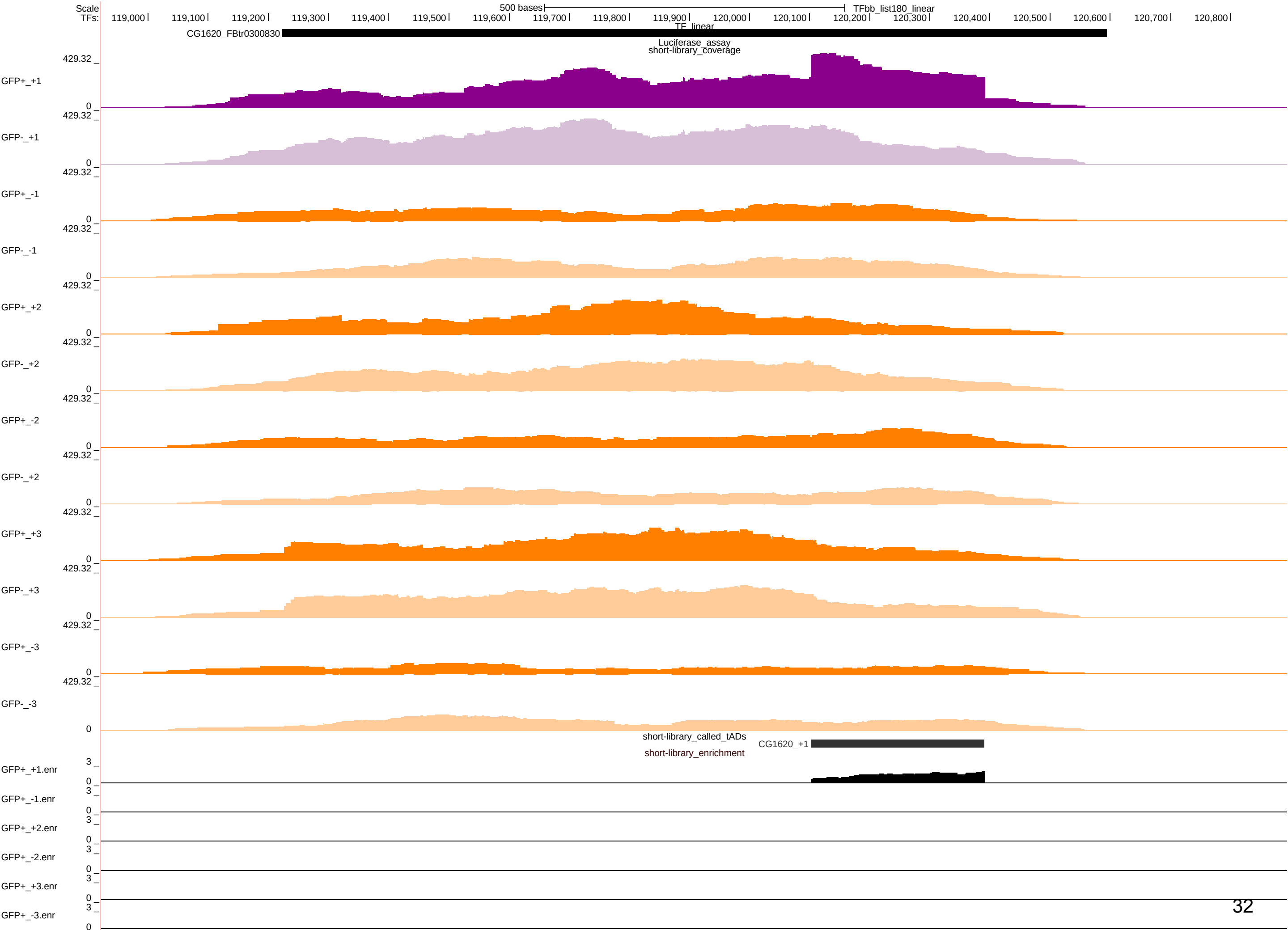

Scale
TFs:
500 bases
TFbb_list180_linear
119,000
119,100
119,200
119,300
119,400
119,500
119,600
119,700
119,800
119,900
120,000
120,100
120,200
120,300
120,400
120,500
120,600
120,700
120,800
TF_linear
CG1620 FBtr0300830
Luciferase_assay
short-library_coverage
429.32
0
GFP+_+1
GFP-_+1
GFP+_-1
GFP-_-1
GFP+_+2
GFP-_+2
GFP+_-2
GFP-_+2
GFP+_+3
GFP-_+3
GFP+_-3
GFP-_-3
short-library_called_tADs
CG1620_+1
short-library_enrichment
3
GFP+_+1.enr
GFP+_-1.enr
GFP+_+2.enr
GFP+_-2.enr
GFP+_+3.enr
GFP+_-3.enr

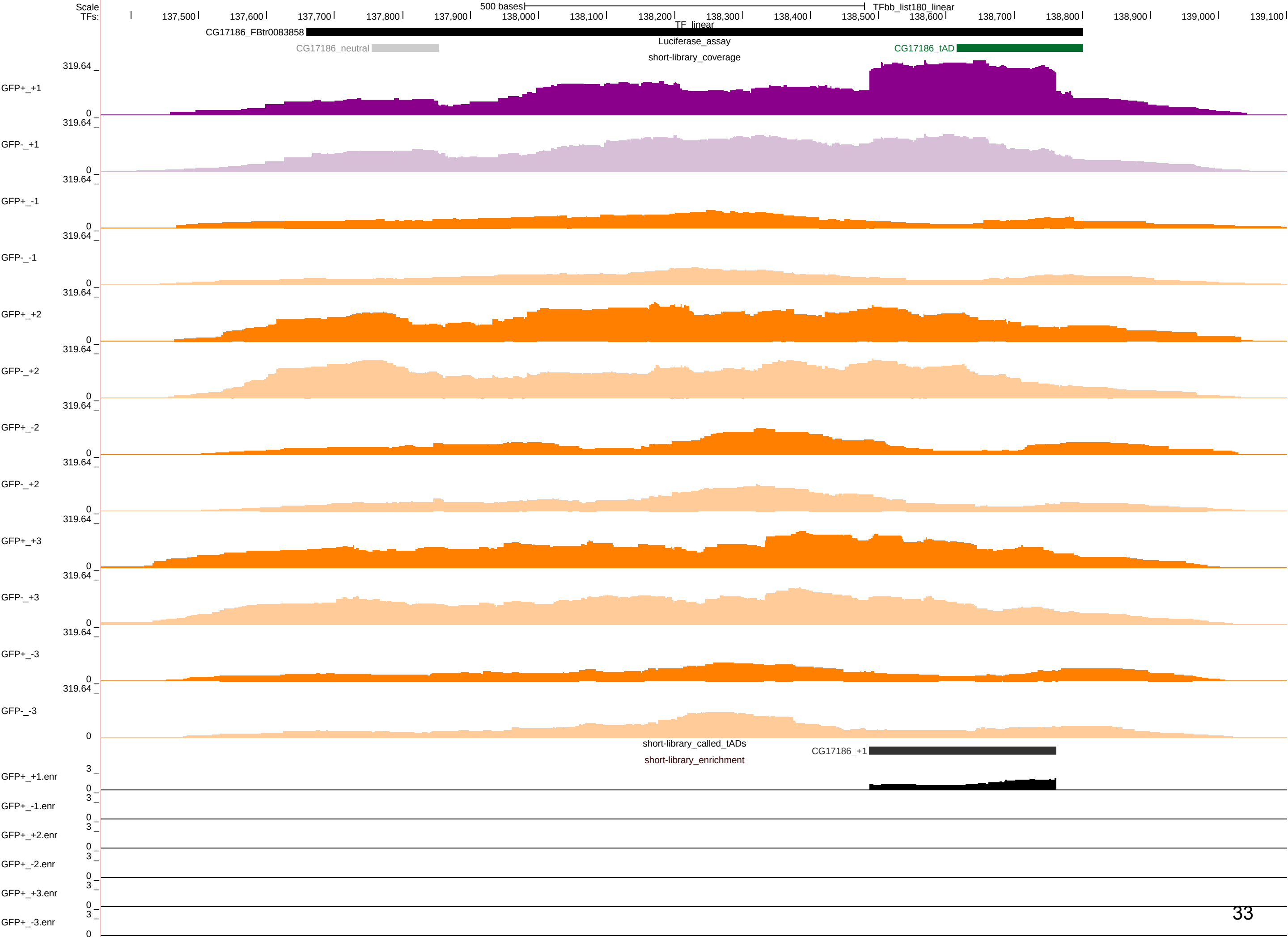
Scale
TFs:
500 bases
TFbb_list180_linear
137,500
137,600
137,700
137,800
137,900
138,000
138,100
138,200
138,300
138,400
138,500
138,600
138,700
138,800
138,900
139,000
139,100
TF_linear
CG17186 FBtr0083858
Luciferase_assay
CG17186 neutral
CG17186 tAD
short-library_coverage
319.64
0
GFP+_+1
GFP-_+1
GFP+_-1
GFP-_-1
GFP+_+2
GFP-_+2
GFP+_-2
GFP-_+2
GFP+_+3
GFP-_+3
GFP+_-3
GFP-_-3
short-library_called_tADs
CG17186_+1
short-library_enrichment
3
GFP+_+1.enr
GFP+_-1.enr
GFP+_+2.enr
GFP+_-2.enr
GFP+_+3.enr
GFP+_-3.enr

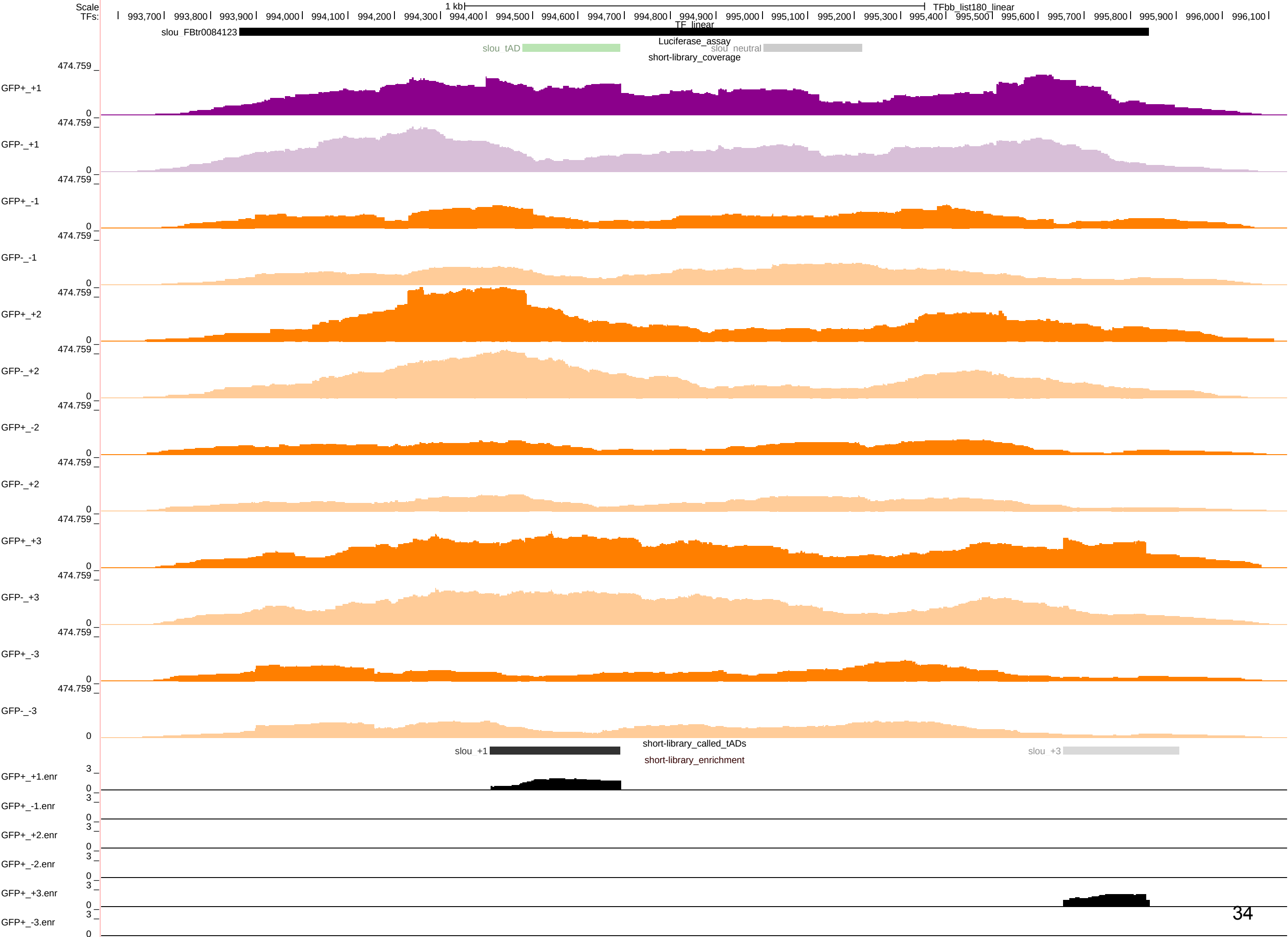

Scale
TFs:
1 kb
TFbb_list180_linear
993,700
993,800
993,900
994,000
994,100
994,200
994,300
994,400
994,500
994,600
994,700
994,800
994,900
995,000
995,100
995,200
995,300
995,400
995,500
995,600
995,700
995,800
995,900
996,000
996,100
TF_linear
slou FBtr0084123
Luciferase_assay
slou tAD
slou neutral
short-library_coverage
474.759
0
GFP+_+1
GFP-_+1
GFP+_-1
GFP-_-1
GFP+_+2
GFP-_+2
GFP+_-2
GFP-_+2
GFP+_+3
GFP-_+3
GFP+_-3
GFP-_-3
short-library_called_tADs
slou +1
slou +3
short-library_enrichment
3
GFP+_+1.enr
GFP+_-1.enr
GFP+_+2.enr
GFP+_-2.enr
GFP+_+3.enr
GFP+_-3.enr

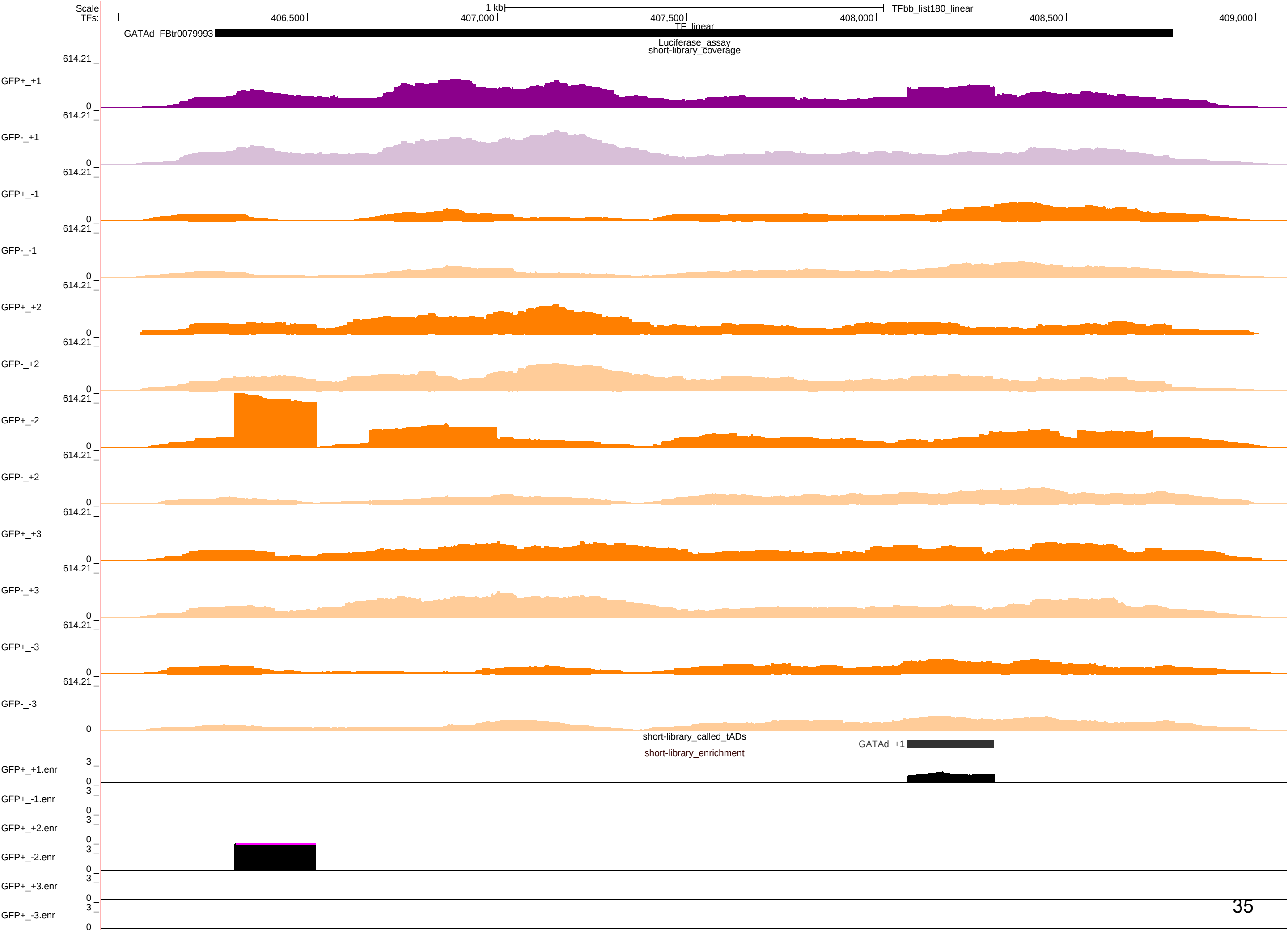
Scale
TFs:
1 kb
TFbb_list180_linear
406,500
407,000
407,500
408,000
408,500
409,000
TF_linear
GATAd FBtr0079993
Luciferase_assay
short-library_coverage
614.21
0
GFP+_+1
GFP-_+1
GFP+_-1
GFP-_-1
GFP+_+2
GFP-_+2
GFP+_-2
GFP-_+2
GFP+_+3
GFP-_+3
GFP+_-3
GFP-_-3
short-library_called_tADs
GATAd_+1
short-library_enrichment
3
GFP+_+1.enr
GFP+_-1.enr
GFP+_+2.enr
GFP+_-2.enr
GFP+_+3.enr
GFP+_-3.enr

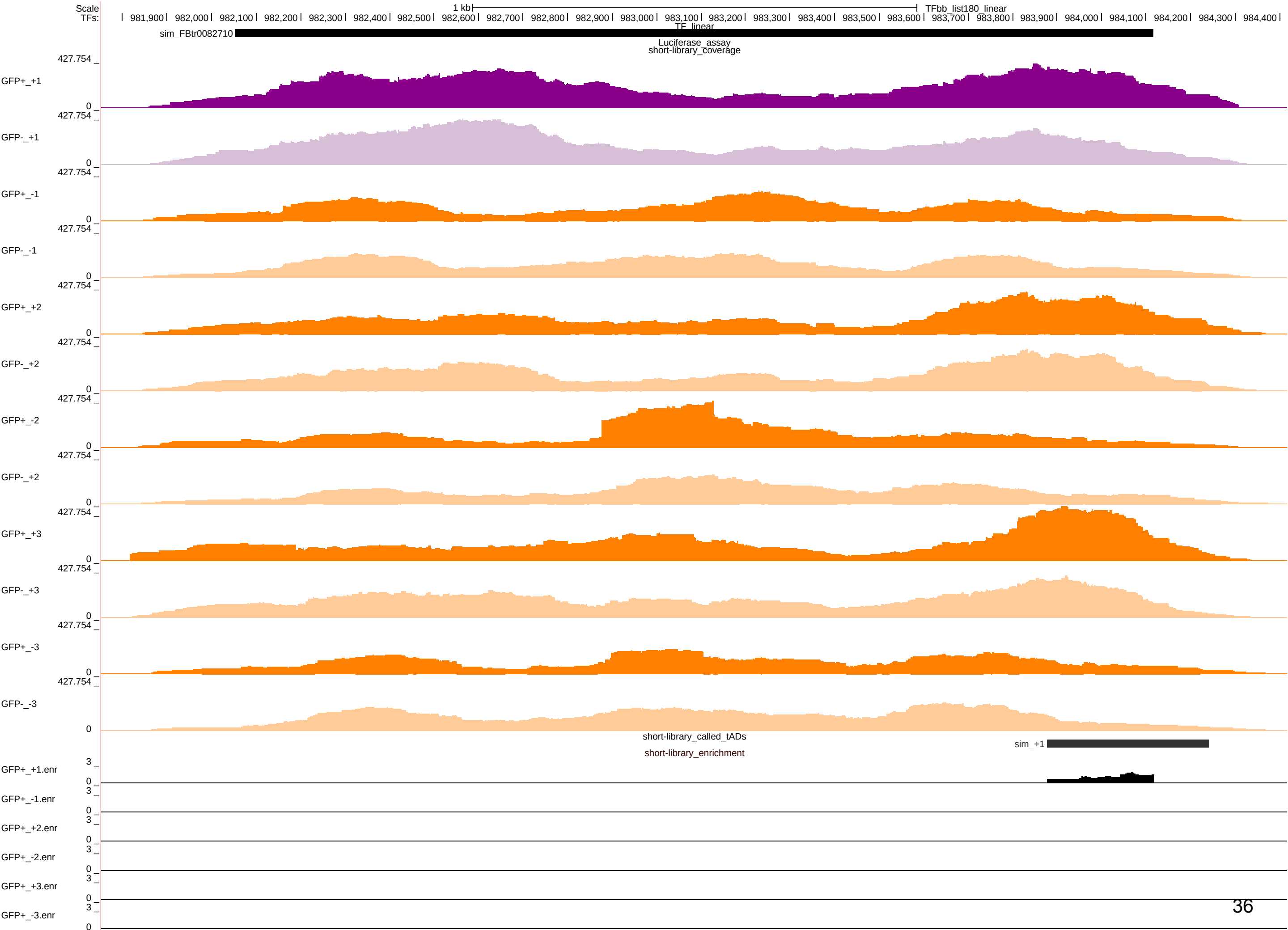
Scale
TFs:
1 kb
TFbb_list180_linear
981,900 982,000 982,100 982,200 982,300 982,400 982,500 982,600 982,700 982,800 982,900 983,000 983,100 983,200 983,300 983,400 983,500 983,600 983,700 983,800 983,900 984,000 984,100 984,200 984,300 984,400
TF_linear
sim_FBtr0082710
Luciferase_assay
short-library_coverage
427.754
0
GFP+_+1
GFP-_+1
GFP+_-1
GFP-_-1
GFP+_+2
GFP-_+2
GFP+_-2
GFP-_+2
GFP+_+3
GFP-_+3
GFP+_-3
GFP-_-3
short-library_called_tADs
sim_+1
short-library_enrichment
3
GFP+_+1.enr
GFP+_-1.enr
GFP+_+2.enr
GFP+_-2.enr
GFP+_+3.enr
GFP+_-3.enr

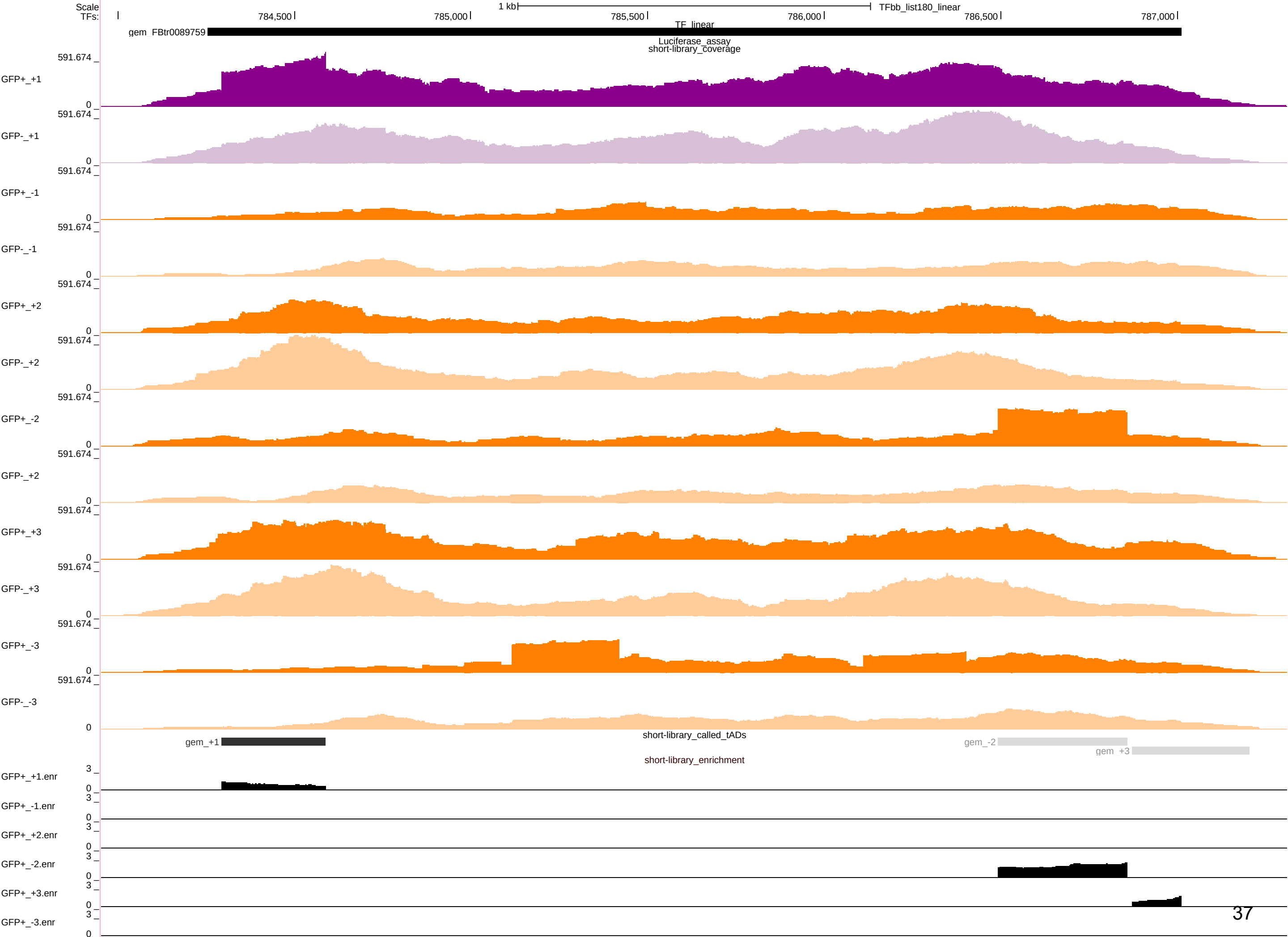

Scale
TFs:
1 kb
TFbb_list180_linear
784,500
785,000
785,500
786,000
786,500
787,000
TF_linear
gem_FBtr0089759
Luciferase_assay
short-library_coverage
591.674
0
GFP+_+1
GFP-_+1
GFP+_-1
GFP-_-1
GFP+_+2
GFP-_+2
GFP+_-2
GFP-_+2
GFP+_+3
GFP-_+3
GFP+_-3
GFP-_-3
short-library_called_tADs
gem_+1
gem_-2
gem_+3
short-library_enrichment
3
GFP+_+1.enr
GFP+_-1.enr
GFP+_+2.enr
GFP+_-2.enr
GFP+_+3.enr
GFP+_-3.enr

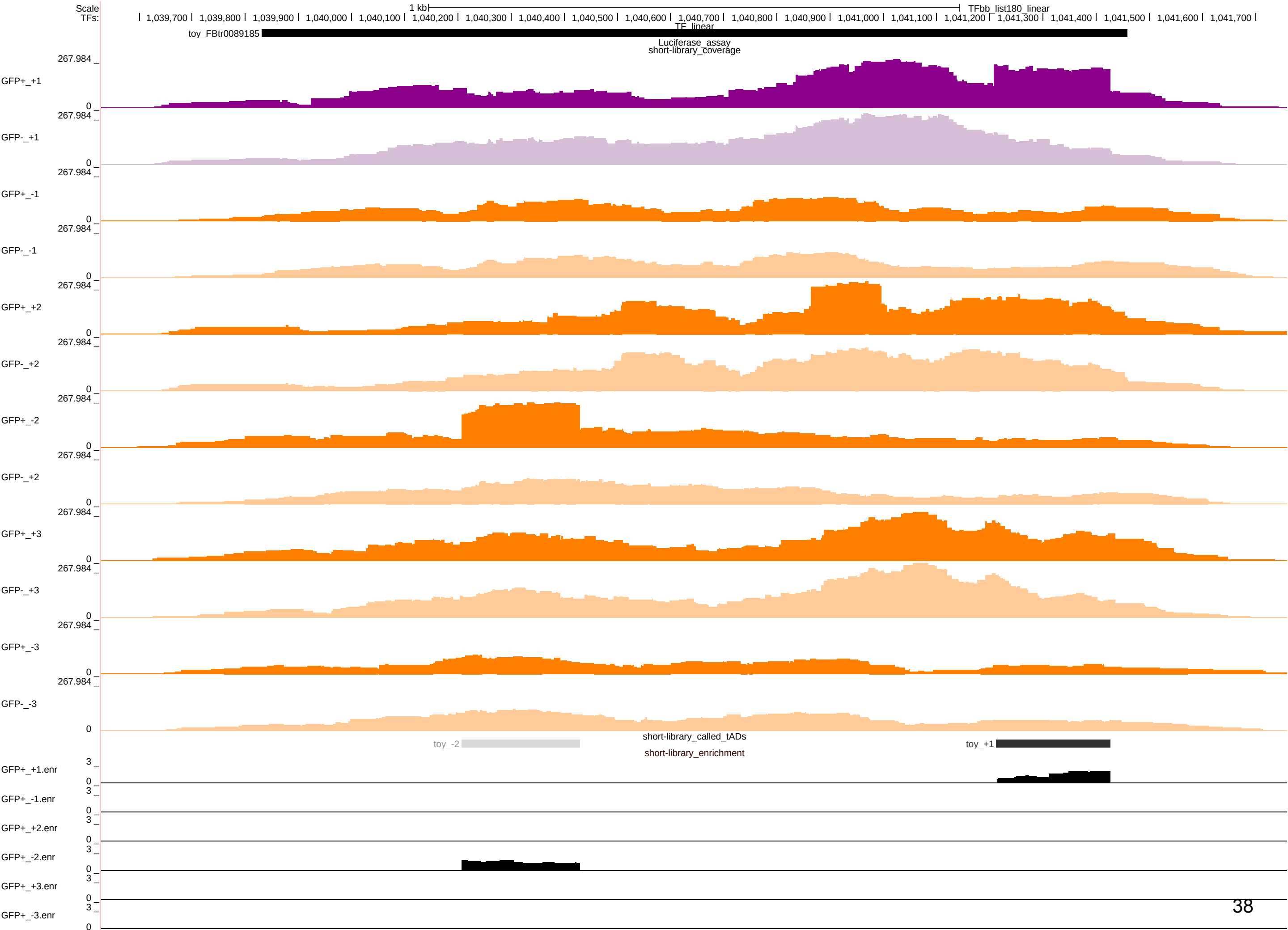
Scale
TFs:
1 kb
TFbb_list180_linear
1,039,700 1,039,800 1,039,900 1,040,000 1,040,100 1,040,200 1,040,300 1,040,400 1,040,500 1,040,600 1,040,700 1,040,800 1,040,900 1,041,000 1,041,100 1,041,200 1,041,300 1,041,400 1,041,500 1,041,600 1,041,700
TF_linear
toy_FBtr0089185
Luciferase_assay
short-library_coverage
GFP+_+1
GFP-_+1
GFP+_-1
GFP-_-1
GFP+_+2
GFP-_+2
GFP+_-2
GFP-_+2
GFP+_+3
GFP-_+3
GFP+_-3
GFP-_-3
267.984
0
short-library_called_tADs
toy_-2
toy_+1
short-library_enrichment
GFP+_+1.enr
GFP+_-1.enr
GFP+_+2.enr
GFP+_-2.enr
GFP+_+3.enr
GFP+_-3.enr
3
0

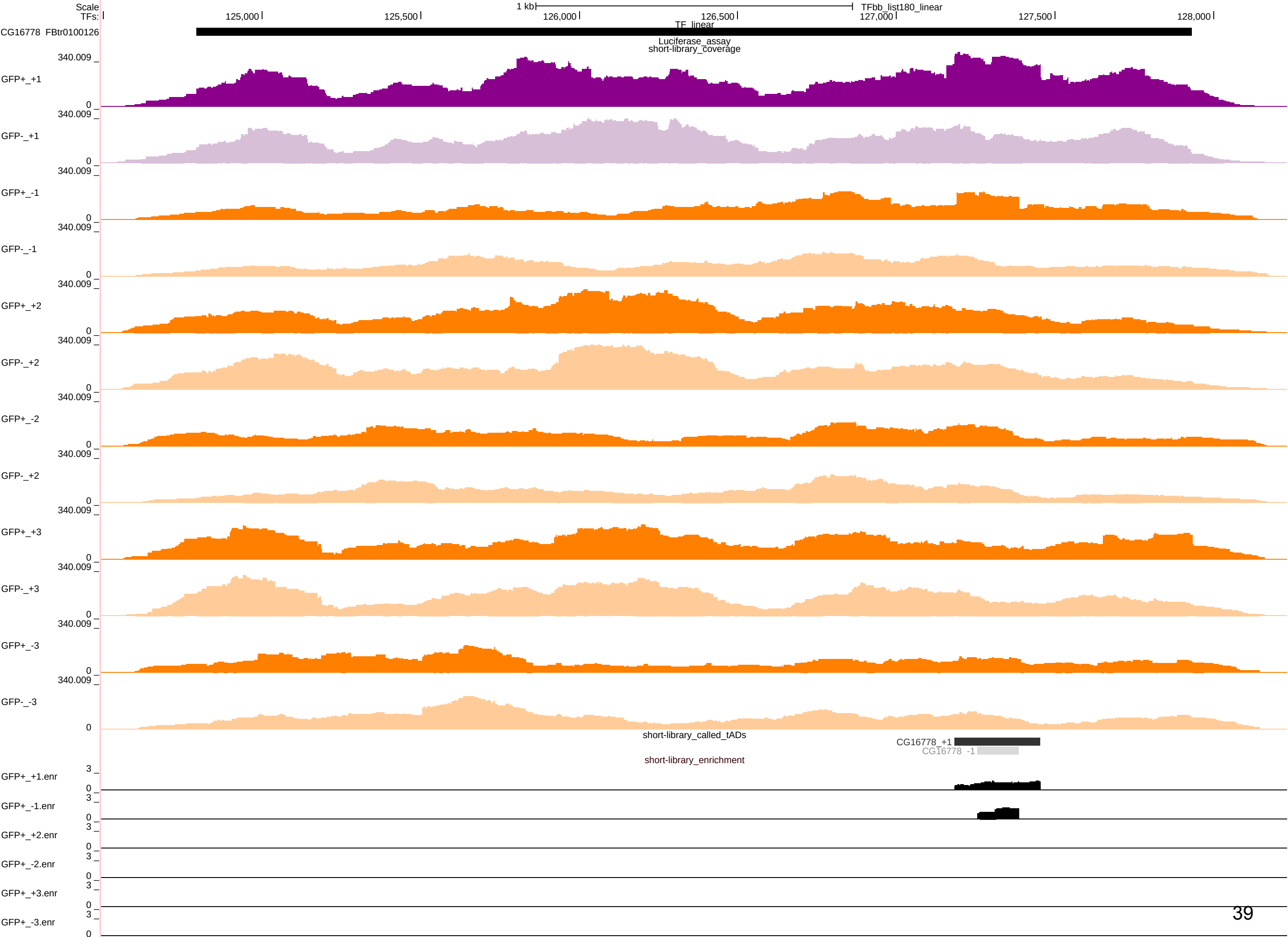

Scale
1 kb
TFs:
125,000
125,500
126,000
126,500
127,000
127,500
128,000
TFbb_list180_linear
TF_linear
CG16778 FBtr0100126
Luciferase_assay
short-library_coverage
GFP+_+1
GFP-_+1
GFP+_-1
GFP-_-1
GFP+_+2
GFP-_+2
GFP+_-2
GFP-_+2
GFP+_+3
GFP-_+3
GFP+_-3
GFP-_-3
340.009
0
short-library_called_tADs
CG16778_+1
CG16778_-1
short-library_enrichment
GFP+_+1.enr
GFP+_-1.enr
GFP+_+2.enr
GFP+_-2.enr
GFP+_+3.enr
GFP+_-3.enr
3
0

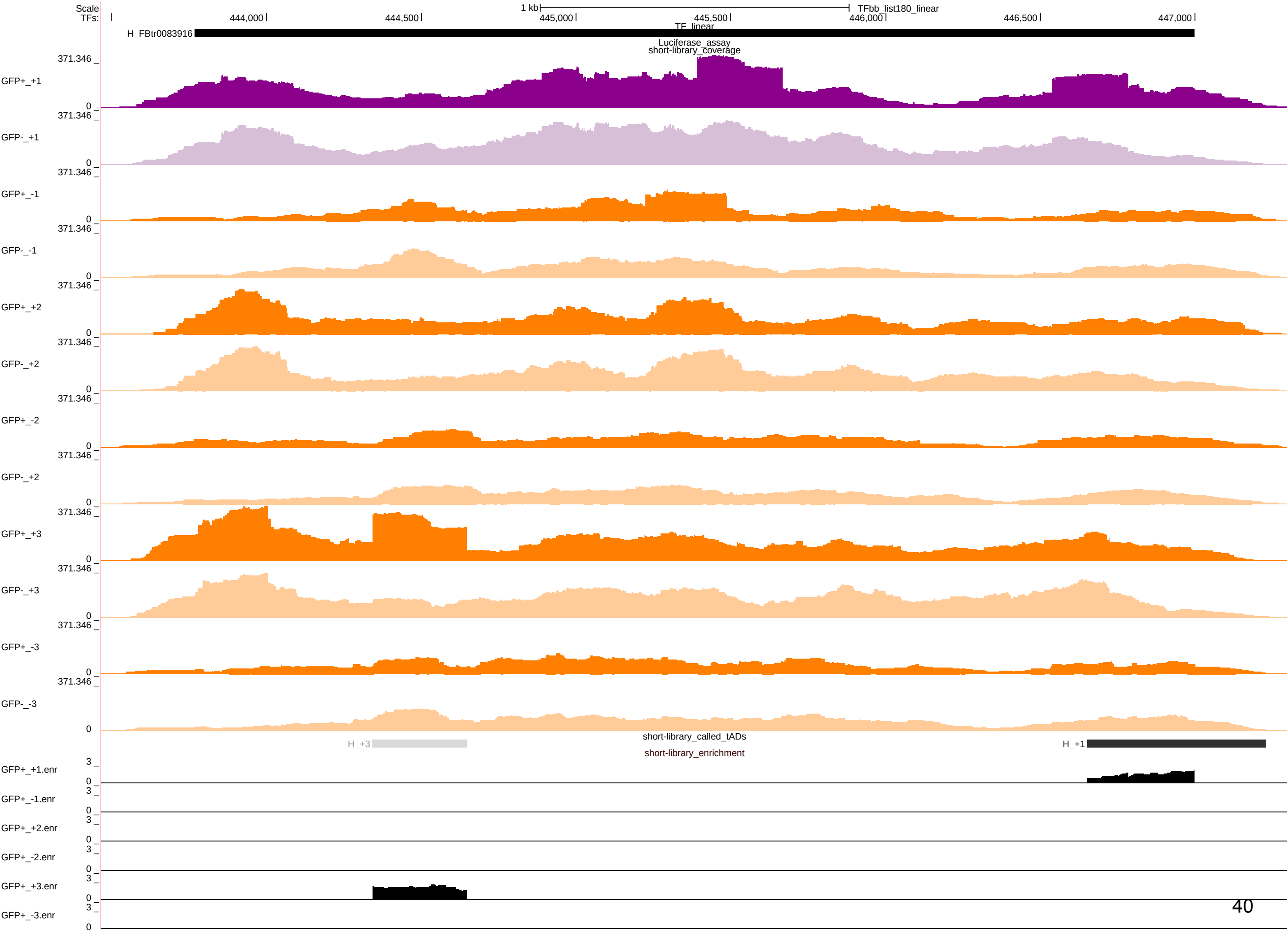

Scale
TFs:
1 kb
TFbb_list180_linear
444,000
444,500
445,000
445,500
446,000
446,500
447,000
H_FBtr0083916
TF_linear
Luciferase_assay
short-library_coverage
371.346
0
GFP+_+1
GFP-_+1
GFP+_-1
GFP-_-1
GFP+_+2
GFP-_+2
GFP+_-2
GFP-_+2
GFP+_+3
GFP-_+3
GFP+_-3
GFP-_-3
short-library_called_tADs
H_+3
H_+1
short-library_enrichment
3
GFP+_+1.enr
GFP+_-1.enr
GFP+_+2.enr
GFP+_-2.enr
GFP+_+3.enr
GFP+_-3.enr

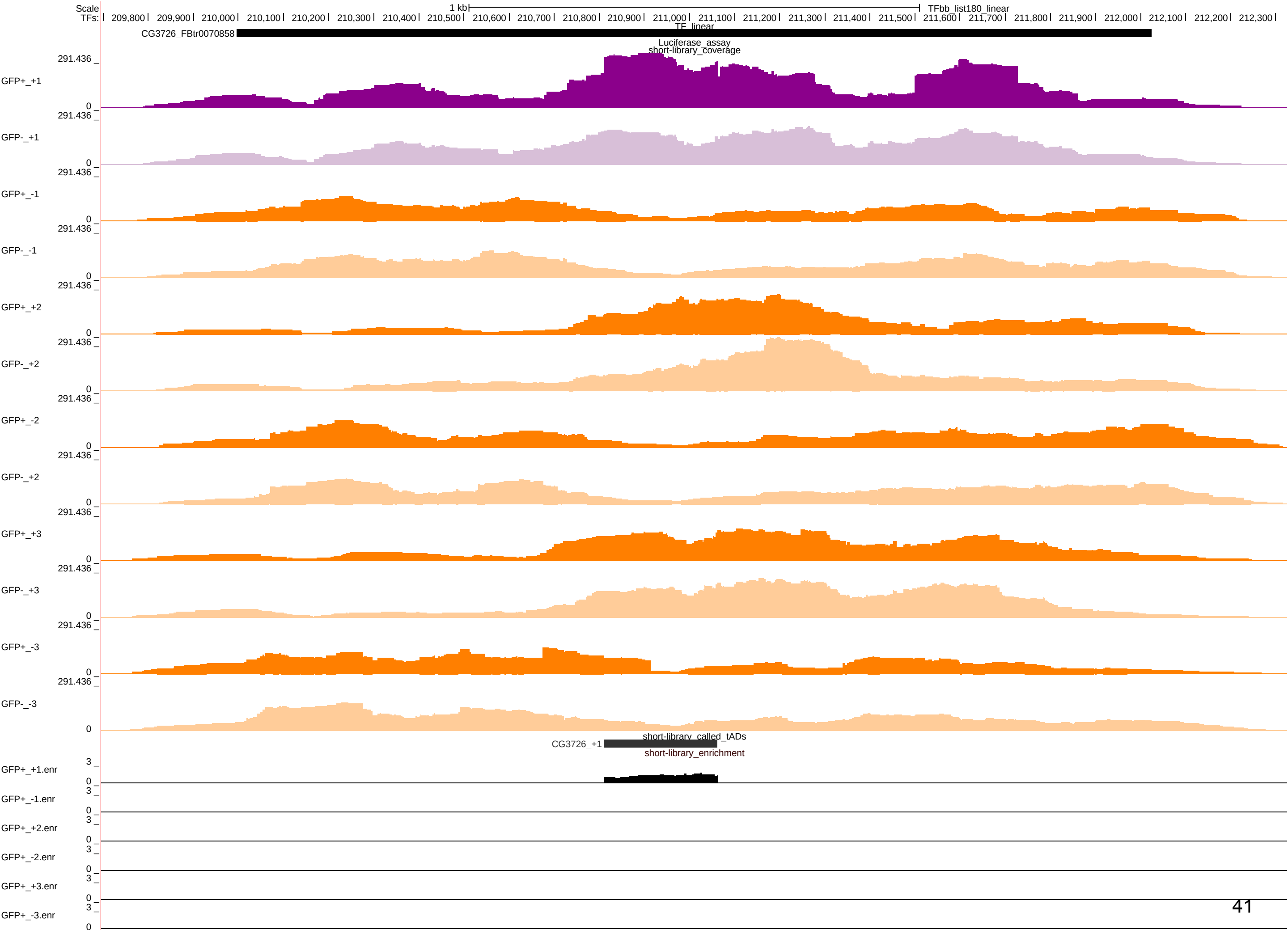
Scale
1 kb
TFbb_list180_linear
TFs:
209,800 209,900 210,000 210,100 210,200 210,300 210,400 210,500 210,600 210,700 210,800 210,900 211,000 211,100 211,200 211,300 211,400 211,500 211,600 211,700 211,800 211,900 212,000 212,100 212,200 212,300
TF_linear
CG3726_FBtr0070858
Luciferase_assay
short-library_coverage
GFP+_+1
GFP-_+1
GFP+_-1
GFP-_-1
GFP+_+2
GFP-_+2
GFP+_-2
GFP-_+2
GFP+_+3
GFP-_+3
GFP+_-3
GFP-_-3
291.436
0
short-library_called_tADs
CG3726_+1
short-library_enrichment
GFP+_+1.enr
GFP+_-1.enr
GFP+_+2.enr
GFP+_-2.enr
GFP+_+3.enr
GFP+_-3.enr
3
0

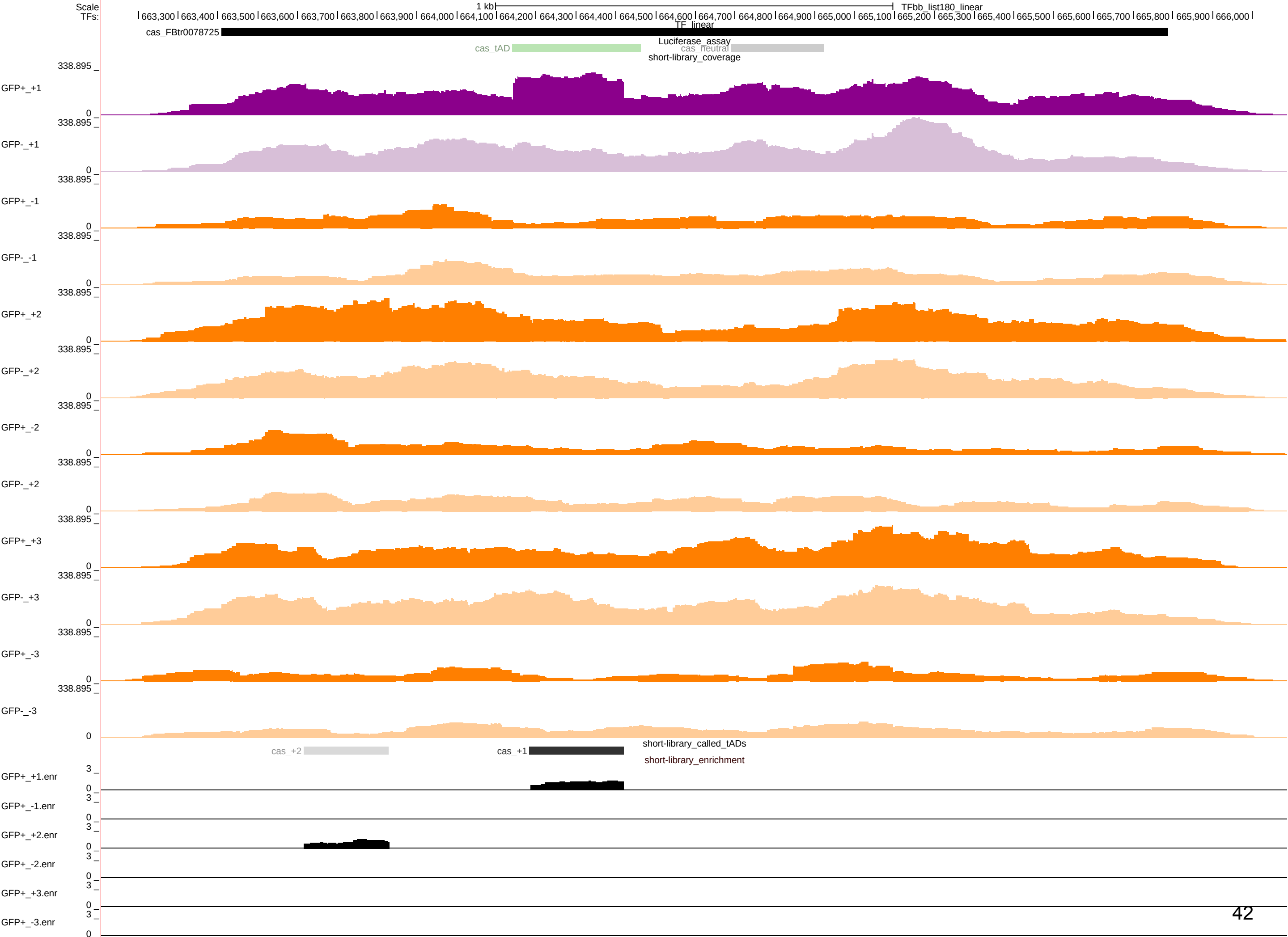
Scale
TFs:
1 kb
TFbb_list180_linear
663,300
663,400
663,500
663,600
663,700
663,800
663,900
664,000
664,100
664,200
664,300
664,400
664,500
664,600
664,700
664,800
664,900
665,000
665,100
665,200
665,300
665,400
665,500
665,600
665,700
665,800
665,900
666,000
TF_linear
cas_FBtr0078725
Luciferase_assay
cas_tAD
cas_neutral
short-library_coverage
GFP+_+1
GFP-_+1
GFP+_-1
GFP-_-1
GFP+_+2
GFP-_+2
GFP+_-2
GFP-_+2
GFP+_+3
GFP-_+3
GFP+_-3
GFP-_-3
338.895
0
short-library_called_tADs
cas_+2
cas_+1
short-library_enrichment
GFP+_+1.enr
GFP+_-1.enr
GFP+_+2.enr
GFP+_-2.enr
GFP+_+3.enr
GFP+_-3.enr
3
0

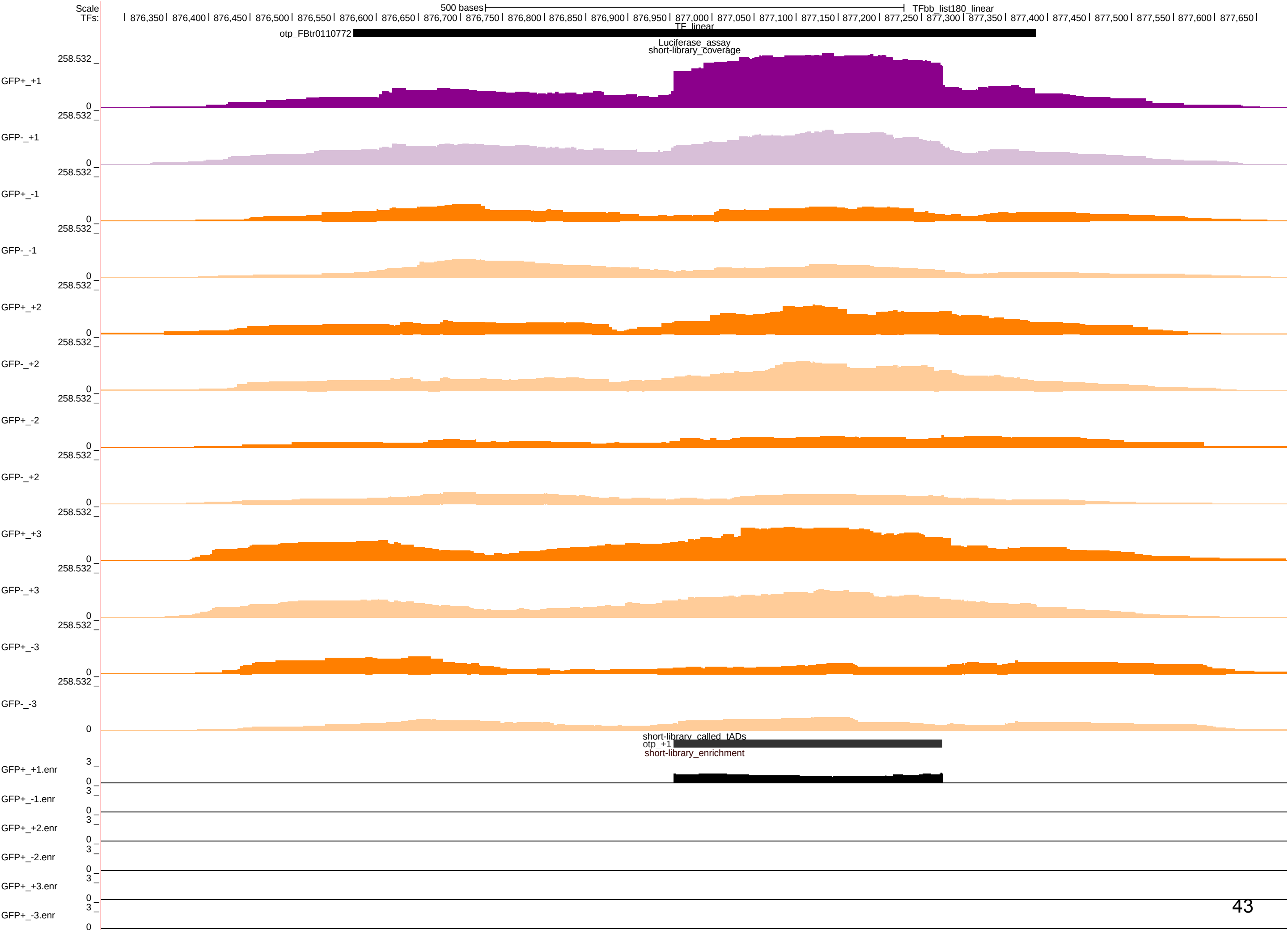

Scale
500 bases
TFbb_list180_linear
TFs:
876,350
876,400
876,450
876,500
876,550
876,600
876,650
876,700
876,750
876,800
876,850
876,900
876,950
877,000
877,050
877,100
877,150
877,200
877,250
877,300
877,350
877,400
877,450
877,500
877,550
877,600
877,650
TF_linear
otp_FBtr0110772
Luciferase_assay
short-library_coverage
258.532
0
GFP+_+1
GFP-_+1
GFP+_-1
GFP-_-1
GFP+_+2
GFP-_+2
GFP+_-2
GFP-_+2
GFP+_+3
GFP-_+3
GFP+_-3
GFP-_-3
short-library_called_tADs
otp_+1
short-library_enrichment
3
GFP+_+1.enr
GFP+_-1.enr
GFP+_+2.enr
GFP+_-2.enr
GFP+_+3.enr
GFP+_-3.enr

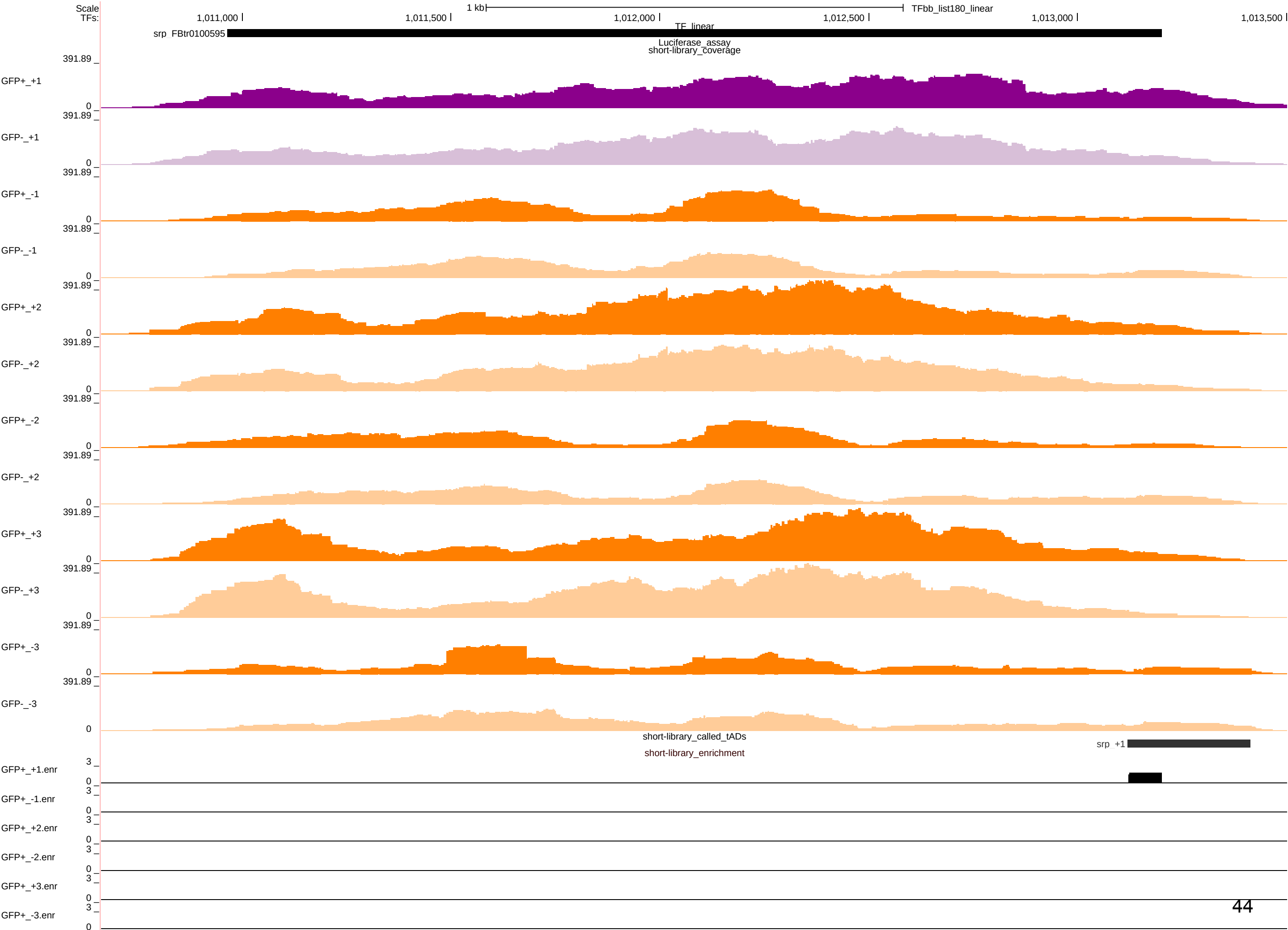
Scale
TFs:
1 kb
TFbb_list180_linear
1,011,000
1,011,500
1,012,000
1,012,500
1,013,000
1,013,500
TF_linear
srp_FBtr0100595
Luciferase_assay
short-library_coverage
391.89
0
GFP+_+1
GFP-_+1
GFP+_-1
GFP-_-1
GFP+_+2
GFP-_+2
GFP+_-2
GFP-_+2
GFP+_+3
GFP-_+3
GFP+_-3
GFP-_-3
short-library_called_tADs
srp_+1
short-library_enrichment
3
GFP+_+1.enr
GFP+_-1.enr
GFP+_+2.enr
GFP+_-2.enr
GFP+_+3.enr
GFP+_-3.enr

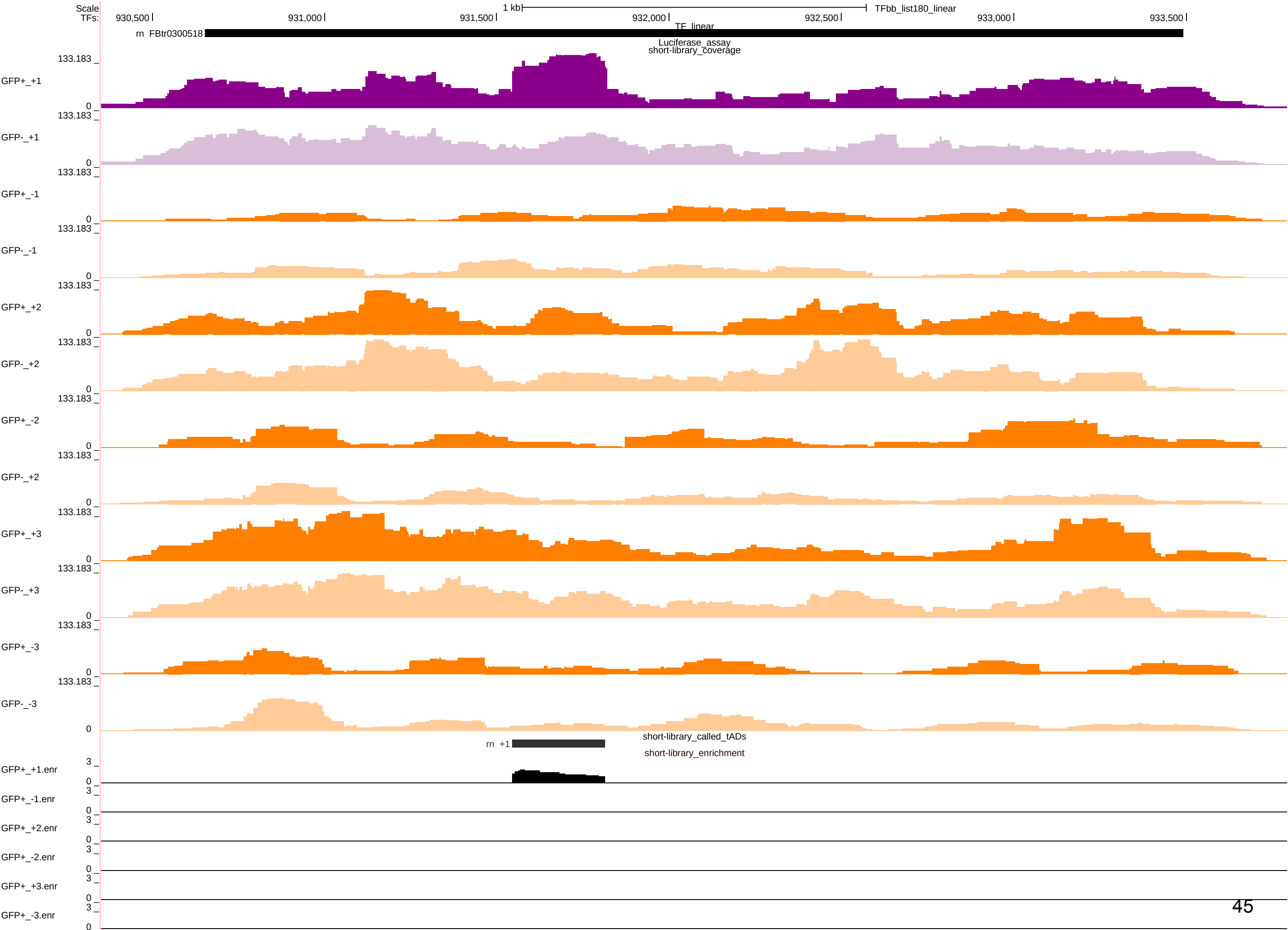
Scale
1 kb
TFbb_list180_linear
TFs:
930,500
931,000
931,500
932,000
932,500
933,000
933,500
TF_linear
rn_FBtr0300518
Luciferase_assay
short-library_coverage
133.183 _
0 _
GFP+_+1
GFP-_+1
GFP+_-1
GFP-_-1
GFP+_+2
GFP-_+2
GFP+_-2
GFP-_+2
GFP+_+3
GFP-_+3
GFP+_-3
GFP-_-3
short-library_called_tADs
rn_+1
short-library_enrichment
3 _
0 _
GFP+_+1.enr
GFP+_-1.enr
GFP+_+2.enr
GFP+_-2.enr
GFP+_+3.enr
GFP+_-3.enr

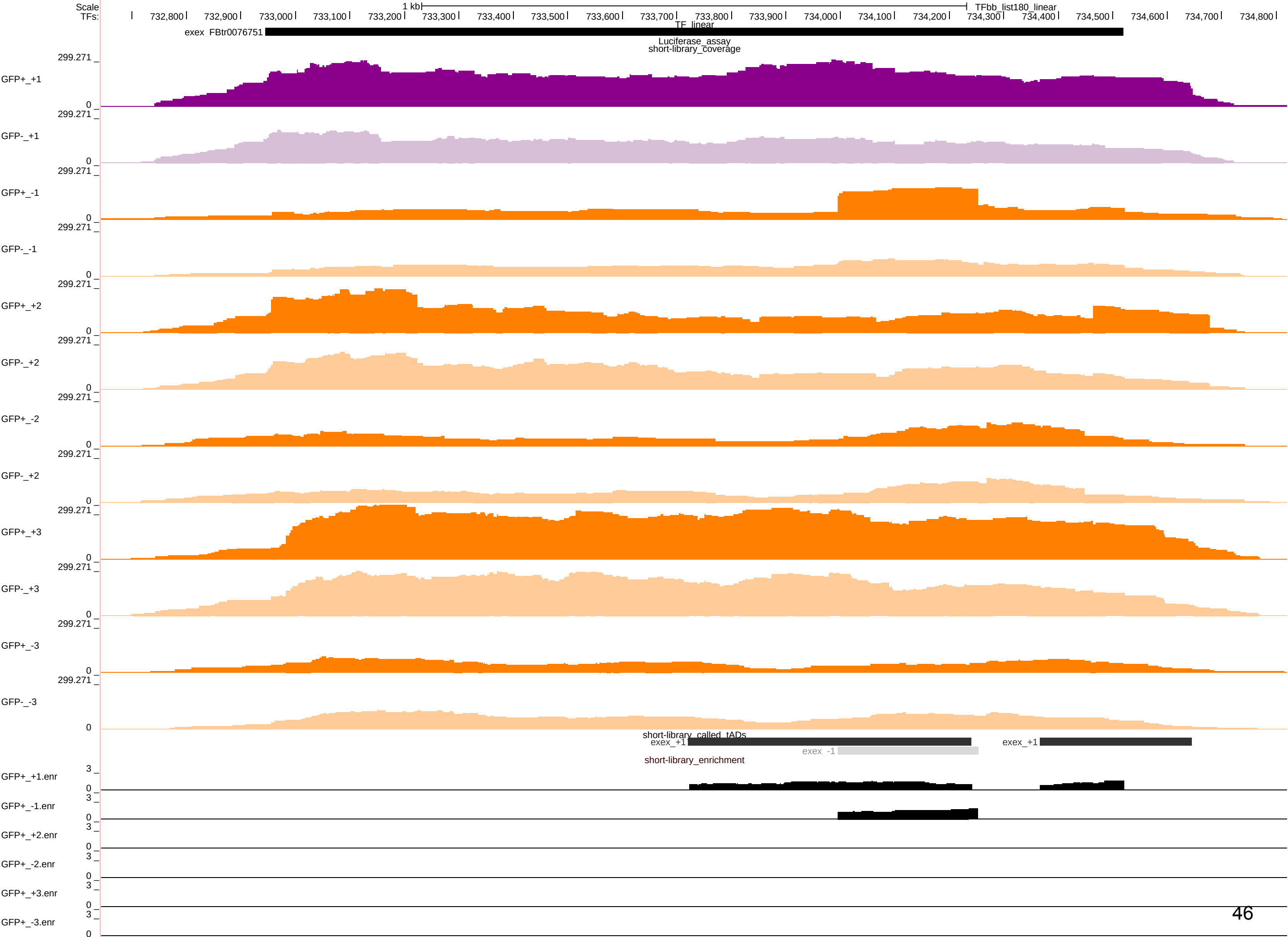
Scale
TFs:
1 kb
TFbb_list180_linear
732,800
732,900
733,000
733,100
733,200
733,300
733,400
733,500
733,600
733,700
733,800
733,900
734,000
734,100
734,200
734,300
734,400
734,500
734,600
734,700
734,800
TF_linear
exex FBtr0076751
Luciferase_assay
short-library_coverage
GFP+_+1
GFP-_+1
GFP+_-1
GFP-_-1
GFP+_+2
GFP-_+2
GFP+_-2
GFP-_+2
GFP+_+3
GFP-_+3
GFP+_-3
GFP-_-3
299.271
0
short-library_called_tADs
exex_+1
exex_-1
short-library_enrichment
GFP+_+1.enr
GFP+_-1.enr
GFP+_+2.enr
GFP+_-2.enr
GFP+_+3.enr
GFP+_-3.enr
3
0

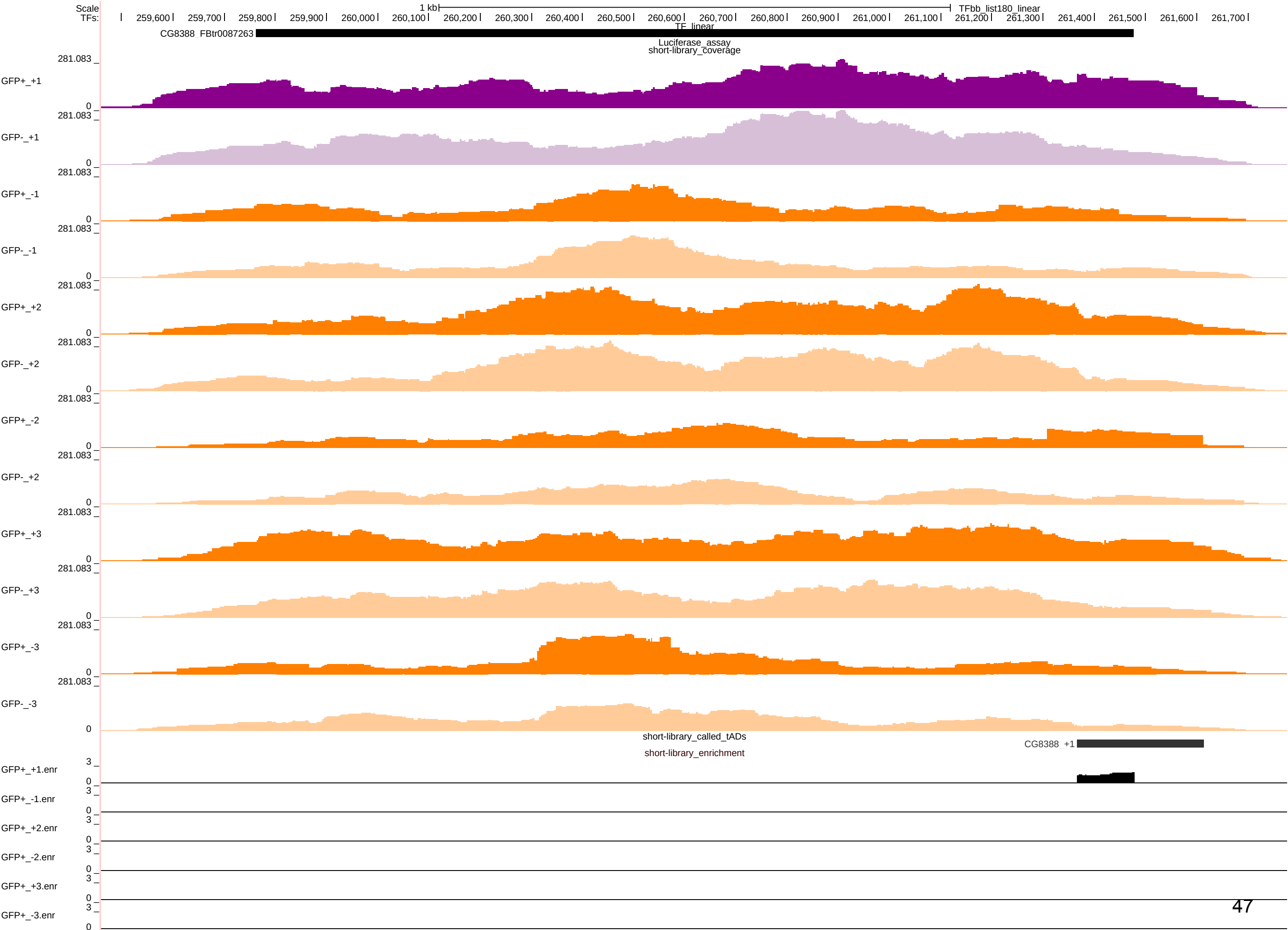
Scale
TFs:
1 kb
TFbb_list180_linear
259,600
259,700
259,800
259,900
260,000
260,100
260,200
260,300
260,400
260,500
260,600
260,700
260,800
260,900
261,000
261,100
261,200
261,300
261,400
261,500
261,600
261,700
TF_linear
CG8388 FBtr0087263
Luciferase_assay
short-library_coverage
GFP+_+1
GFP-_+1
GFP+_-1
GFP-_-1
GFP+_+2
GFP-_+2
GFP+_-2
GFP-_+2
GFP+_+3
GFP-_+3
GFP+_-3
GFP-_-3
281.083
0
short-library_called_tADs
CG8388_+1
short-library_enrichment
GFP+_+1.enr
GFP+_-1.enr
GFP+_+2.enr
GFP+_-2.enr
GFP+_+3.enr
GFP+_-3.enr
3
0

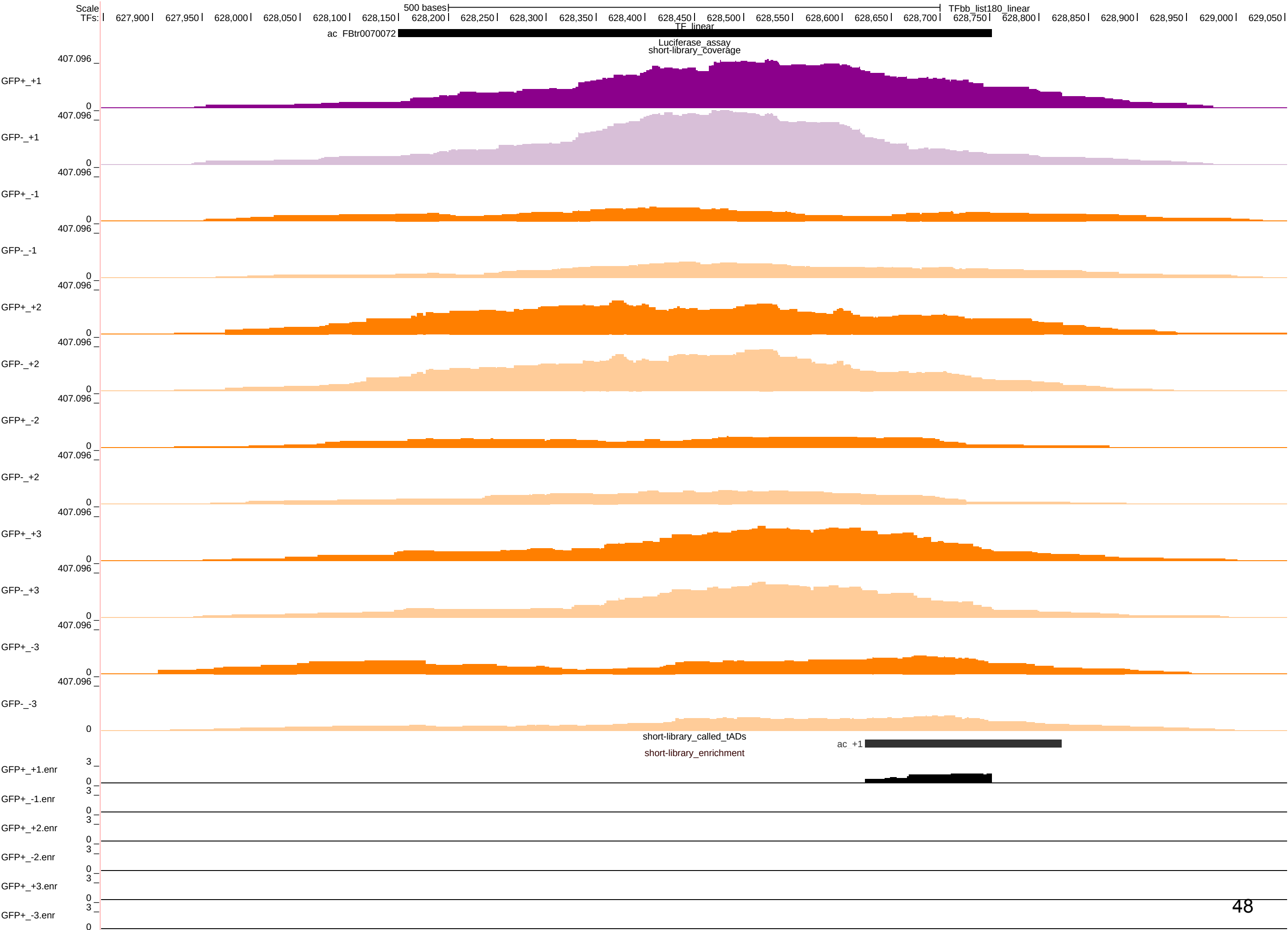
Scale
500 bases
TFbb_list180_linear
TFs:
627,900
627,950
628,000
628,050
628,100
628,150
628,200
628,250
628,300
628,350
628,400
628,450
628,500
628,550
628,600
628,650
628,700
628,750
628,800
628,850
628,900
628,950
629,000
629,050
TF_linear
ac FBtr0070072
Luciferase_assay
short-library_coverage
GFP+_+1
GFP-_+1
GFP+_-1
GFP-_-1
GFP+_+2
GFP-_+2
GFP+_-2
GFP-_+2
GFP+_+3
GFP-_+3
GFP+_-3
GFP-_-3
407.096
0
short-library_called_tADs
ac_+1
short-library_enrichment
GFP+_+1.enr
GFP+_-1.enr
GFP+_+2.enr
GFP+_-2.enr
GFP+_+3.enr
GFP+_-3.enr
3
0

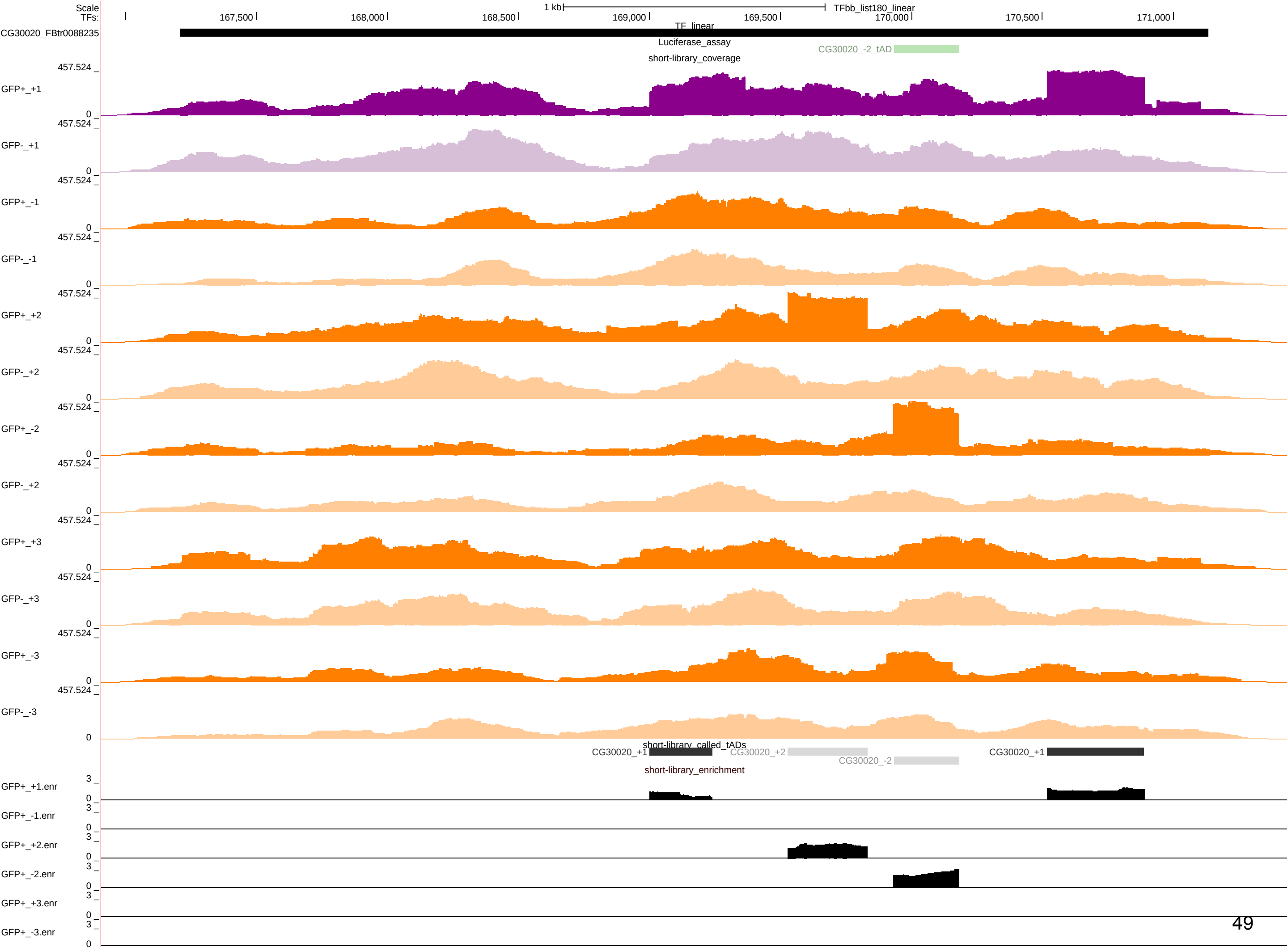

Scale
TFs:
1 kb
167,500
168,000
168,500
169,000
169,500
170,000
170,500
171,000
TFbb_list180_linear
TF_linear
CG30020 FBtr0088235
Luciferase_assay
CG30020 -2 tAD
short-library_coverage
457.524
0
GFP+_+1
GFP-_+1
GFP+_-1
GFP-_-1
GFP+_+2
GFP-_+2
GFP+_-2
GFP-_+2
GFP+_+3
GFP-_+3
GFP+_-3
GFP-_-3
short-library_called_tADs
CG30020_+1
CG30020_+2
CG30020_-2
CG30020_+1
short-library_enrichment
3
0
GFP+_+1.enr
GFP+_-1.enr
GFP+_+2.enr
GFP+_-2.enr
GFP+_+3.enr
GFP+_-3.enr

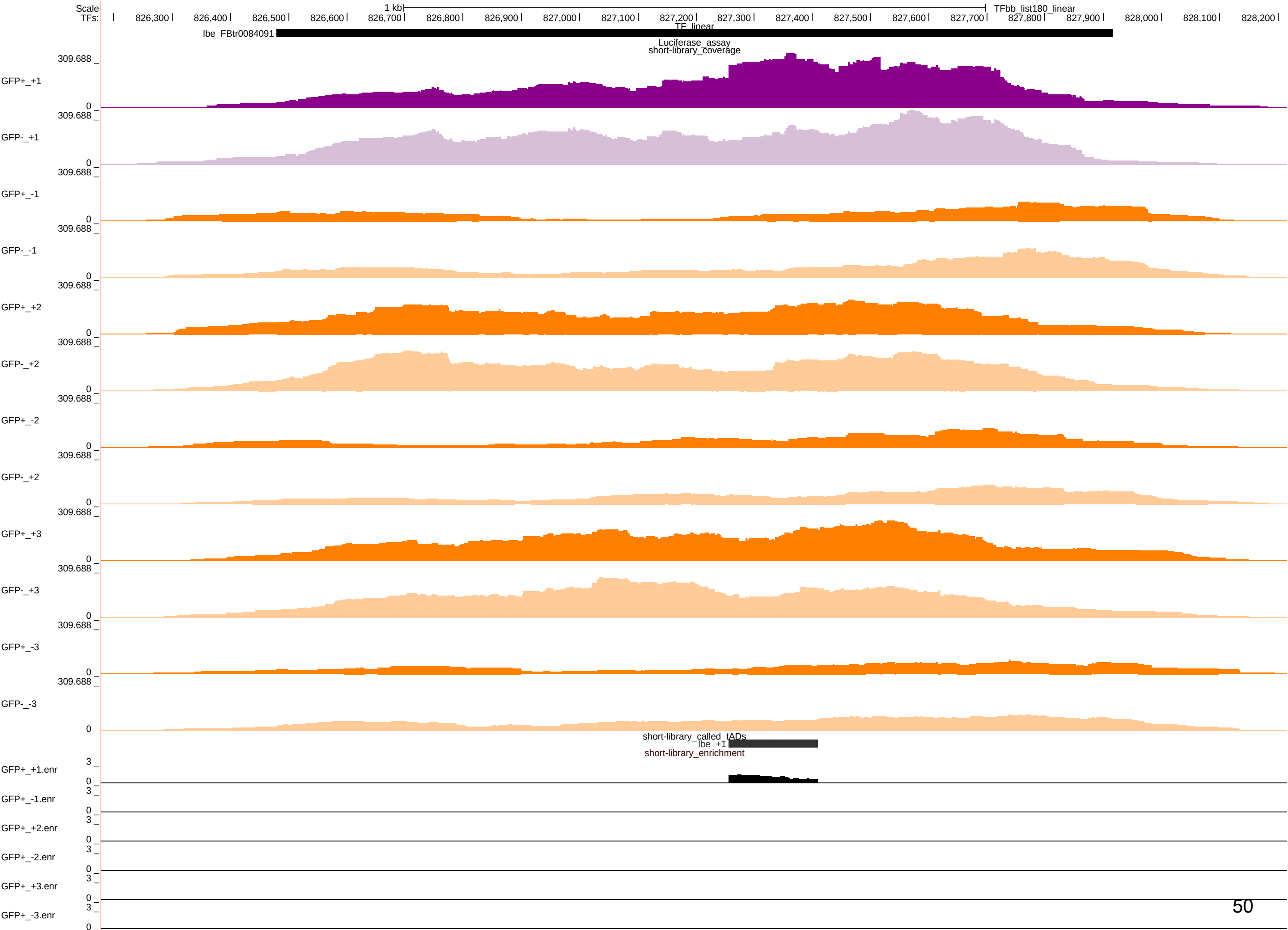
Scale
TFs:
1 kb
826,300
826,400
826,500
826,600
826,700
826,800
826,900
827,000
827,100
827,200
827,300
827,400
827,500
827,600
827,700
827,800
827,900
828,000
828,100
828,200
TFbb_list180_linear
TF_linear
lbe_FBtr0084091
Luciferase_assay
short-library_coverage
309.688
0
GFP+_+1
GFP-_+1
GFP+_-1
GFP-_-1
GFP+_+2
GFP-_+2
GFP+_-2
GFP-_+2
GFP+_+3
GFP-_+3
GFP+_-3
GFP-_-3
short-library_called_tADs
lbe_+1
short-library_enrichment
3
GFP+_+1.enr
GFP+_-1.enr
GFP+_+2.enr
GFP+_-2.enr
GFP+_+3.enr
GFP+_-3.enr

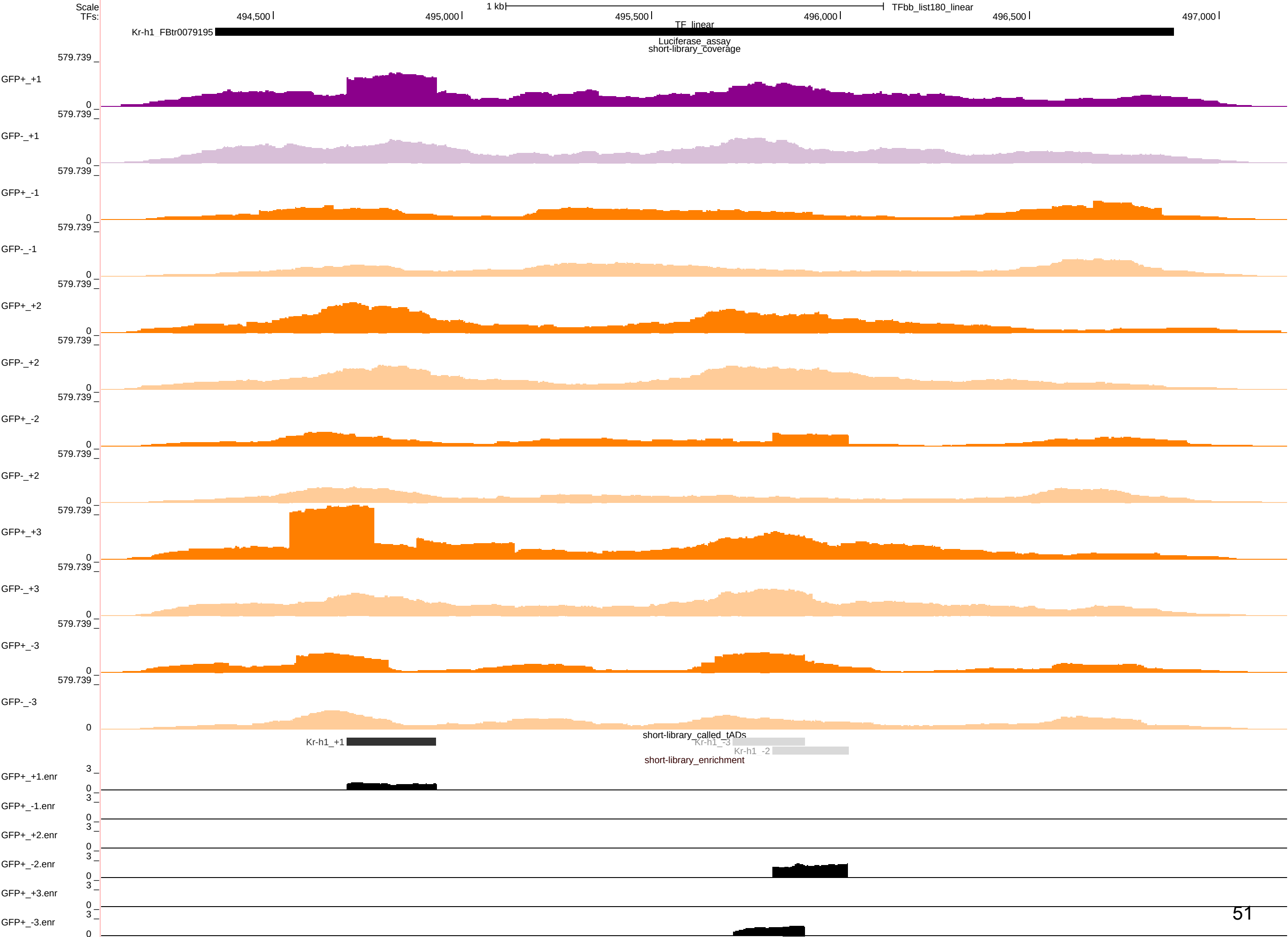

Scale
TFs:
1 kb
TFbb_list180_linear
494,500
495,000
495,500
496,000
496,500
497,000
TF_linear
Kr-h1 FBtr0079195
Luciferase_assay
short-library_coverage
GFP+_+1
GFP-_+1
GFP+_-1
GFP-_-1
GFP+_+2
GFP-_+2
GFP+_-2
GFP-_+2
GFP+_+3
GFP-_+3
GFP+_-3
GFP-_-3
579.739
0
short-library_called_tADs
Kr-h1_+1
Kr-h1_-3
Kr-h1_-2
short-library_enrichment
GFP+_+1.enr
GFP+_-1.enr
GFP+_+2.enr
GFP+_-2.enr
GFP+_+3.enr
GFP+_-3.enr
3
0

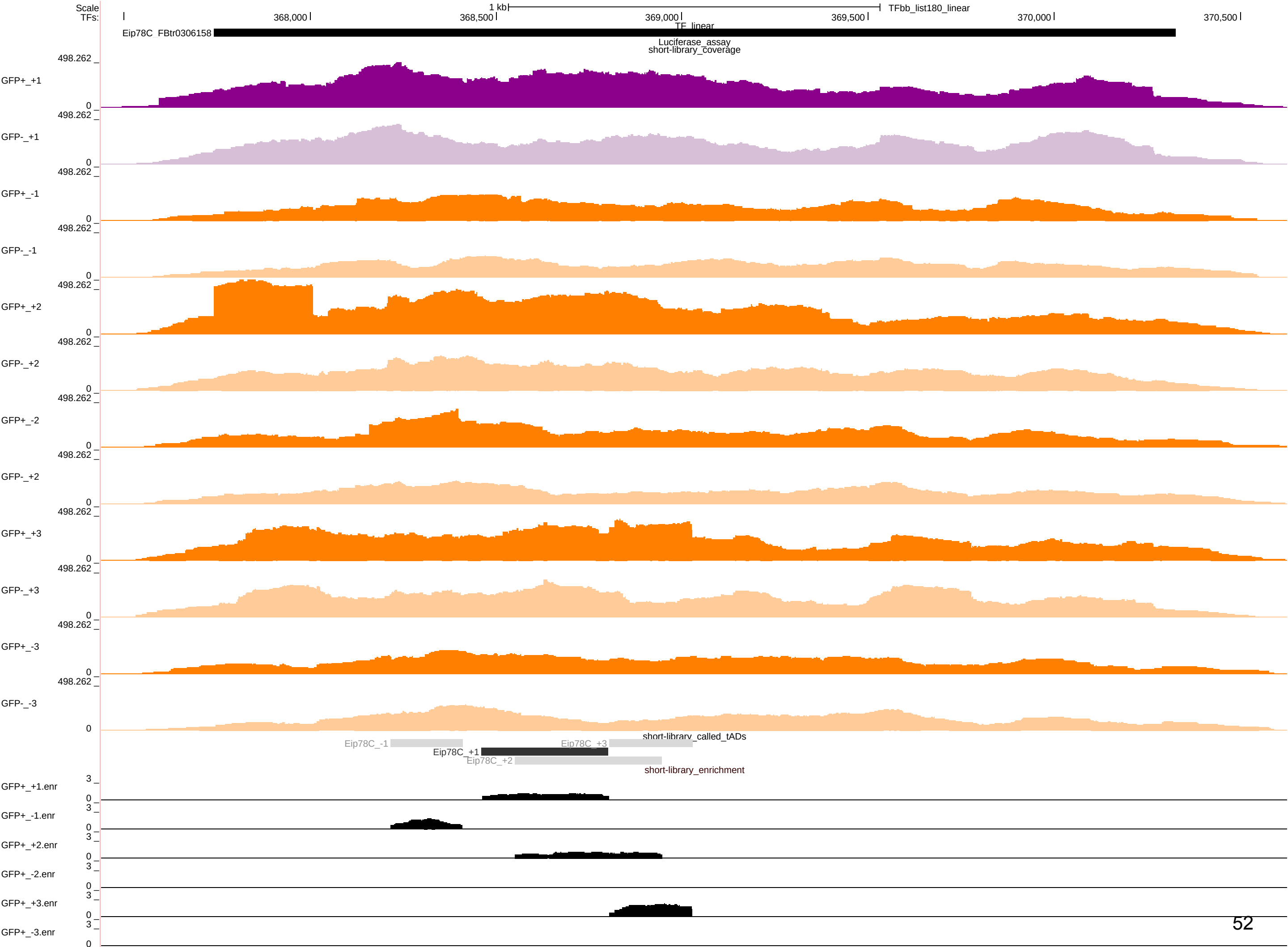
Scale
TFs:
1 kb
TFbb_list180_linear
368,000
368,500
369,000
369,500
370,000
370,500
TF_linear
Eip78C FBtr0306158
Luciferase_assay
short-library_coverage
498.262
0
GFP+_+1
GFP-_+1
GFP+_-1
GFP-_-1
GFP+_+2
GFP-_+2
GFP+_-2
GFP-_+2
GFP+_+3
GFP-_+3
GFP+_-3
GFP-_-3
short-library_called_tADs
Eip78C_-1
Eip78C_+3
Eip78C_+1
Eip78C_+2
short-library_enrichment
3
GFP+_+1.enr
GFP+_-1.enr
GFP+_+2.enr
GFP+_-2.enr
GFP+_+3.enr
GFP+_-3.enr

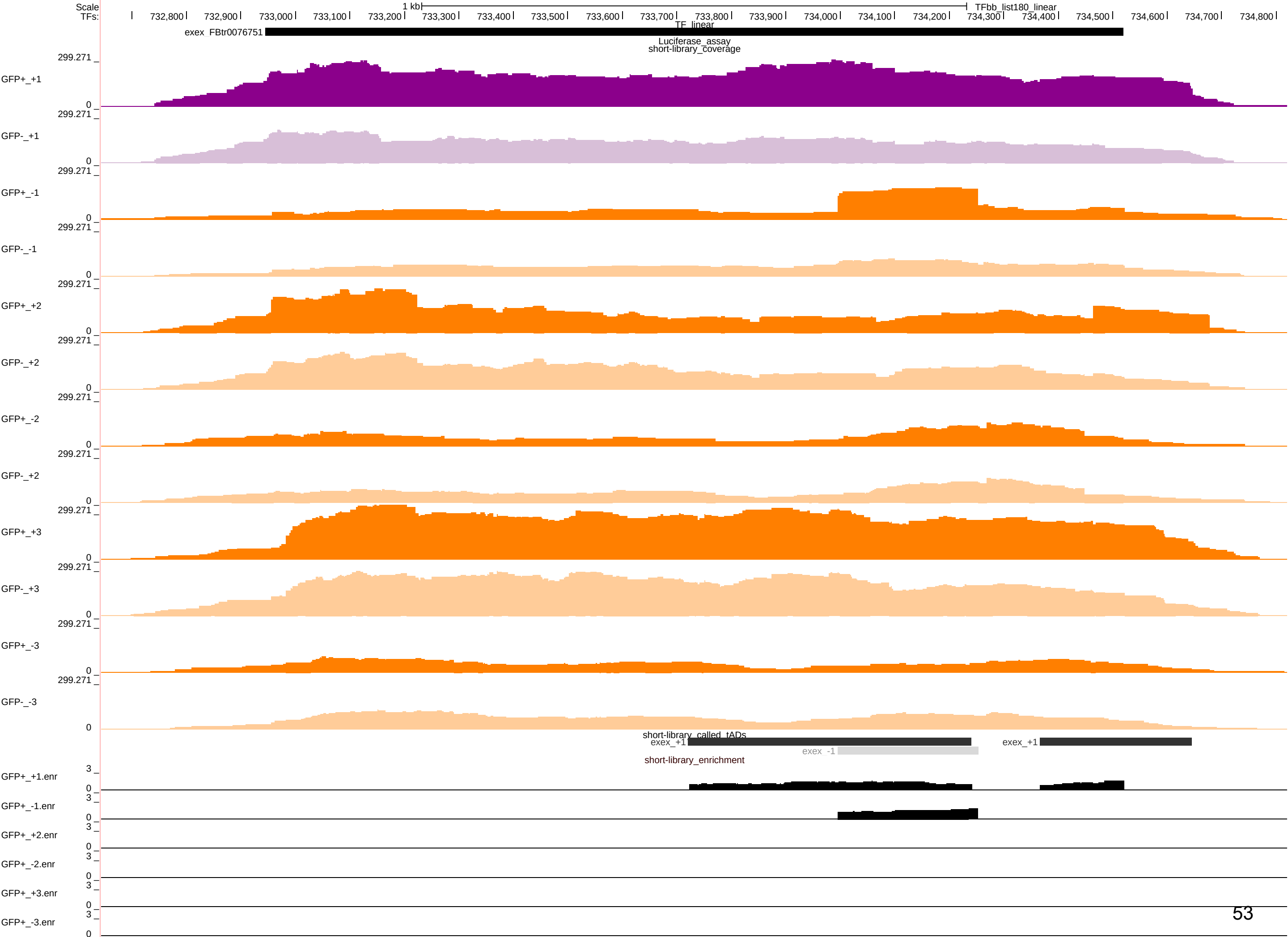
Scale
TFs:
1 kb
TFbb_list180_linear
732,800
732,900
733,000
733,100
733,200
733,300
733,400
733,500
733,600
733,700
733,800
733,900
734,000
734,100
734,200
734,300
734,400
734,500
734,600
734,700
734,800
TF_linear
exex_FBtr0076751
Luciferase_assay
short-library_coverage
GFP+_+1
GFP-_+1
GFP+_-1
GFP-_-1
GFP+_+2
GFP-_+2
GFP+_-2
GFP-_+2
GFP+_+3
GFP-_+3
GFP+_-3
GFP-_-3
299.271
0
short-library_called_tADs
exex_+1
exex_+1
exex_-1
short-library_enrichment
GFP+_+1.enr
GFP+_-1.enr
GFP+_+2.enr
GFP+_-2.enr
GFP+_+3.enr
GFP+_-3.enr
3
0

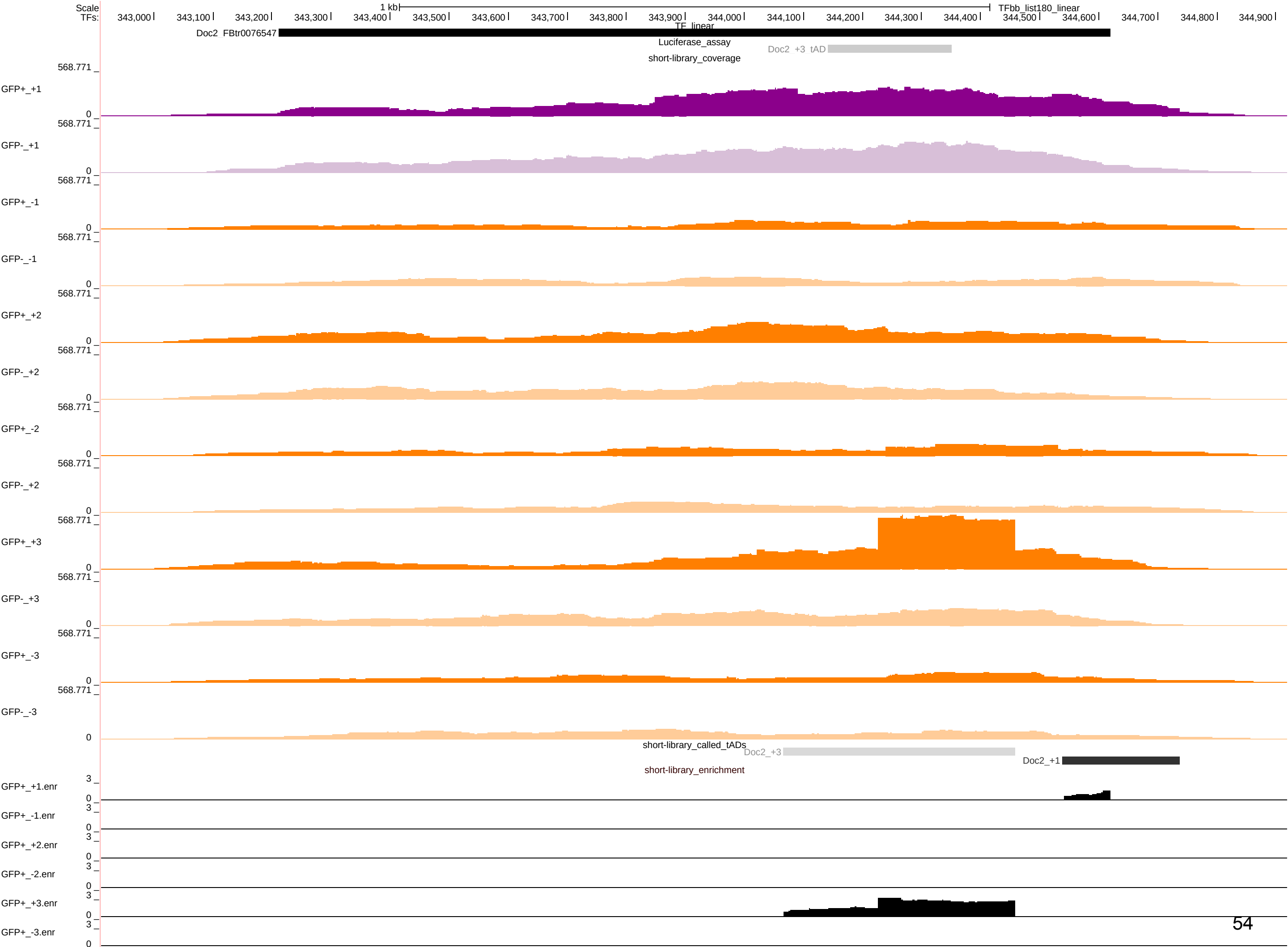

Scale
1 kb
TFbb_list180_linear
TFs:
343,000
343,100
343,200
343,300
343,400
343,500
343,600
343,700
343,800
343,900
344,000
344,100
344,200
344,300
344,400
344,500
344,600
344,700
344,800
344,900
TF_linear
Doc2 FBtr0076547
Luciferase_assay
Doc2 +3 tAD
short-library_coverage
568.771
GFP+_+1
GFP-_+1
GFP+_-1
GFP-_-1
GFP+_+2
GFP-_+2
GFP+_-2
GFP-_+2
GFP+_+3
GFP-_+3
GFP+_-3
GFP-_-3
0
short-library_called_tADs
Doc2_+3
Doc2_+1
short-library_enrichment
3
GFP+_+1.enr
GFP+_-1.enr
GFP+_+2.enr
GFP+_-2.enr
GFP+_+3.enr
GFP+_-3.enr

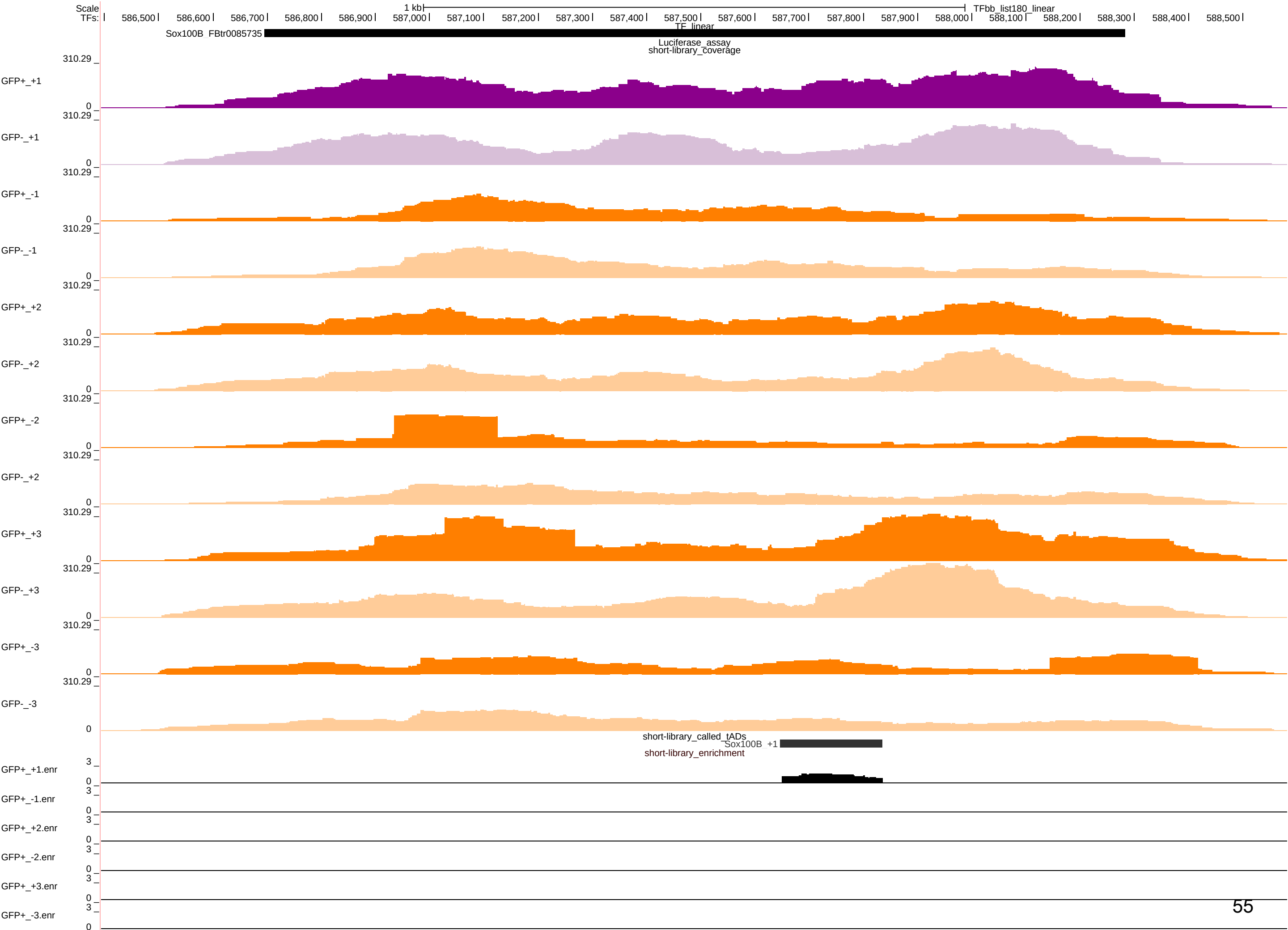

Scale
1 kb
TFbb_list180_linear
TFs:
586,500
586,600
586,700
586,800
586,900
587,000
587,100
587,200
587,300
587,400
587,500
587,600
587,700
587,800
587,900
588,000
588,100
588,200
588,300
588,400
588,500
TF_linear
Sox100B_FBtr0085735
Luciferase_assay
short-library_coverage
310.29
0
GFP+_+1
GFP-_+1
GFP+_-1
GFP-_-1
GFP+_+2
GFP-_+2
GFP+_-2
GFP-_+2
GFP+_+3
GFP-_+3
GFP+_-3
GFP-_-3
short-library_called_tADs
Sox100B_+1
short-library_enrichment
3
GFP+_+1.enr
GFP+_-1.enr
GFP+_+2.enr
GFP+_-2.enr
GFP+_+3.enr
GFP+_-3.enr

Appendix Figure S2

103 short-fragment library non-native frame tADs

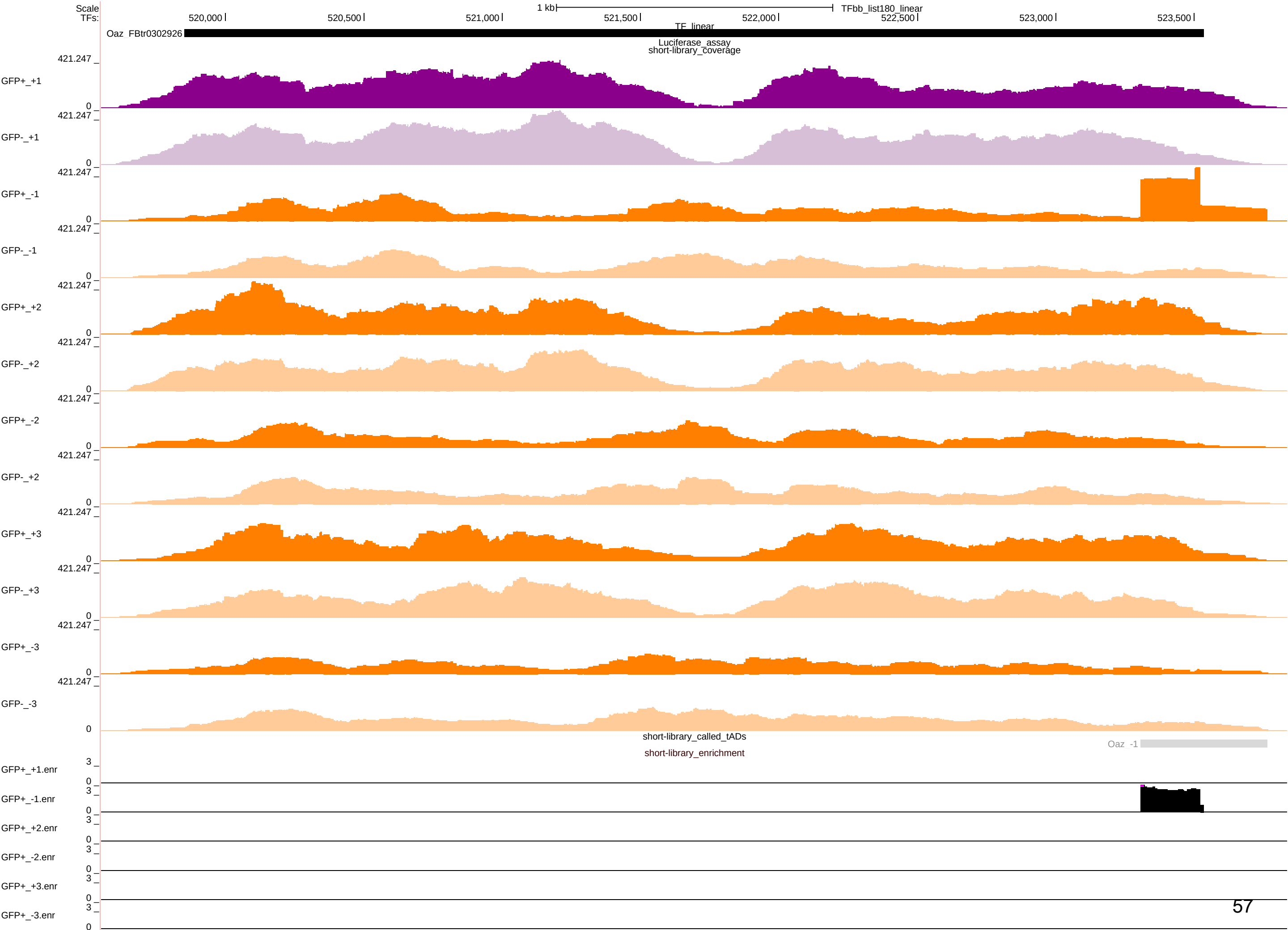

Scale
TFs:
1 kb
TFbb_list180_linear
520,000
520,500
521,000
521,500
522,000
522,500
523,000
523,500
TF_linear
Oaz FBtr0302926
Luciferase_assay
short-library_coverage
421.247
0
GFP+_+1
GFP-_+1
GFP+_-1
GFP-_-1
GFP+_+2
GFP-_+2
GFP+_-2
GFP-_+2
GFP+_+3
GFP-_+3
GFP+_-3
GFP-_-3
short-library_called_tADs
Oaz -1
short-library_enrichment
3
GFP+_+1.enr
GFP+_-1.enr
GFP+_+2.enr
GFP+_-2.enr
GFP+_+3.enr
GFP+_-3.enr

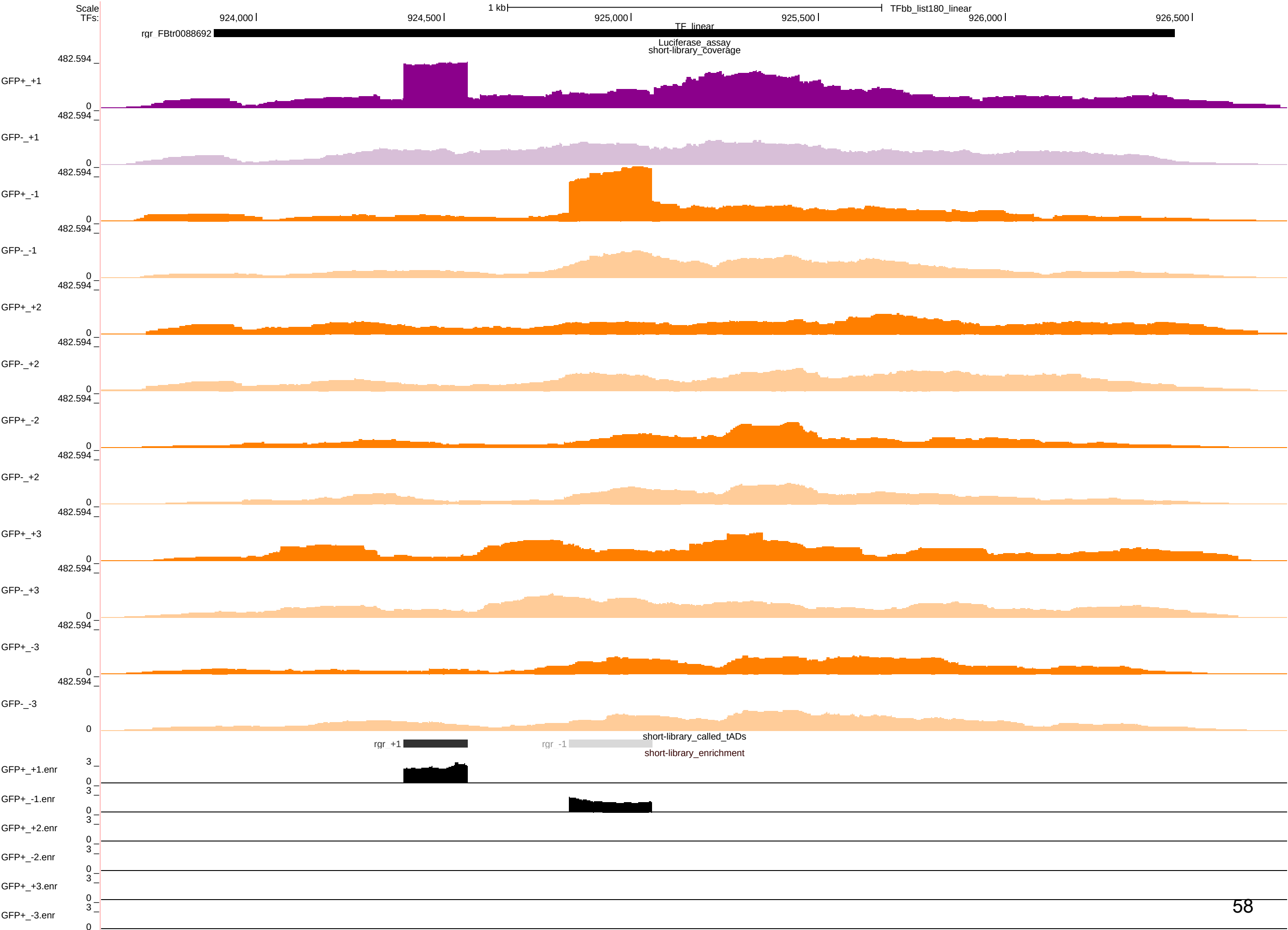

Scale
TFs:
1 kb
TFbb_list180_linear
924,000
924,500
925,000
925,500
926,000
926,500
TF_linear
rgr_FBtr0088692
Luciferase_assay
short-library_coverage
482.594
0
GFP+_+1
GFP-_+1
GFP+_-1
GFP-_-1
GFP+_+2
GFP-_+2
GFP+_-2
GFP-_+2
GFP+_+3
GFP-_+3
GFP+_-3
GFP-_-3
short-library_called_tADs
rgr_+1
rgr_-1
short-library_enrichment
3
GFP+_+1.enr
GFP+_-1.enr
GFP+_+2.enr
GFP+_-2.enr
GFP+_+3.enr
GFP+_-3.enr

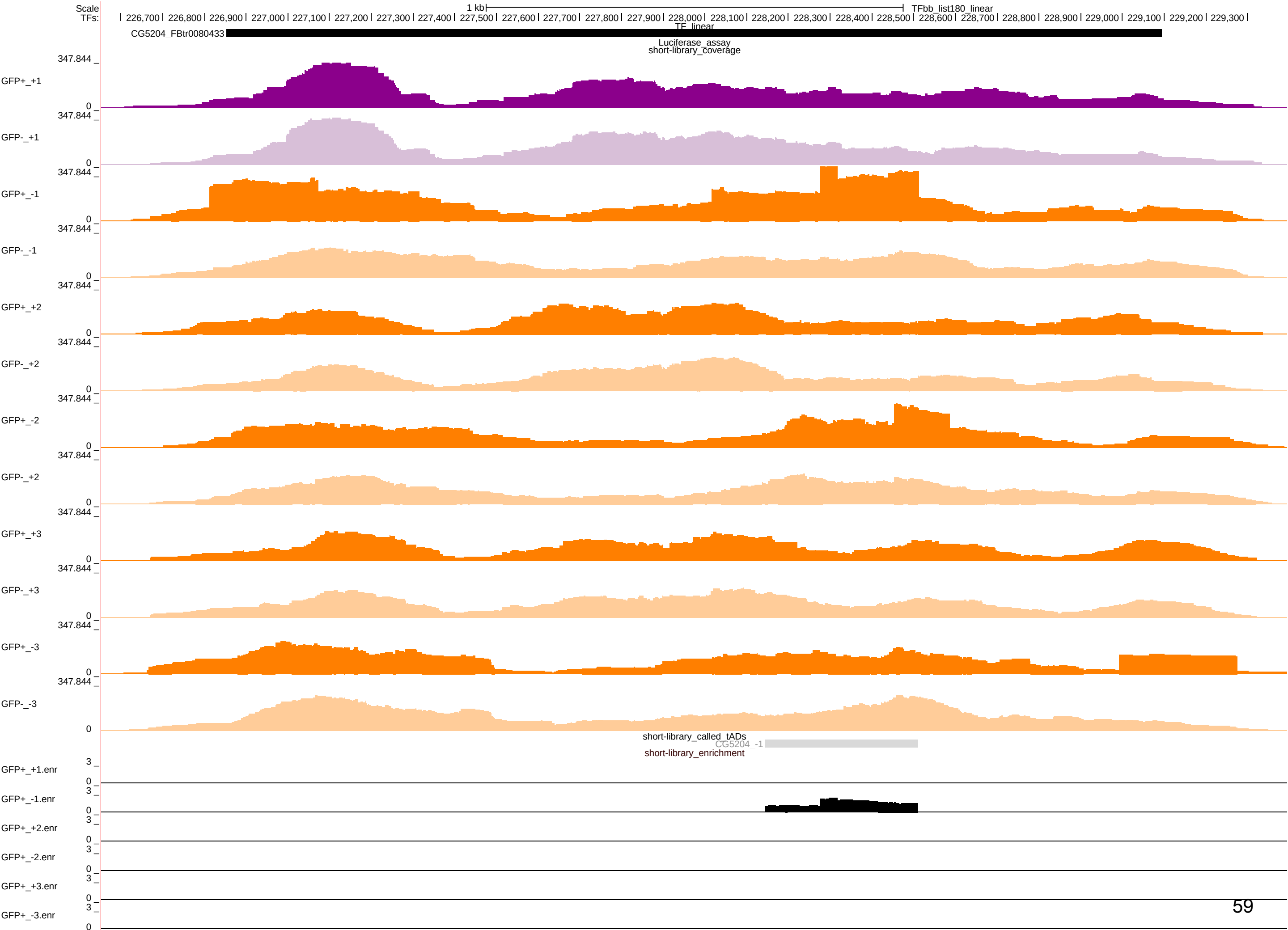
Scale
TFs:
1 kb
TFbb_list180_linear
226,700 226,800 226,900 227,000 227,100 227,200 227,300 227,400 227,500 227,600 227,700 227,800 227,900 228,000 228,100 228,200 228,300 228,400 228,500 228,600 228,700 228,800 228,900 229,000 229,100 229,200 229,300
TF_linear
CG5204 FBtr0080433
Luciferase_assay
short-library_coverage
347.844
0
GFP+_+1
GFP-_+1
GFP+_-1
GFP-_-1
GFP+_+2
GFP-_+2
GFP+_-2
GFP-_+2
GFP+_+3
GFP-_+3
GFP+_-3
GFP-_-3
short-library_called_tADs
CG5204 -1
short-library_enrichment
3
0
GFP+_+1.enr
GFP+_-1.enr
GFP+_+2.enr
GFP+_-2.enr
GFP+_+3.enr
GFP+_-3.enr

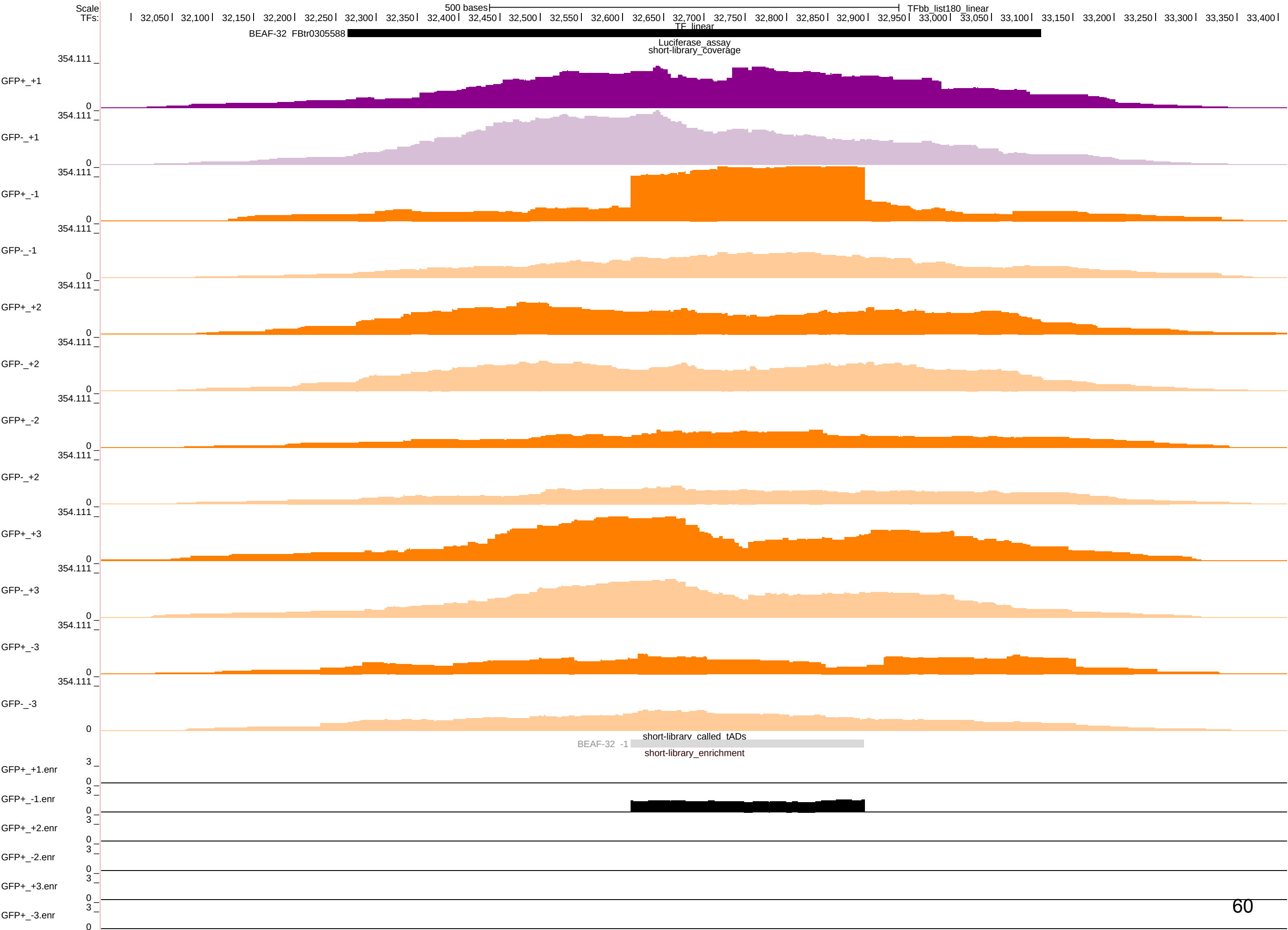

Scale
TFs:
500 bases
TFbb_list180_linear
32,050
32,100
32,150
32,200
32,250
32,300
32,350
32,400
32,450
32,500
32,550
32,600
32,650
32,700
32,750
32,800
32,850
32,900
32,950
33,000
33,050
33,100
33,150
33,200
33,250
33,300
33,350
33,400
TF_linear
BEAF-32 FBtr0305588
Luciferase_assay
short-library_coverage
354.111
0
GFP+_+1
GFP-_+1
GFP+_-1
GFP-_-1
GFP+_+2
GFP-_+2
GFP+_-2
GFP-_+2
GFP+_+3
GFP-_+3
GFP+_-3
GFP-_-3
short-library_called_tADs
BEAF-32_-1
short-library_enrichment
3
GFP+_+1.enr
GFP+_-1.enr
GFP+_+2.enr
GFP+_-2.enr
GFP+_+3.enr
GFP+_-3.enr

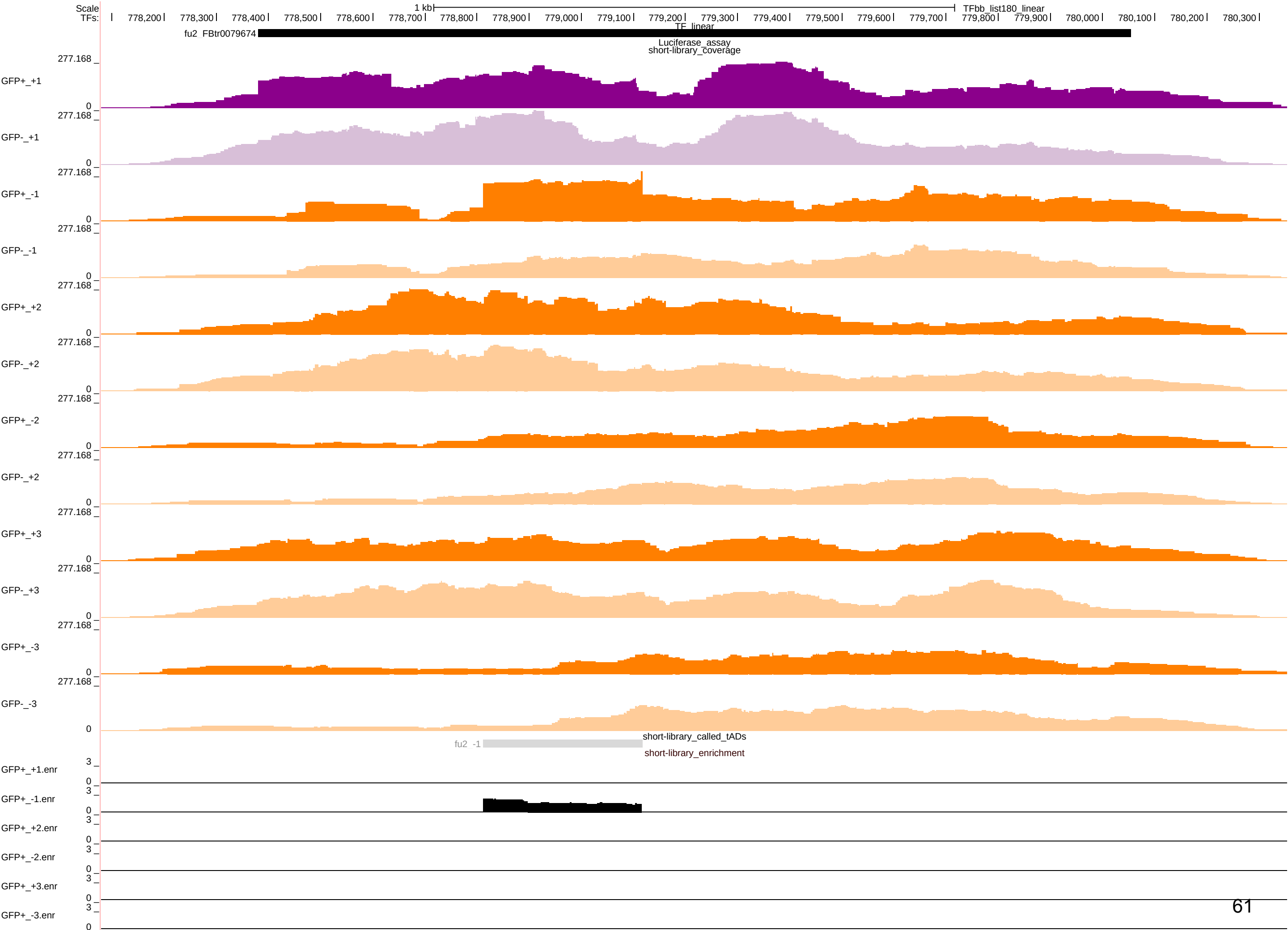

Scale
TFs:
1 kb
TFbb_list180_linear
778,200
778,300
778,400
778,500
778,600
778,700
778,800
778,900
779,000
779,100
779,200
779,300
779,400
779,500
779,600
779,700
779,800
779,900
780,000
780,100
780,200
780,300
TF_linear
fu2 FBtr0079674
Luciferase_assay
short-library_coverage
277.168
0
GFP+_+1
GFP-_+1
GFP+_-1
GFP-_-1
GFP+_+2
GFP-_+2
GFP+_-2
GFP-_+2
GFP+_+3
GFP-_+3
GFP+_-3
GFP-_-3
short-library_called_tADs
fu2 -1
short-library_enrichment
3
GFP+_+1.enr
GFP+_-1.enr
GFP+_+2.enr
GFP+_-2.enr
GFP+_+3.enr
GFP+_-3.enr

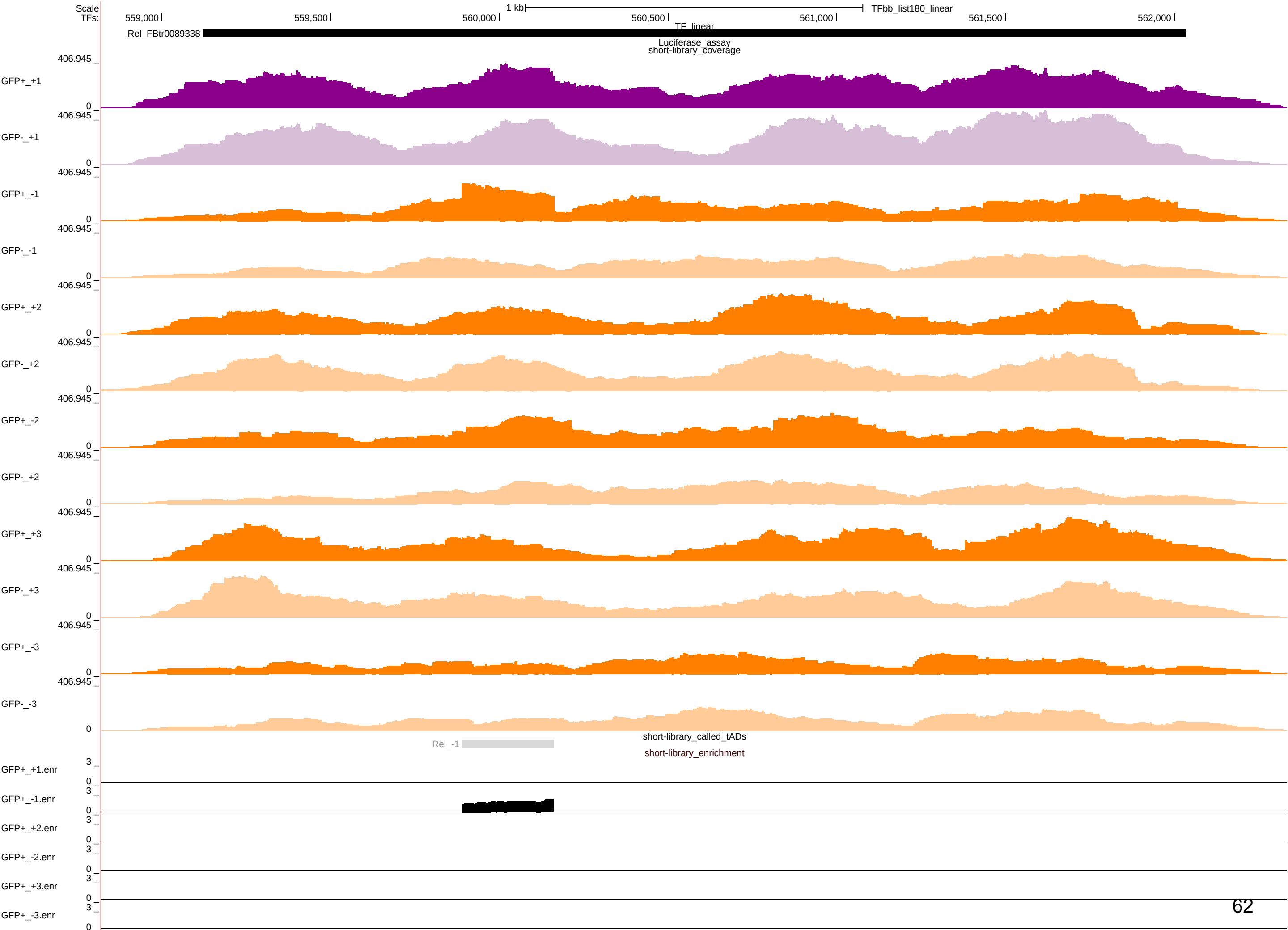

Scale
TFs:
559,000
559,500
560,000
1 kb
560,500
561,000
TFbb_list180_linear
561,500
562,000
TF_linear
Rel FBtr0089338
Luciferase_assay
short-library_coverage
406.945
GFP+_+1
0
GFP-_+1
GFP+_-1
GFP-_-1
GFP+_+2
GFP-_+2
GFP+_-2
GFP-_+2
GFP+_+3
GFP-_+3
GFP+_-3
GFP-_-3
short-library_called_tADs
Rel_-1
short-library_enrichment
3
GFP+_+1.enr
GFP+_-1.enr
GFP+_+2.enr
GFP+_-2.enr
GFP+_+3.enr
GFP+_-3.enr

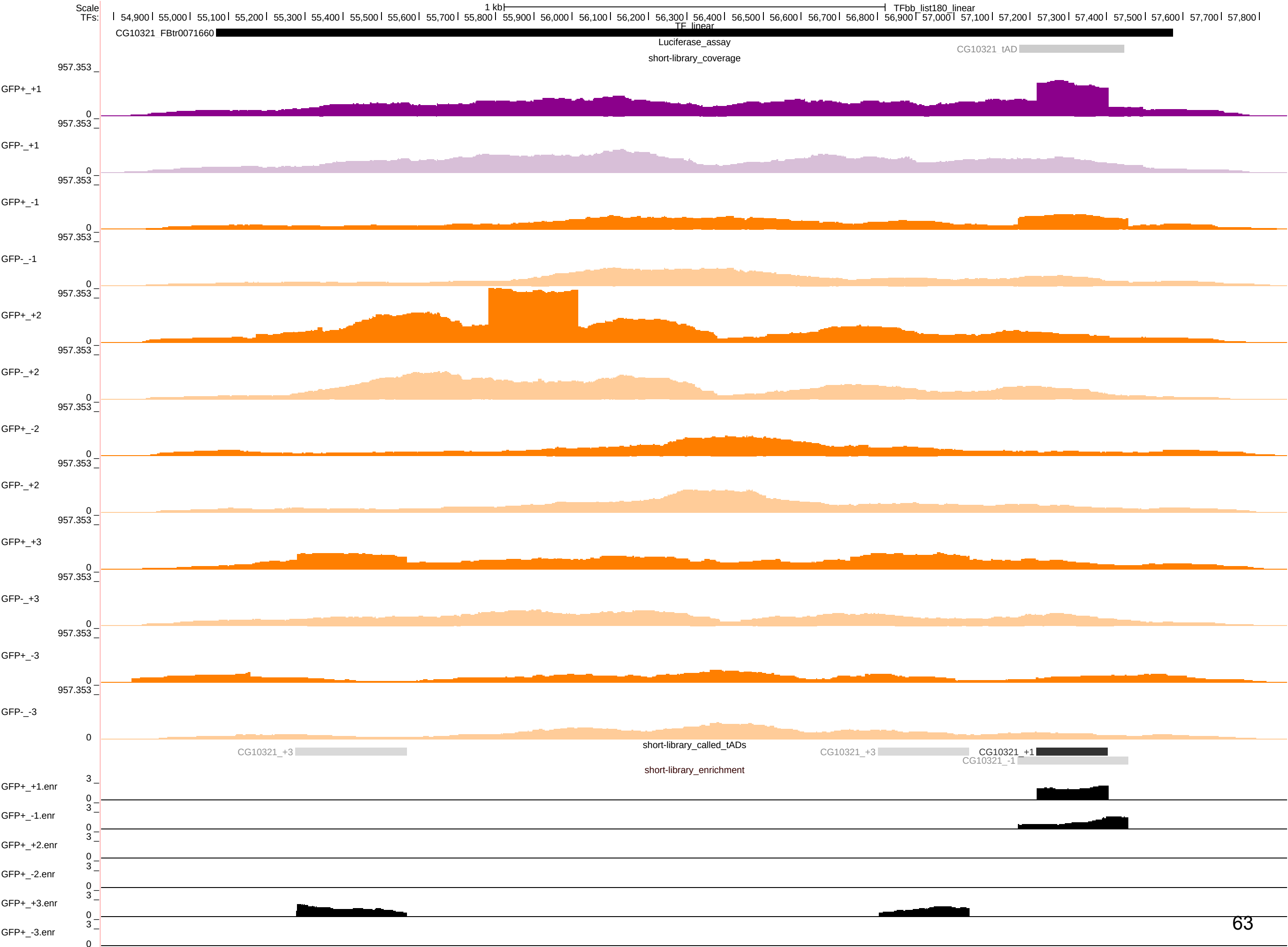
Scale
TFs:
1 kb
TFbb_list180_linear
54,900 55,000 55,100 55,200 55,300 55,400 55,500 55,600 55,700 55,800 55,900 56,000 56,100 56,200 56,300 56,400 56,500 56,600 56,700 56,800 56,900 57,000 57,100 57,200 57,300 57,400 57,500 57,600 57,700 57,800
TF_linear
CG10321 FBtr0071660
Luciferase_assay
CG10321 tAD
short-library_coverage
957.353
0
GFP+_+1
GFP-_+1
GFP+_-1
GFP-_-1
GFP+_+2
GFP-_+2
GFP+_-2
GFP-_+2
GFP+_+3
GFP-_+3
GFP+_-3
GFP-_-3
short-library_called_tADs
CG10321_+3
CG10321_+3
CG10321_+1
CG10321_-1
short-library_enrichment
3
0
GFP+_+1.enr
GFP+_-1.enr
GFP+_+2.enr
GFP+_-2.enr
GFP+_+3.enr
GFP+_-3.enr

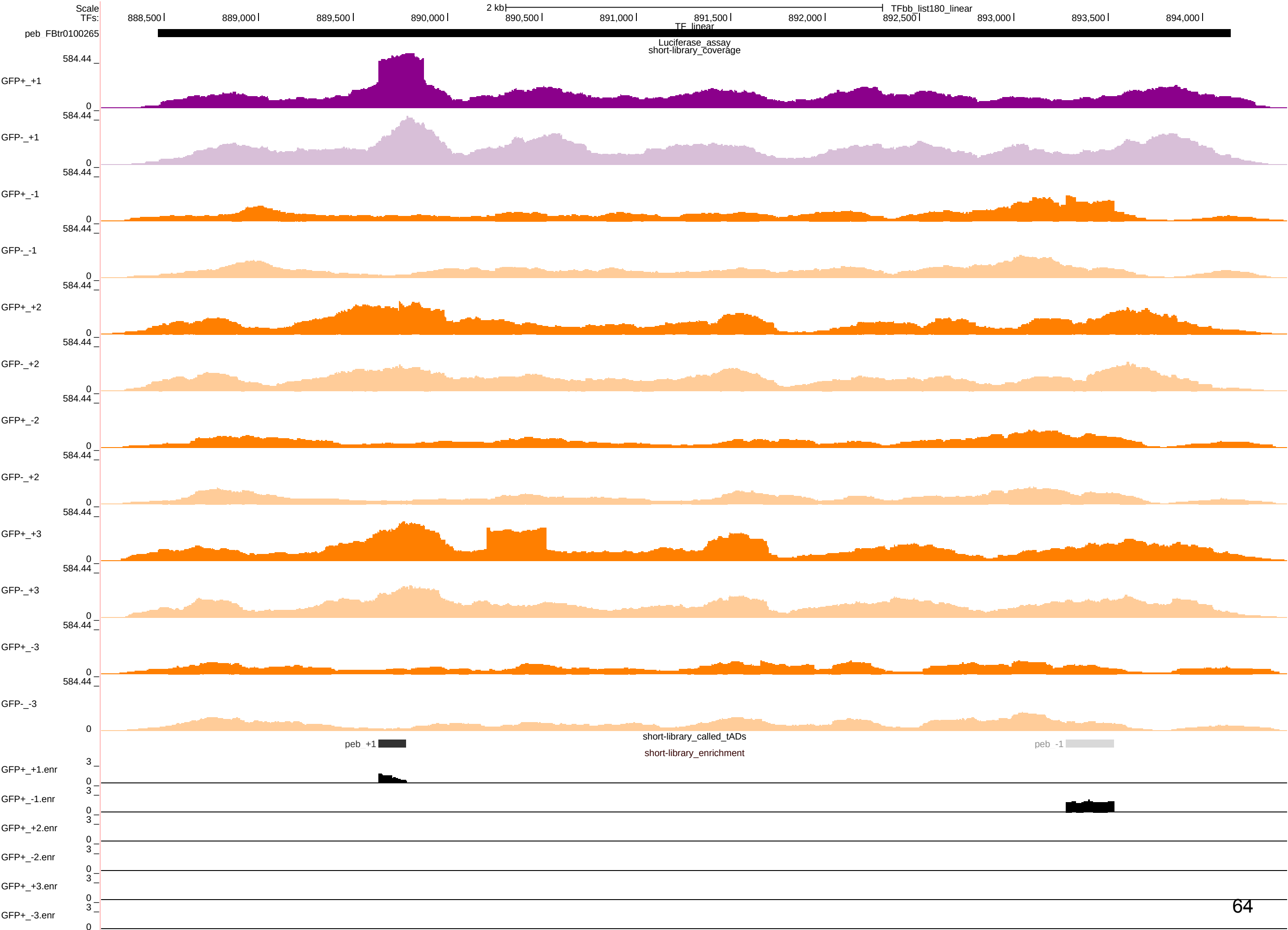
Scale
2 kb
TFbb_list180_linear
TFs:
888,500
889,000
889,500
890,000
890,500
891,000
891,500
892,000
892,500
893,000
893,500
894,000
TF_linear
peb_FBtr0100265
Luciferase_assay
short-library_coverage
584.44
0
GFP+_+1
GFP-_+1
GFP+_-1
GFP-_-1
GFP+_+2
GFP-_+2
GFP+_-2
GFP-_+2
GFP+_+3
GFP-_+3
GFP+_-3
GFP-_-3
short-library_called_tADs
peb_+1
peb_-1
short-library_enrichment
3
GFP+_+1.enr
GFP+_-1.enr
GFP+_+2.enr
GFP+_-2.enr
GFP+_+3.enr
GFP+_-3.enr

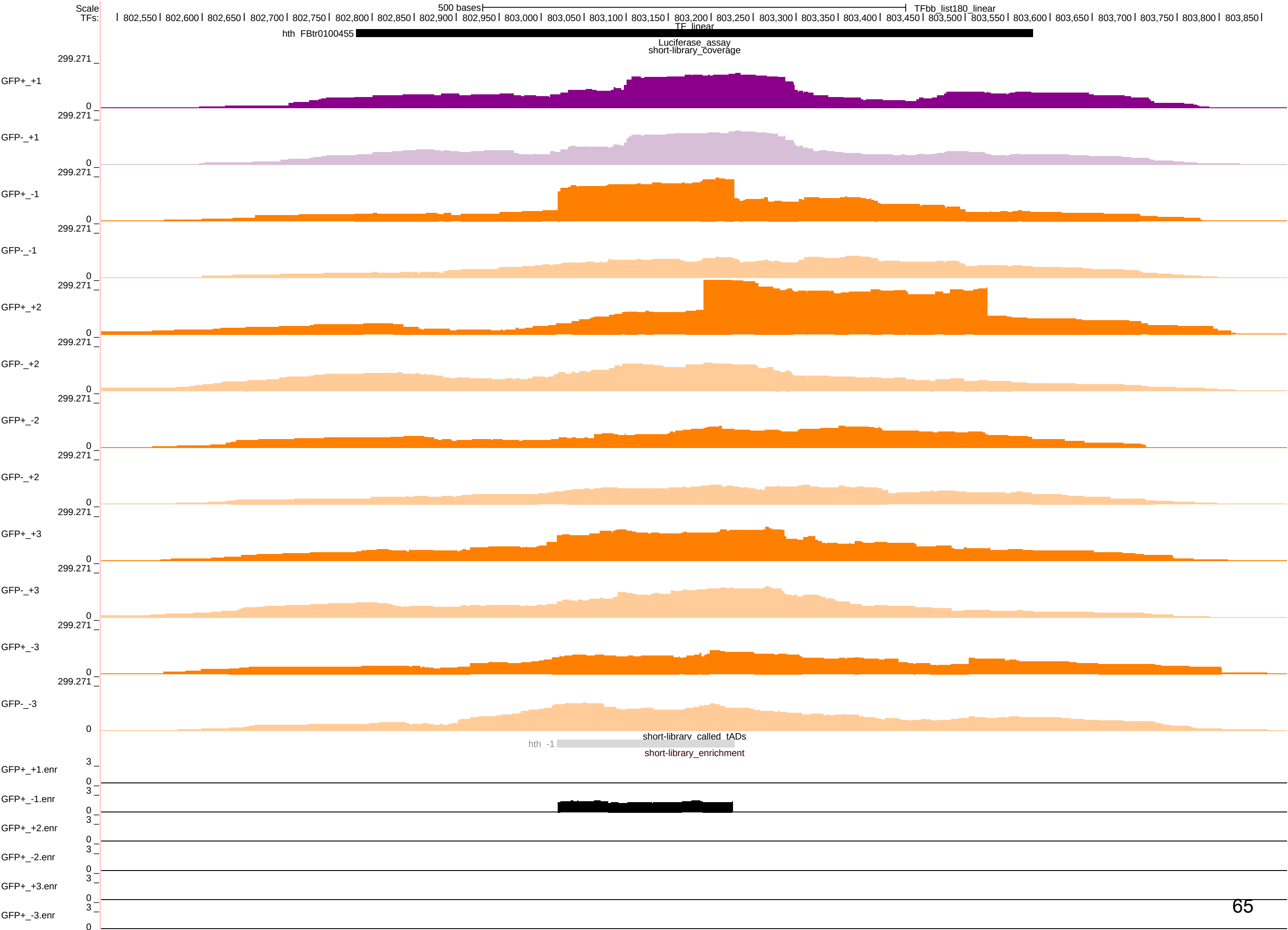
Scale
TFs:
500 bases
TFbb_list180_linear
802,550 802,600 802,650 802,700 802,750 802,800 802,850 802,900 802,950 803,000 803,050 803,100 803,150 803,200 803,250 803,300 803,350 803,400 803,450 803,500 803,550 803,600 803,650 803,700 803,750 803,800 803,850
TF_linear
hth_FBtr0100455
Luciferase_assay
short-library_coverage
GFP+_+1
GFP-_+1
GFP+_-1
GFP-_-1
GFP+_+2
GFP-_+2
GFP+_-2
GFP-_+2
GFP+_+3
GFP-_+3
GFP+_-3
GFP-_-3
299.271
0
short-library_called_tADs
hth_-1
short-library_enrichment
GFP+_+1.enr
GFP+_-1.enr
GFP+_+2.enr
GFP+_-2.enr
GFP+_+3.enr
GFP+_-3.enr
3
0

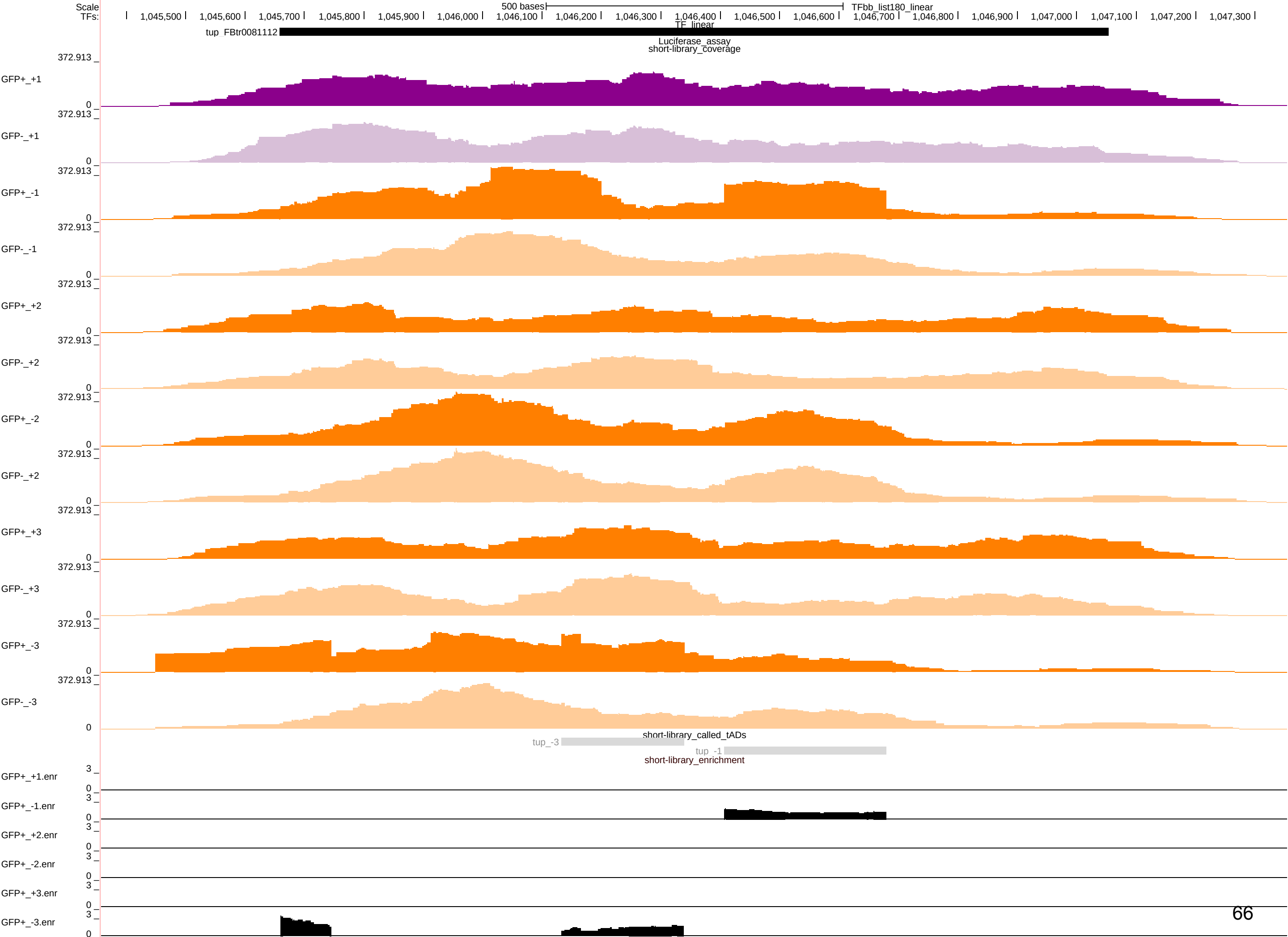
Scale
TFs:
500 bases
1,045,500
1,045,600
1,045,700
1,045,800
1,045,900
1,046,000
1,046,100
1,046,200
1,046,300
1,046,400
1,046,500
1,046,600
1,046,700
1,046,800
1,046,900
1,047,000
1,047,100
1,047,200
1,047,300
TFbb_list180_linear
TF_linear
tup FBtr0081112
Luciferase_assay
short-library_coverage
GFP+_+1
GFP-_+1
GFP+_-1
GFP-_-1
GFP+_+2
GFP-_+2
GFP+_-2
GFP-_+2
GFP+_+3
GFP-_+3
GFP+_-3
GFP-_-3
372.913
0
short-library_called_tADs
tup_-3
tup_-1
short-library_enrichment
GFP+_+1.enr
GFP+_-1.enr
GFP+_+2.enr
GFP+_-2.enr
GFP+_+3.enr
GFP+_-3.enr
3
0

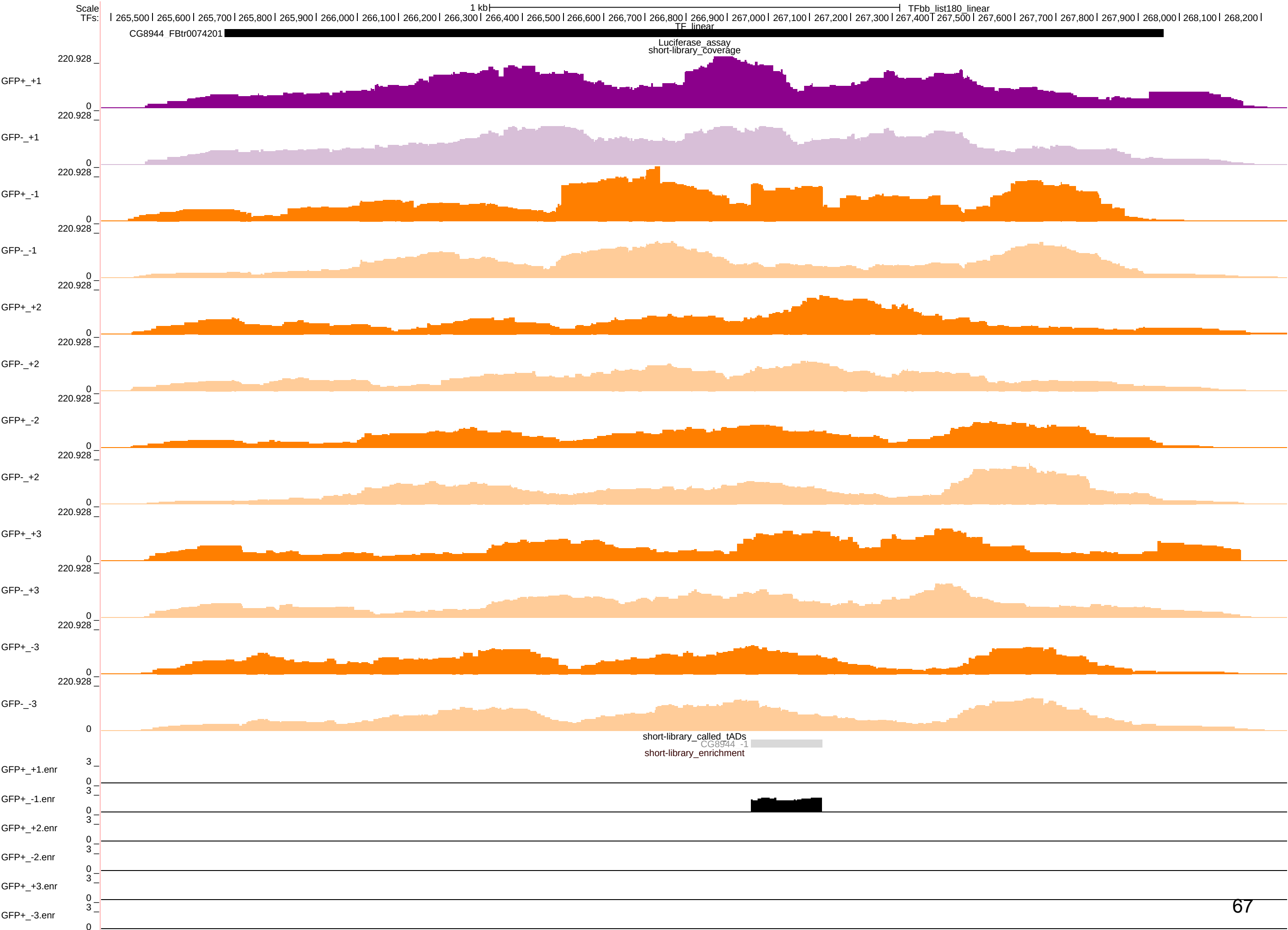

Scale
1 kb
TFbb_list180_linear
TFs:
265,500
265,600
265,700
265,800
265,900
266,000
266,100
266,200
266,300
266,400
266,500
266,600
266,700
266,800
266,900
267,000
267,100
267,200
267,300
267,400
267,500
267,600
267,700
267,800
267,900
268,000
268,100
268,200
TF_linear
CG8944 FBtr0074201
Luciferase_assay
short-library_coverage
220.928
0
GFP+_+1
GFP-_+1
GFP+_-1
GFP-_-1
GFP+_+2
GFP-_+2
GFP+_-2
GFP-_+2
GFP+_+3
GFP-_+3
GFP+_-3
GFP-_-3
short-library_called_tADs
CG8944 -1
short-library_enrichment
3
GFP+_+1.enr
GFP+_-1.enr
GFP+_+2.enr
GFP+_-2.enr
GFP+_+3.enr
GFP+_-3.enr

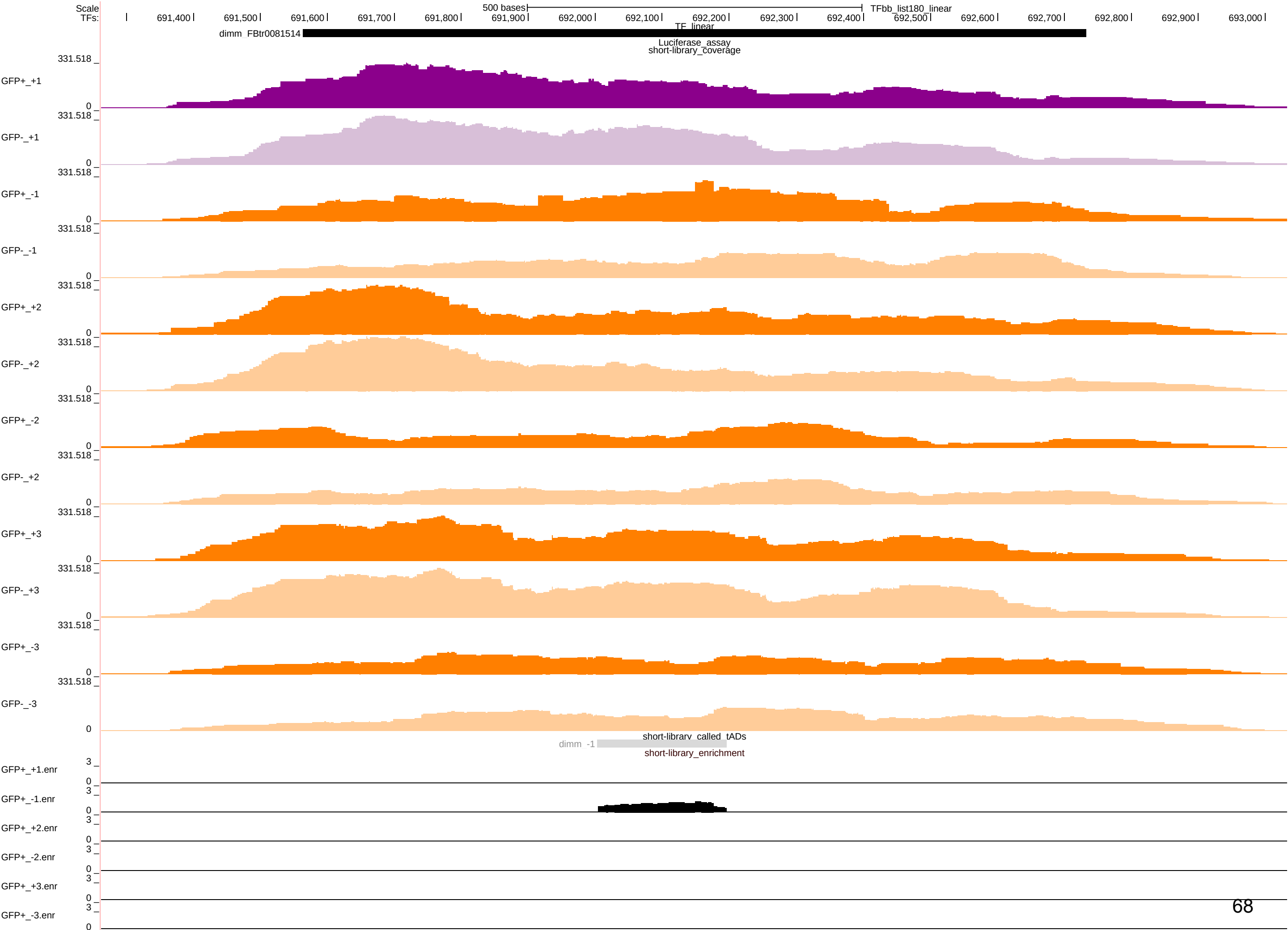

Scale
TFs:
500 bases
691,400
691,500
691,600
691,700
691,800
691,900
692,000
692,100
692,200
692,300
692,400
692,500
692,600
692,700
692,800
692,900
693,000
TFbb_list180_linear
TF_linear
dimm_FBtr0081514
Luciferase_assay
short-library_coverage
331.518
0
GFP+_+1
GFP-_+1
GFP+_-1
GFP-_-1
GFP+_+2
GFP-_+2
GFP+_-2
GFP-_+2
GFP+_+3
GFP-_+3
GFP+_-3
GFP-_-3
short-library_called_tADs
dimm_-1
short-library_enrichment
3
GFP+_+1.enr
GFP+_-1.enr
GFP+_+2.enr
GFP+_-2.enr
GFP+_+3.enr
GFP+_-3.enr

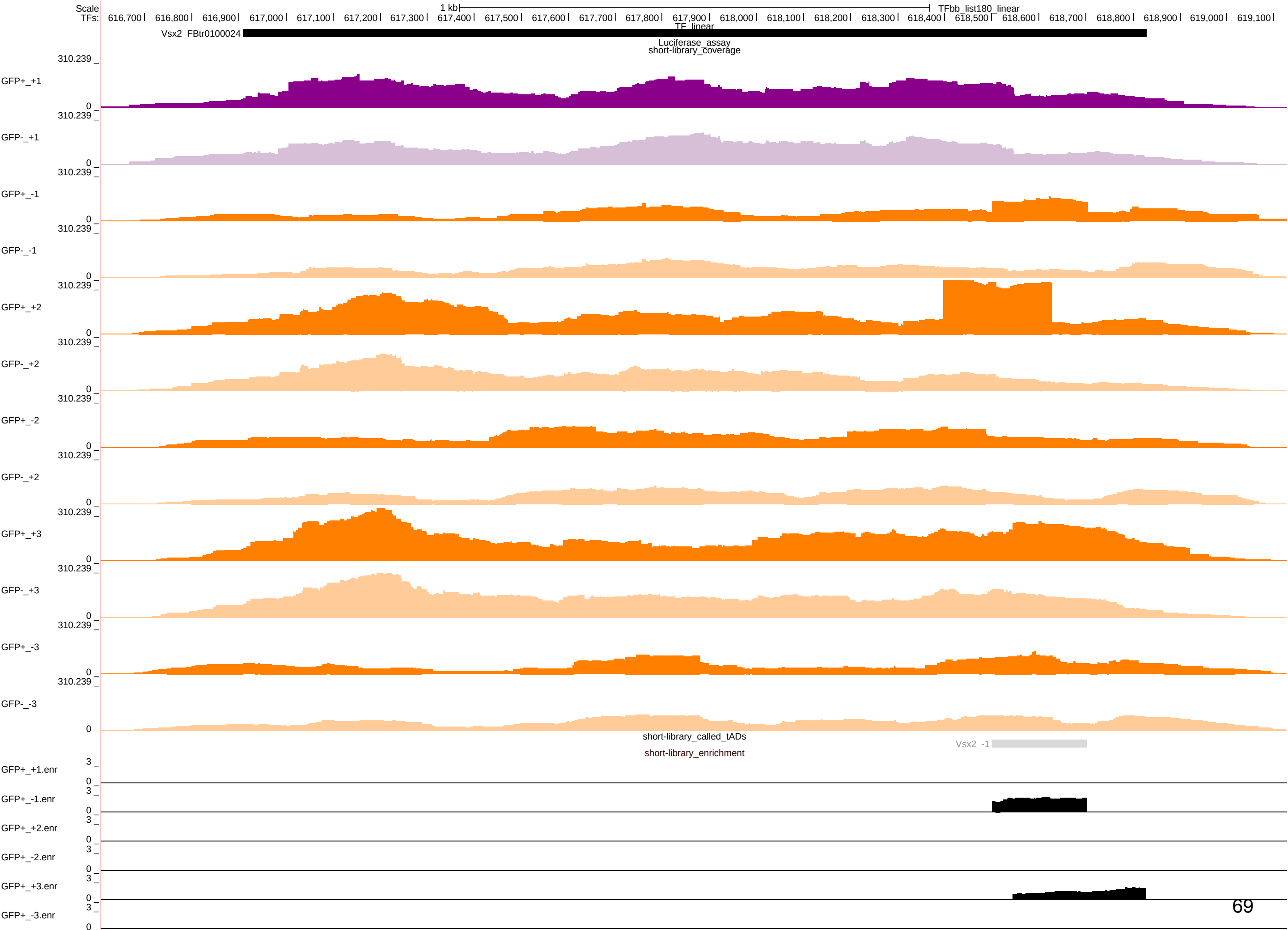
Scale
TFs:
1 kb
TFbb_list180_linear
616,700
616,800
616,900
617,000
617,100
617,200
617,300
617,400
617,500
617,600
617,700
617,800
617,900
618,000
618,100
618,200
618,300
618,400
618,500
618,600
618,700
618,800
618,900
619,000
619,100
TF_linear
Vsx2 FBtr0100024
Luciferase_assay
short-library_coverage
310.239
0
GFP+_+1
GFP-_+1
GFP+_-1
GFP-_-1
GFP+_+2
GFP-_+2
GFP+_-2
GFP-_+2
GFP+_+3
GFP-_+3
GFP+_-3
GFP-_-3
short-library_called_tADs
Vsx2 -1
short-library_enrichment
3
GFP+_+1.enr
GFP+_-1.enr
GFP+_+2.enr
GFP+_-2.enr
GFP+_+3.enr
GFP+_-3.enr

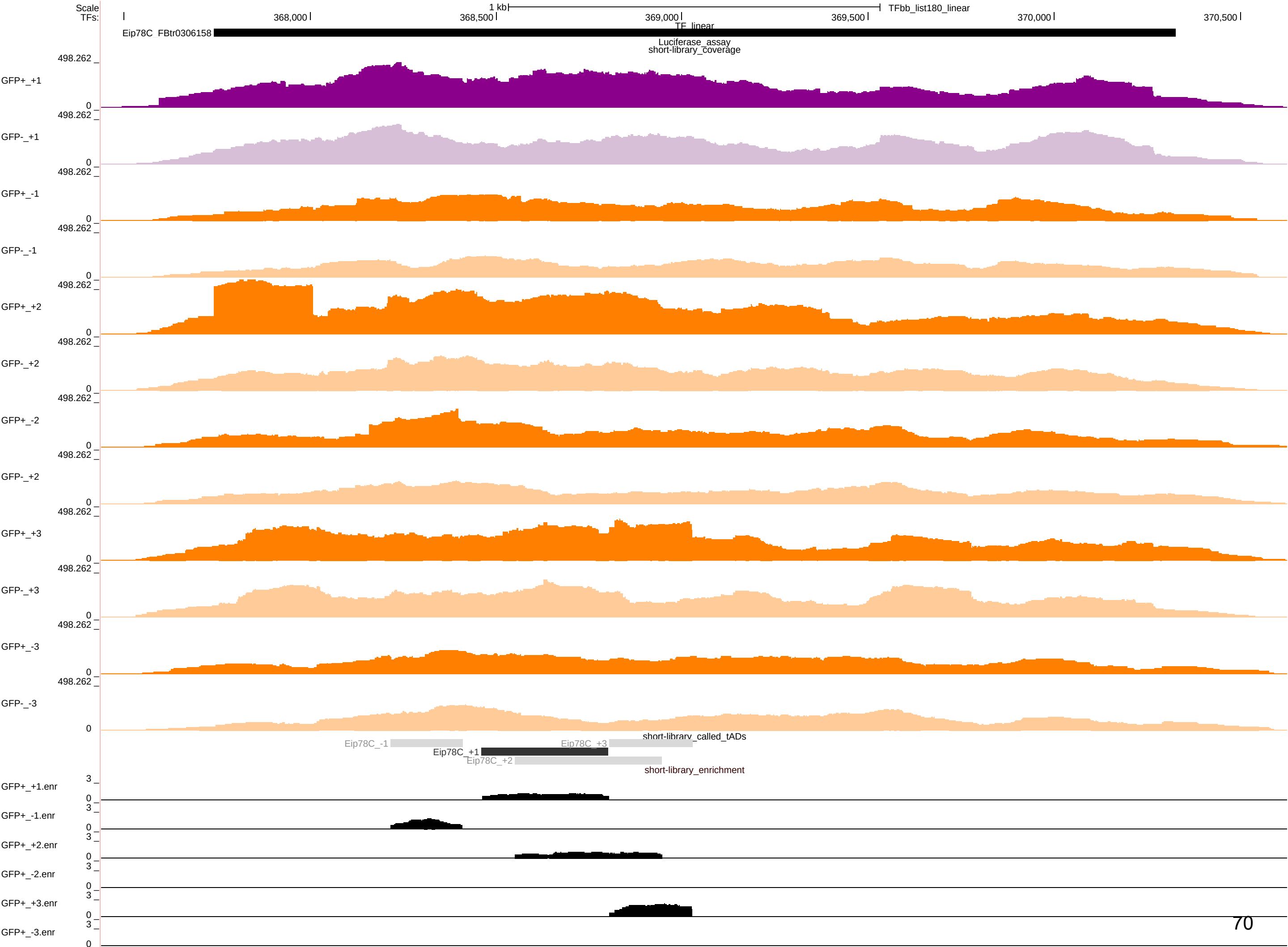

Scale
1 kb
TFbb_list180_linear
TFs:
368,000
368,500
369,000
369,500
370,000
370,500
Eip78C FBtr0306158
TF_linear
Luciferase_assay
short-library_coverage
GFP+_+1
GFP-_+1
GFP+_-1
GFP-_-1
GFP+_+2
GFP-_+2
GFP+_-2
GFP-_+2
GFP+_+3
GFP-_+3
GFP+_-3
GFP-_-3
498.262
0
short-library_called_tADs
Eip78C_-1
Eip78C_+3
Eip78C_+1
Eip78C_+2
short-library_enrichment
GFP+_+1.enr
GFP+_-1.enr
GFP+_+2.enr
GFP+_-2.enr
GFP+_+3.enr
GFP+_-3.enr
3
0

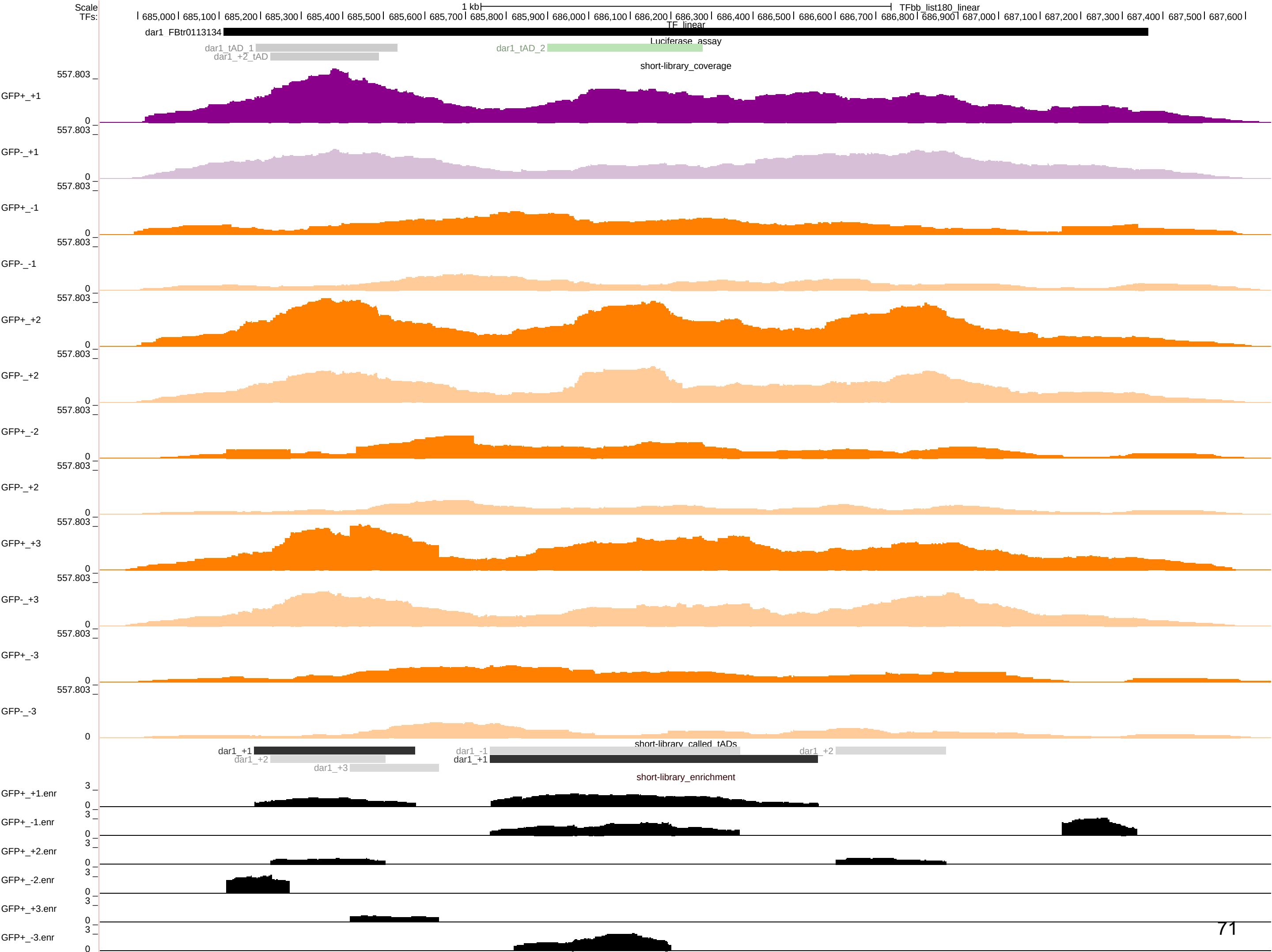

Scale
1 kb
TFbb_list180_linear
TFs:
685,000 685,100 685,200 685,300 685,400 685,500 685,600 685,700 685,800 685,900 686,000 686,100 686,200 686,300 686,400 686,500 686,600 686,700 686,800 686,900 687,000 687,100 687,200 687,300 687,400 687,500 687,600
TF_linear
dar1 FBtr0113134
Luciferase_assay
dar1_tAD_1
dar1_+2_tAD
dar1_tAD_2
short-library_coverage
GFP+_+1
GFP-_+1
GFP+_-1
GFP-_-1
GFP+_+2
GFP-_+2
GFP+_-2
GFP-_+2
GFP+_+3
GFP-_+3
GFP+_-3
GFP-_-3
557.803
0
short-library_called_tADs
dar1_+1
dar1_+2
dar1_+3
dar1_-1
dar1_+1
dar1_+2
short-library_enrichment
GFP+_+1.enr
GFP+_-1.enr
GFP+_+2.enr
GFP+_-2.enr
GFP+_+3.enr
GFP+_-3.enr
3
0

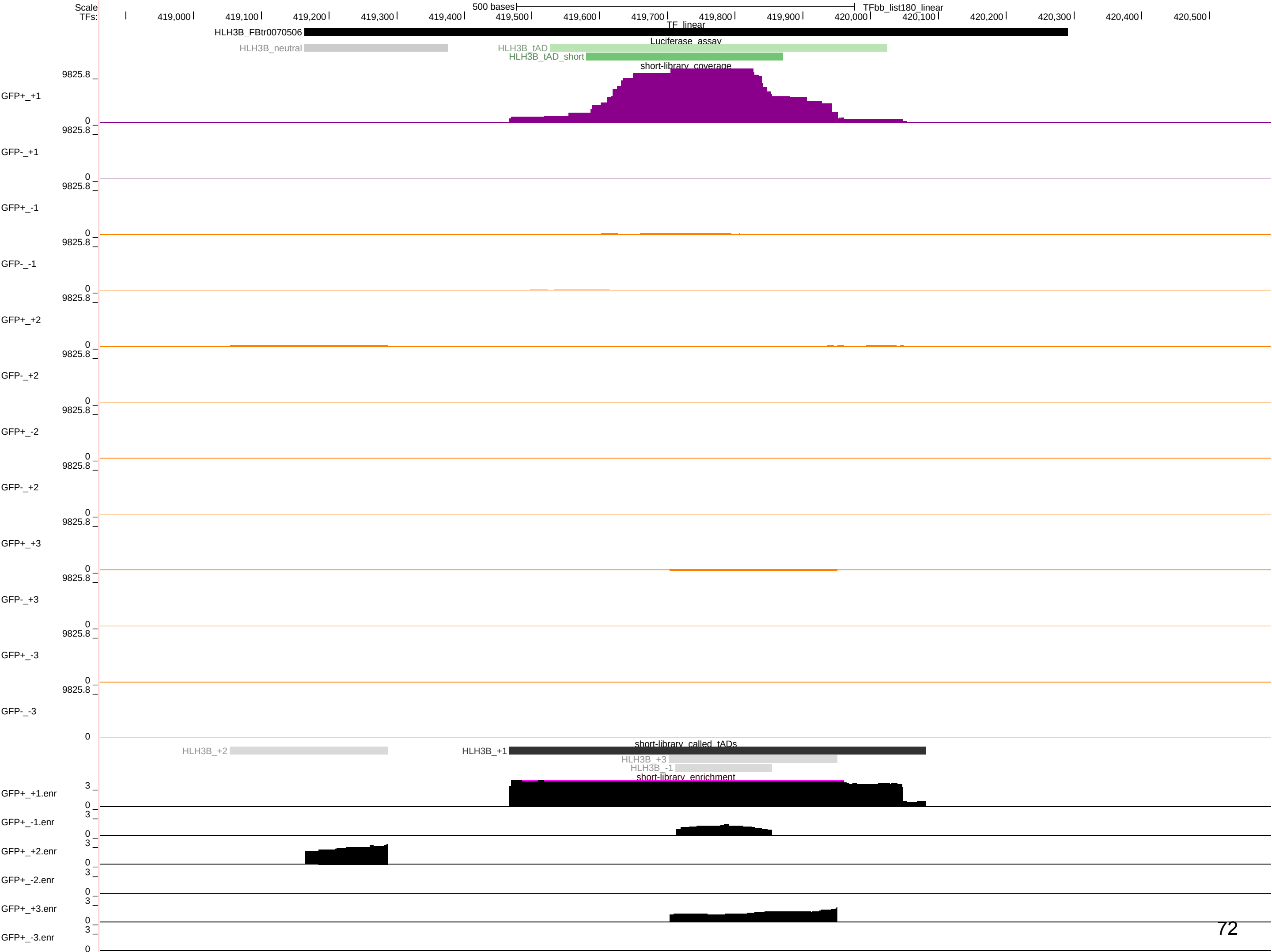

Scale
500 bases
TFs:
419,000
419,100
419,200
419,300
419,400
419,500
419,600
419,700
419,800
419,900
420,000
420,100
420,200
420,300
420,400
420,500
TFbb_list180_linear
TF_linear
HLH3B_FBtr0070506
Luciferase_assay
HLH3B_neutral
HLH3B_tAD
HLH3B_tAD_short
short-library_coverage
9825.8
0
GFP+_+1
GFP-_+1
GFP+_-1
GFP-_-1
GFP+_+2
GFP-_+2
GFP+_-2
GFP-_+2
GFP+_+3
GFP-_+3
GFP+_-3
GFP-_-3
short-library_called_tADs
HLH3B_+2
HLH3B_+1
HLH3B_+3
HLH3B_-1
short-library_enrichment
3
GFP+_+1.enr
GFP+_-1.enr
GFP+_+2.enr
GFP+_-2.enr
GFP+_+3.enr
GFP+_-3.enr

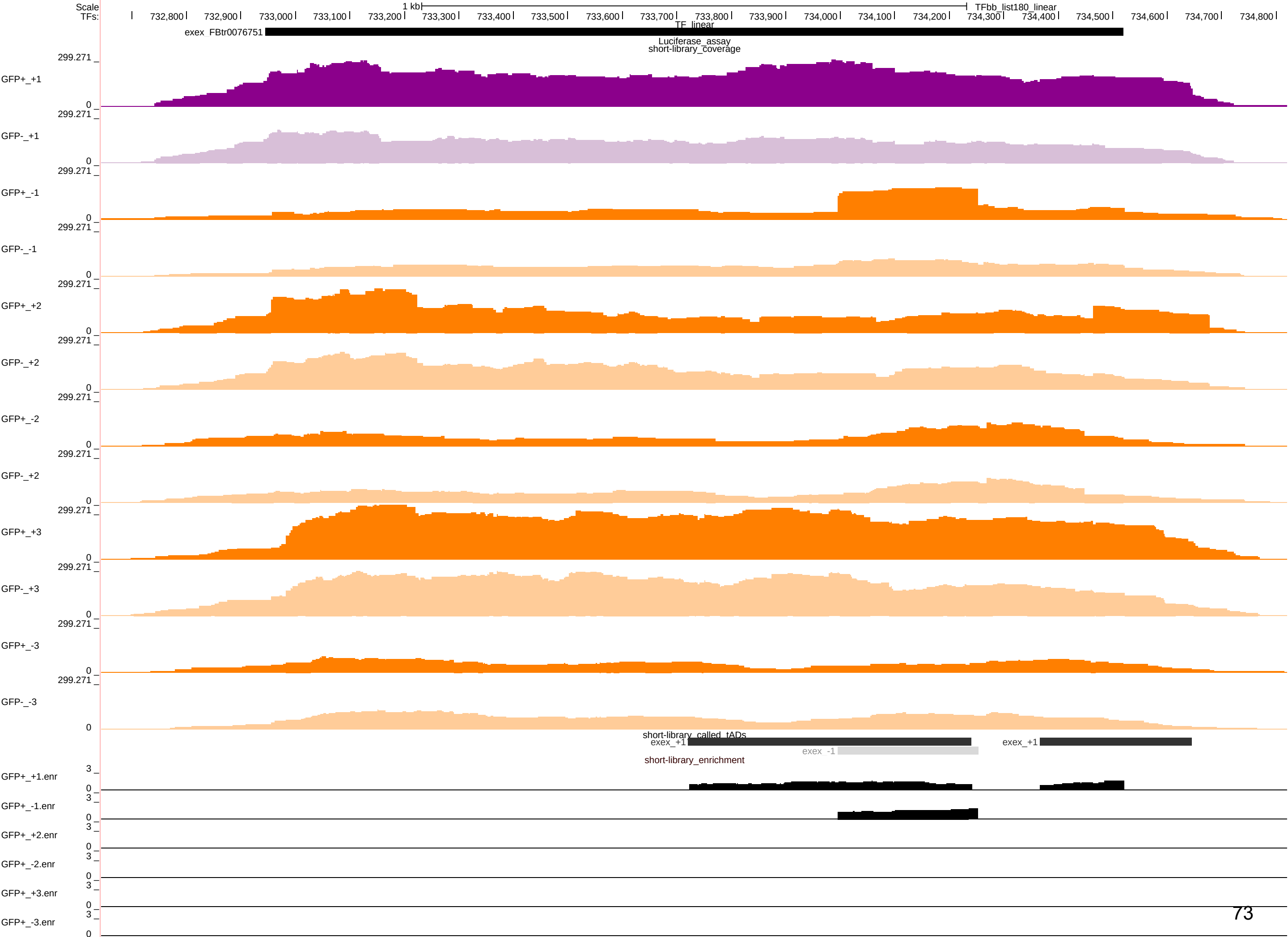

Scale
TFs:
1 kb
TFbb_list180_linear
732,800
732,900
733,000
733,100
733,200
733,300
733,400
733,500
733,600
733,700
733,800
733,900
734,000
734,100
734,200
734,300
734,400
734,500
734,600
734,700
734,800
TF_linear
exex_FBtr0076751
Luciferase_assay
short-library_coverage
GFP+_+1
GFP-_+1
GFP+_-1
GFP-_-1
GFP+_+2
GFP-_+2
GFP+_-2
GFP-_+2
GFP+_+3
GFP-_+3
GFP+_-3
GFP-_-3
299.271
0
short-library_called_tADs
exex_+1
exex_+1
exex_-1
short-library_enrichment
GFP+_+1.enr
GFP+_-1.enr
GFP+_+2.enr
GFP+_-2.enr
GFP+_+3.enr
GFP+_-3.enr
3
0

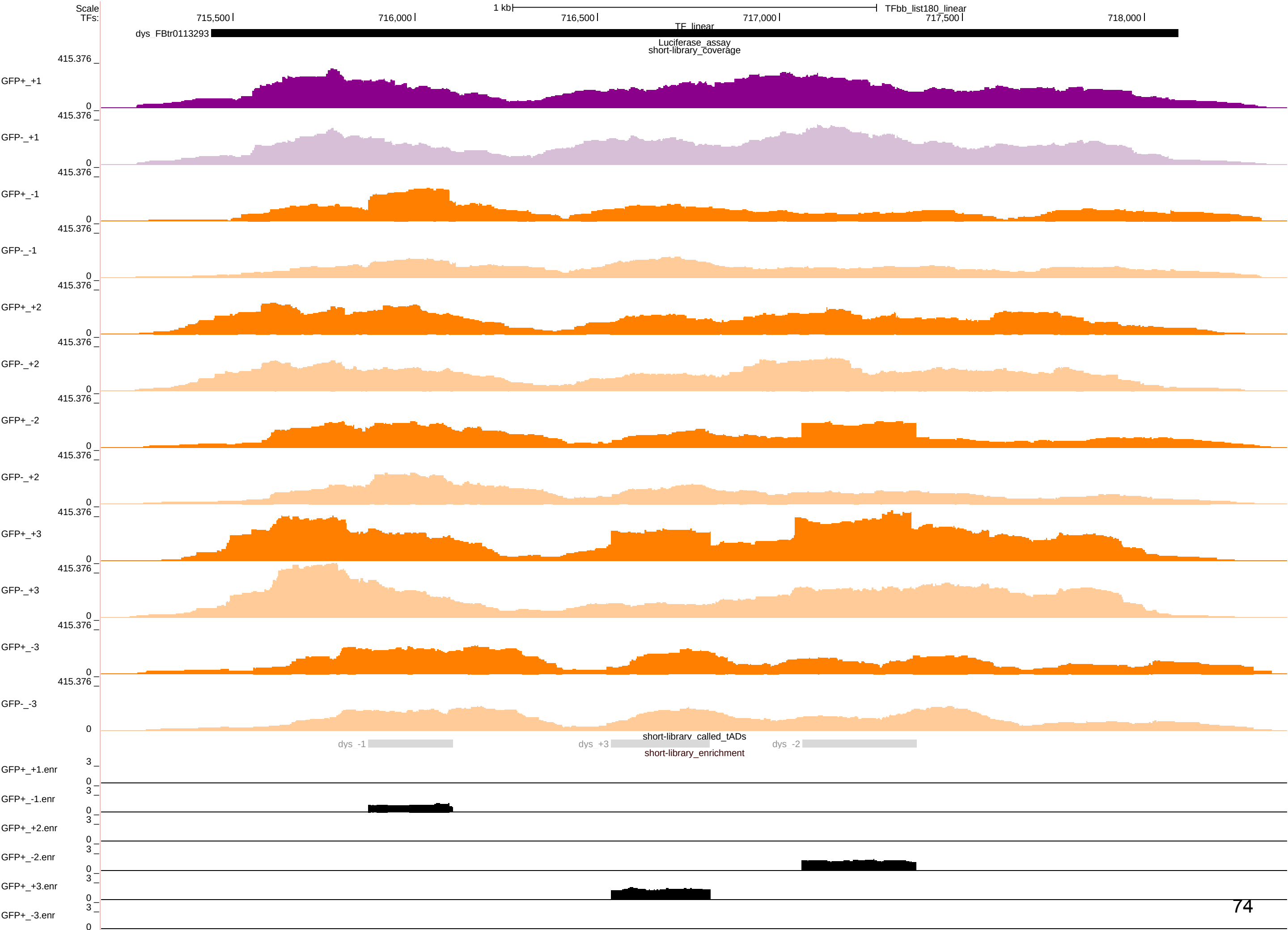

Scale
1 kb
TFs:
715,500
716,000
716,500
717,000
717,500
718,000
TFbb_list180_linear
TF_linear
dys_FBtr0113293
Luciferase_assay
short-library_coverage
GFP+_+1
GFP-_+1
GFP+_-1
GFP-_-1
GFP+_+2
GFP-_+2
GFP+_-2
GFP-_+2
GFP+_+3
GFP-_+3
GFP+_-3
GFP-_-3
415.376
0
short-library_called_tADs
dys_-1
dys_+3
dys_-2
short-library_enrichment
GFP+_+1.enr
GFP+_-1.enr
GFP+_+2.enr
GFP+_-2.enr
GFP+_+3.enr
GFP+_-3.enr
3
0

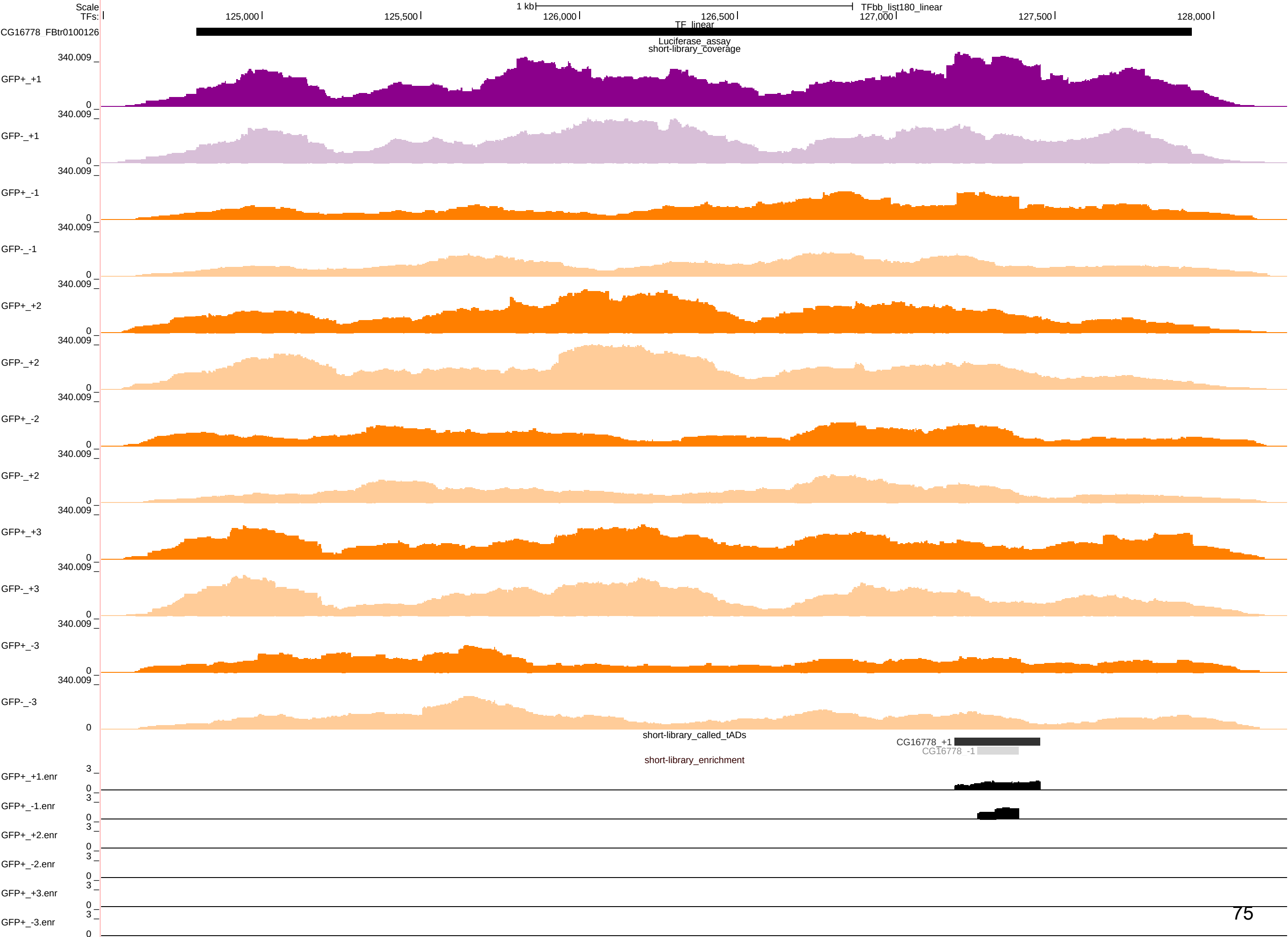

Scale
1 kb
TFbb_list180_linear
TFs:
125,000
125,500
126,000
126,500
127,000
127,500
128,000
TF_linear
CG16778 FBtr0100126
Luciferase_assay
short-library_coverage
340.009
GFP+_+1
GFP-_+1
GFP+_-1
GFP-_-1
GFP+_+2
GFP-_+2
GFP+_-2
GFP-_+2
GFP+_+3
GFP-_+3
GFP+_-3
GFP-_-3
0
short-library_called_tADs
CG16778_+1
CG16778_-1
short-library_enrichment
GFP+_+1.enr
GFP+_-1.enr
GFP+_+2.enr
GFP+_-2.enr
GFP+_+3.enr
GFP+_-3.enr
3
0

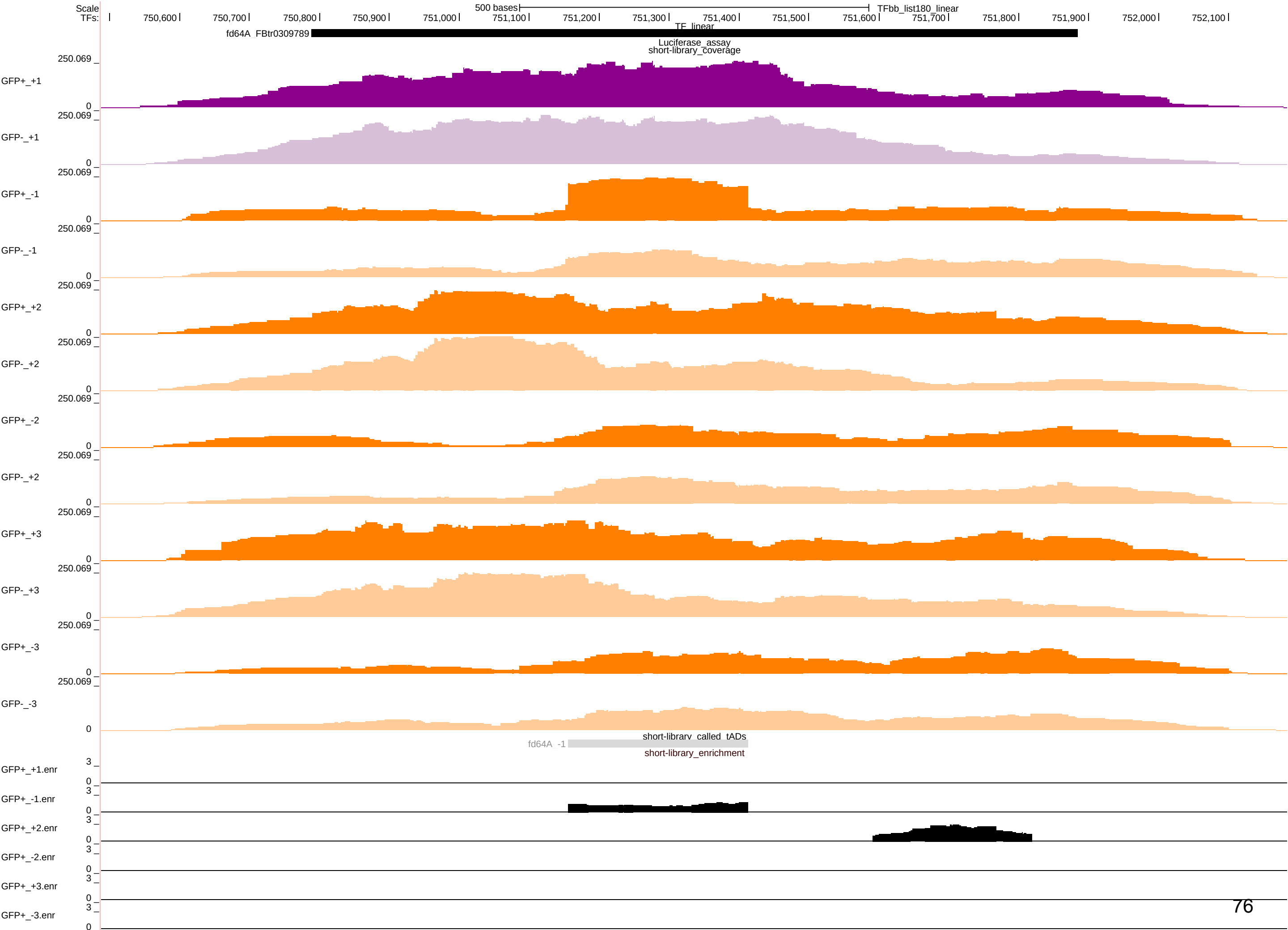

Scale
TFs:
500 bases
TFbb_list180_linear
750,600
750,700
750,800
750,900
751,000
751,100
751,200
751,300
751,400
751,500
751,600
751,700
751,800
751,900
752,000
752,100
TF_linear
fd64A_FBtr0309789
Luciferase_assay
short-library_coverage
GFP+_+1
GFP-_+1
GFP+_-1
GFP-_-1
GFP+_+2
GFP-_+2
GFP+_-2
GFP-_+2
GFP+_+3
GFP-_+3
GFP+_-3
GFP-_-3
250.069
0
short-library_called_tADs
fd64A_-1
short-library_enrichment
GFP+_+1.enr
GFP+_-1.enr
GFP+_+2.enr
GFP+_-2.enr
GFP+_+3.enr
GFP+_-3.enr
3
0

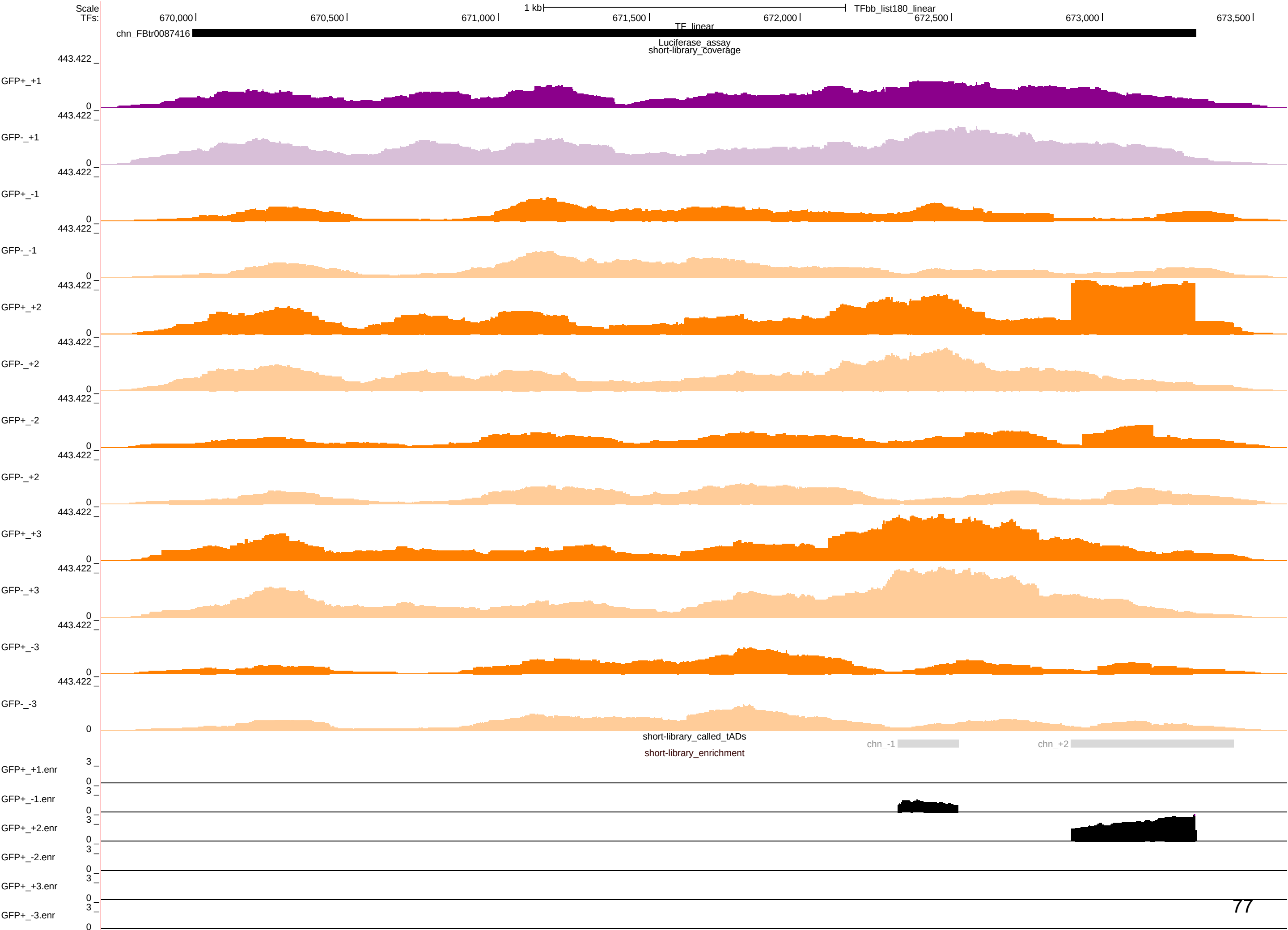
Scale
1 kb
TFbb_list180_linear
TFs:
670,000
670,500
671,000
671,500
672,000
672,500
673,000
673,500
TF_linear
chn FBtr0087416
Luciferase_assay
short-library_coverage
443.422
0
GFP+_+1
GFP-_+1
GFP+_-1
GFP-_-1
GFP+_+2
GFP-_+2
GFP+_-2
GFP-_+2
GFP+_+3
GFP-_+3
GFP+_-3
GFP-_-3
short-library_called_tADs
chn -1
chn +2
short-library_enrichment
3
GFP+_+1.enr
GFP+_-1.enr
GFP+_+2.enr
GFP+_-2.enr
GFP+_+3.enr
GFP+_-3.enr

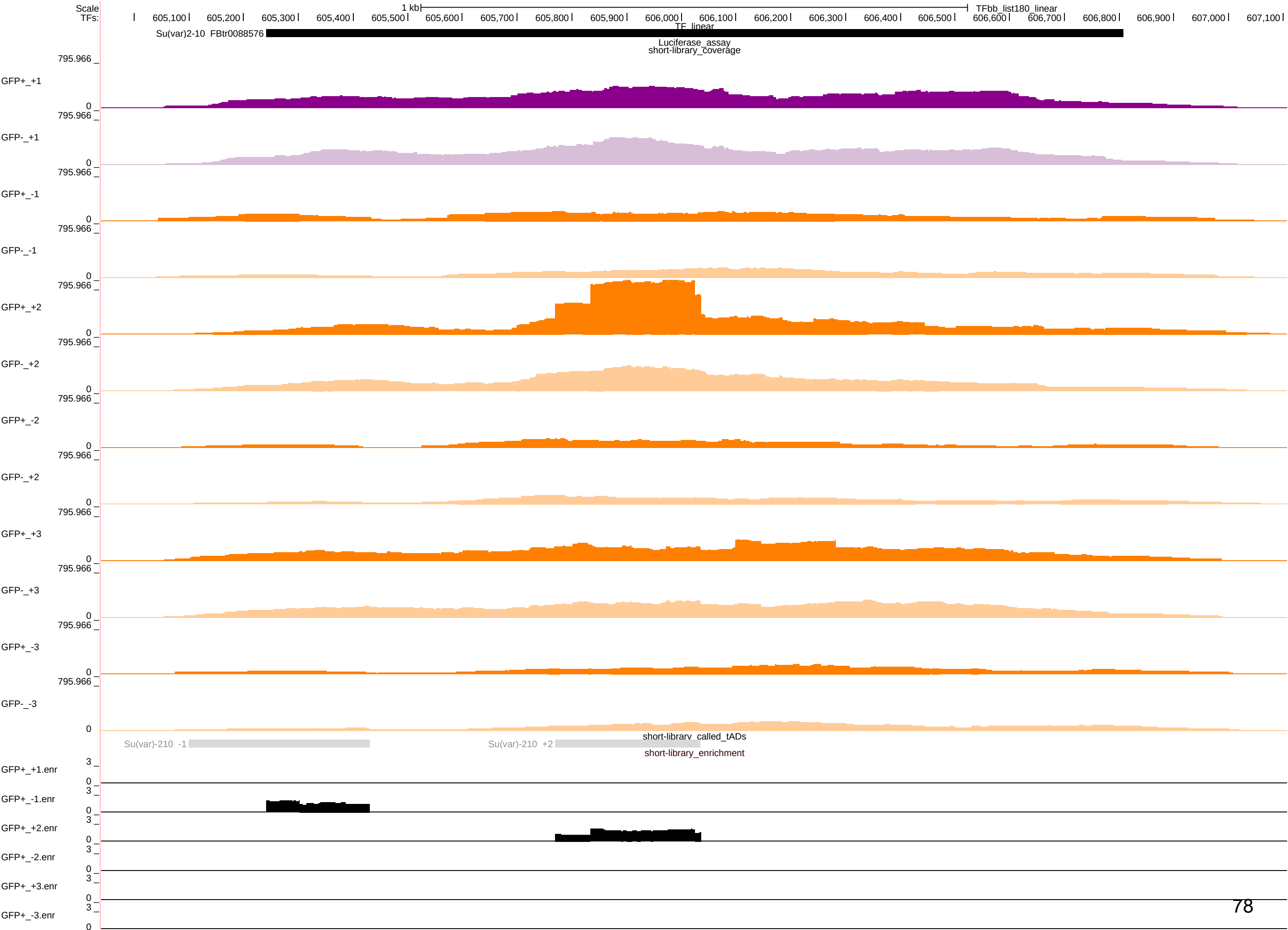

Scale
TFs:
1 kb
TFbb_list180_linear
605,100
605,200
605,300
605,400
605,500
605,600
605,700
605,800
605,900
606,000
606,100
606,200
606,300
606,400
606,500
606,600
606,700
606,800
606,900
607,000
607,100
TF_linear
Su(var)2-10 FBtr0088576
Luciferase_assay
short-library_coverage
795.966
GFP+_+1
0
GFP-_+1
GFP+_-1
GFP-_-1
GFP+_+2
GFP-_+2
GFP+_-2
GFP-_+2
GFP+_+3
GFP-_+3
GFP+_-3
GFP-_-3
Su(var)-210 -1
Su(var)-210 +2
short-library_called_tADs
short-library_enrichment
3
GFP+_+1.enr
GFP+_-1.enr
GFP+_+2.enr
GFP+_-2.enr
GFP+_+3.enr
GFP+_-3.enr

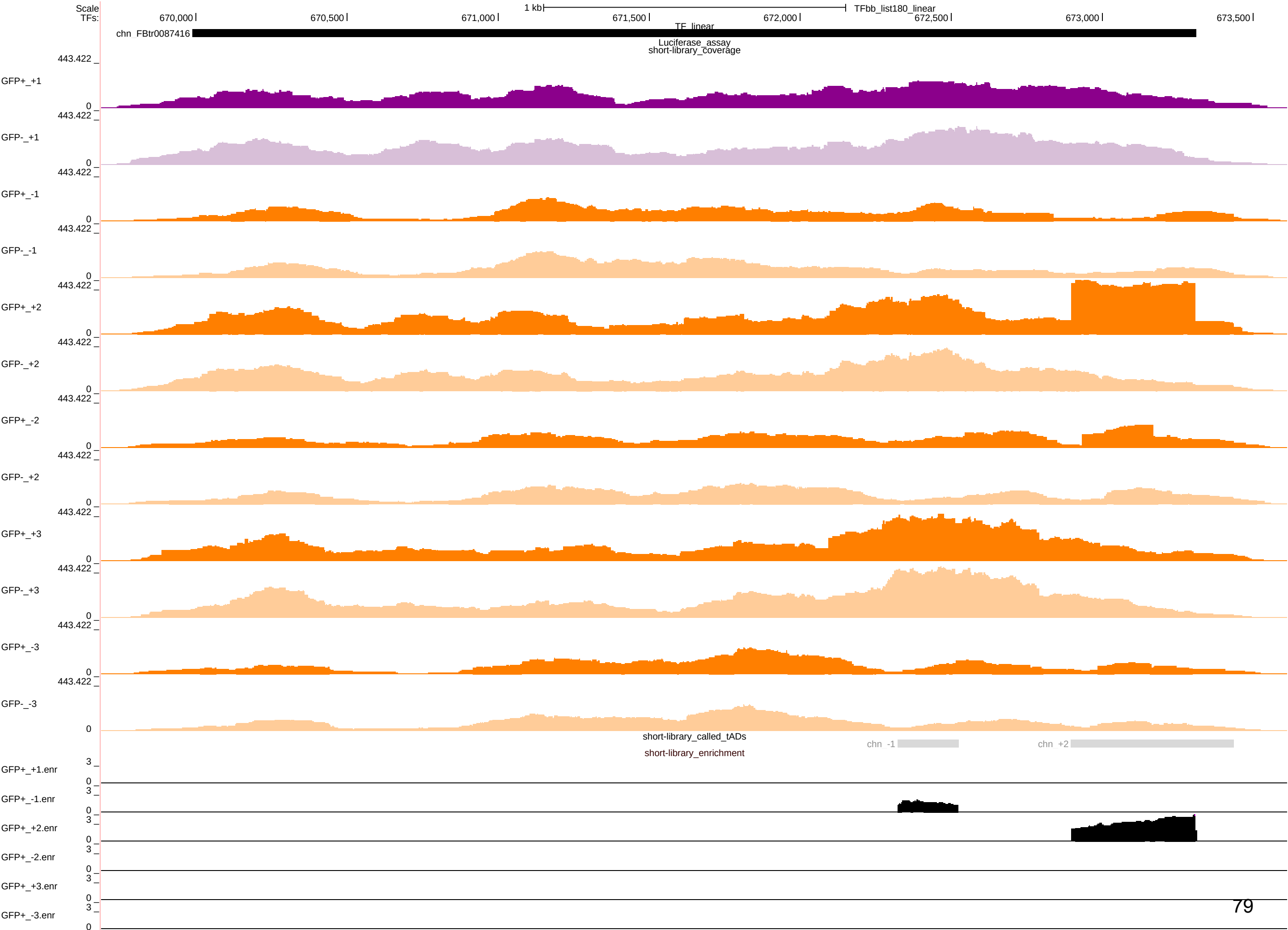

Scale
TFs:
1 kb
TFbb_list180_linear
670,000
670,500
671,000
671,500
672,000
672,500
673,000
673,500
TF_linear
chn FBtr0087416
Luciferase_assay
short-library_coverage
GFP+_+1
GFP-_+1
GFP+_-1
GFP-_-1
GFP+_+2
GFP-_+2
GFP+_-2
GFP-_-2
GFP+_+3
GFP-_+3
GFP+_-3
GFP-_-3
443.422
0
short-library_called_tADs
chn -1
chn +2
short-library_enrichment
GFP+_+1.enr
GFP+_-1.enr
GFP+_+2.enr
GFP+_-2.enr
GFP+_+3.enr
GFP+_-3.enr
3
0

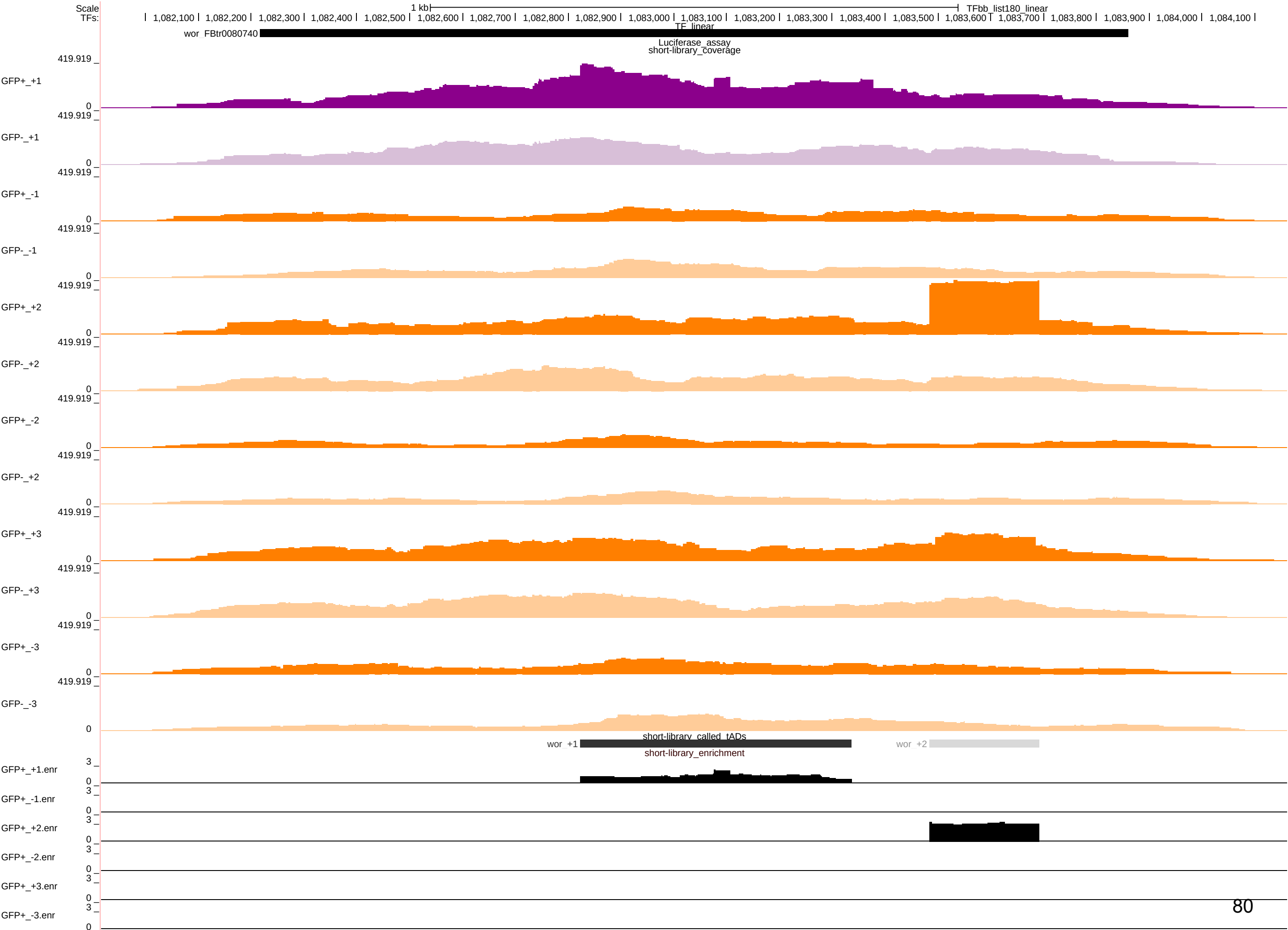

Scale
TFs:
1 kb
TFbb_list180_linear
1,082,100
1,082,200
1,082,300
1,082,400
1,082,500
1,082,600
1,082,700
1,082,800
1,082,900
1,083,000
1,083,100
1,083,200
1,083,300
1,083,400
1,083,500
1,083,600
1,083,700
1,083,800
1,083,900
1,084,000
1,084,100
TF_linear
wor FBtr0080740
Luciferase_assay
short-library_coverage
419.919 _
0 _
GFP+_+1
GFP-_+1
GFP+_-1
GFP-_-1
GFP+_+2
GFP-_+2
GFP+_-2
GFP-_+2
GFP+_+3
GFP-_+3
GFP+_-3
GFP-_-3
short-library_called_tADs
wor_+1
wor_+2
short-library_enrichment
3 _
GFP+_+1.enr
GFP+_-1.enr
GFP+_+2.enr
GFP+_-2.enr
GFP+_+3.enr
GFP+_-3.enr

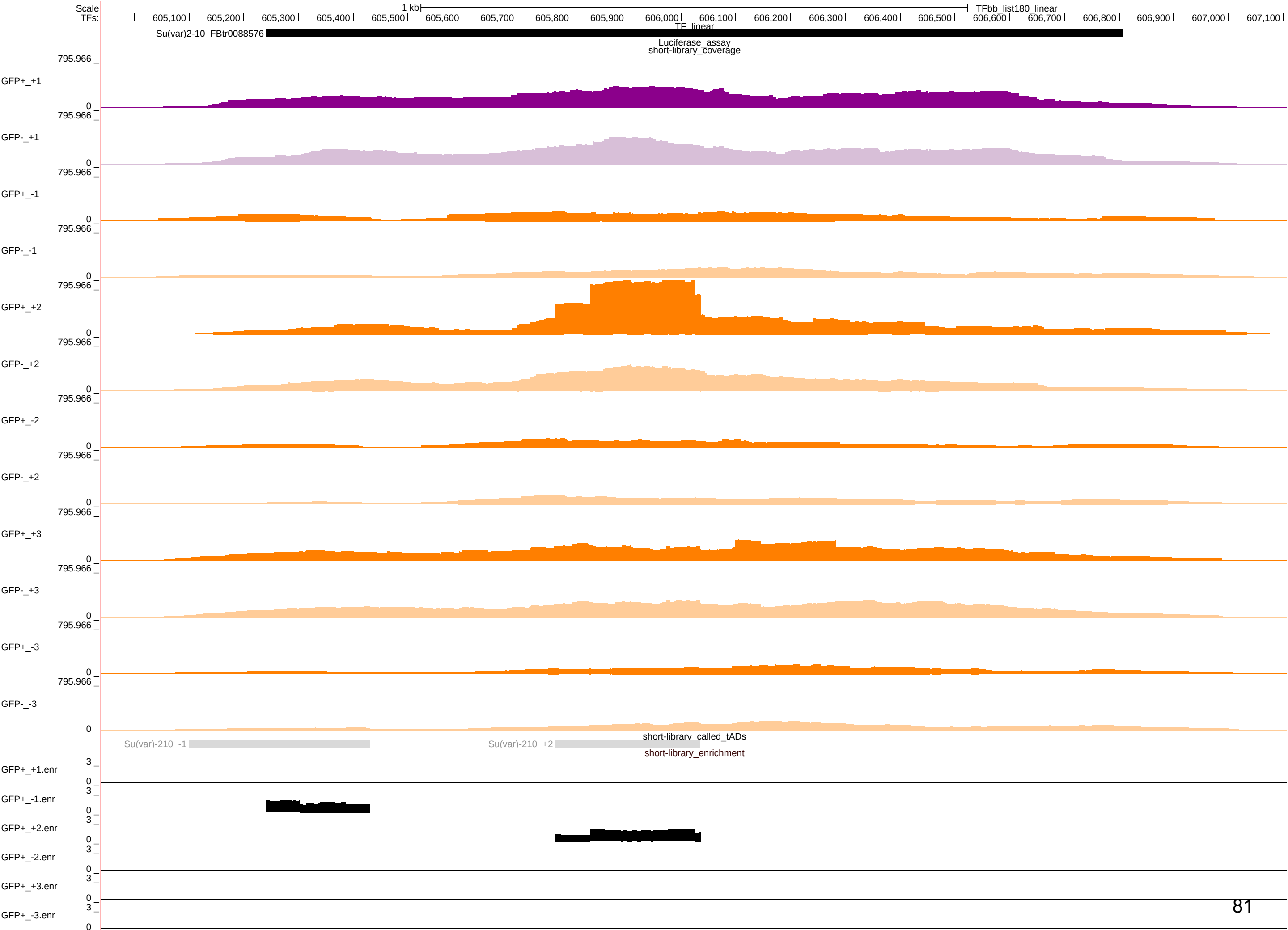

Scale
TFs:
1 kb
TFbb_list180_linear
605,100
605,200
605,300
605,400
605,500
605,600
605,700
605,800
605,900
606,000
606,100
606,200
606,300
606,400
606,500
606,600
606,700
606,800
606,900
607,000
607,100
TF_linear
Su(var)2-10 FBtr0088576
Luciferase_assay
short-library_coverage
795.966
GFP+_+1
0
GFP-_+1
GFP+_-1
GFP-_-1
GFP+_+2
GFP-_+2
GFP+_-2
GFP-_+2
GFP+_+3
GFP-_+3
GFP+_-3
GFP-_-3
Su(var)-210 -1
Su(var)-210 +2
short-library_called_tADs
short-library_enrichment
3
GFP+_+1.enr
GFP+_-1.enr
GFP+_+2.enr
GFP+_-2.enr
GFP+_+3.enr
GFP+_-3.enr

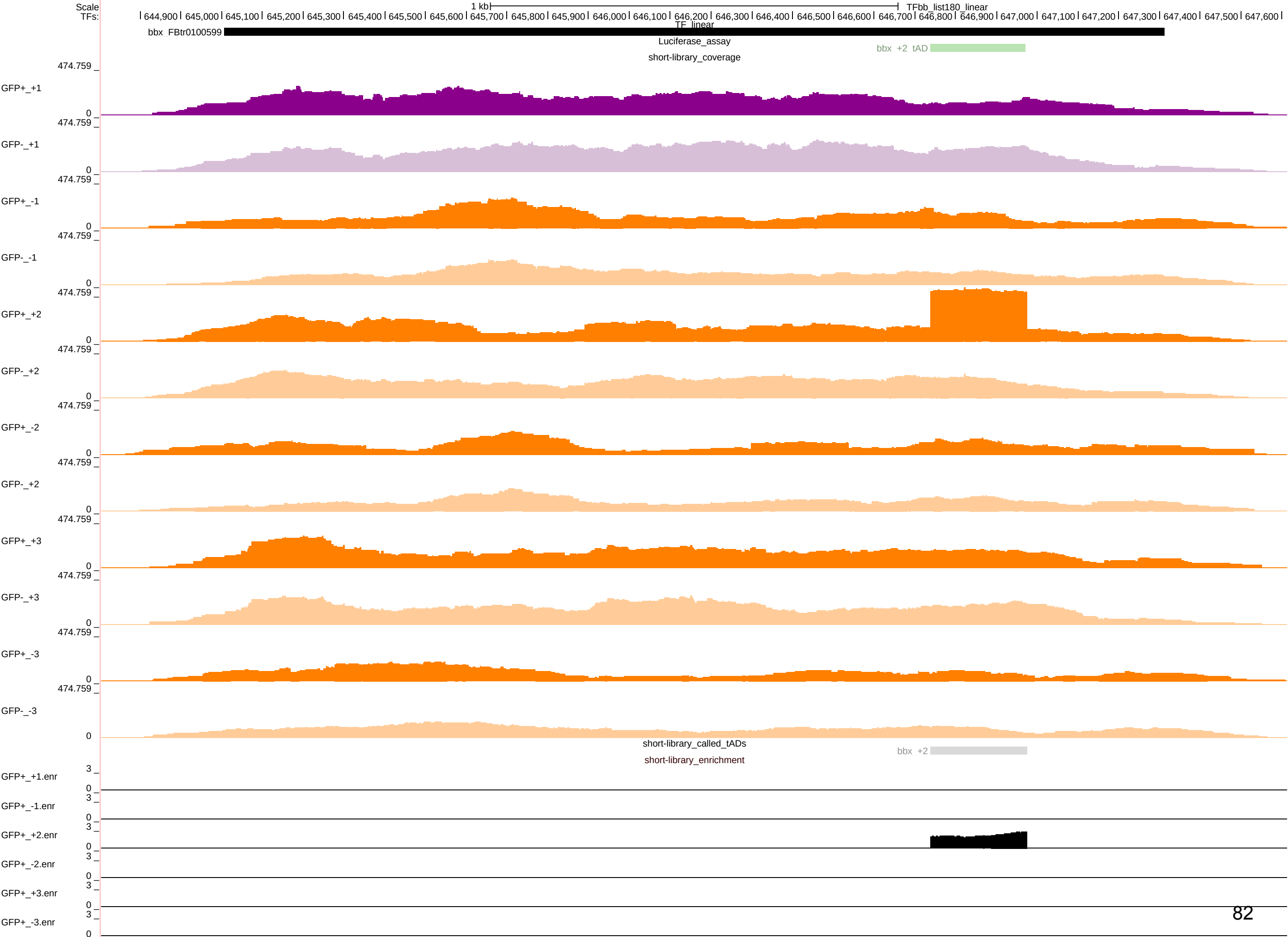

Scale
TFs:
1 kb
TFbb_list180_linear
644,900 645,000 645,100 645,200 645,300 645,400 645,500 645,600 645,700 645,800 645,900 646,000 646,100 646,200 646,300 646,400 646,500 646,600 646,700 646,800 646,900 647,000 647,100 647,200 647,300 647,400 647,500 647,600
TF_linear
bbx FBtr0100599
Luciferase_assay
bbx +2 tAD
short-library_coverage
GFP+_+1
GFP-_+1
GFP+_-1
GFP-_-1
GFP+_+2
GFP-_+2
GFP+_-2
GFP-_+2
GFP+_+3
GFP-_+3
GFP+_-3
GFP-_-3
474.759
0
short-library_called_tADs
bbx +2
short-library_enrichment
GFP+_+1.enr
GFP+_-1.enr
GFP+_+2.enr
GFP+_-2.enr
GFP+_+3.enr
GFP+_-3.enr
3
0

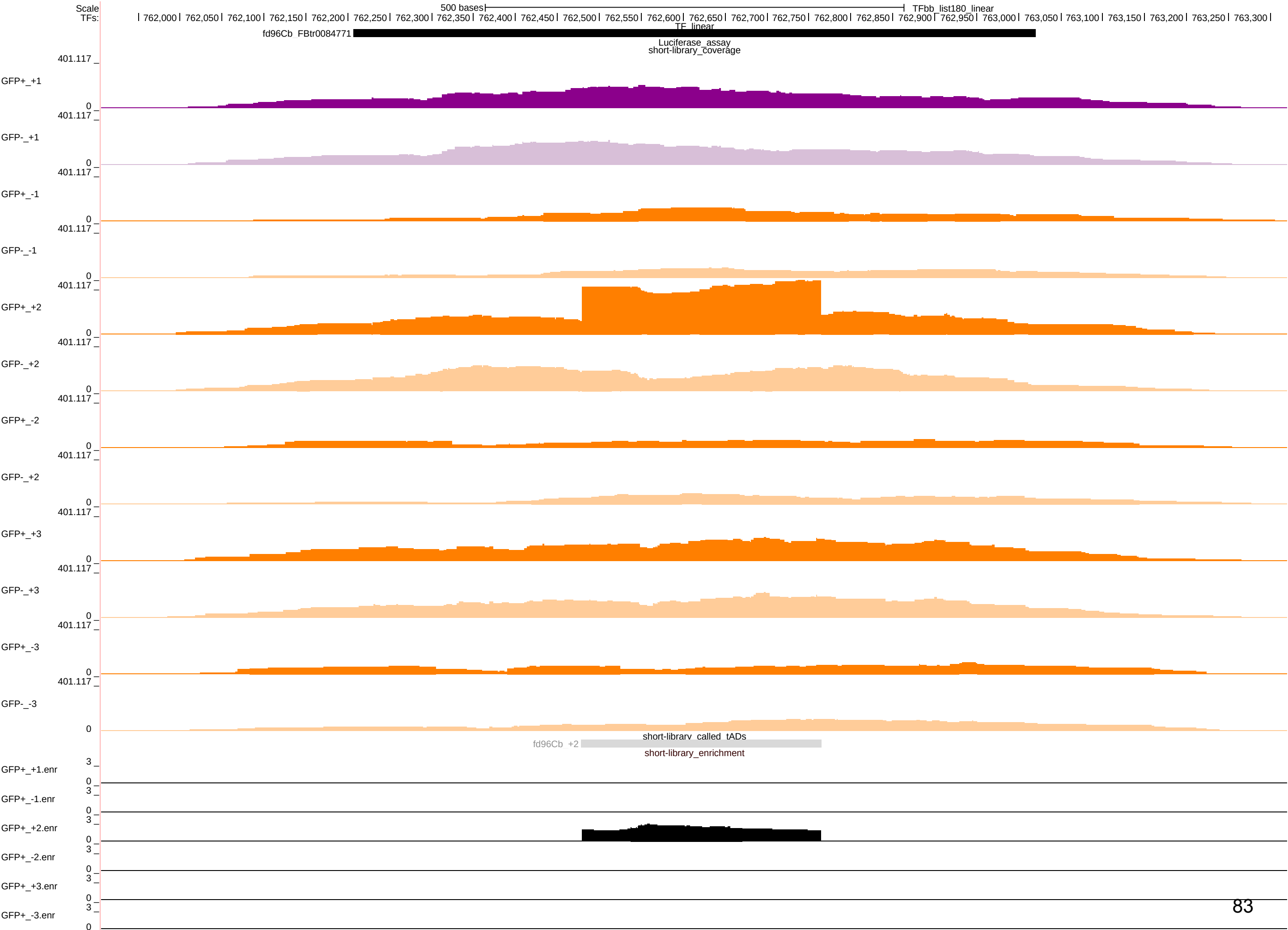

Scale
TFs:
500 bases
TFbb_list180_linear
762,000 762,050 762,100 762,150 762,200 762,250 762,300 762,350 762,400 762,450 762,500 762,550 762,600 762,650 762,700 762,750 762,800 762,850 762,900 762,950 763,000 763,050 763,100 763,150 763,200 763,250 763,300
TF_linear
fd96Cb_FBtr0084771
Luciferase_assay
short-library_coverage
401.117
0
GFP+_+1
GFP-_+1
GFP+_-1
GFP-_-1
GFP+_+2
GFP-_+2
GFP+_-2
GFP-_+2
GFP+_+3
GFP-_+3
GFP+_-3
GFP-_-3
short-library_called_tADs
fd96Cb_+2
short-library_enrichment
3
GFP+_+1.enr
GFP+_-1.enr
GFP+_+2.enr
GFP+_-2.enr
GFP+_+3.enr
GFP+_-3.enr

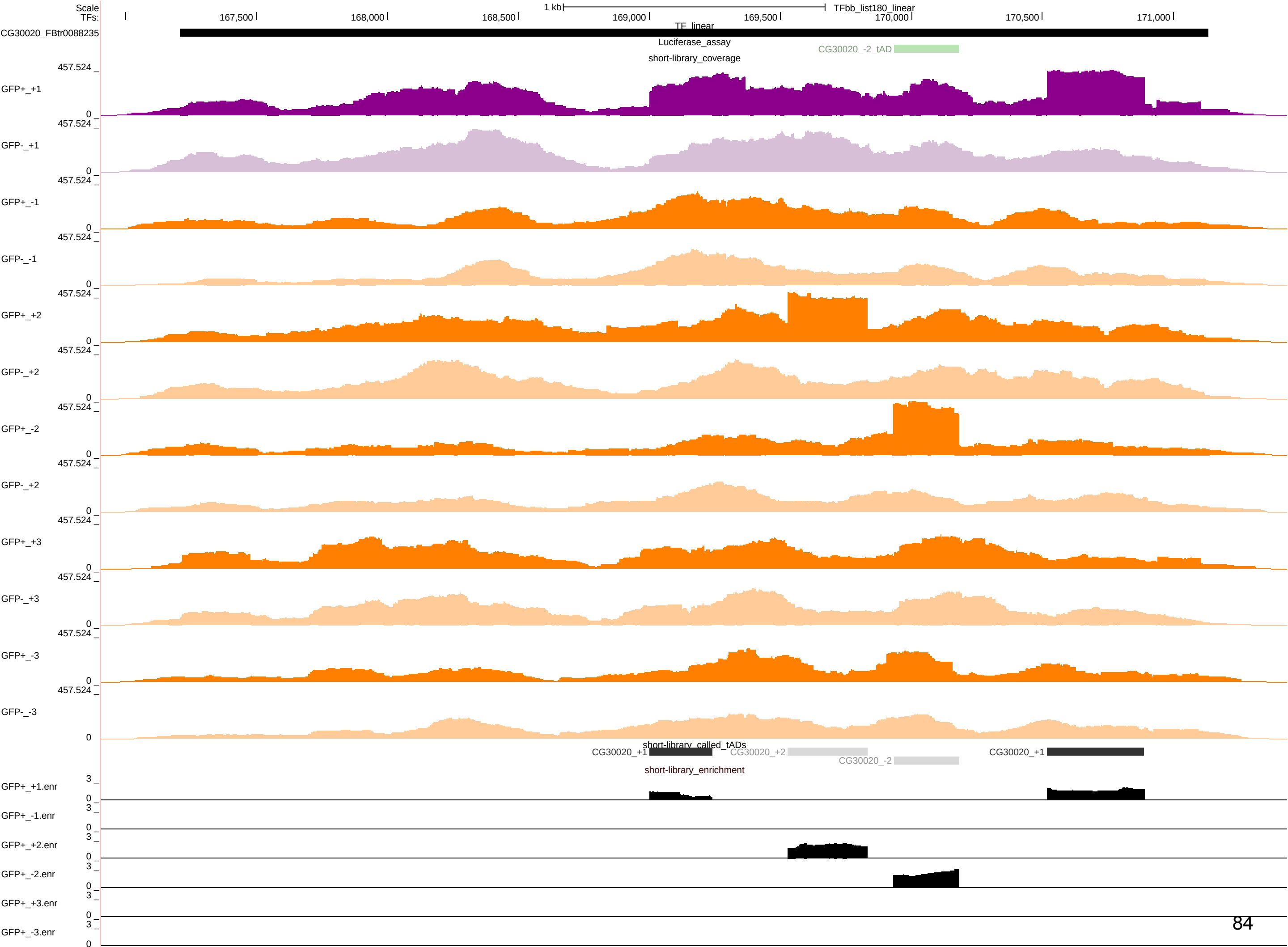
Scale
TFs:
1 kb
TFbb_list180_linear
167,500
168,000
168,500
169,000
169,500
170,000
170,500
171,000
CG30020 FBtr0088235
TF_linear
Luciferase_assay
CG30020 -2 tAD
short-library_coverage
457.524
0
GFP+_+1
GFP-_+1
GFP+_-1
GFP-_-1
GFP+_+2
GFP-_+2
GFP+_-2
GFP-_+2
GFP+_+3
GFP-_+3
GFP+_-3
GFP-_-3
short-library_called_tADs
CG30020_+1
CG30020_+2
CG30020_-2
CG30020_+1
short-library_enrichment
3
GFP+_+1.enr
GFP+_-1.enr
GFP+_+2.enr
GFP+_-2.enr
GFP+_+3.enr
GFP+_-3.enr

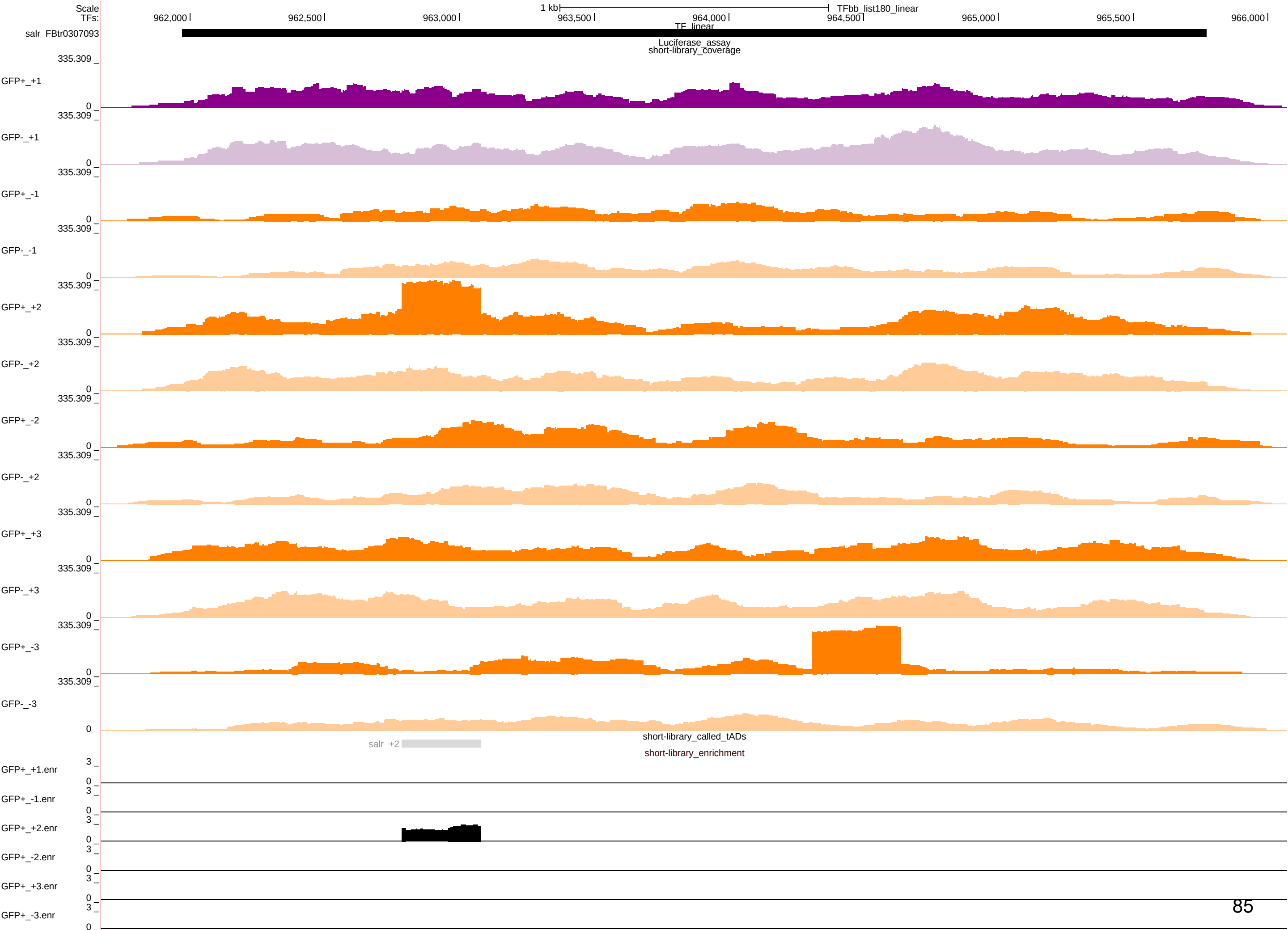
Scale
TFs:
1 kb
TFbb_list180_linear
962,000
962,500
963,000
963,500
964,000
964,500
965,000
965,500
966,000
TF_linear
salr_FBtr0307093
Luciferase_assay
short-library_coverage
335.309
GFP+_+1
0
GFP-_+1
GFP+_-1
GFP-_-1
GFP+_+2
GFP-_+2
GFP+_-2
GFP-_+2
GFP+_+3
GFP-_+3
GFP+_-3
GFP-_-3
short-library_called_tADs
salr_+2
short-library_enrichment
3
GFP+_+1.enr
GFP+_-1.enr
GFP+_+2.enr
GFP+_-2.enr
GFP+_+3.enr
GFP+_-3.enr

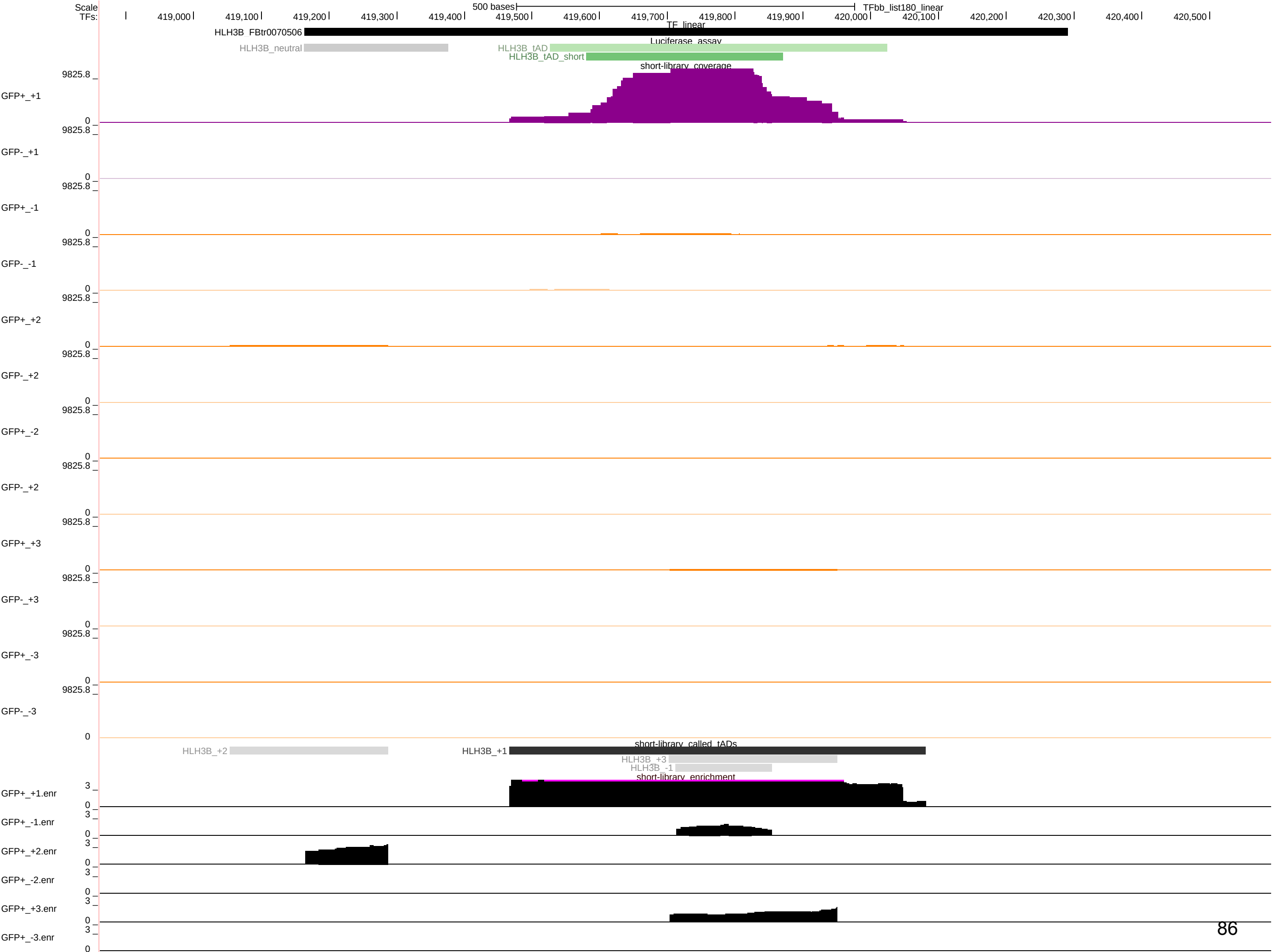

Scale
500 bases
TFs:
419,000
419,100
419,200
419,300
419,400
419,500
419,600
419,700
419,800
419,900
420,000
420,100
420,200
420,300
420,400
420,500
TFbb_list180_linear
TF_linear
HLH3B_FBtr0070506
Luciferase_assay
HLH3B_neutral
HLH3B_tAD
HLH3B_tAD_short
short-library_coverage
GFP+_+1
GFP-_+1
GFP+_-1
GFP-_-1
GFP+_+2
GFP-_+2
GFP+_-2
GFP-_+2
GFP+_+3
GFP-_+3
GFP+_-3
GFP-_-3
9825.8
0
short-library_called_tADs
HLH3B_+2
HLH3B_+1
HLH3B_+3
HLH3B_-1
short-library_enrichment
GFP+_+1.enr
GFP+_-1.enr
GFP+_+2.enr
GFP+_-2.enr
GFP+_+3.enr
GFP+_-3.enr
3
0

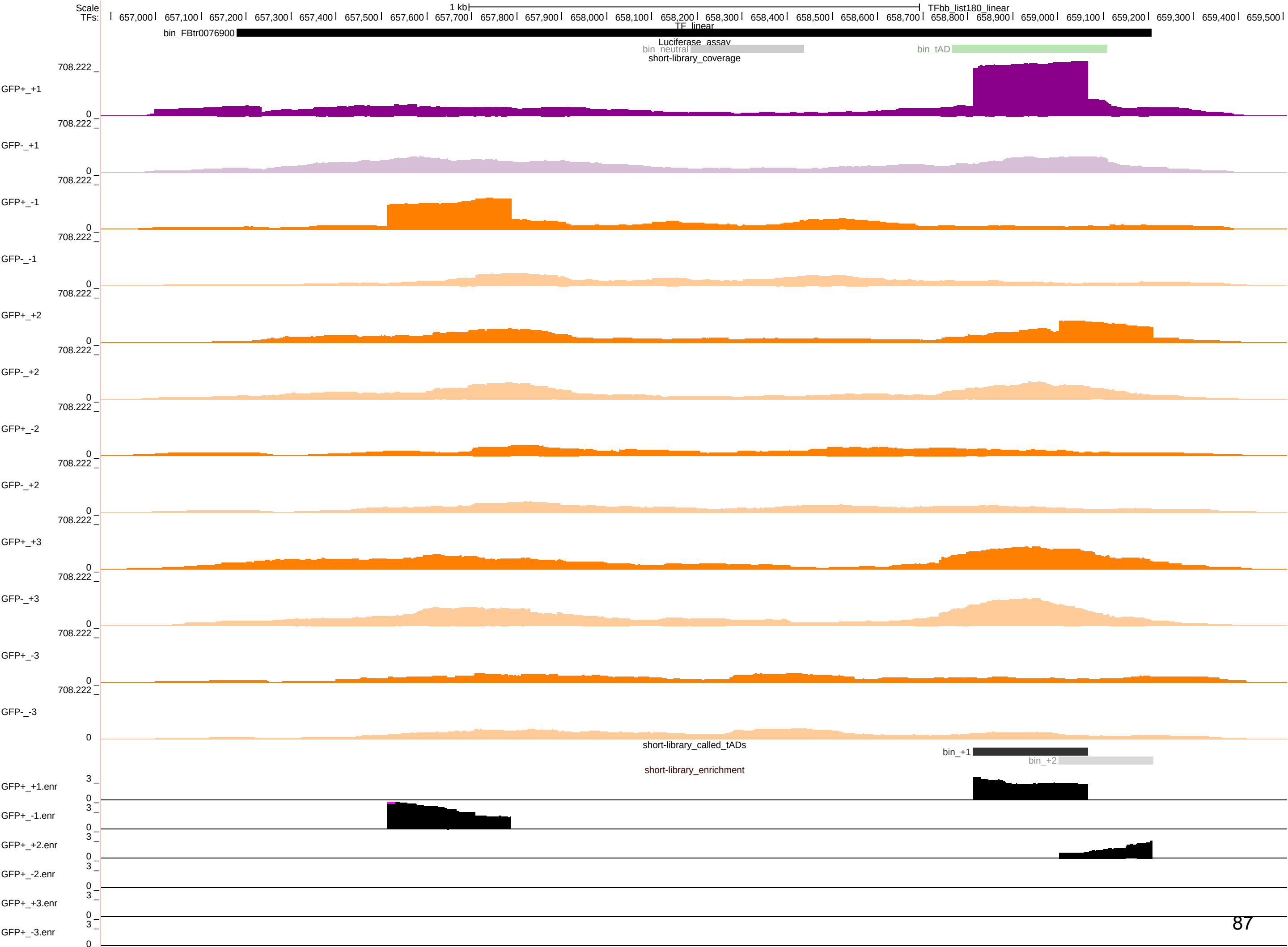

Scale
TFs:
1 kb
TFbb_list180_linear
657,000
657,100
657,200
657,300
657,400
657,500
657,600
657,700
657,800
657,900
658,000
658,100
658,200
658,300
658,400
658,500
658,600
658,700
658,800
658,900
659,000
659,100
659,200
659,300
659,400
659,500
TF_linear
bin_FBtr0076900
Luciferase_assay
bin_neutral
bin_tAD
short-library_coverage
GFP+_+1
GFP-_+1
GFP+_-1
GFP-_-1
GFP+_+2
GFP-_+2
GFP+_-2
GFP-_+2
GFP+_+3
GFP-_+3
GFP+_-3
GFP-_-3
708.222
0
short-library_called_tADs
bin_+1
bin_+2
short-library_enrichment
GFP+_+1.enr
GFP+_-1.enr
GFP+_+2.enr
GFP+_-2.enr
GFP+_+3.enr
GFP+_-3.enr
3
0

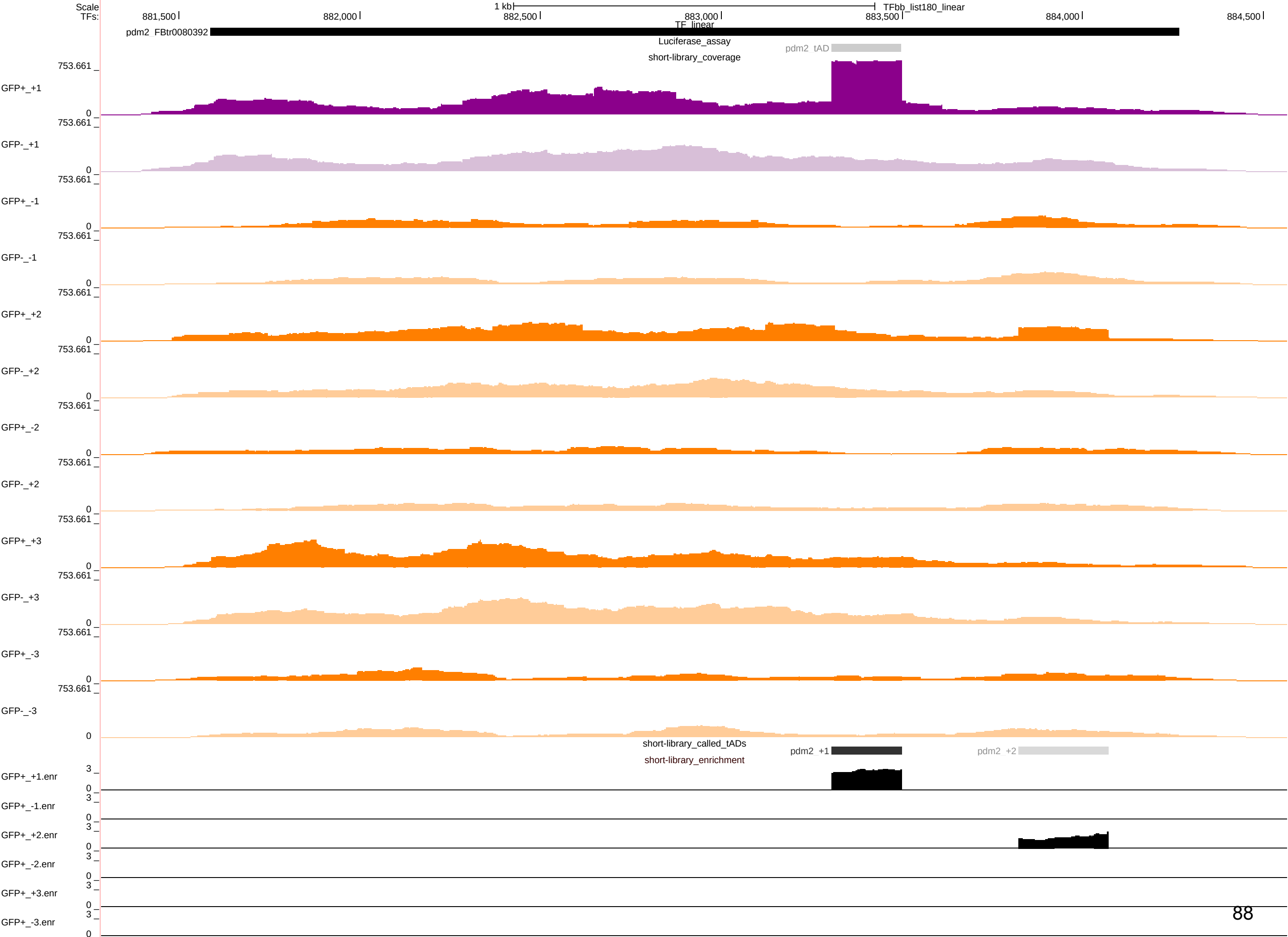
Scale
TFs:
1 kb
TFbb_list180_linear
881,500
882,000
882,500
883,000
883,500
884,000
884,500
pdm2 FBtr0080392
TF_linear
Luciferase_assay
pdm2 tAD
short-library_coverage
753.661
GFP+_+1
0
GFP-_+1
GFP+_-1
GFP-_-1
GFP+_+2
GFP-_+2
GFP+_-2
GFP-_+2
GFP+_+3
GFP-_+3
GFP+_-3
GFP-_-3
short-library_called_tADs
pdm2_+1
pdm2_+2
short-library_enrichment
3
GFP+_+1.enr
GFP+_-1.enr
GFP+_+2.enr
GFP+_-2.enr
GFP+_+3.enr
GFP+_-3.enr

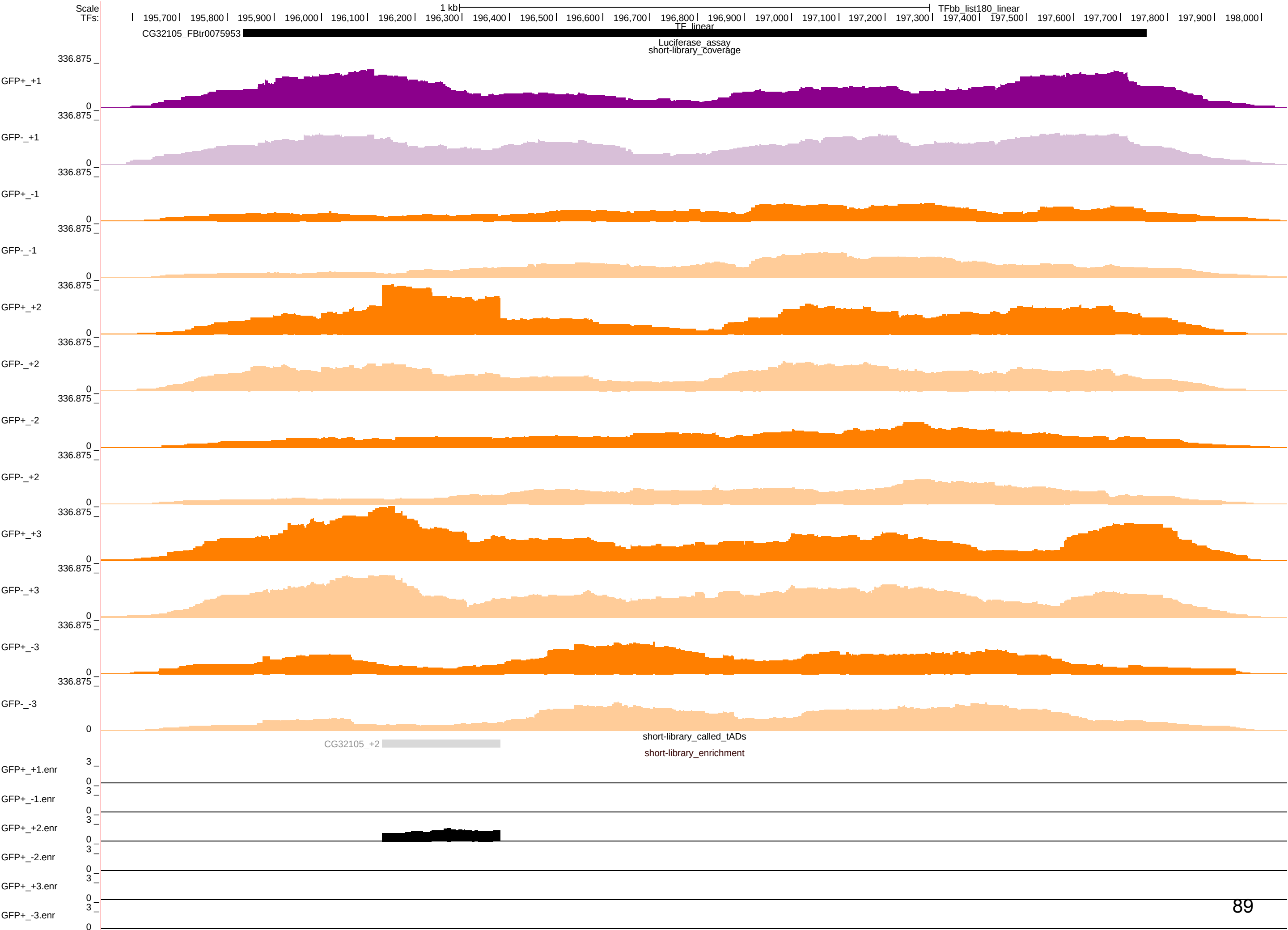

Scale
TFs:
1 kb
TFbb_list180_linear
195,700
195,800
195,900
196,000
196,100
196,200
196,300
196,400
196,500
196,600
196,700
196,800
196,900
197,000
197,100
197,200
197,300
197,400
197,500
197,600
197,700
197,800
197,900
198,000
TF_linear
CG32105 FBtr0075953
Luciferase_assay
short-library_coverage
336.875
0
GFP+_+1
GFP-_+1
GFP+_-1
GFP-_-1
GFP+_+2
GFP-_+2
GFP+_-2
GFP-_+2
GFP+_+3
GFP-_+3
GFP+_-3
GFP-_-3
short-library_called_tADs
CG32105 +2
short-library_enrichment
3
GFP+_+1.enr
GFP+_-1.enr
GFP+_+2.enr
GFP+_-2.enr
GFP+_+3.enr
GFP+_-3.enr

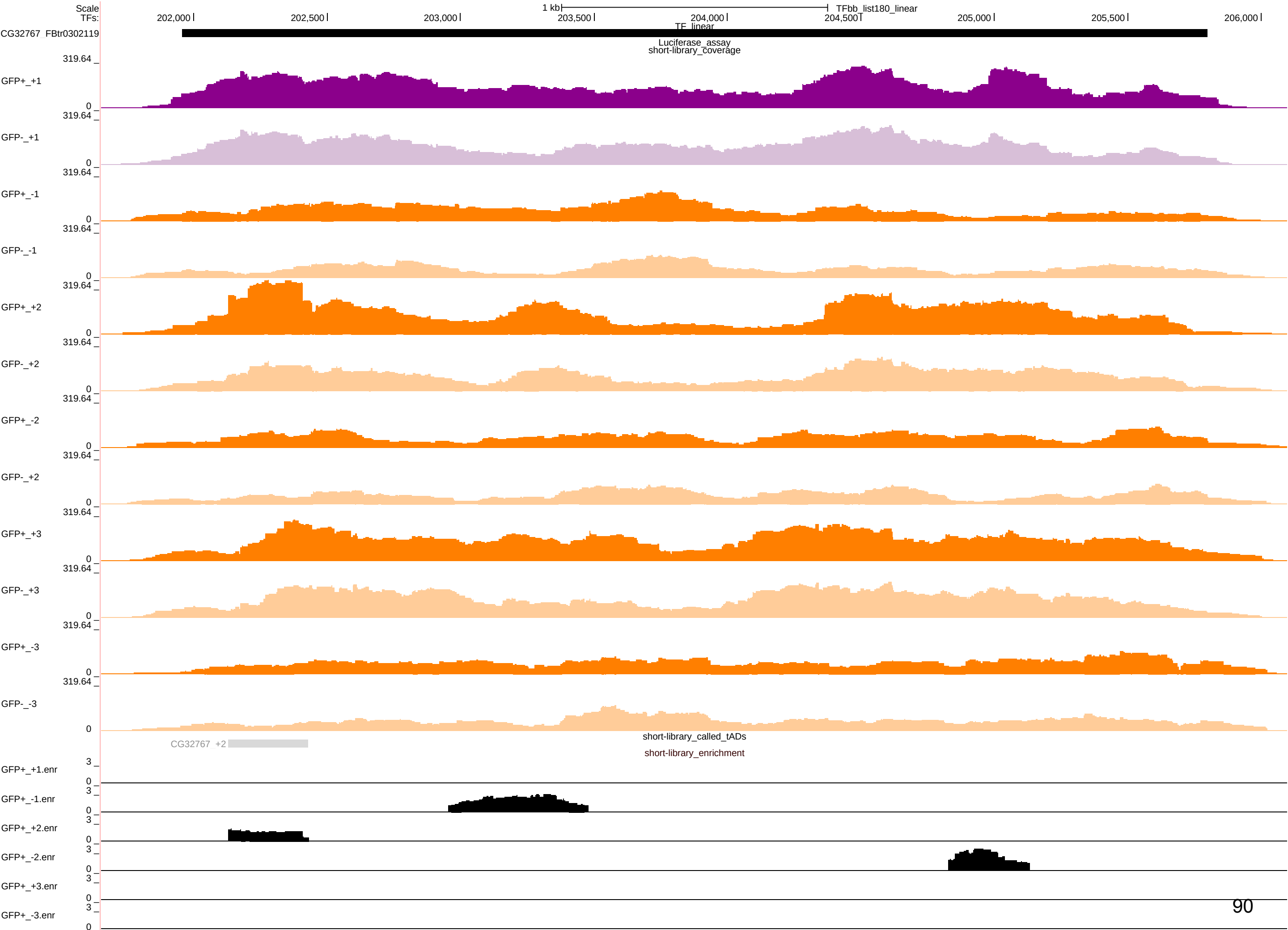
Scale
TFs:
1 kb
TFbb_list180_linear
202,000
202,500
203,000
203,500
204,000
204,500
205,000
205,500
206,000
TF_linear
CG32767_FBtr0302119
Luciferase_assay
short-library_coverage
GFP+_+1
GFP-_+1
GFP+_-1
GFP-_-1
GFP+_+2
GFP-_+2
GFP+_-2
GFP-_+2
GFP+_+3
GFP-_+3
GFP+_-3
GFP-_-3
319.64
0
short-library_called_tADs
CG32767_+2
short-library_enrichment
GFP+_+1.enr
GFP+_-1.enr
GFP+_+2.enr
GFP+_-2.enr
GFP+_+3.enr
GFP+_-3.enr
3
0

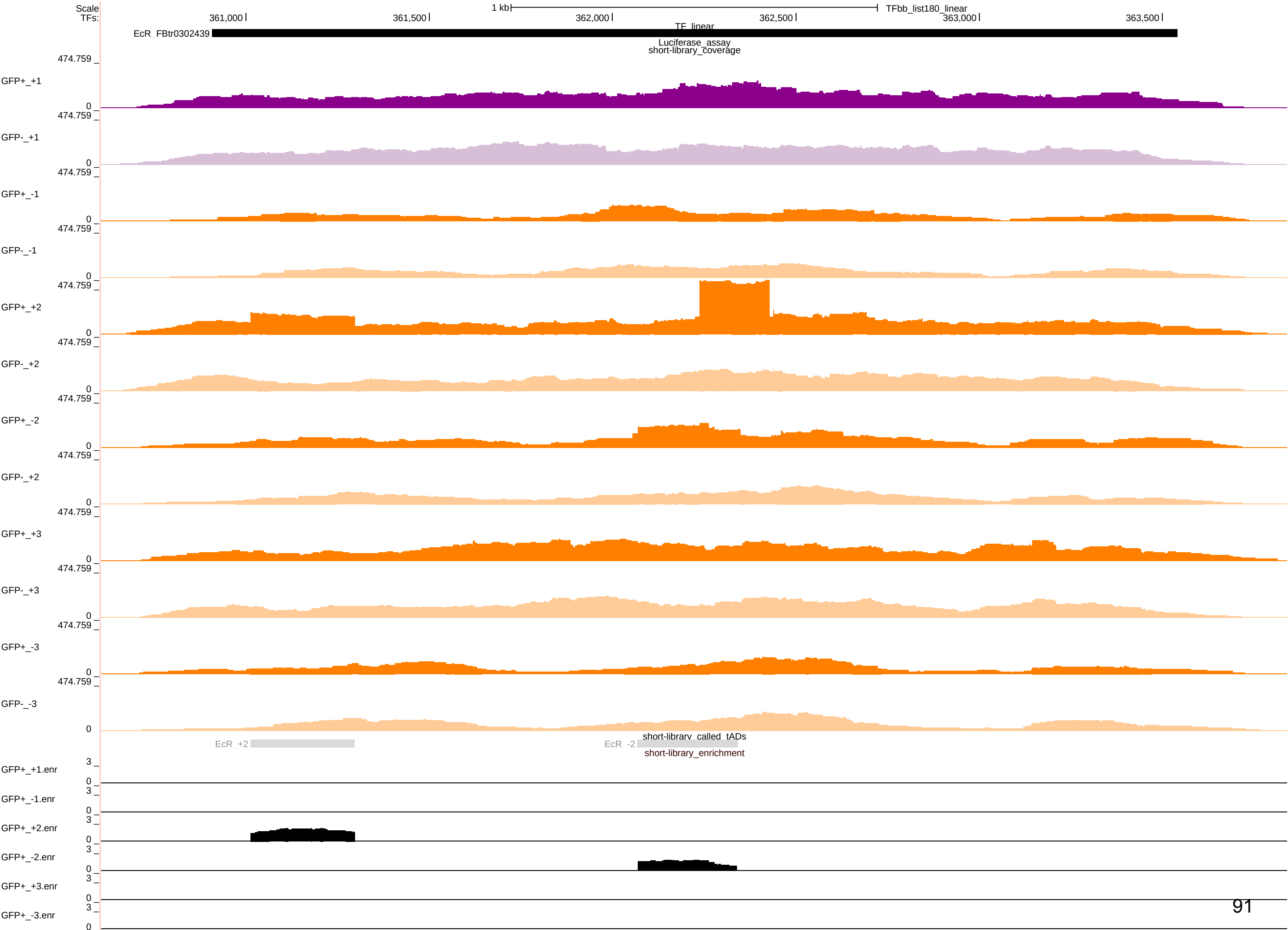
Scale
TFs:
1 kb
TFbb_list180_linear
361,000
361,500
362,000
362,500
363,000
363,500
TF_linear
EcR_FBtr0302439
Luciferase_assay
short-library_coverage
474.759
GFP+_+1
0
GFP-_+1
GFP+_-1
GFP-_-1
GFP+_+2
GFP-_+2
GFP+_-2
GFP-_+2
GFP+_+3
GFP-_+3
GFP+_-3
GFP-_-3
short-library_called_tADs
EcR_+2
EcR_-2
short-library_enrichment
3
GFP+_+1.enr
GFP+_-1.enr
GFP+_+2.enr
GFP+_-2.enr
GFP+_+3.enr
GFP+_-3.enr

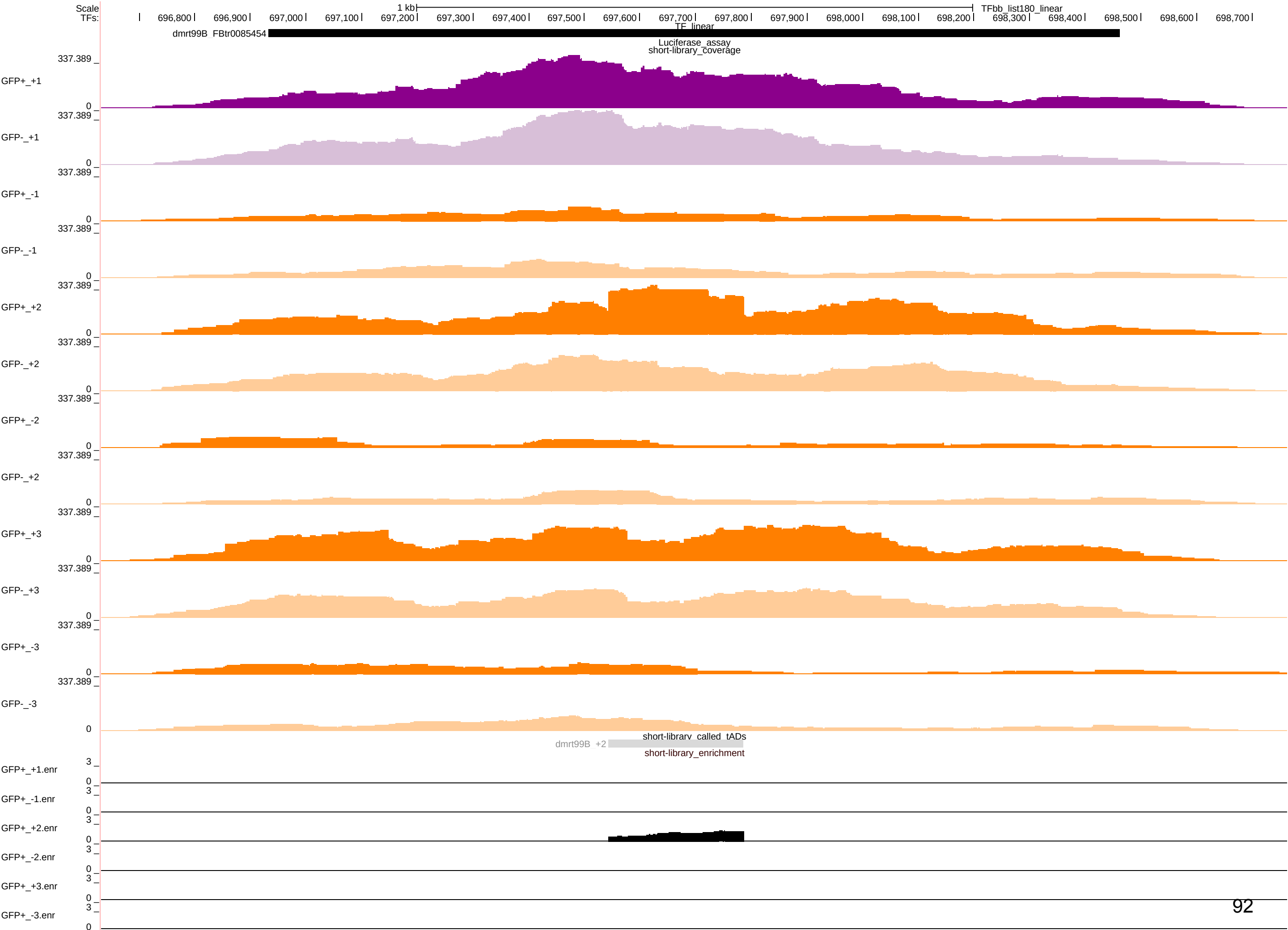

Scale
TFs:
1 kb
696,800
696,900
697,000
697,100
697,200
697,300
697,400
697,500
697,600
697,700
697,800
697,900
698,000
698,100
698,200
698,300
698,400
698,500
698,600
698,700
TFbb_list180_linear
TF_linear
dmrt99B_FBtr0085454
Luciferase_assay
short-library_coverage
GFP+_+1
GFP-_+1
GFP+_-1
GFP-_-1
GFP+_+2
GFP-_+2
GFP+_-2
GFP-_+2
GFP+_+3
GFP-_+3
GFP+_-3
GFP-_-3
337.389
0
short-library_called_tADs
dmrt99B_+2
short-library_enrichment
GFP+_+1.enr
GFP+_-1.enr
GFP+_+2.enr
GFP+_-2.enr
GFP+_+3.enr
GFP+_-3.enr
3

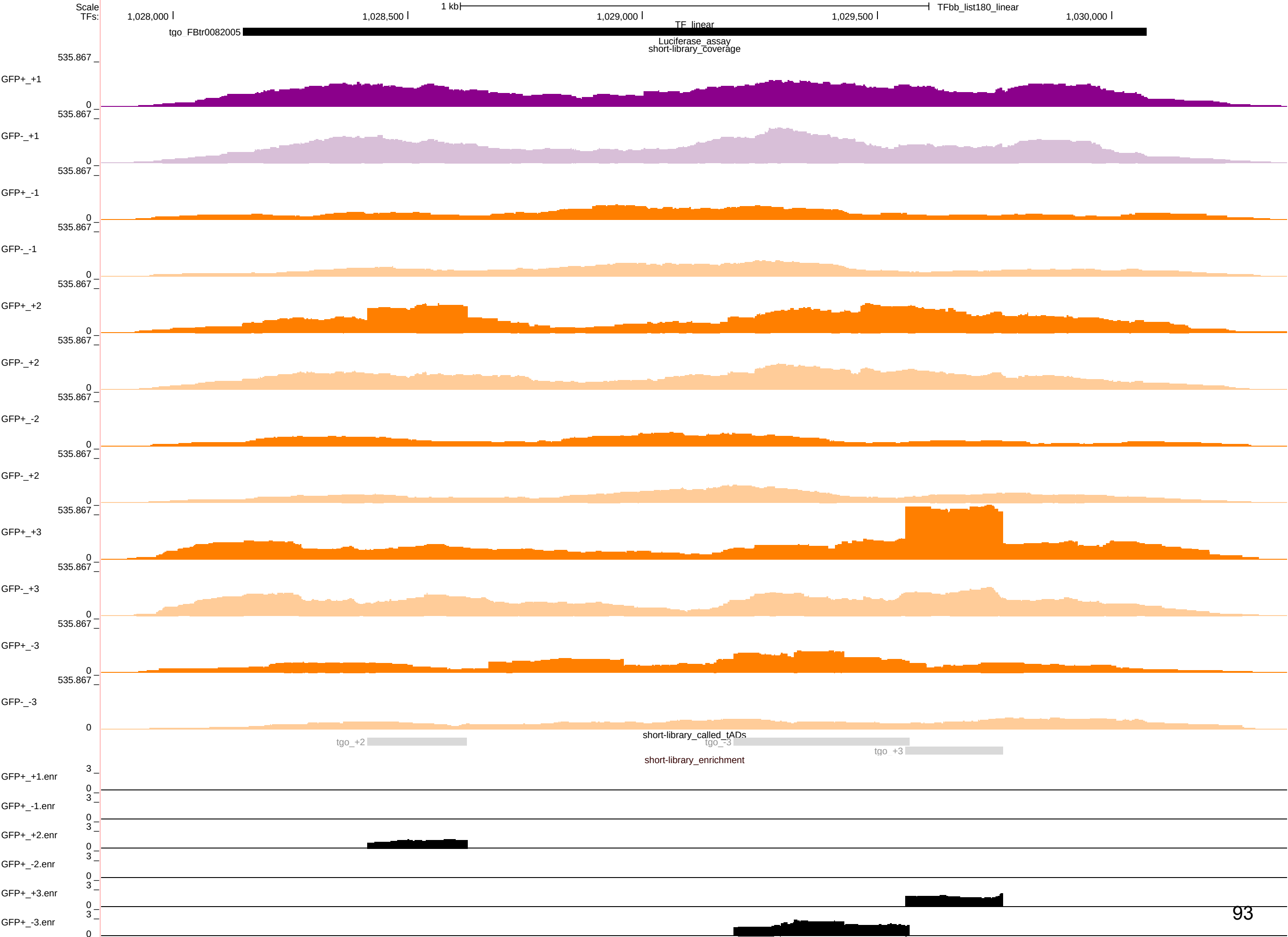
Scale
TFs:
1 kb
TFbb_list180_linear
1,028,000
1,028,500
1,029,000
1,029,500
1,030,000
TF_linear
tgo FBtr0082005
Luciferase_assay
short-library_coverage
535.867
0
GFP+_+1
GFP-_+1
GFP+_-1
GFP-_-1
GFP+_+2
GFP-_+2
GFP+_-2
GFP-_+2
GFP+_+3
GFP-_+3
GFP+_-3
GFP-_-3
short-library_called_tADs
tgo_+2
tgo_-3
tgo_+3
short-library_enrichment
3
GFP+_+1.enr
GFP+_-1.enr
GFP+_+2.enr
GFP+_-2.enr
GFP+_+3.enr
GFP+_-3.enr

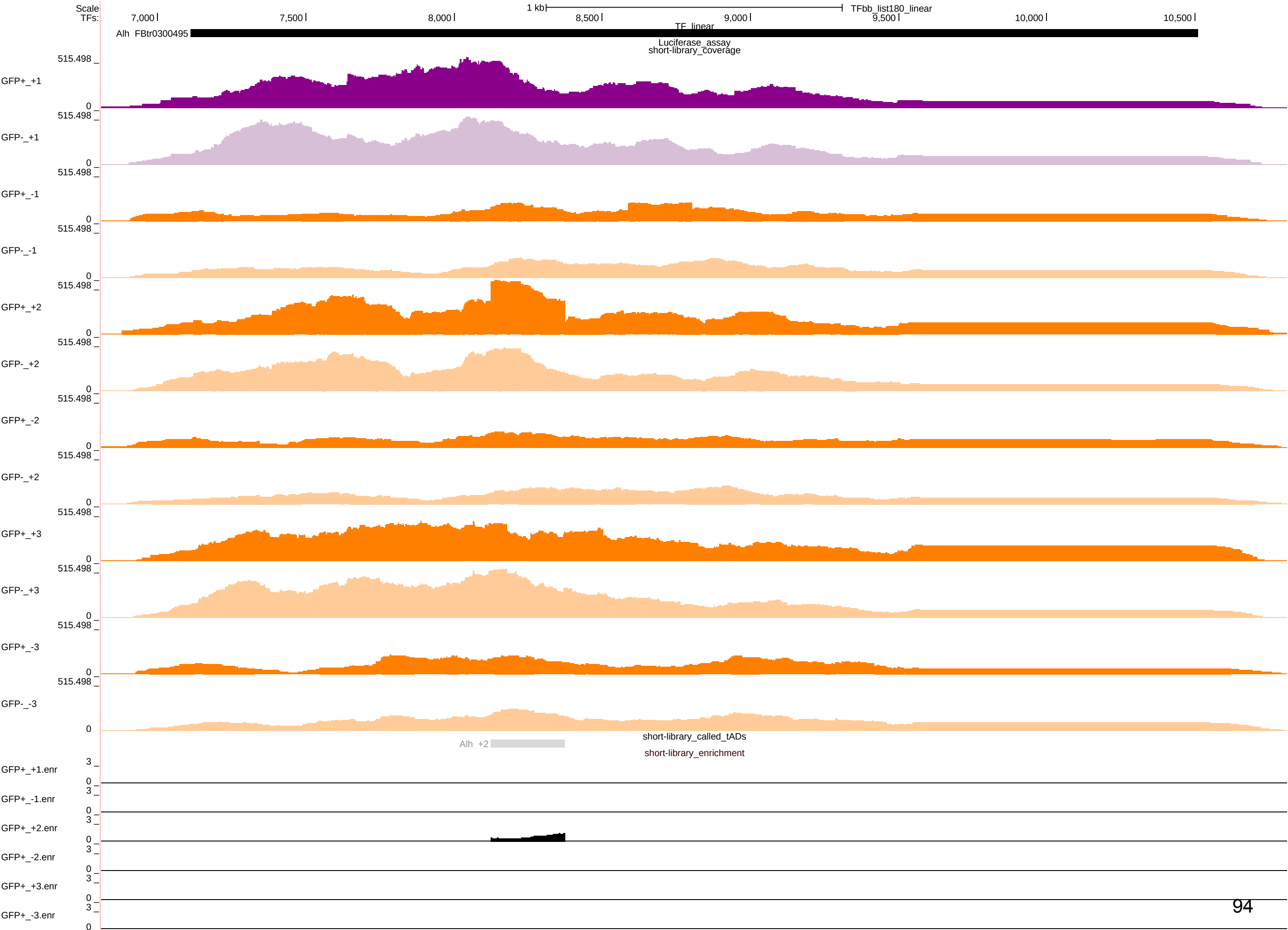

Scale
TFs:
1 kb
7,000
7,500
8,000
8,500
9,000
9,500
10,000
10,500
TFbb_list180_linear
TF_linear
Alh_FBtr0300495
Luciferase_assay
short-library_coverage
515.498
0
GFP+_+1
GFP-_+1
GFP+_-1
GFP-_-1
GFP+_+2
GFP-_+2
GFP+_-2
GFP-_+2
GFP+_+3
GFP-_+3
GFP+_-3
GFP-_-3
short-library_called_tADs
Alh_+2
short-library_enrichment
3
GFP+_+1.enr
GFP+_-1.enr
GFP+_+2.enr
GFP+_-2.enr
GFP+_+3.enr
GFP+_-3.enr

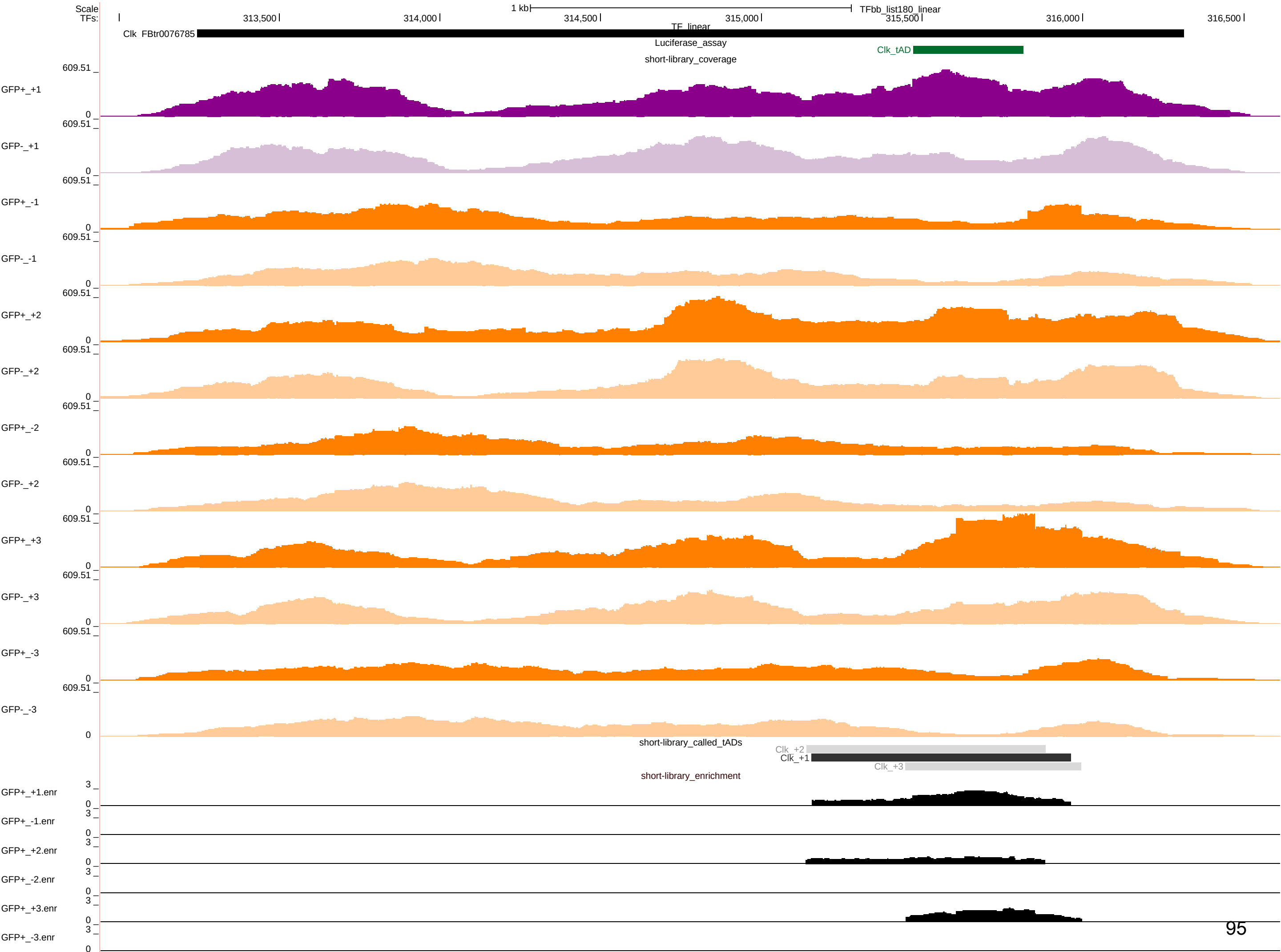

Scale
TFs:
1 kb
313,500
314,000
314,500
315,000
315,500
316,000
316,500
TFbb_list180_linear
TF_linear
Clk FBtr0076785
Luciferase_assay
Clk_tAD
short-library_coverage
609.51
0
GFP+_+1
GFP-_+1
GFP+_-1
GFP-_-1
GFP+_+2
GFP-_+2
GFP+_-2
GFP-_+2
GFP+_+3
GFP-_+3
GFP+_-3
GFP-_-3
short-library_called_tADs
Clk_+2
Clk_+1
Clk_+3
short-library_enrichment
3
GFP+_+1.enr
GFP+_-1.enr
GFP+_+2.enr
GFP+_-2.enr
GFP+_+3.enr
GFP+_-3.enr

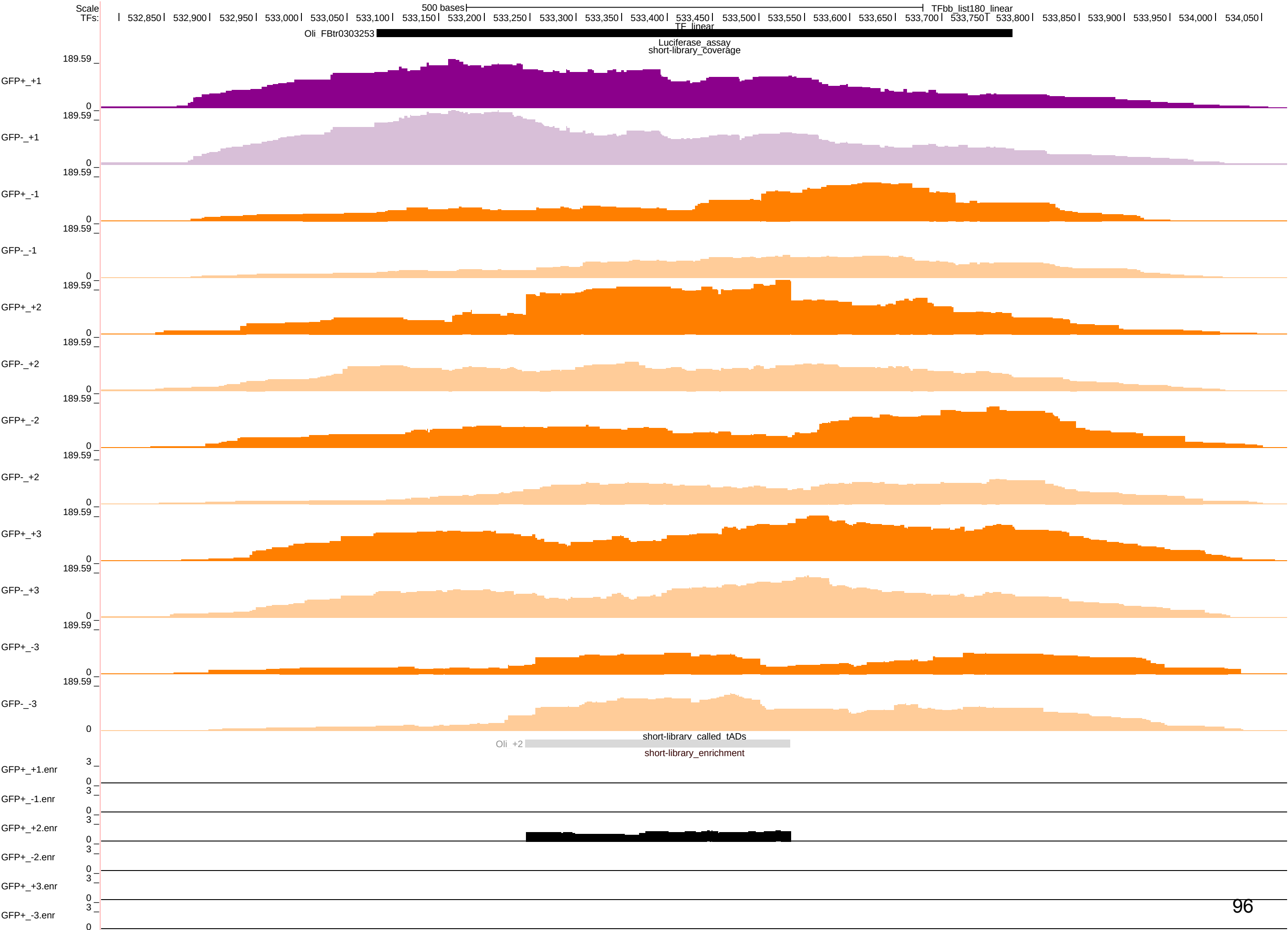
Scale
TFs:
500 bases
TFbb_list180_linear
532,850
532,900
532,950
533,000
533,050
533,100
533,150
533,200
533,250
533,300
533,350
533,400
533,450
533,500
533,550
533,600
533,650
533,700
533,750
533,800
533,850
533,900
533,950
534,000
534,050
TF_linear
Oli_FBtr0303253
Luciferase_assay
short-library_coverage
189.59
0
GFP+_+1
GFP-_+1
GFP+_-1
GFP-_-1
GFP+_+2
GFP-_+2
GFP+_-2
GFP-_+2
GFP+_+3
GFP-_+3
GFP+_-3
GFP-_-3
short-library_called_tADs
Oli_+2
short-library_enrichment
3
GFP+_+1.enr
GFP+_-1.enr
GFP+_+2.enr
GFP+_-2.enr
GFP+_+3.enr
GFP+_-3.enr

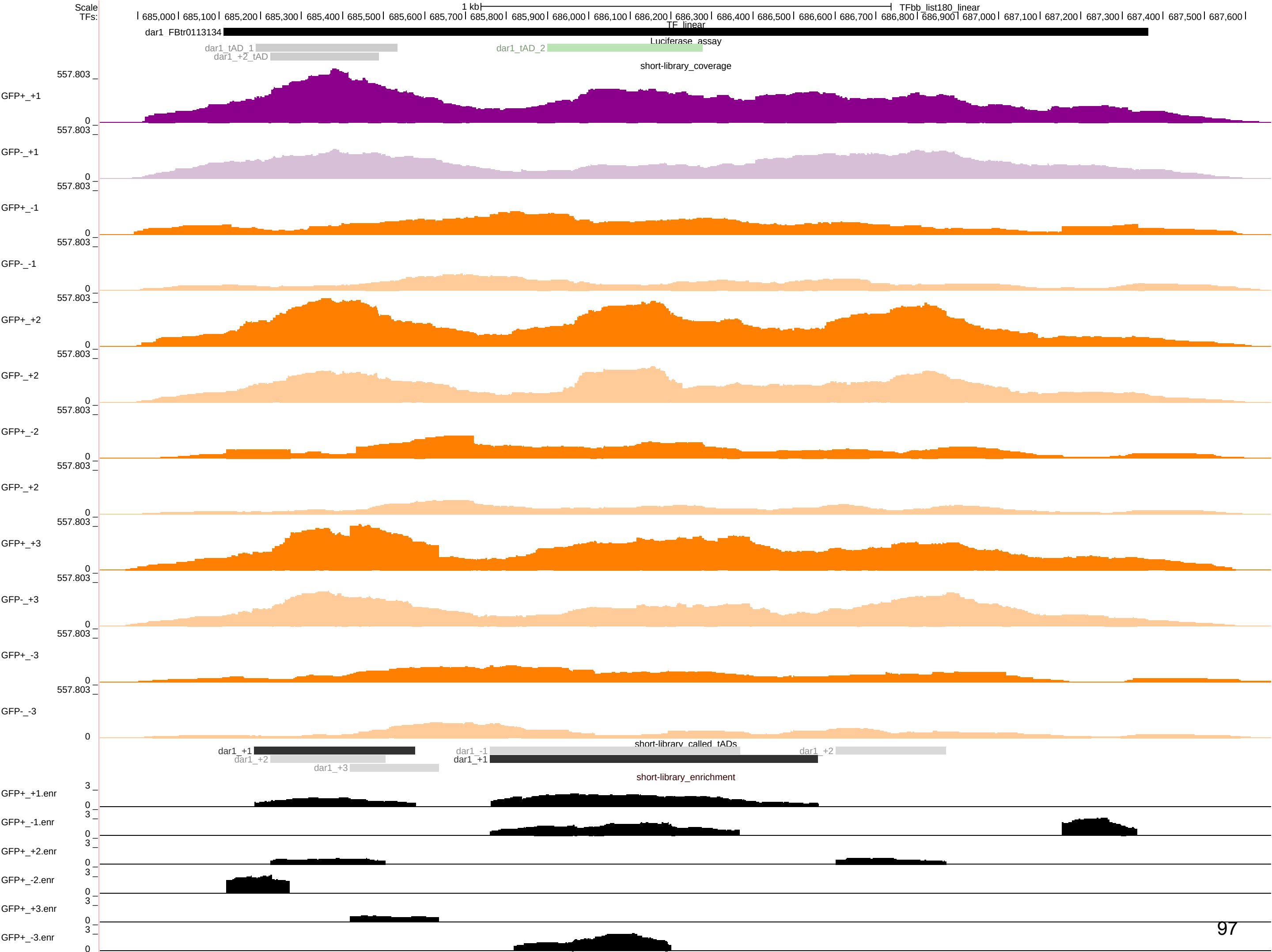

Scale
TFs:
1 kb
TFbb_list180_linear
685,000 685,100 685,200 685,300 685,400 685,500 685,600 685,700 685,800 685,900 686,000 686,100 686,200 686,300 686,400 686,500 686,600 686,700 686,800 686,900 687,000 687,100 687,200 687,300 687,400 687,500 687,600
TF_linear
dar1 FBtr0113134
Luciferase_assay
dar1_tAD_1
dar1_+2_tAD
dar1_tAD_2
short-library_coverage
GFP+_+1
GFP-_+1
GFP+_-1
GFP-_-1
GFP+_+2
GFP-_+2
GFP+_-2
GFP-_+2
GFP+_+3
GFP-_+3
GFP+_-3
GFP-_-3
557.803
0
short-library_called_tADs
dar1_+1
dar1_+2
dar1_+3
dar1_-1
dar1_+1
dar1_+2
short-library_enrichment
GFP+_+1.enr
GFP+_-1.enr
GFP+_+2.enr
GFP+_-2.enr
GFP+_+3.enr
GFP+_-3.enr
3
0

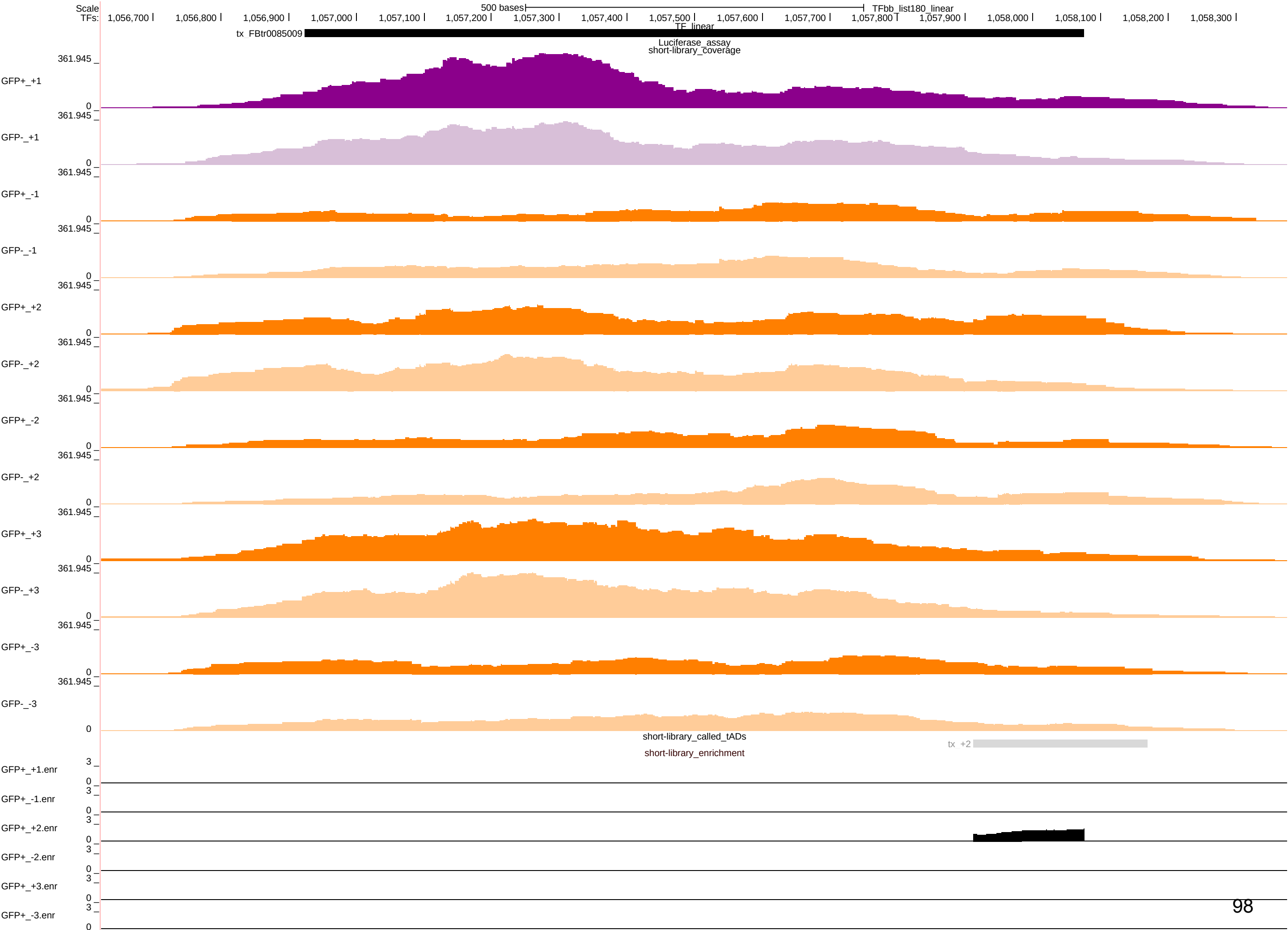

Scale
500 bases
TFbb_list180_linear
TFs:
1,056,700
1,056,800
1,056,900
1,057,000
1,057,100
1,057,200
1,057,300
1,057,400
1,057,500
1,057,600
1,057,700
1,057,800
1,057,900
1,058,000
1,058,100
1,058,200
1,058,300
TF_linear
tx FBtr0085009
Luciferase_assay
short-library_coverage
GFP+_+1
GFP-_+1
GFP+_-1
GFP-_-1
GFP+_+2
GFP-_+2
GFP+_-2
GFP-_+2
GFP+_+3
GFP-_+3
GFP+_-3
GFP-_-3
361.945
0
short-library_called_tADs
tx_+2
short-library_enrichment
GFP+_+1.enr
GFP+_-1.enr
GFP+_+2.enr
GFP+_-2.enr
GFP+_+3.enr
GFP+_-3.enr
3
0

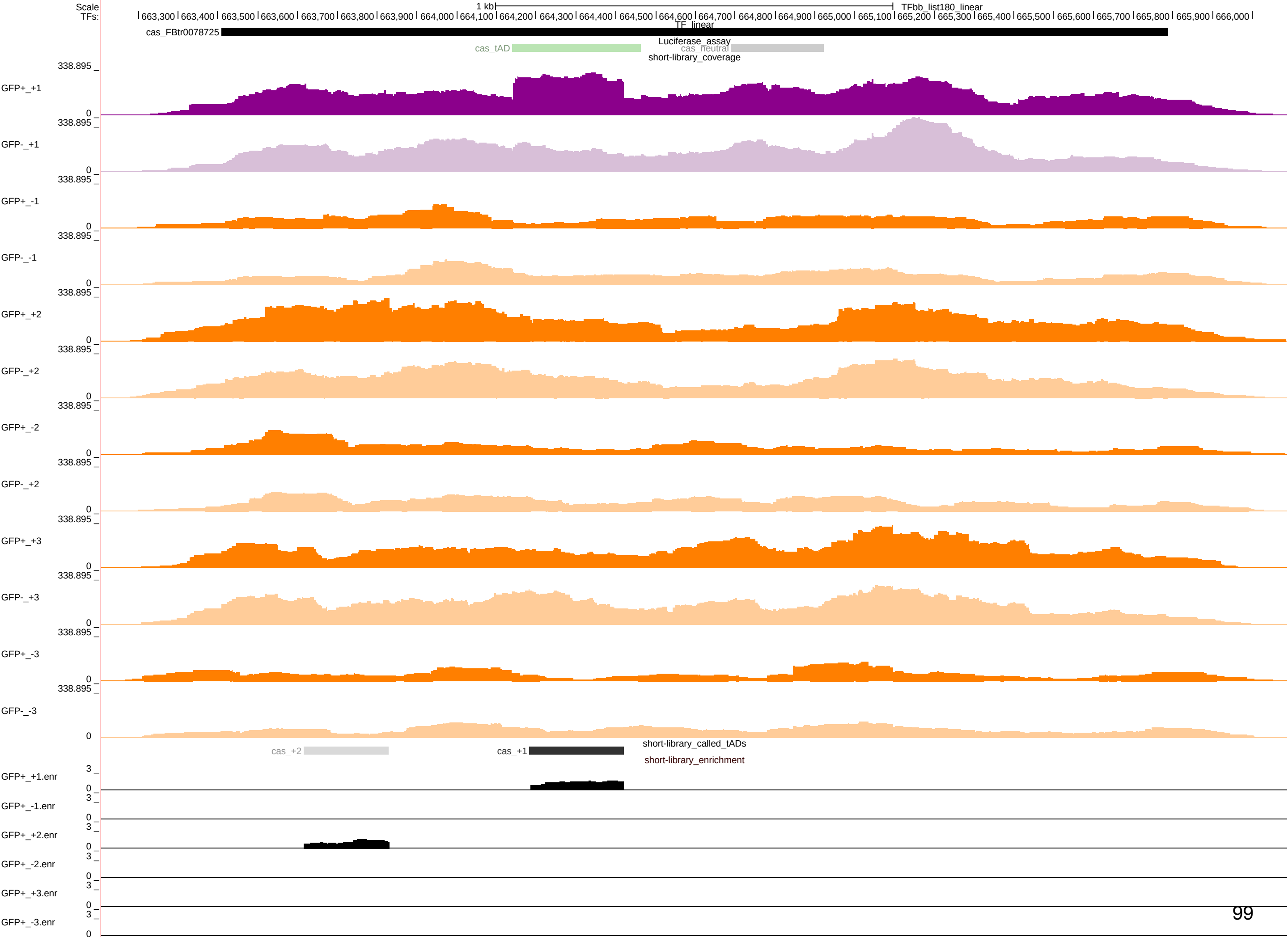

Scale
TFs:
1 kb
TFbb_list180_linear
663,300 663,400 663,500 663,600 663,700 663,800 663,900 664,000 664,100 664,200 664,300 664,400 664,500 664,600 664,700 664,800 664,900 665,000 665,100 665,200 665,300 665,400 665,500 665,600 665,700 665,800 665,900 666,000
TF_linear
cas_FBtr0078725
Luciferase_assay
cas_tAD
cas_neutral
short-library_coverage
GFP+_+1
GFP-_+1
GFP+_-1
GFP-_-1
GFP+_+2
GFP-_+2
GFP+_-2
GFP-_+2
GFP+_+3
GFP-_+3
GFP+_-3
GFP-_-3
338.895
0
short-library_called_tADs
cas_+2
cas_+1
short-library_enrichment
GFP+_+1.enr
GFP+_-1.enr
GFP+_+2.enr
GFP+_-2.enr
GFP+_+3.enr
GFP+_-3.enr
3
0

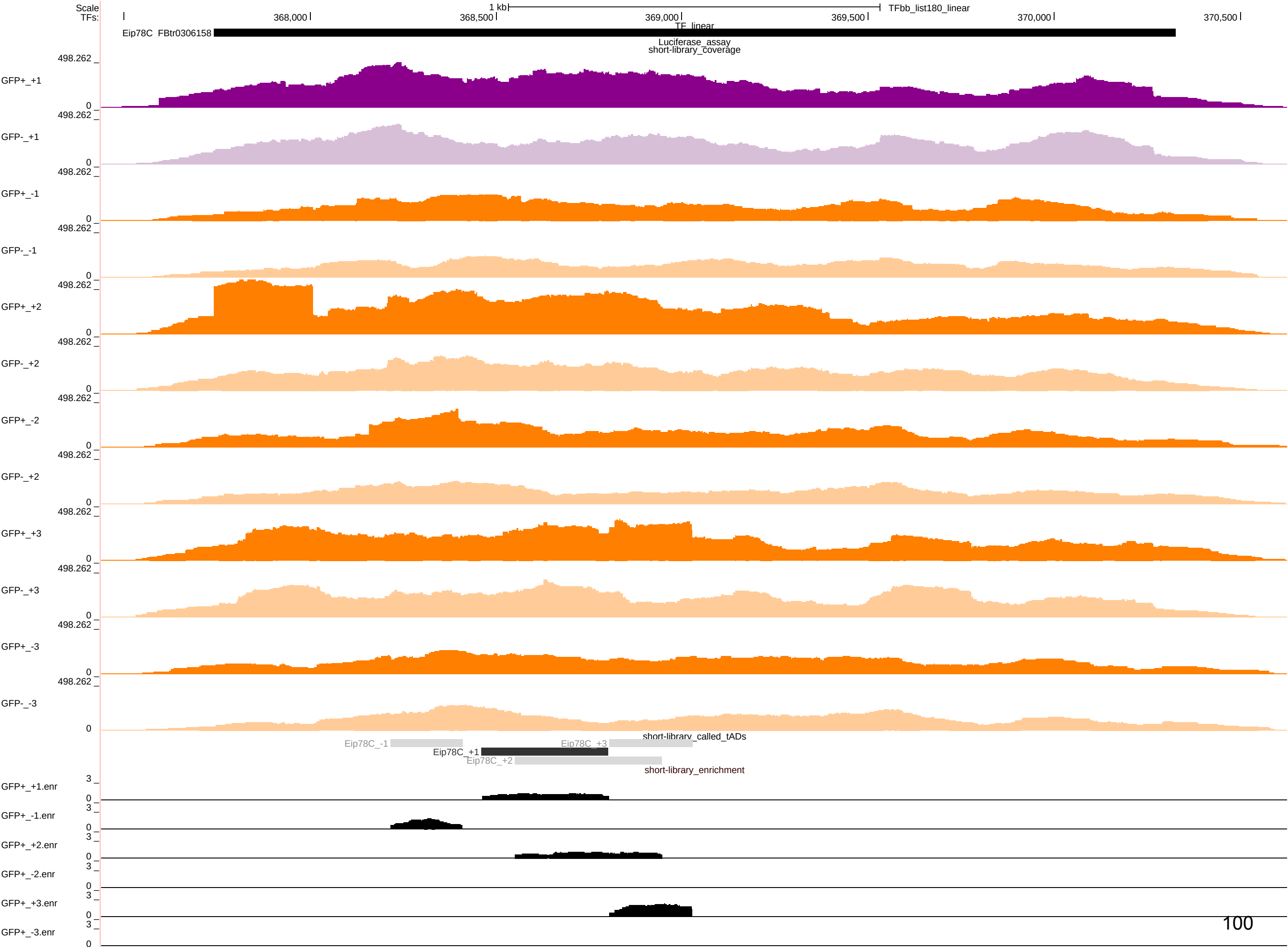
Scale
TFs:
1 kb
TFbb_list180_linear
368,000
368,500
369,000
369,500
370,000
370,500
Eip78C FBtr0306158
TF_linear
Luciferase_assay
short-library_coverage
498.262
0
GFP+_+1
GFP-_+1
GFP+_-1
GFP-_-1
GFP+_+2
GFP-_+2
GFP+_-2
GFP-_+2
GFP+_+3
GFP-_+3
GFP+_-3
GFP-_-3
short-library_called_tADs
Eip78C_-1
Eip78C_+3
Eip78C_+1
Eip78C_+2
short-library_enrichment
3
GFP+_+1.enr
GFP+_-1.enr
GFP+_+2.enr
GFP+_-2.enr
GFP+_+3.enr
GFP+_-3.enr

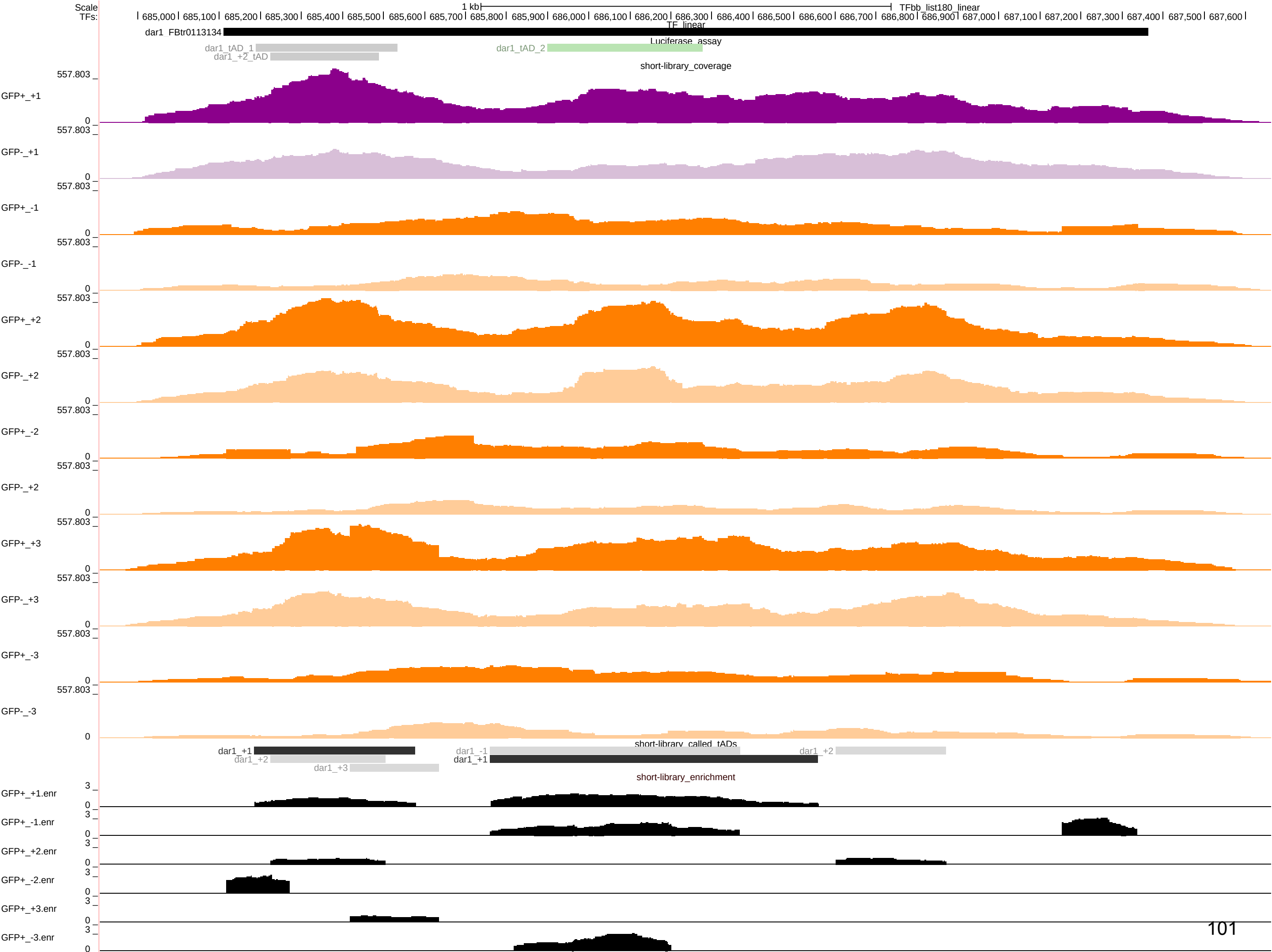

Scale
TFs:
1 kb
TFbb_list180_linear
685,000 685,100 685,200 685,300 685,400 685,500 685,600 685,700 685,800 685,900 686,000 686,100 686,200 686,300 686,400 686,500 686,600 686,700 686,800 686,900 687,000 687,100 687,200 687,300 687,400 687,500 687,600
TF_linear
dar1 FBtr0113134
Luciferase_assay
dar1_tAD_1
dar1_+2_tAD
dar1_tAD_2
short-library_coverage
557.803
0
GFP+_+1
GFP-_+1
GFP+_-1
GFP-_-1
GFP+_+2
GFP-_+2
GFP+_-2
GFP-_+2
GFP+_+3
GFP-_+3
GFP+_-3
GFP-_-3
short-library_called_tADs
dar1_+1
dar1_+2
dar1_+3
dar1_-1
dar1_+1
dar1_+2
short-library_enrichment
3
0
GFP+_+1.enr
GFP+_-1.enr
GFP+_+2.enr
GFP+_-2.enr
GFP+_+3.enr
GFP+_-3.enr

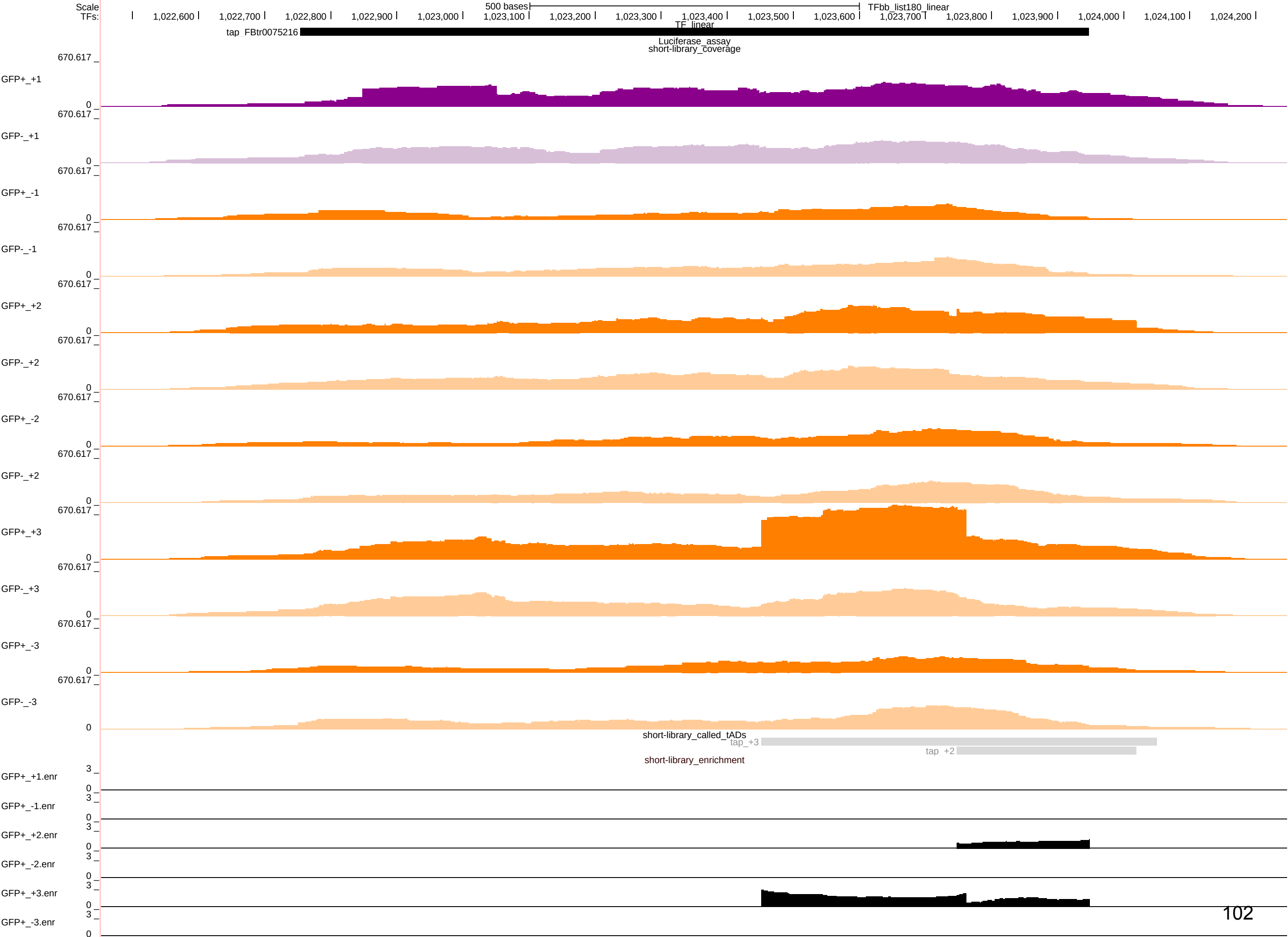

Scale
TFs:
500 bases
1,022,600
1,022,700
1,022,800
1,022,900
1,023,000
1,023,100
1,023,200
1,023,300
1,023,400
1,023,500
1,023,600
1,023,700
1,023,800
1,023,900
1,024,000
1,024,100
1,024,200
TFbb_list180_linear
TF_linear
tap FBtr0075216
Luciferase_assay
short-library_coverage
GFP+_+1
GFP-_+1
GFP+_-1
GFP-_-1
GFP+_+2
GFP-_+2
GFP+_-2
GFP-_+2
GFP+_+3
GFP-_+3
GFP+_-3
GFP-_-3
670.617
0
short-library_called_tADs
tap_+3
tap_+2
short-library_enrichment
GFP+_+1.enr
GFP+_-1.enr
GFP+_+2.enr
GFP+_-2.enr
GFP+_+3.enr
GFP+_-3.enr
3
0

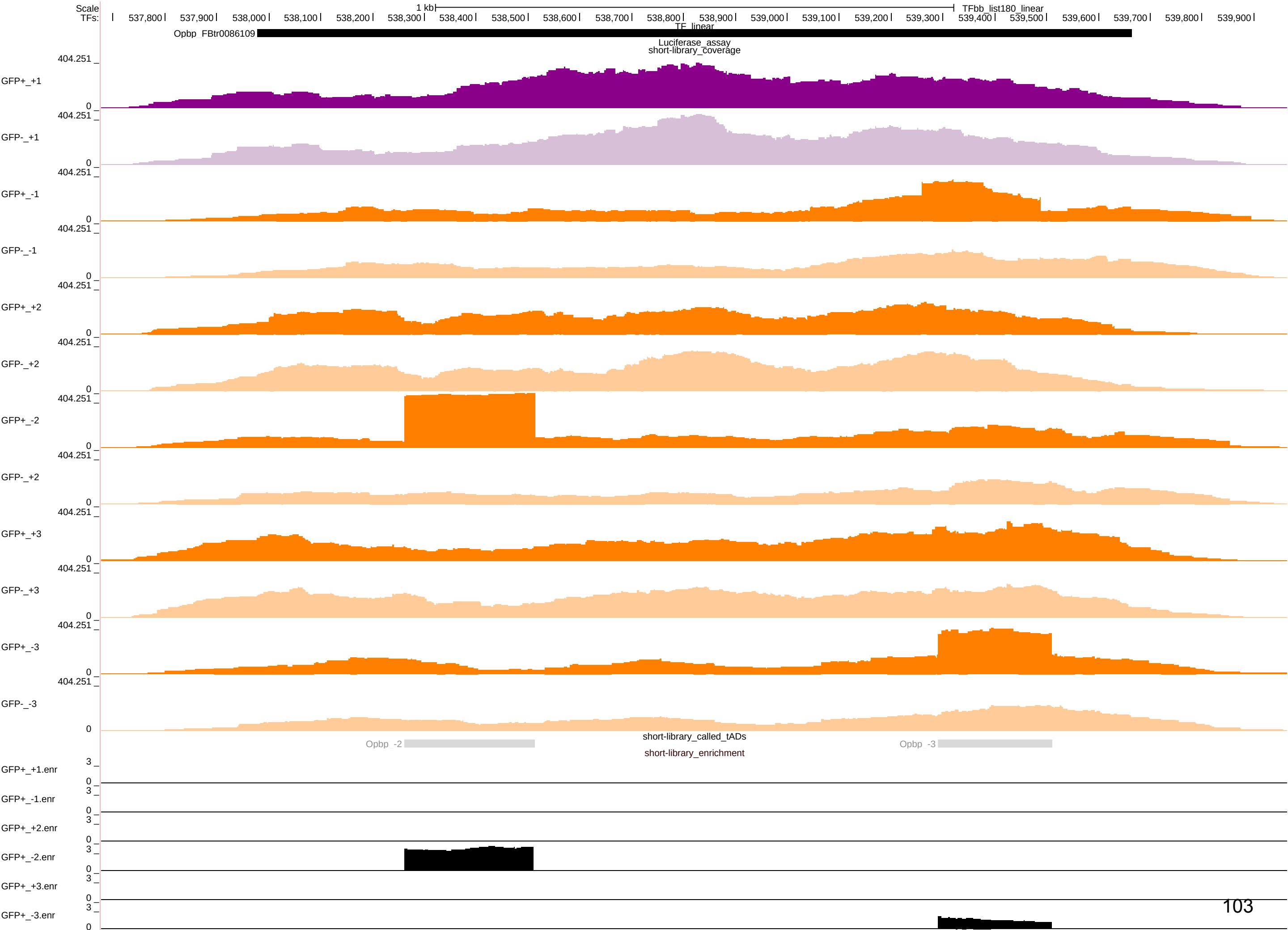
Scale
1 kb
TFbb_list180_linear
TFs:
537,800
537,900
538,000
538,100
538,200
538,300
538,400
538,500
538,600
538,700
538,800
538,900
539,000
539,100
539,200
539,300
539,400
539,500
539,600
539,700
539,800
539,900
TF_linear
Opbp FBtr0086109
Luciferase_assay
short-library_coverage
GFP+_+1
GFP-_+1
GFP+_-1
GFP-_-1
GFP+_+2
GFP-_+2
GFP+_-2
GFP-_+2
GFP+_+3
GFP-_+3
GFP+_-3
GFP-_-3
404.251
0
short-library_called_tADs
Opbp_-2
Opbp_-3
short-library_enrichment
GFP+_+1.enr
GFP+_-1.enr
GFP+_+2.enr
GFP+_-2.enr
GFP+_+3.enr
GFP+_-3.enr
3
0

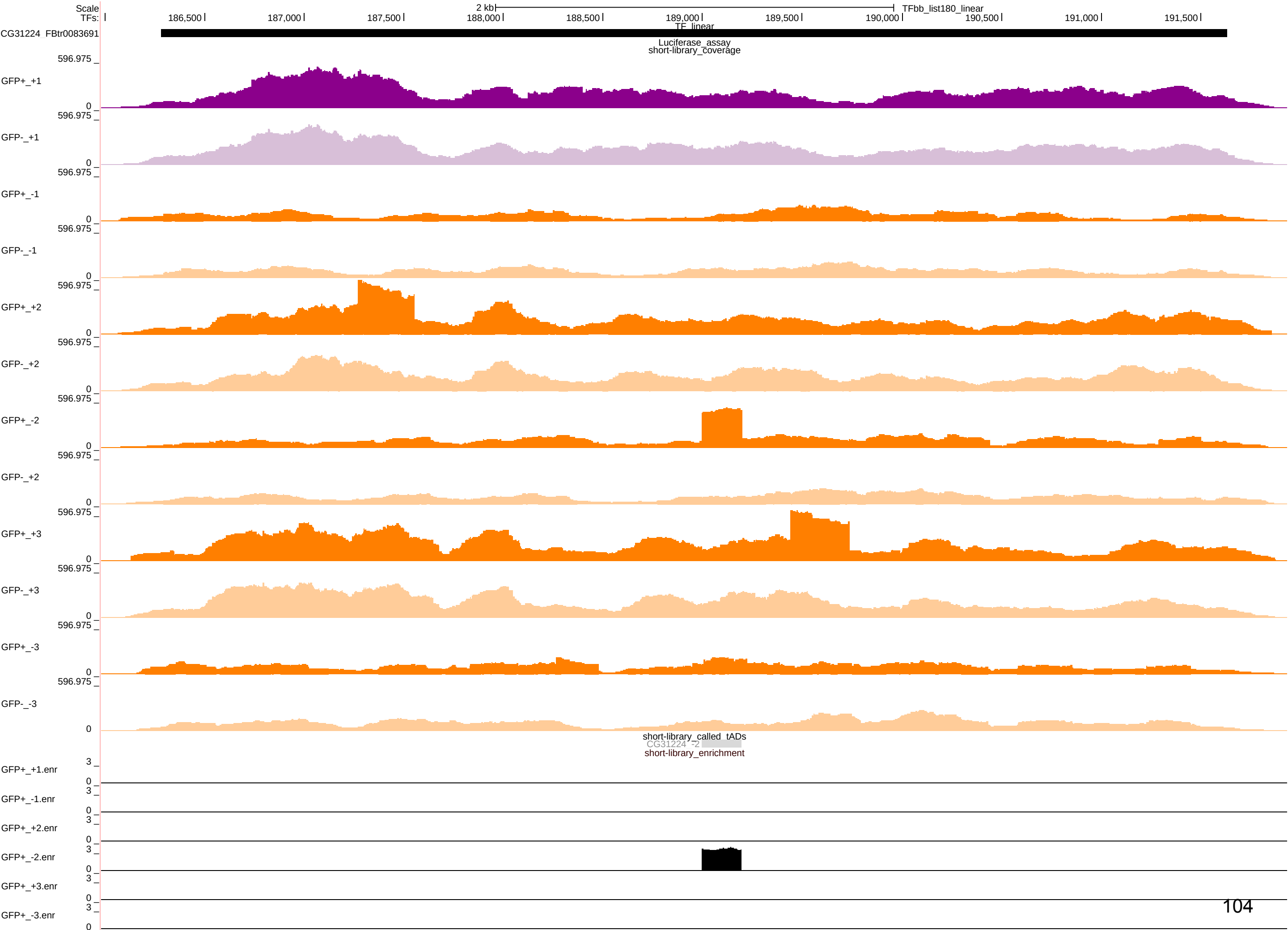

Scale
TFs:
2 kb
TFbb_list180_linear
186,500
187,000
187,500
188,000
188,500
189,000
189,500
190,000
190,500
191,000
191,500
TF_linear
CG31224_FBtr0083691
Luciferase_assay
short-library_coverage
596.975
0
GFP+_+1
GFP-_+1
GFP+_-1
GFP-_-1
GFP+_+2
GFP-_+2
GFP+_-2
GFP-_+2
GFP+_+3
GFP-_+3
GFP+_-3
GFP-_-3
short-library_called_tADs
CG31224_-2
short-library_enrichment
3
GFP+_+1.enr
GFP+_-1.enr
GFP+_+2.enr
GFP+_-2.enr
GFP+_+3.enr
GFP+_-3.enr

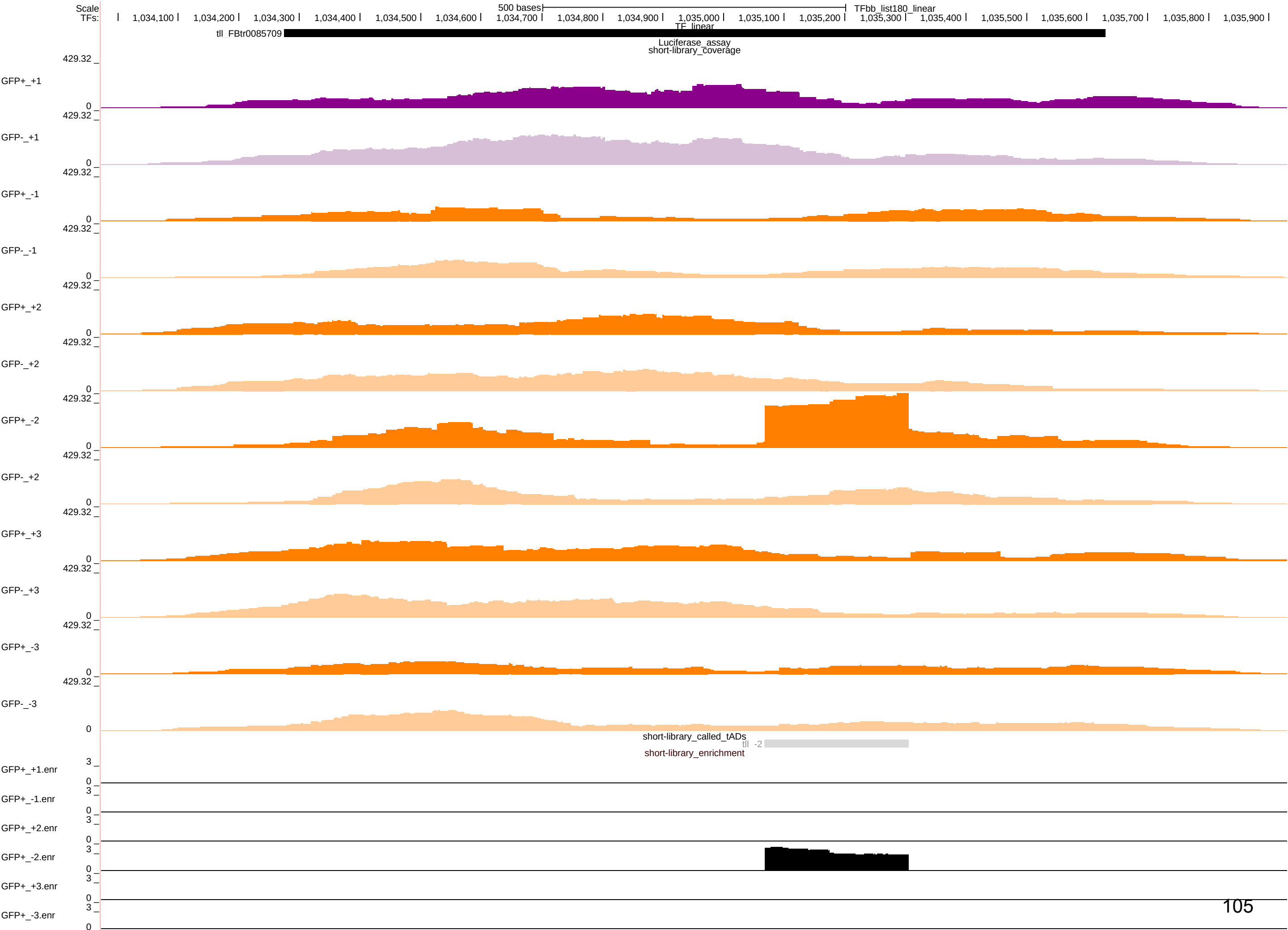
Scale
TFs:
500 bases
TFbb_list180_linear
1,034,100
1,034,200
1,034,300
1,034,400
1,034,500
1,034,600
1,034,700
1,034,800
1,034,900
1,035,000
1,035,100
1,035,200
1,035,300
1,035,400
1,035,500
1,035,600
1,035,700
1,035,800
1,035,900
TF_linear
tll FBtr0085709
Luciferase_assay
short-library_coverage
GFP+_+1
GFP-_+1
GFP+_-1
GFP-_-1
GFP+_+2
GFP-_+2
GFP+_-2
GFP-_+2
GFP+_+3
GFP-_+3
GFP+_-3
GFP-_-3
429.32
0
short-library_called_tADs
tll -2
short-library_enrichment
GFP+_+1.enr
GFP+_-1.enr
GFP+_+2.enr
GFP+_-2.enr
GFP+_+3.enr
GFP+_-3.enr
3
0

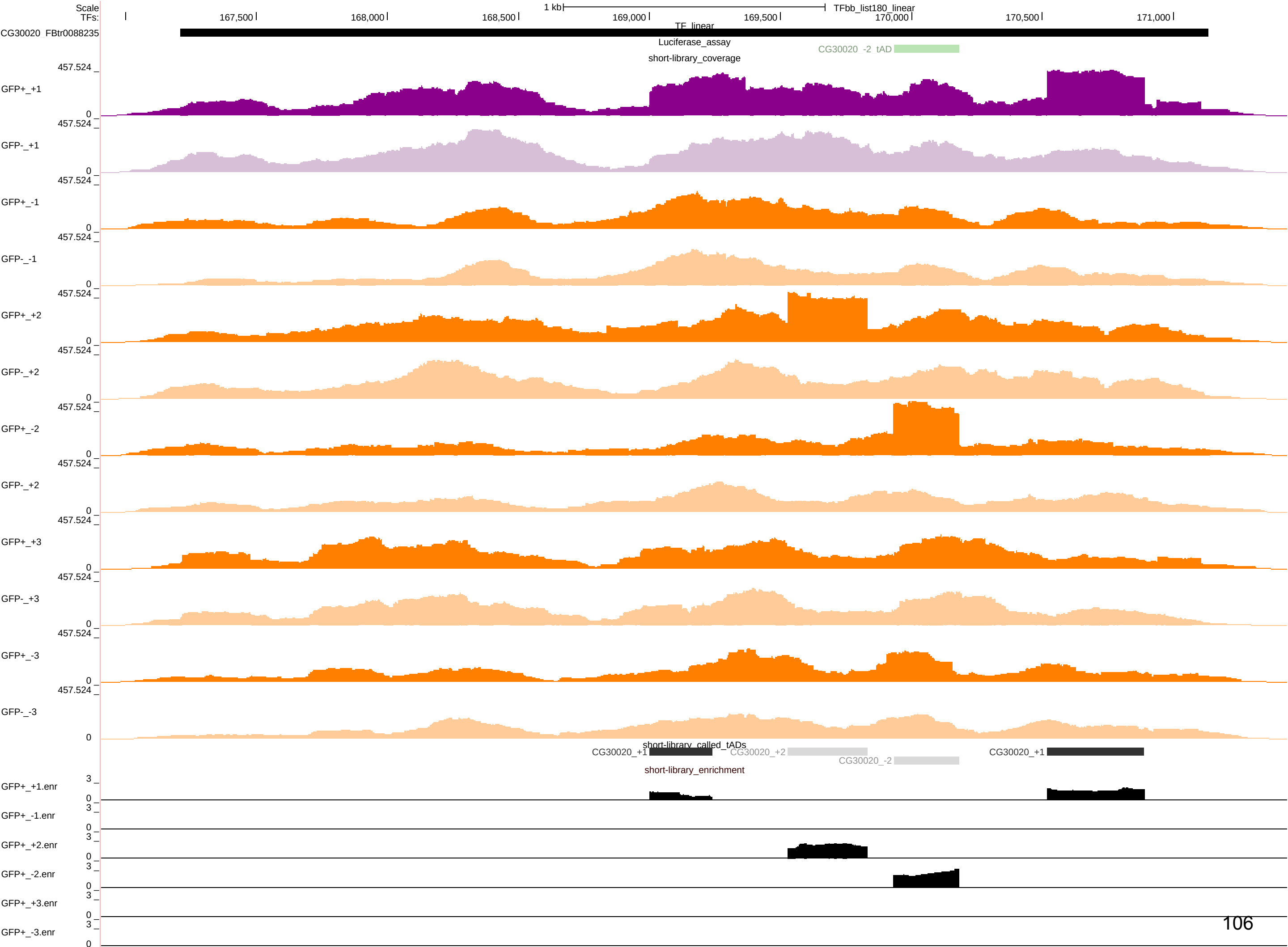
Scale
1 kb
TFbb_list180_linear
TFs:
167,500
168,000
168,500
169,000
169,500
170,000
170,500
171,000
CG30020 FBtr0088235
TF_linear
Luciferase_assay
CG30020 -2 tAD
short-library_coverage
457.524
GFP+_+1
0
GFP-_+1
GFP+_-1
GFP-_-1
GFP+_+2
GFP-_+2
GFP+_-2
GFP-_+2
GFP+_+3
GFP-_+3
GFP+_-3
GFP-_-3
short-library_called_tADs
CG30020_+1
CG30020_+2
CG30020_-2
CG30020_+1
short-library_enrichment
3
GFP+_+1.enr
GFP+_-1.enr
GFP+_+2.enr
GFP+_-2.enr
GFP+_+3.enr
GFP+_-3.enr

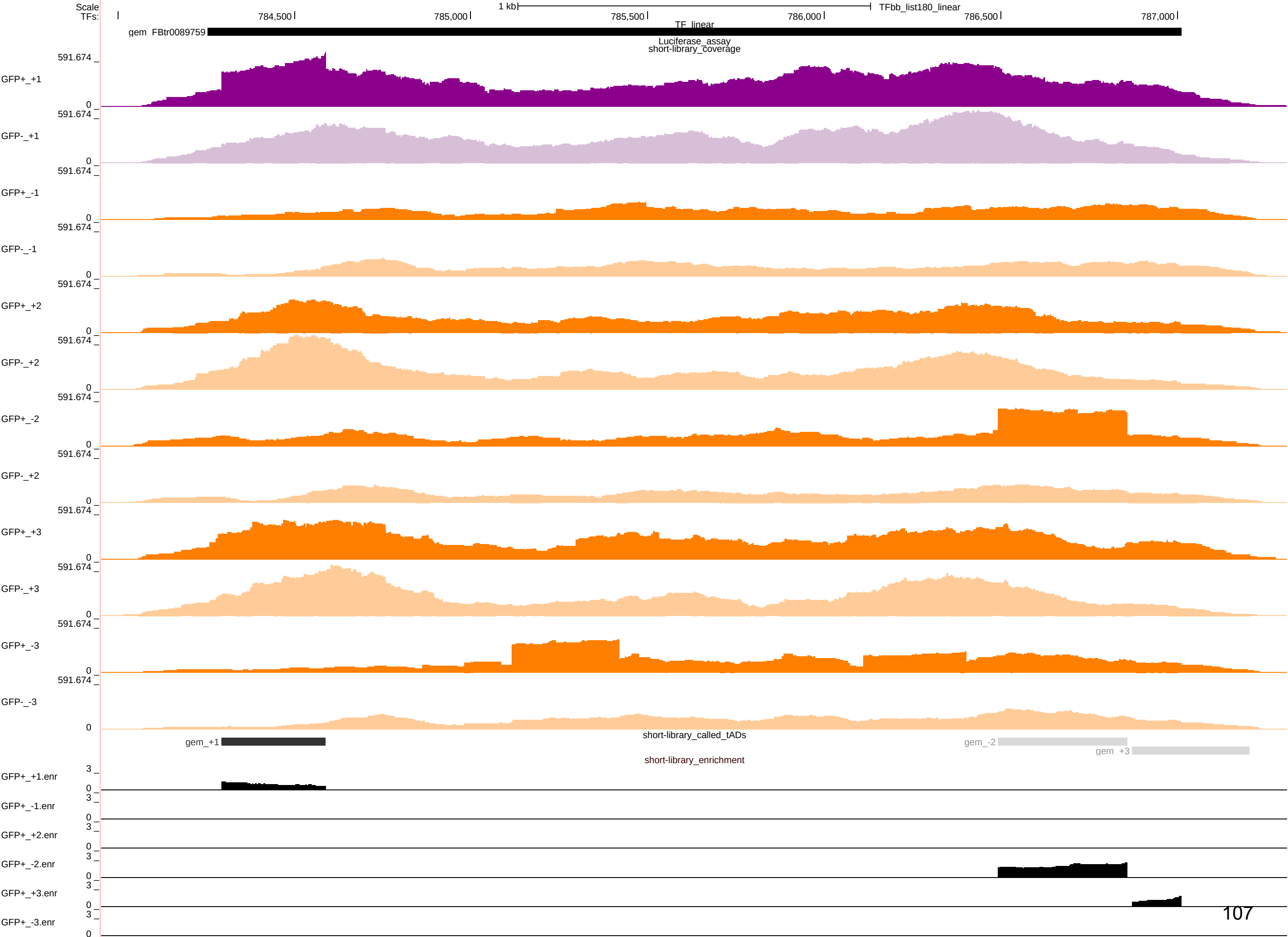
Scale
TFs:
1 kb
TFbb_list180_linear
784,500
785,000
785,500
786,000
786,500
787,000
TF_linear
gem_FBtr0089759
Luciferase_assay
short-library_coverage
591.674
0
GFP+_+1
GFP-_+1
GFP+_-1
GFP-_-1
GFP+_+2
GFP-_+2
GFP+_-2
GFP-_+2
GFP+_+3
GFP-_+3
GFP+_-3
GFP-_-3
short-library_called_tADs
gem_+1
gem_-2
gem_+3
short-library_enrichment
3
GFP+_+1.enr
GFP+_-1.enr
GFP+_+2.enr
GFP+_-2.enr
GFP+_+3.enr
GFP+_-3.enr

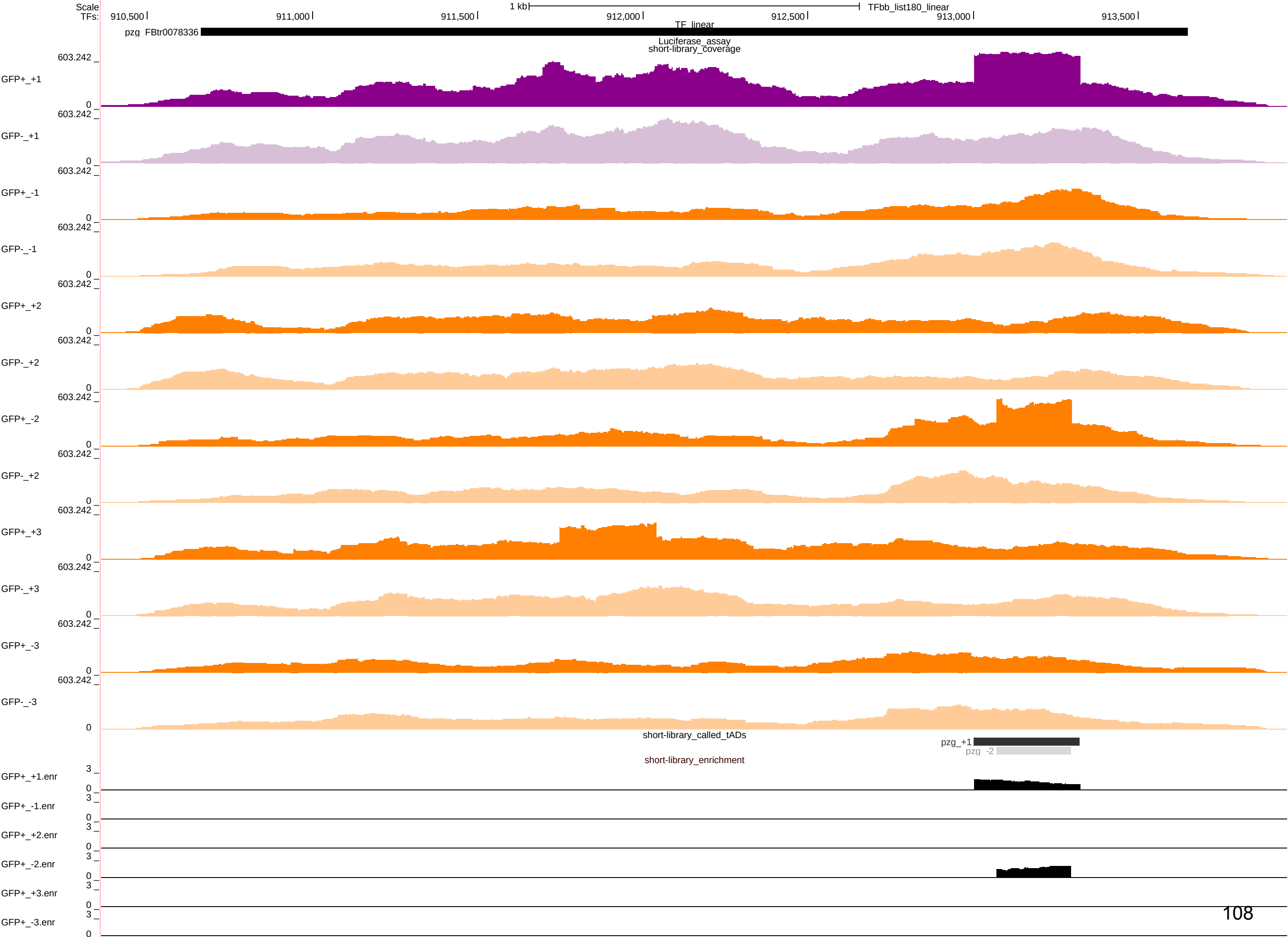
Scale
TFs:
1 kb
TFbb_list180_linear
910,500
911,000
911,500
912,000
912,500
913,000
913,500
TF_linear
pzg_FBtr0078336
Luciferase_assay
short-library_coverage
GFP+_+1
GFP-_+1
GFP+_-1
GFP-_-1
GFP+_+2
GFP-_+2
GFP+_-2
GFP-_+2
GFP+_+3
GFP-_+3
GFP+_-3
GFP-_-3
603.242
0
short-library_called_tADs
pzg_+1
pzg_-2
short-library_enrichment
GFP+_+1.enr
GFP+_-1.enr
GFP+_+2.enr
GFP+_-2.enr
GFP+_+3.enr
GFP+_-3.enr
3
0

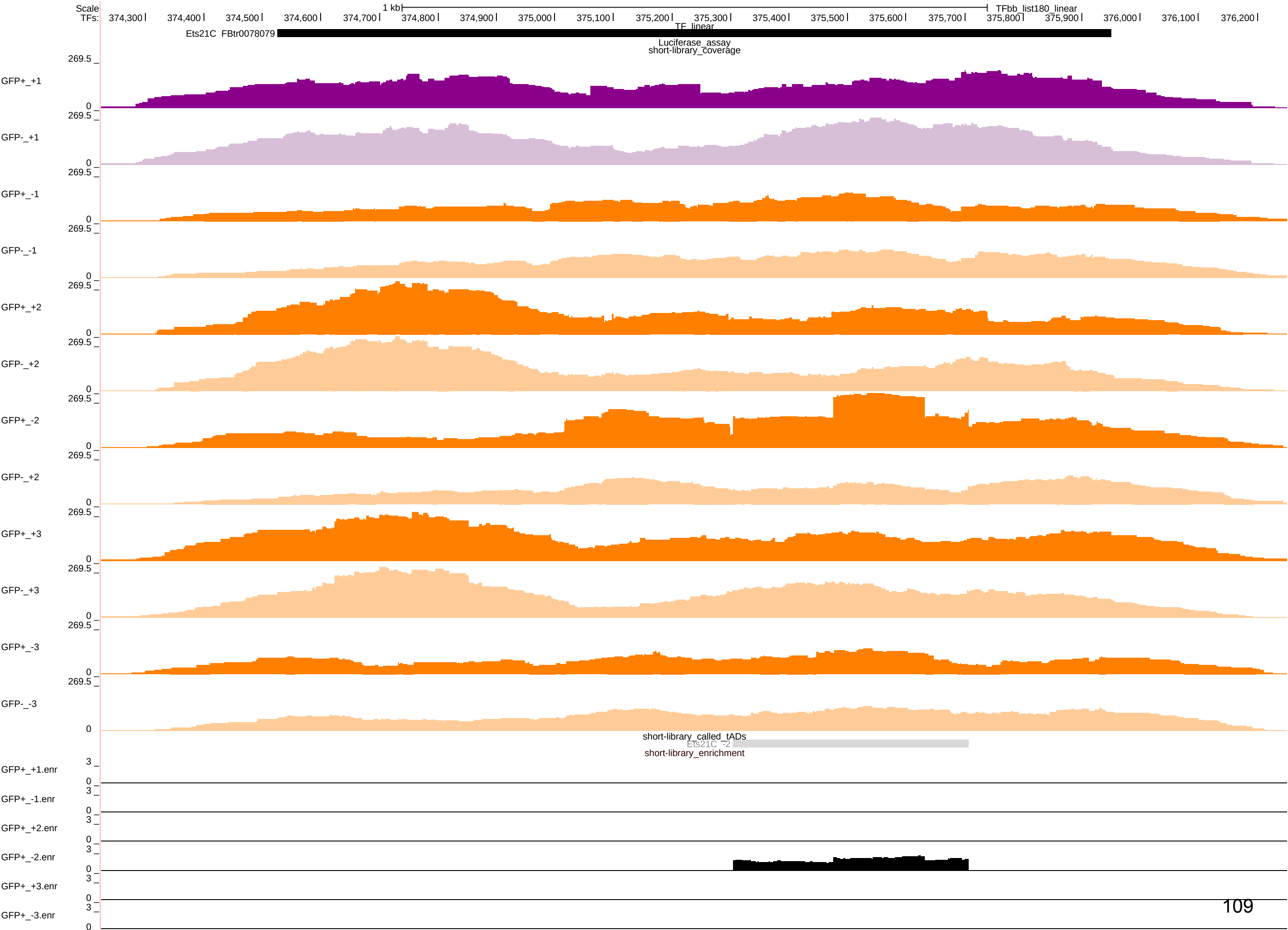
Scale
1 kb
TFbb_list180_linear
TFs:
374,300
374,400
374,500
374,600
374,700
374,800
374,900
375,000
375,100
375,200
375,300
375,400
375,500
375,600
375,700
375,800
375,900
376,000
376,100
376,200
TF_linear
Ets21C_FBtr0078079
Luciferase_assay
short-library_coverage
GFP+_+1
GFP-_+1
GFP+_-1
GFP-_-1
GFP+_+2
GFP-_+2
GFP+_-2
GFP-_+2
GFP+_+3
GFP-_+3
GFP+_-3
GFP-_-3
269.5
0
short-library_called_tADs
Ets21C_-2
short-library_enrichment
GFP+_+1.enr
GFP+_-1.enr
GFP+_+2.enr
GFP+_-2.enr
GFP+_+3.enr
GFP+_-3.enr
3
0

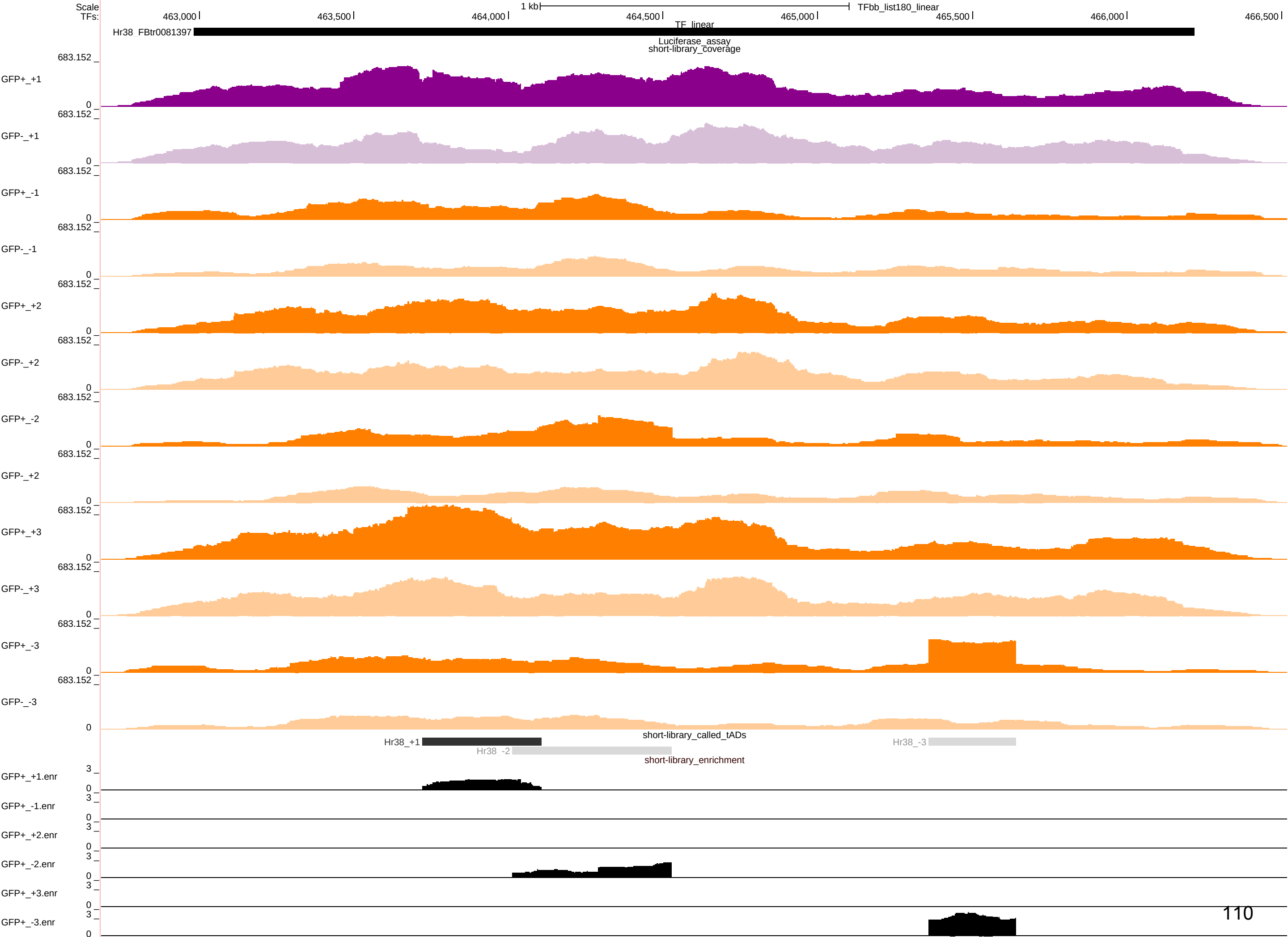
Scale
TFs:
1 kb
TFbb_list180_linear
463,000
463,500
464,000
464,500
465,000
465,500
466,000
466,500
TF_linear
Hr38 FBtr0081397
Luciferase_assay
short-library_coverage
683.152
0
GFP+_+1
GFP-_+1
GFP+_-1
GFP-_-1
GFP+_+2
GFP-_+2
GFP+_-2
GFP-_+2
GFP+_+3
GFP-_+3
GFP+_-3
GFP-_-3
short-library_called_tADs
Hr38_+1
Hr38_-2
Hr38_-3
short-library_enrichment
3
GFP+_+1.enr
GFP+_-1.enr
GFP+_+2.enr
GFP+_-2.enr
GFP+_+3.enr
GFP+_-3.enr

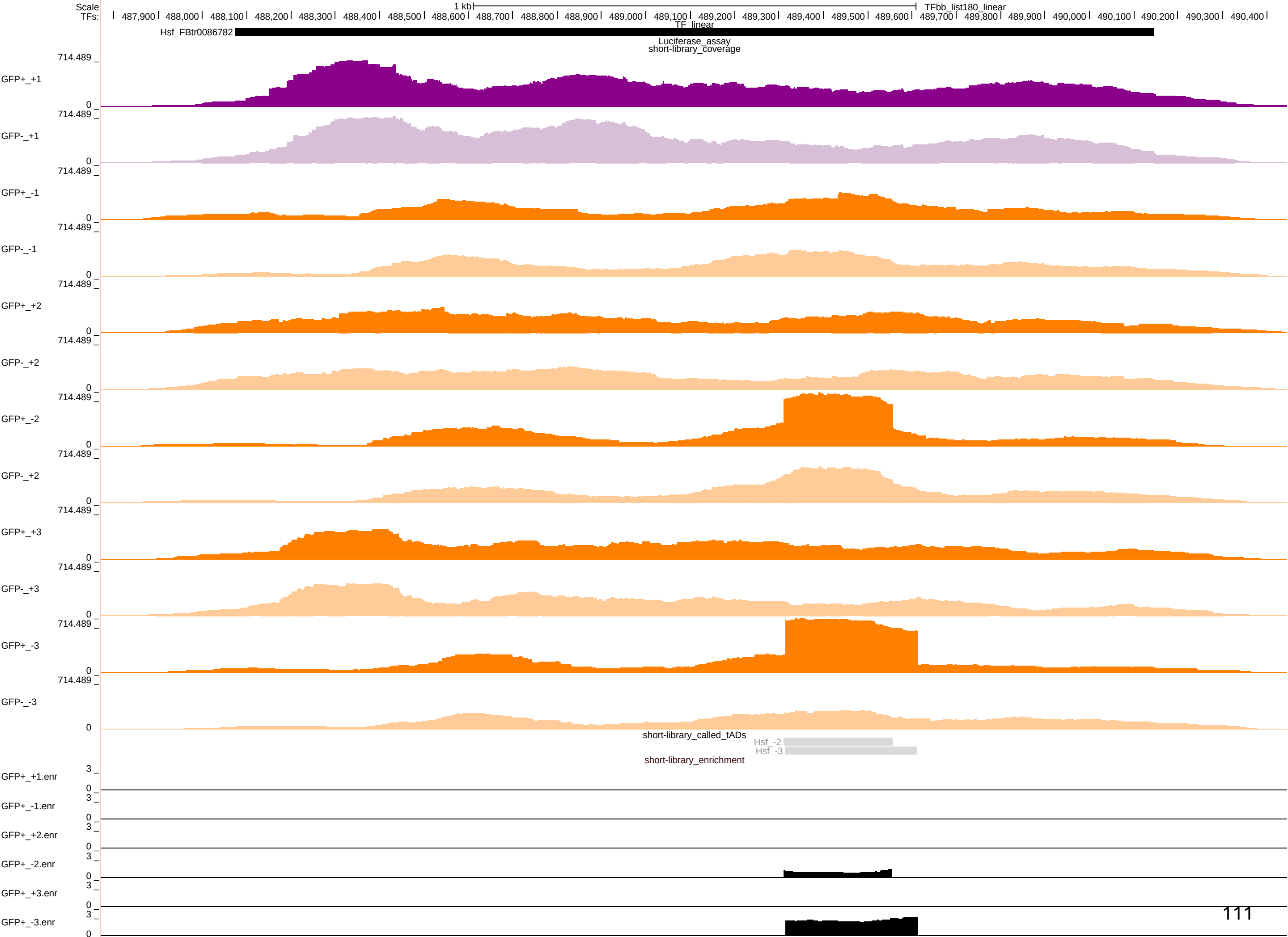
Scale
TFs:
1 kb
TFbb_list180_linear
487,900 488,000 488,100 488,200 488,300 488,400 488,500 488,600 488,700 488,800 488,900 489,000 489,100 489,200 489,300 489,400 489,500 489,600 489,700 489,800 489,900 490,000 490,100 490,200 490,300 490,400
TF_linear
Hsf_FBtr0086782
Luciferase_assay
short-library_coverage
GFP+_+1
GFP-_+1
GFP+_-1
GFP-_-1
GFP+_+2
GFP-_+2
GFP+_-2
GFP-_+2
GFP+_+3
GFP-_+3
GFP+_-3
GFP-_-3
714.489
0
short-library_called_tADs
Hsf_-2
Hsf_-3
short-library_enrichment
GFP+_+1.enr
GFP+_-1.enr
GFP+_+2.enr
GFP+_-2.enr
GFP+_+3.enr
GFP+_-3.enr
3
0

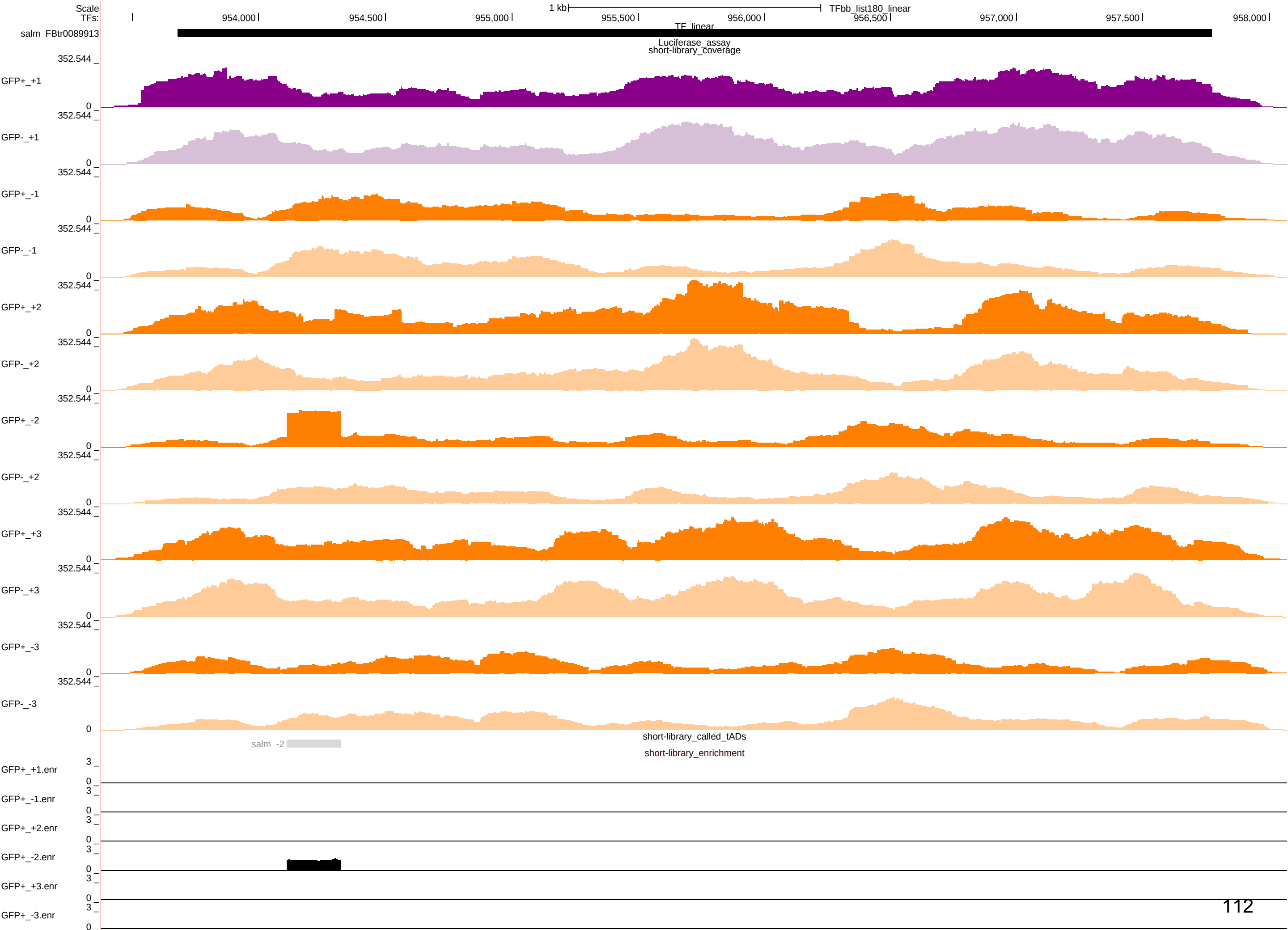

Scale
TFs:
1 kb
954,000
954,500
955,000
955,500
956,000
956,500
957,000
957,500
958,000
TFbb_list180_linear
TF_linear
salm FBtr0089913
Luciferase_assay
short-library_coverage
352.544
0
GFP+_+1
GFP-_+1
GFP+_-1
GFP-_-1
GFP+_+2
GFP-_+2
GFP+_-2
GFP-_+2
GFP+_+3
GFP-_+3
GFP+_-3
GFP-_-3
short-library_called_tADs
salm_-2
short-library_enrichment
3
GFP+_+1.enr
GFP+_-1.enr
GFP+_+2.enr
GFP+_-2.enr
GFP+_+3.enr
GFP+_-3.enr

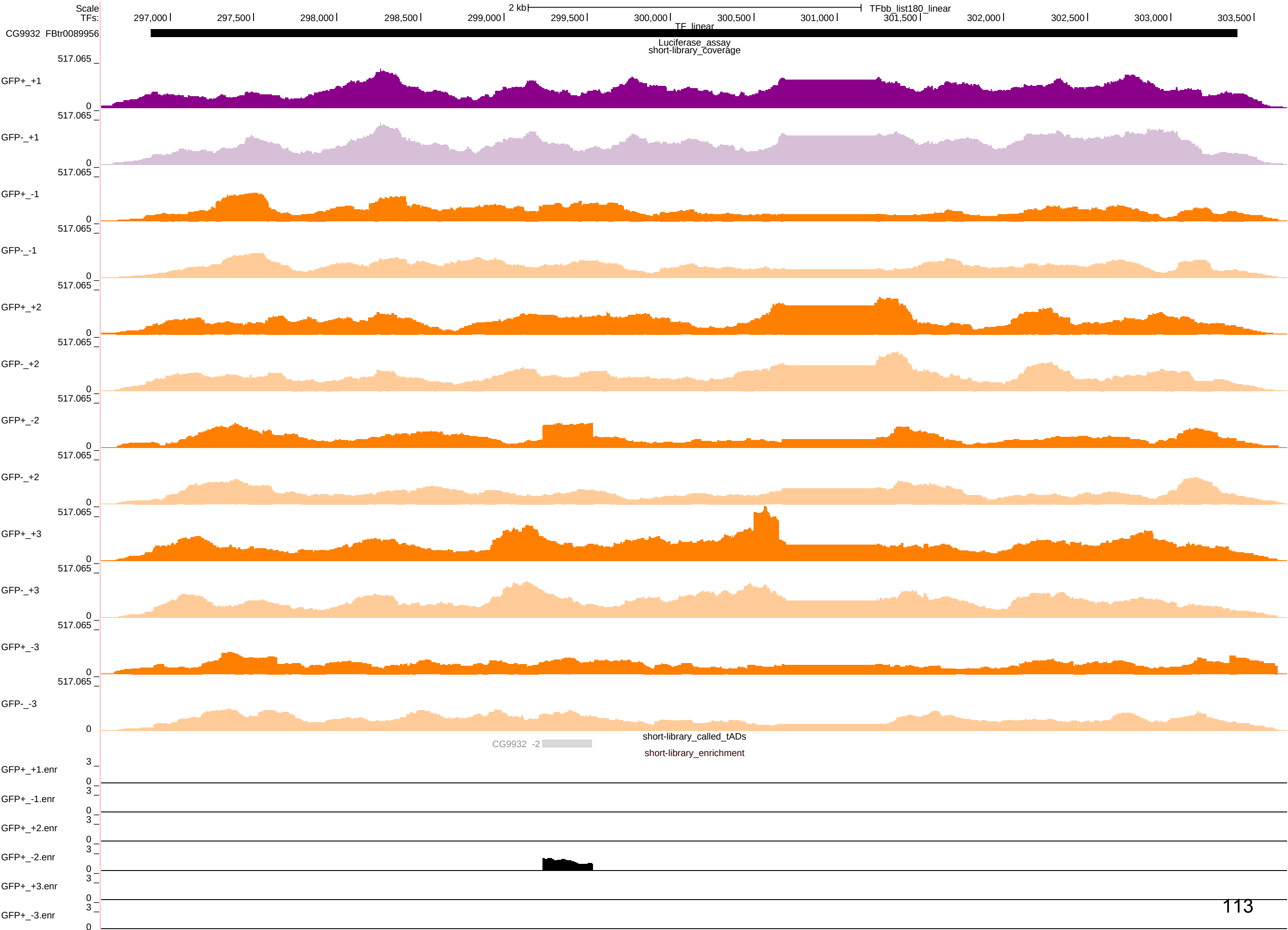

Scale
2 kb
TFbb_list180_linear
TFs:
297,000
297,500
298,000
298,500
299,000
299,500
300,000
300,500
301,000
301,500
302,000
302,500
303,000
303,500
TF_linear
CG9932_FBtr0089956
Luciferase_assay
short-library_coverage
GFP+_+1
GFP-_+1
GFP+_-1
GFP-_-1
GFP+_+2
GFP-_+2
GFP+_-2
GFP-_+2
GFP+_+3
GFP-_+3
GFP+_-3
GFP-_-3
517.065
0
short-library_called_tADs
CG9932_-2
short-library_enrichment
GFP+_+1.enr
GFP+_-1.enr
GFP+_+2.enr
GFP+_-2.enr
GFP+_+3.enr
GFP+_-3.enr
3
0

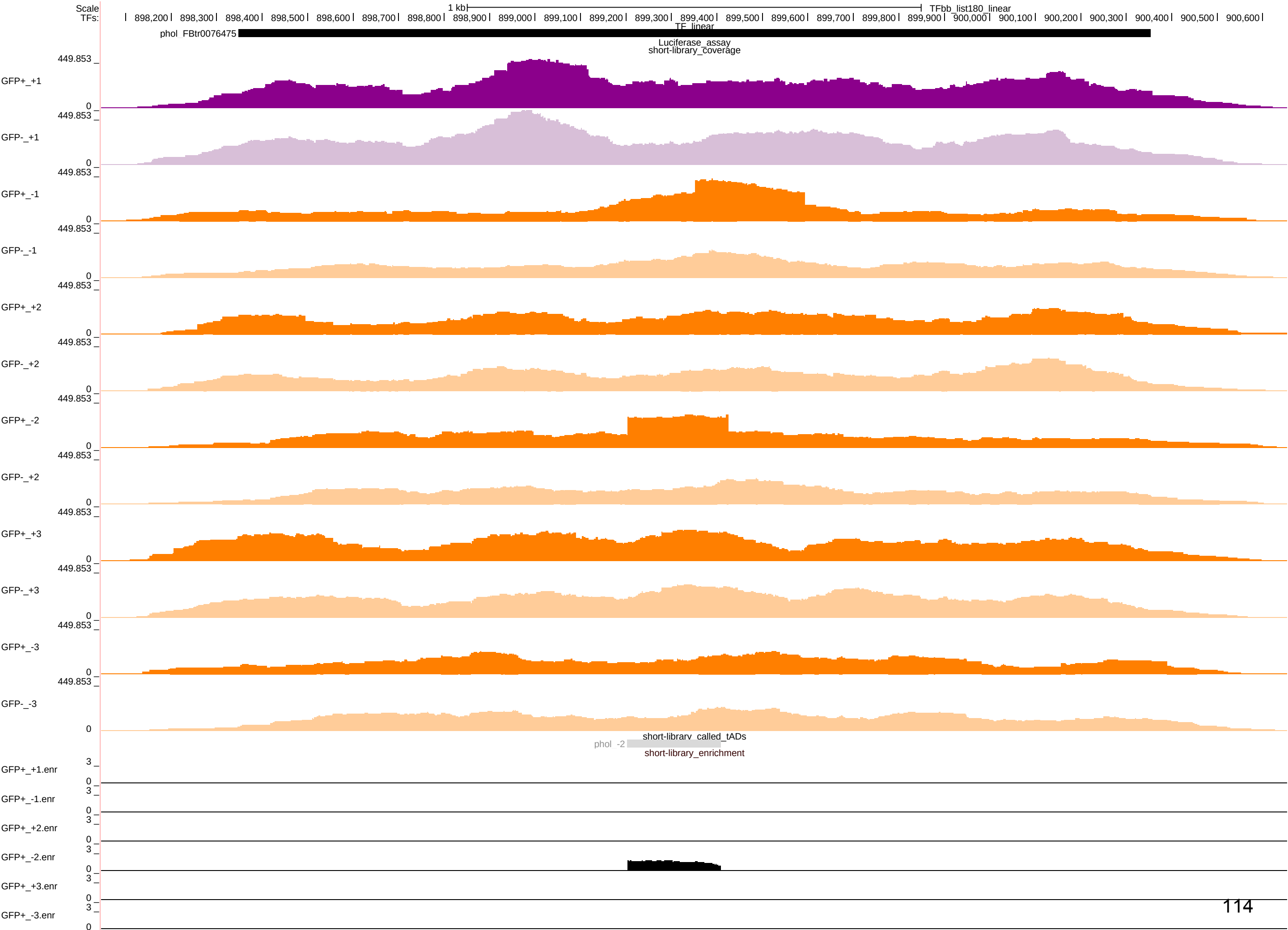
Scale
TFs:
1 kb
TFbb_list180_linear
898,200
898,300
898,400
898,500
898,600
898,700
898,800
898,900
899,000
899,100
899,200
899,300
899,400
899,500
899,600
899,700
899,800
899,900
900,000
900,100
900,200
900,300
900,400
900,500
900,600
TF_linear
phol_FBtr0076475
Luciferase_assay
short-library_coverage
GFP+_+1
GFP-_+1
GFP+_-1
GFP-_-1
GFP+_+2
GFP-_+2
GFP+_-2
GFP-_+2
GFP+_+3
GFP-_+3
GFP+_-3
GFP-_-3
449.853
0
short-library_called_tADs
phol_-2
short-library_enrichment
GFP+_+1.enr
GFP+_-1.enr
GFP+_+2.enr
GFP+_-2.enr
GFP+_+3.enr
GFP+_-3.enr
3
0

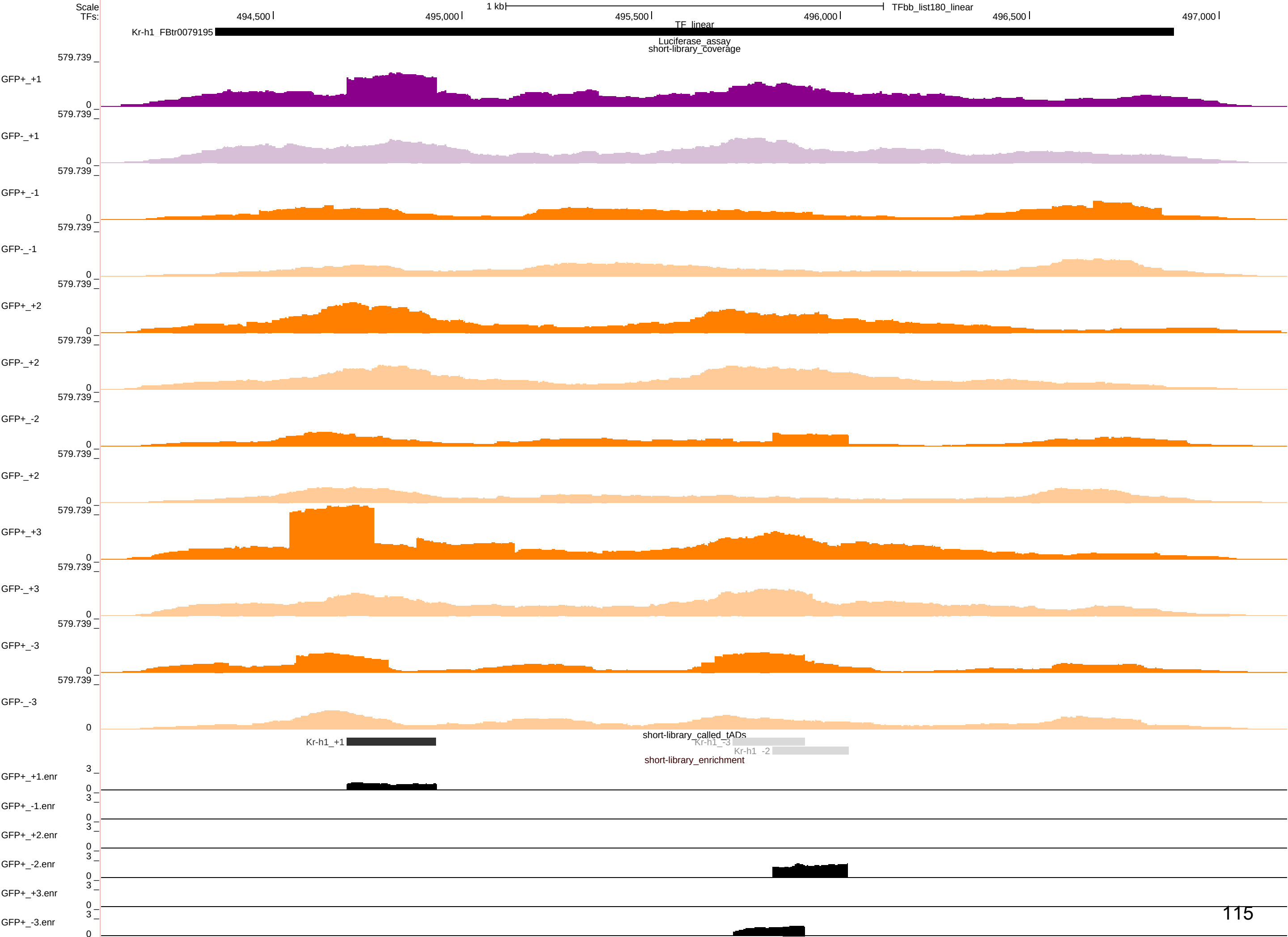

Scale
TFs:
1 kb
TFbb_list180_linear
494,500
495,000
495,500
496,000
496,500
497,000
TF_linear
Kr-h1 FBtr0079195
Luciferase_assay
short-library_coverage
579.739
GFP+_+1
0
GFP-_+1
GFP+_-1
GFP-_-1
GFP+_+2
GFP-_+2
GFP+_-2
GFP-_+2
GFP+_+3
GFP-_+3
GFP+_-3
GFP-_-3
short-library_called_tADs
Kr-h1_+1
Kr-h1_-3
Kr-h1_-2
short-library_enrichment
3
GFP+_+1.enr
GFP+_-1.enr
GFP+_+2.enr
GFP+_-2.enr
GFP+_+3.enr
GFP+_-3.enr

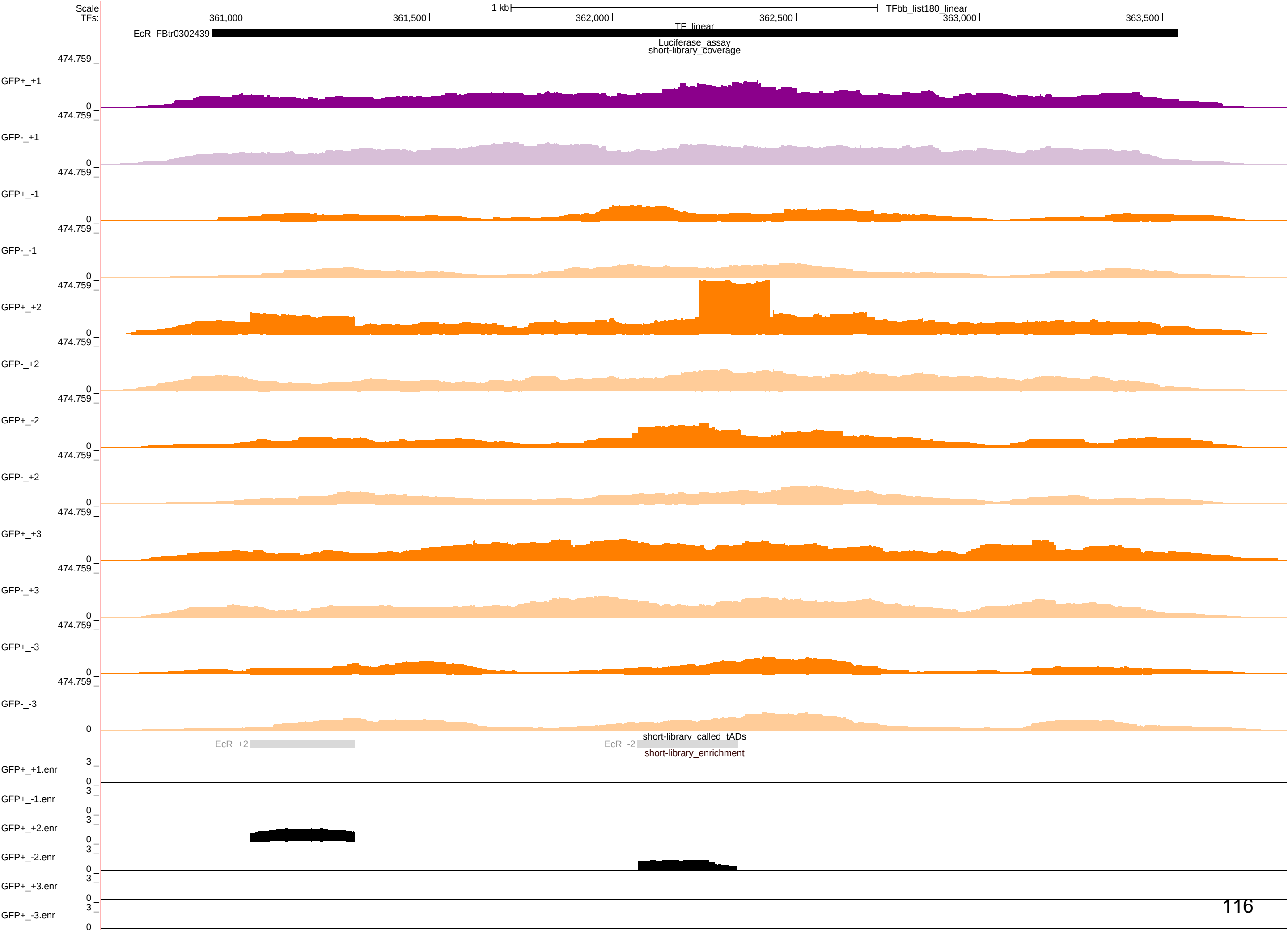

Scale
TFs:
1 kb
TFbb_list180_linear
361,000
361,500
362,000
362,500
363,000
363,500
TF_linear
EcR_FBtr0302439
Luciferase_assay
short-library_coverage
474.759
0
GFP+_+1
GFP-_+1
GFP+_-1
GFP-_-1
GFP+_+2
GFP-_+2
GFP+_-2
GFP-_+2
GFP+_+3
GFP-_+3
GFP+_-3
GFP-_-3
short-library_called_tADs
EcR_+2
EcR_-2
short-library_enrichment
3
GFP+_+1.enr
GFP+_-1.enr
GFP+_+2.enr
GFP+_-2.enr
GFP+_+3.enr
GFP+_-3.enr

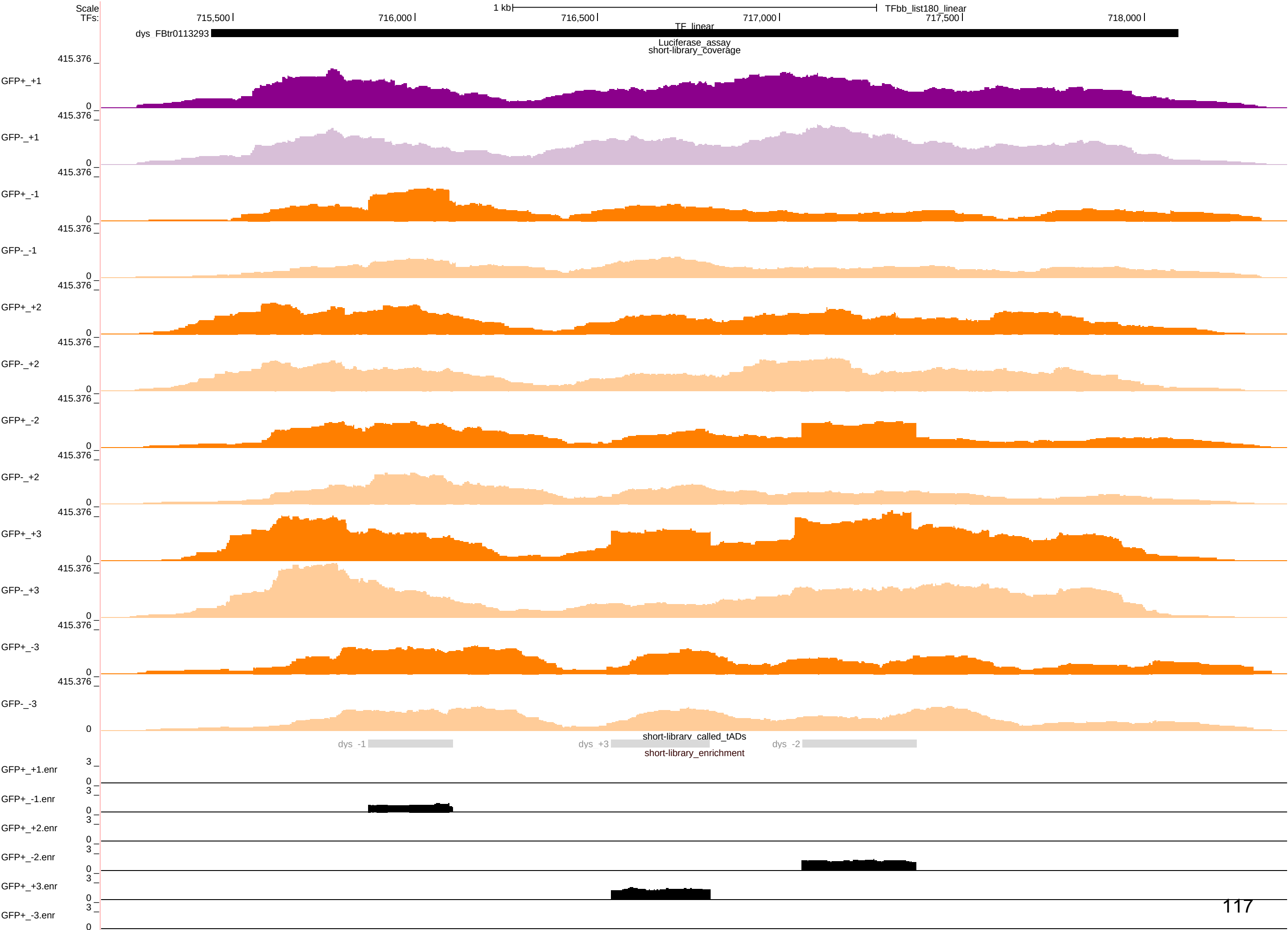
Scale
TFs:
1 kb
TFbb_list180_linear
715,500
716,000
716,500
717,000
717,500
718,000
dys FBtr0113293
TF_linear
Luciferase_assay
short-library_coverage
415.376
0
GFP+_+1
GFP-_+1
GFP+_-1
GFP-_-1
GFP+_+2
GFP-_+2
GFP+_-2
GFP-_+2
GFP+_+3
GFP-_+3
GFP+_-3
GFP-_-3
short-library_called_tADs
dys_-1
dys_+3
dys_-2
short-library_enrichment
3
GFP+_+1.enr
GFP+_-1.enr
GFP+_+2.enr
GFP+_-2.enr
GFP+_+3.enr
GFP+_-3.enr

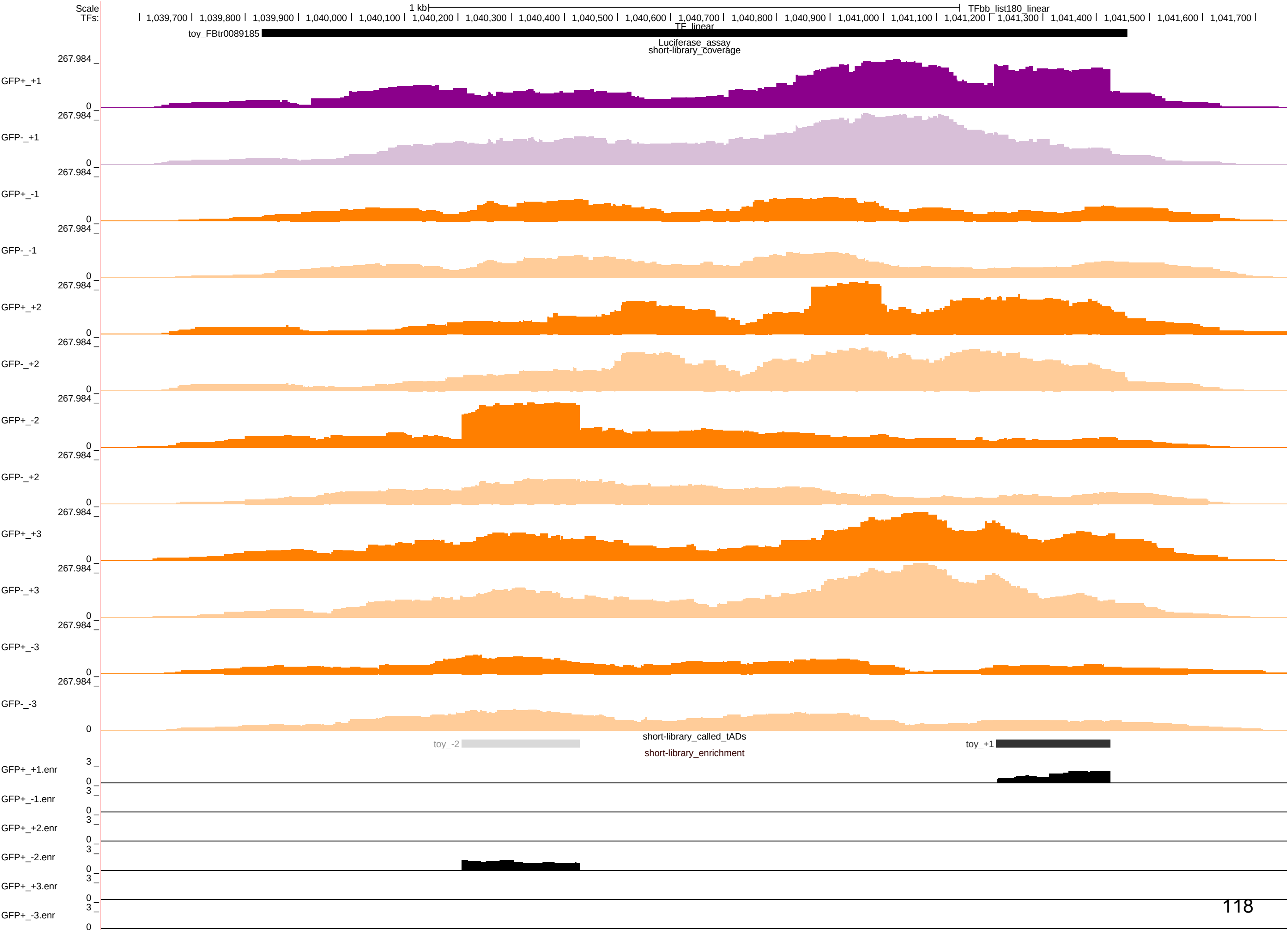
Scale
TFs:
1 kb
TFbb_list180_linear
1,039,700
1,039,800
1,039,900
1,040,000
1,040,100
1,040,200
1,040,300
1,040,400
1,040,500
1,040,600
1,040,700
1,040,800
1,040,900
1,041,000
1,041,100
1,041,200
1,041,300
1,041,400
1,041,500
1,041,600
1,041,700
TF_linear
toy_FBtr0089185
Luciferase_assay
short-library_coverage
267.984
0
GFP+_+1
GFP-_+1
GFP+_-1
GFP-_-1
GFP+_+2
GFP-_+2
GFP+_-2
GFP-_+2
GFP+_+3
GFP-_+3
GFP+_-3
GFP-_-3
short-library_called_tADs
toy_-2
toy_+1
short-library_enrichment
3
GFP+_+1.enr
GFP+_-1.enr
GFP+_+2.enr
GFP+_-2.enr
GFP+_+3.enr
GFP+_-3.enr

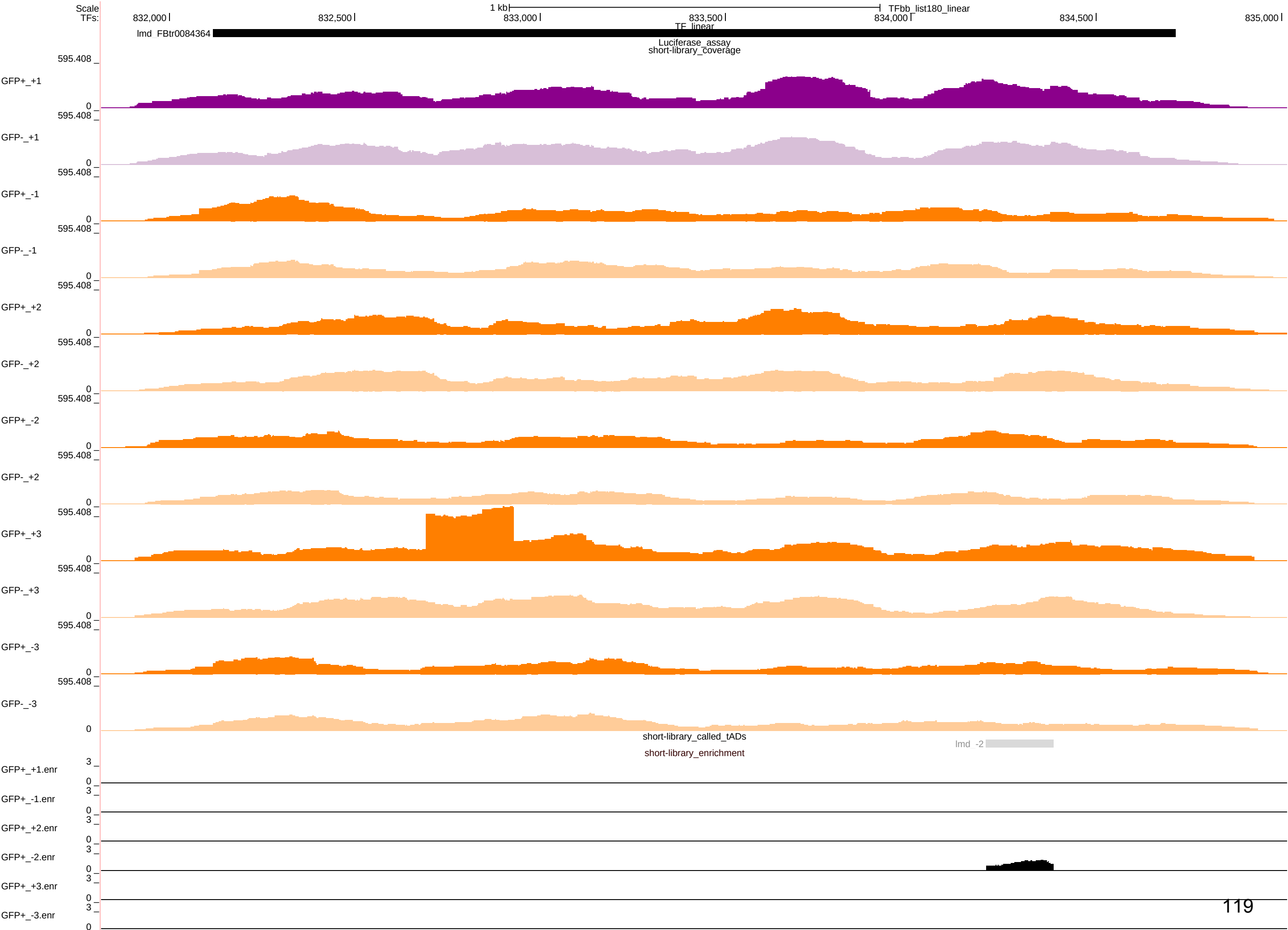
Scale
TFs:
1 kb
TFbb_list180_linear
832,000
832,500
833,000
833,500
834,000
834,500
835,000
TF_linear
lmd_FBtr0084364
Luciferase_assay
short-library_coverage
595.408
0
GFP+_+1
GFP-_+1
GFP+_-1
GFP-_-1
GFP+_+2
GFP-_+2
GFP+_-2
GFP-_+2
GFP+_+3
GFP-_+3
GFP+_-3
GFP-_-3
short-library_called_tADs
lmd_-2
short-library_enrichment
3
GFP+_+1.enr
GFP+_-1.enr
GFP+_+2.enr
GFP+_-2.enr
GFP+_+3.enr
GFP+_-3.enr

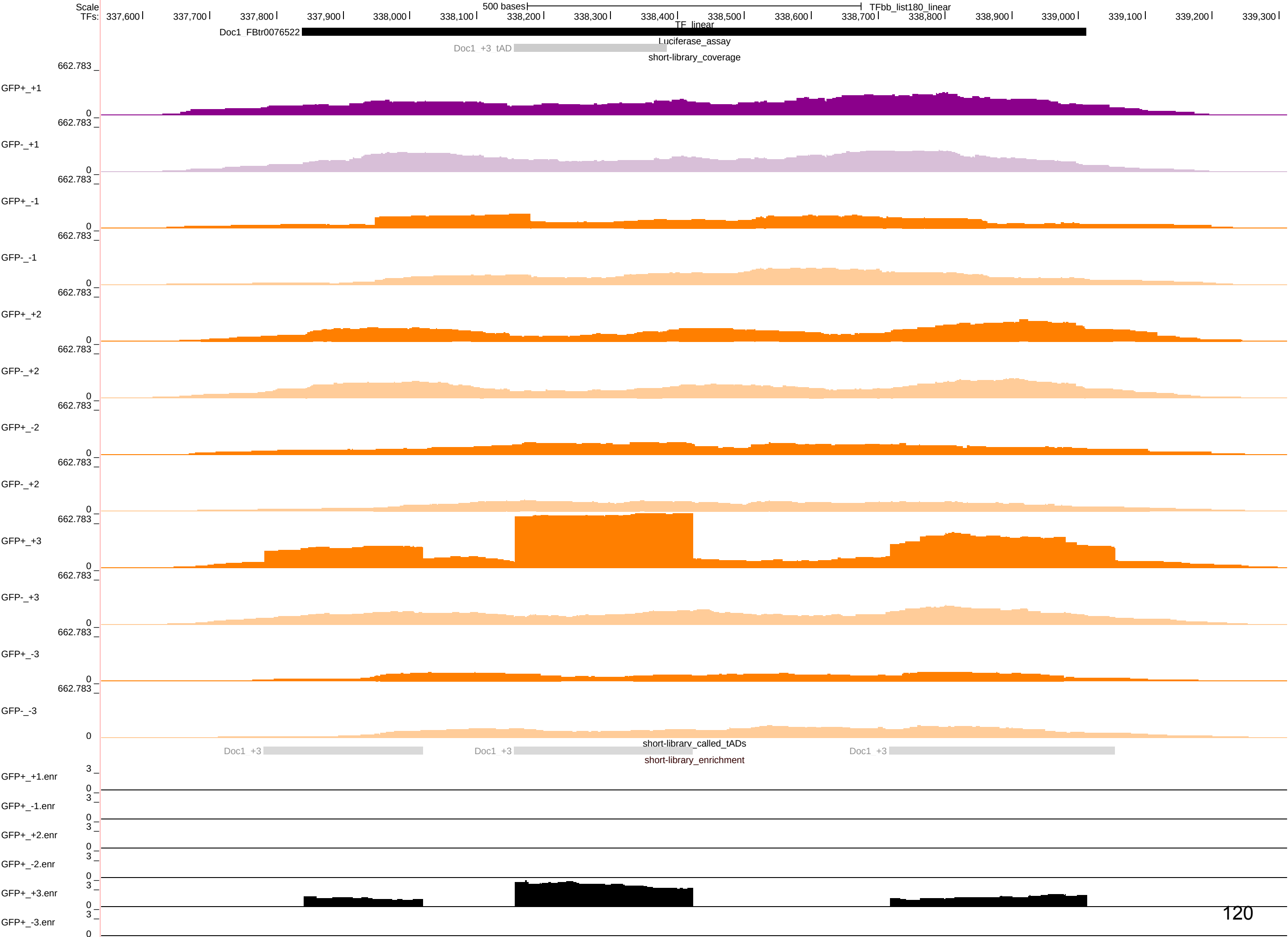

Scale
500 bases
TFs: 337,600 337,700 337,800 337,900 338,000 338,100 338,200 338,300 338,400 338,500 338,600 338,700 338,800 338,900 339,000 339,100 339,200 339,300
TFbb_list180_linear
TF_linear
Doc1_FBtr0076522
Luciferase_assay
Doc1_+3_tAD
short-library_coverage
662.783
0
GFP+_+1
GFP-_+1
GFP+_-1
GFP-_-1
GFP+_+2
GFP-_+2
GFP+_-2
GFP-_+2
GFP+_+3
GFP-_+3
GFP+_-3
GFP-_-3
short-library_called_tADs
Doc1_+3
short-library_enrichment
3
GFP+_+1.enr
GFP+_-1.enr
GFP+_+2.enr
GFP+_-2.enr
GFP+_+3.enr
GFP+_-3.enr

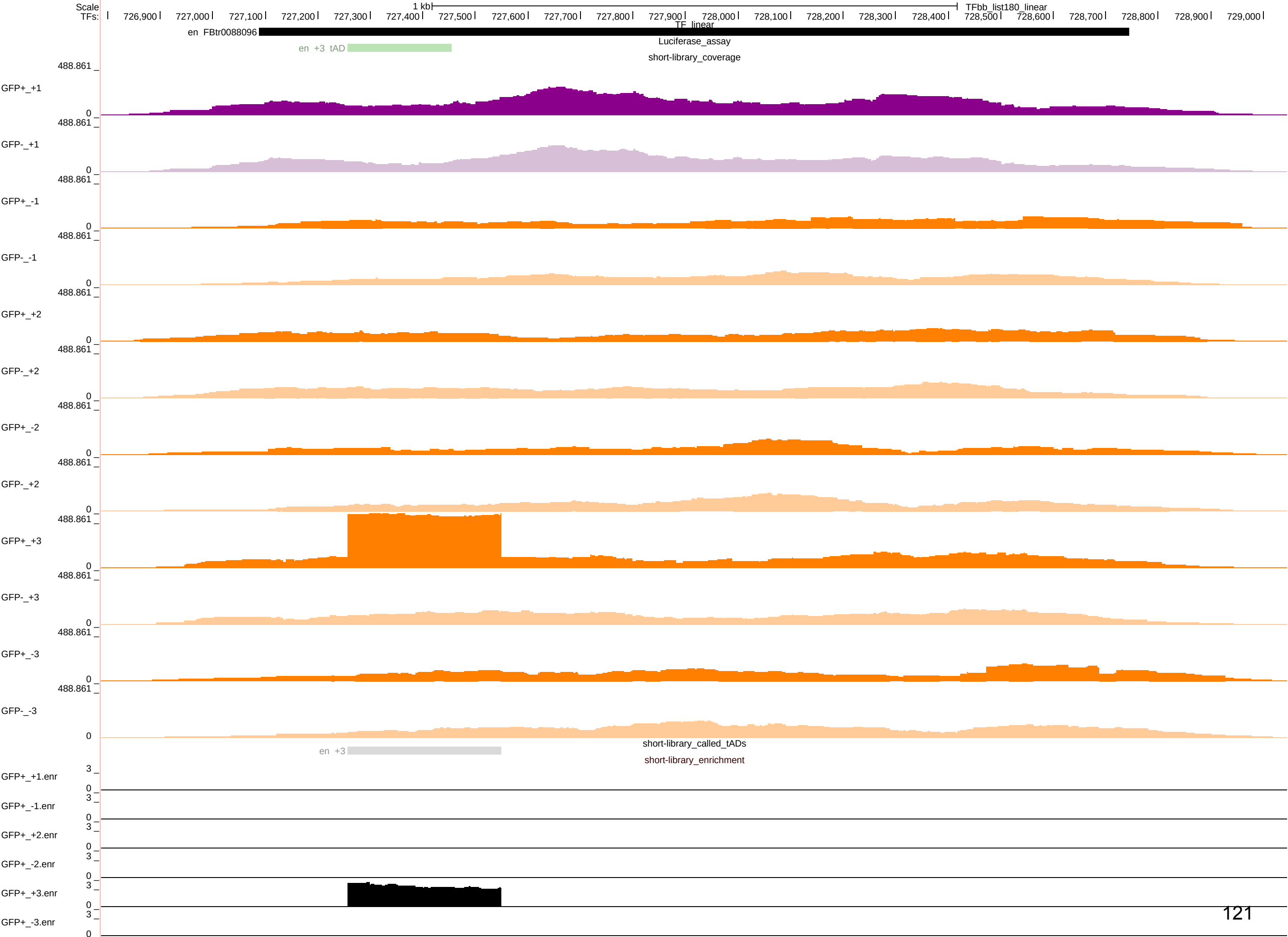

Scale
1 kb
TFs:
726,900
727,000
727,100
727,200
727,300
727,400
727,500
727,600
727,700
727,800
727,900
728,000
728,100
728,200
728,300
728,400
728,500
728,600
728,700
728,800
728,900
729,000
TFbb_list180_linear
TF_linear
en FBtr0088096
Luciferase_assay
en +3 tAD
short-library_coverage
488.861
0
GFP+_+1
GFP-_+1
GFP+_-1
GFP-_-1
GFP+_+2
GFP-_+2
GFP+_-2
GFP-_+2
GFP+_+3
GFP-_+3
GFP+_-3
GFP-_-3
short-library_called_tADs
en +3
short-library_enrichment
3
GFP+_+1.enr
GFP+_-1.enr
GFP+_+2.enr
GFP+_-2.enr
GFP+_+3.enr
GFP+_-3.enr

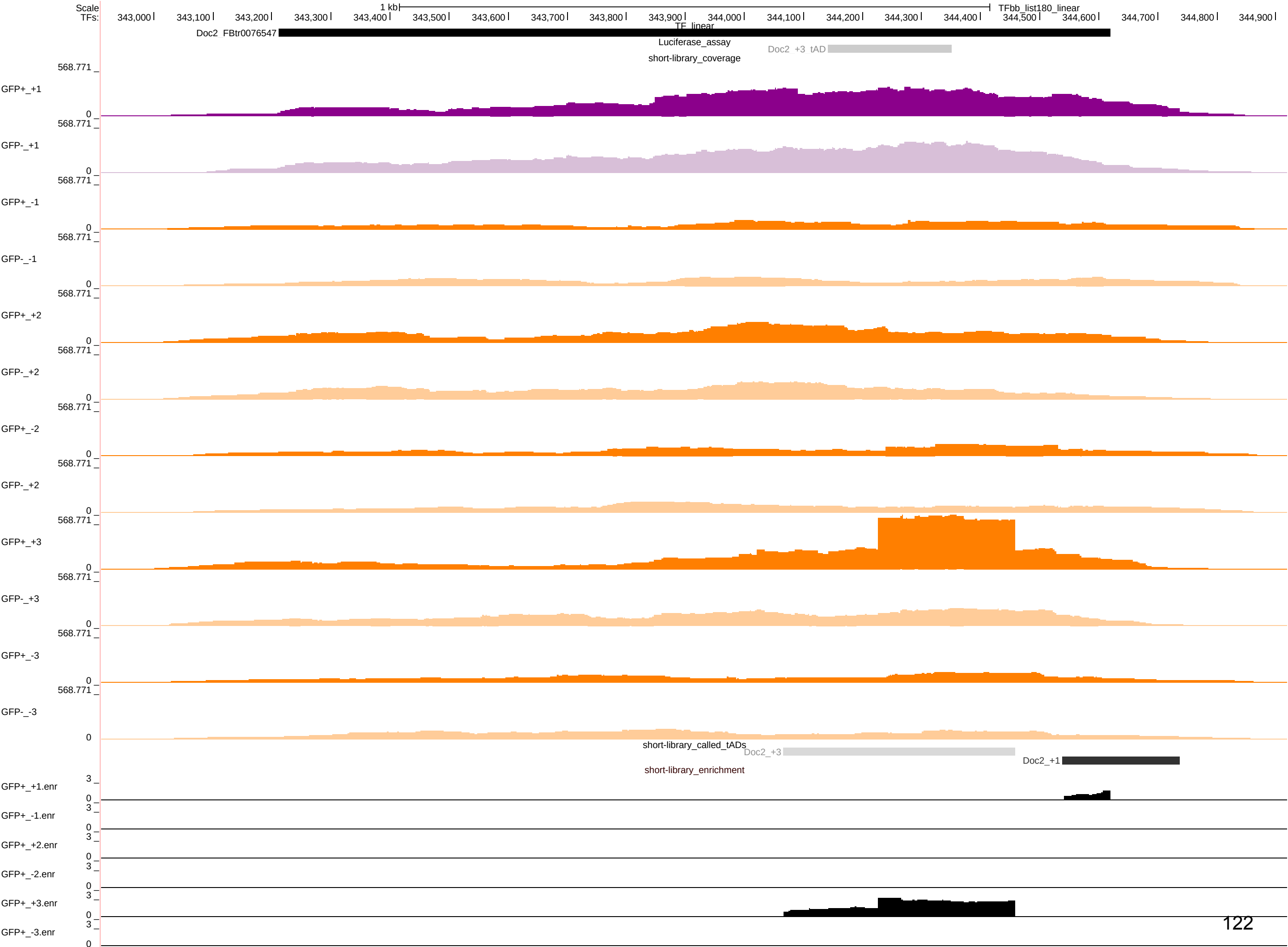
Scale
TFs:
1 kb
343,000
343,100
343,200
343,300
343,400
343,500
343,600
343,700
343,800
343,900
344,000
344,100
344,200
344,300
344,400
344,500
344,600
344,700
344,800
344,900
TFbb_list180_linear
TF_linear
Doc2 FBtr0076547
Luciferase_assay
Doc2 +3 tAD
short-library_coverage
568.771
0
GFP+_+1
GFP-_+1
GFP+_-1
GFP-_-1
GFP+_+2
GFP-_+2
GFP+_-2
GFP-_+2
GFP+_+3
GFP-_+3
GFP+_-3
GFP-_-3
short-library_called_tADs
Doc2_+3
Doc2_+1
short-library_enrichment
3
GFP+_+1.enr
GFP+_-1.enr
GFP+_+2.enr
GFP+_-2.enr
GFP+_+3.enr
GFP+_-3.enr

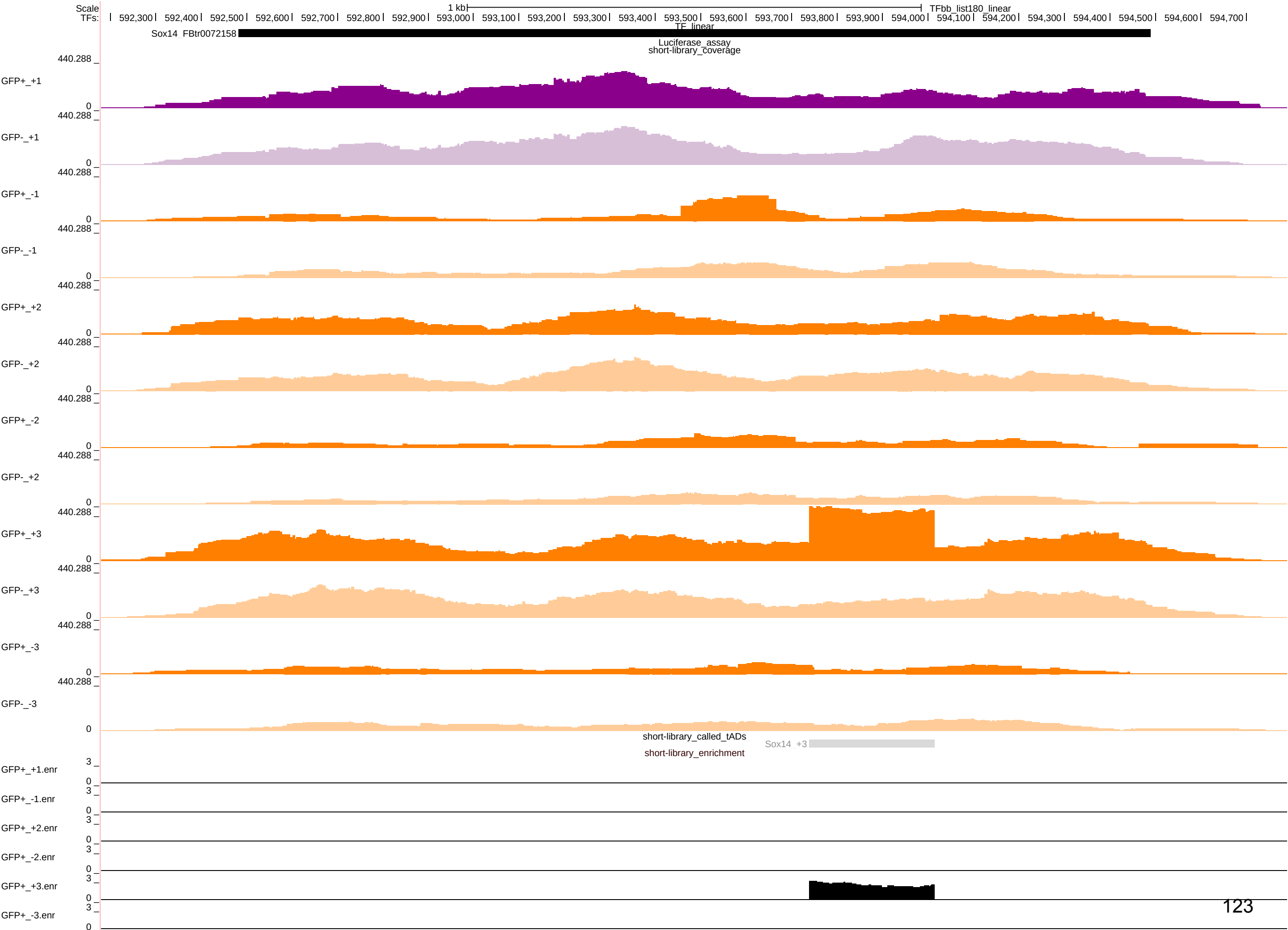
Scale
1 kb
TFbb_list180_linear
TFs:
592,300
592,400
592,500
592,600
592,700
592,800
592,900
593,000
593,100
593,200
593,300
593,400
593,500
593,600
593,700
593,800
593,900
594,000
594,100
594,200
594,300
594,400
594,500
594,600
594,700
TF_linear
Sox14 FBtr0072158
Luciferase_assay
short-library_coverage
440.288
0
GFP+_+1
GFP-_+1
GFP+_-1
GFP-_-1
GFP+_+2
GFP-_+2
GFP+_-2
GFP-_+2
GFP+_+3
GFP-_+3
GFP+_-3
GFP-_-3
short-library_called_tADs
Sox14 +3
short-library_enrichment
3
GFP+_+1.enr
GFP+_-1.enr
GFP+_+2.enr
GFP+_-2.enr
GFP+_+3.enr
GFP+_-3.enr

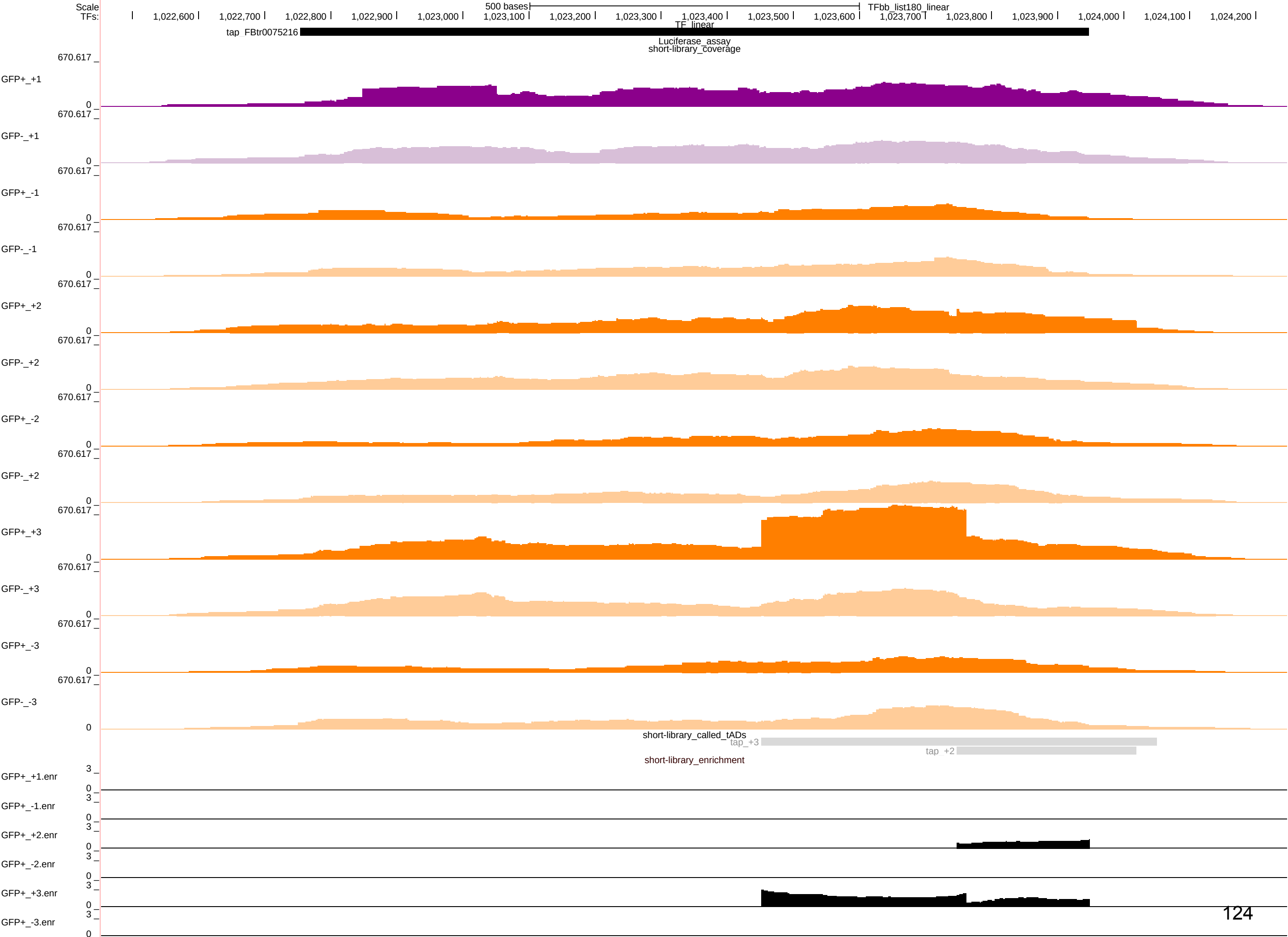
Scale
500 bases
TFs:
1,022,600
1,022,700
1,022,800
1,022,900
1,023,000
1,023,100
1,023,200
1,023,300
1,023,400
1,023,500
1,023,600
1,023,700
1,023,800
1,023,900
1,024,000
1,024,100
1,024,200
TFbb_list180_linear
TF_linear
tap FBtr0075216
Luciferase_assay
short-library_coverage
GFP+_+1
GFP-_+1
GFP+_-1
GFP-_-1
GFP+_+2
GFP-_+2
GFP+_-2
GFP-_+2
GFP+_+3
GFP-_+3
GFP+_-3
GFP-_-3
670.617
0
short-library_called_tADs
tap_+3
tap_+2
short-library_enrichment
GFP+_+1.enr
GFP+_-1.enr
GFP+_+2.enr
GFP+_-2.enr
GFP+_+3.enr
GFP+_-3.enr
3
0

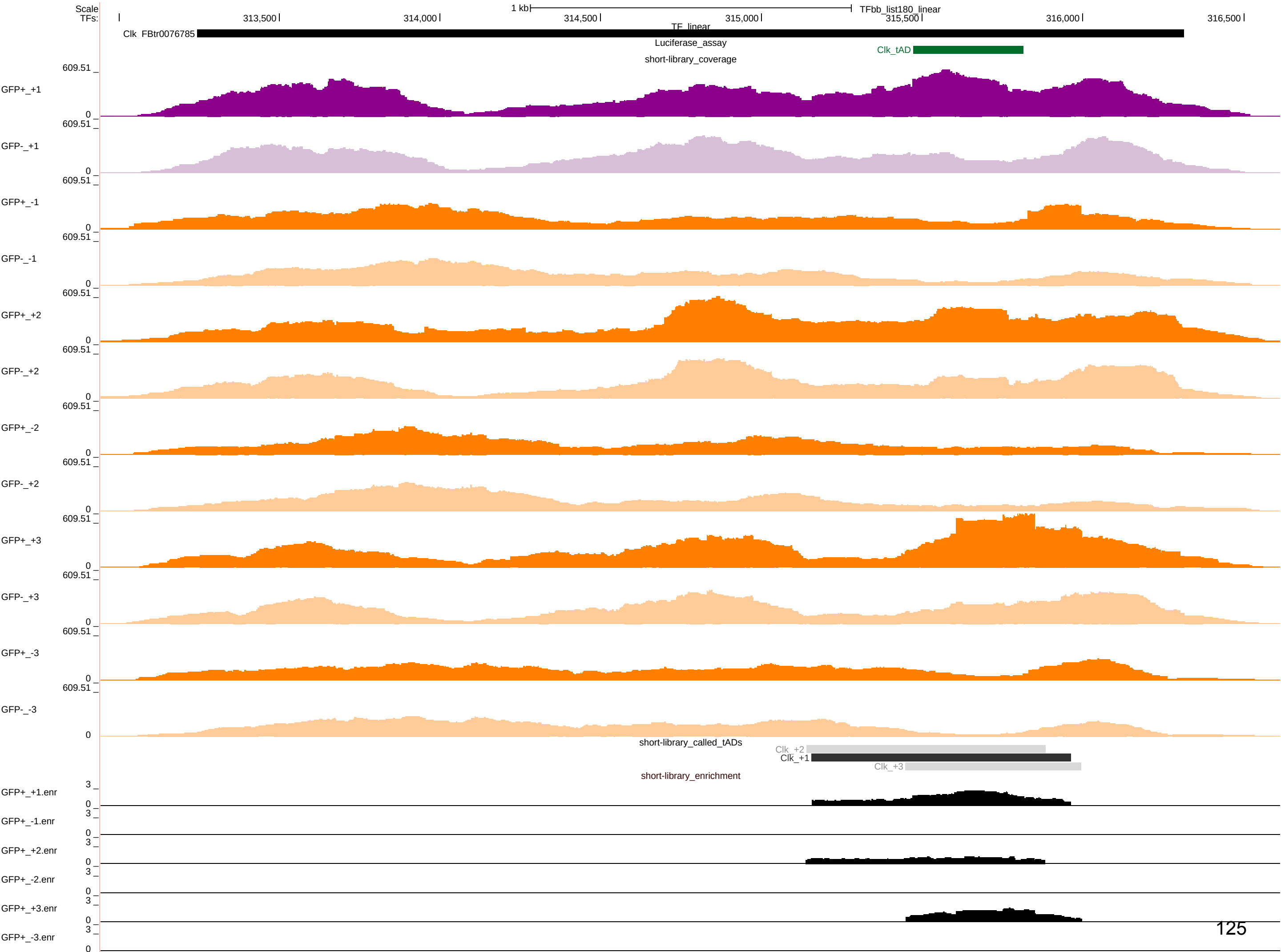

Scale
TFs:
1 kb
TFbb_list180_linear
313,500
314,000
314,500
315,000
315,500
316,000
316,500
Clk FBtr0076785
TF_linear
Luciferase_assay
Clk_tAD
short-library_coverage
GFP+_+1
GFP-_+1
GFP+_-1
GFP-_-1
GFP+_+2
GFP-_+2
GFP+_-2
GFP-_+2
GFP+_+3
GFP-_+3
GFP+_-3
GFP-_-3
609.51
0
short-library_called_tADs
Clk_+2
Clk_+1
Clk_+3
short-library_enrichment
GFP+_+1.enr
GFP+_-1.enr
GFP+_+2.enr
GFP+_-2.enr
GFP+_+3.enr
GFP+_-3.enr
3
0

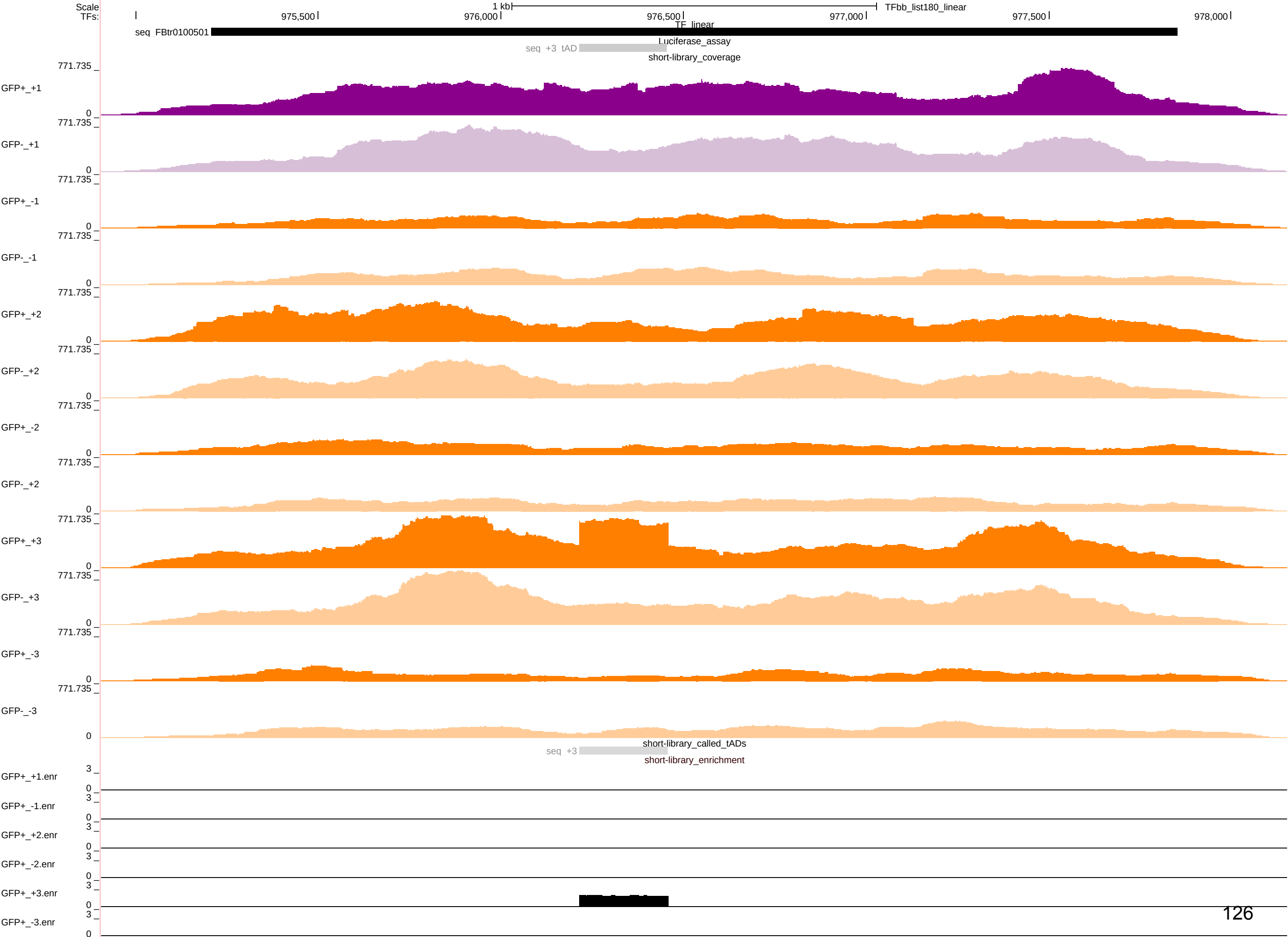
Scale
TFs:
1 kb
975,500
976,000
976,500
977,000
977,500
978,000
TFbb_list180_linear
TF_linear
seq FBtr0100501
Luciferase_assay
seq +3 tAD
short-library_coverage
771.735
0
GFP+_+1
GFP-_+1
GFP+_-1
GFP-_-1
GFP+_+2
GFP-_+2
GFP+_-2
GFP-_+2
GFP+_+3
GFP-_+3
GFP+_-3
GFP-_-3
short-library_called_tADs
seq +3
short-library_enrichment
3
GFP+_+1.enr
GFP+_-1.enr
GFP+_+2.enr
GFP+_-2.enr
GFP+_+3.enr
GFP+_-3.enr

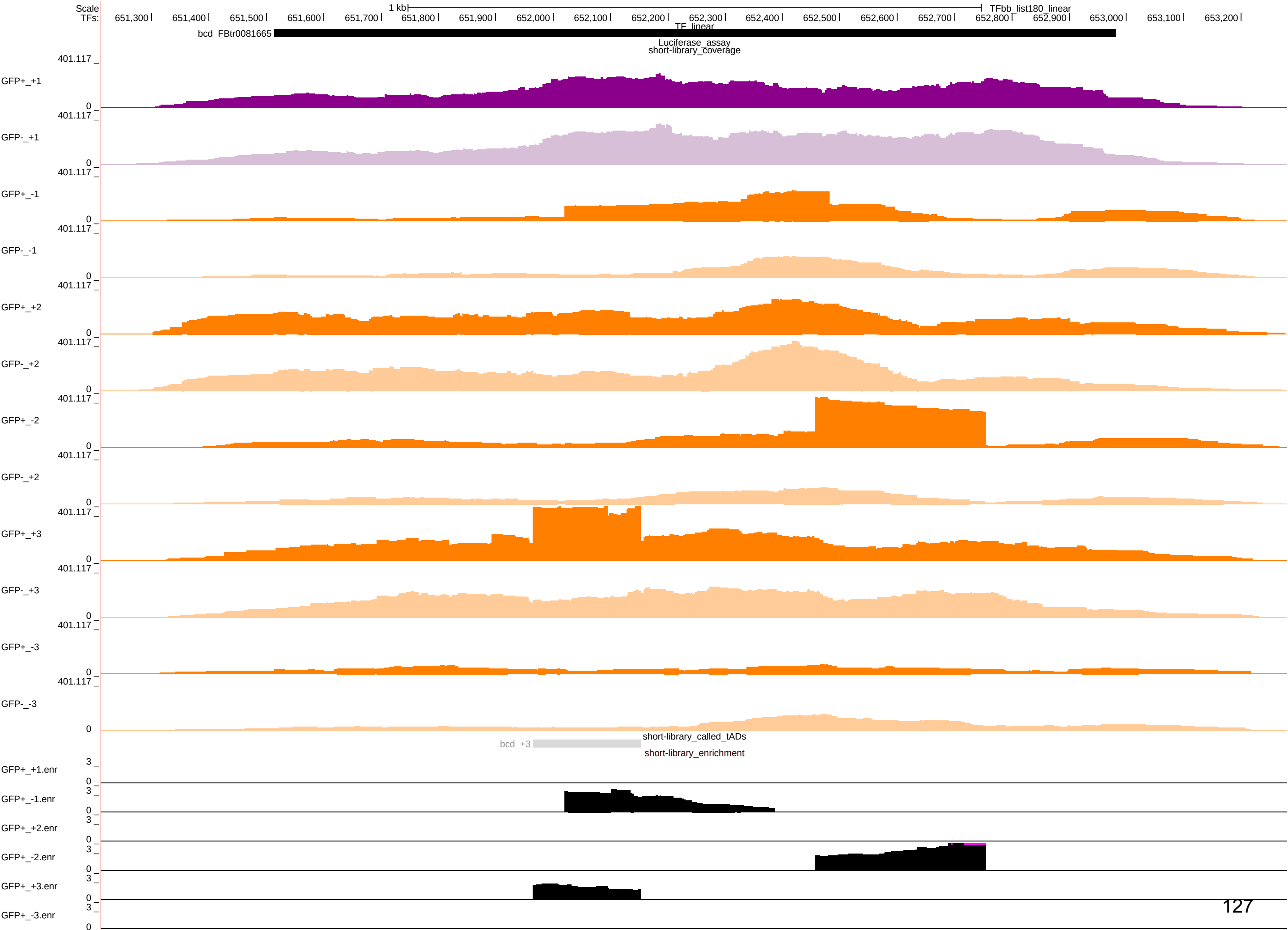

Scale
1 kb
TFbb_list180_linear
TFs:
651,300
651,400
651,500
651,600
651,700
651,800
651,900
652,000
652,100
652,200
652,300
652,400
652,500
652,600
652,700
652,800
652,900
653,000
653,100
653,200
TF_linear
bcd FBtr0081665
Luciferase_assay
short-library_coverage
401.117
0
GFP+_+1
GFP-_+1
GFP+_-1
GFP-_-1
GFP+_+2
GFP-_+2
GFP+_-2
GFP-_+2
GFP+_+3
GFP-_+3
GFP+_-3
GFP-_-3
bcd +3
short-library_called_tADs
short-library_enrichment
3
GFP+_+1.enr
GFP+_-1.enr
GFP+_+2.enr
GFP+_-2.enr
GFP+_+3.enr
GFP+_-3.enr

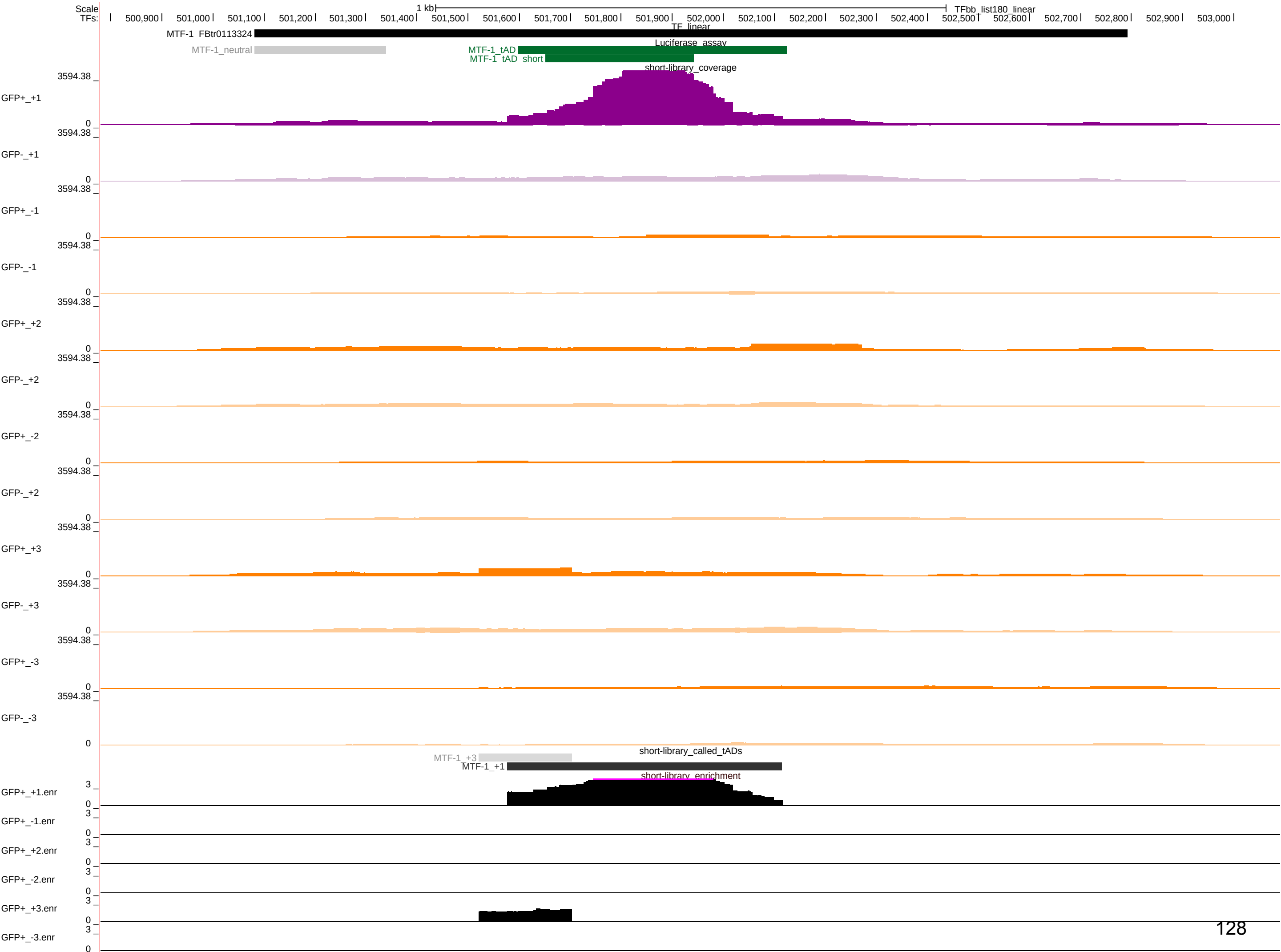
Scale
TFs:
1 kb
TFbb_list180_linear
500,900
501,000
501,100
501,200
501,300
501,400
501,500
501,600
501,700
501,800
501,900
502,000
502,100
502,200
502,300
502,400
502,500
502,600
502,700
502,800
502,900
503,000
TF_linear
MTF-1_FBtr0113324
Luciferase_assay
MTF-1_neutral
MTF-1_tAD
MTF-1_tAD_short
short-library_coverage
GFP+_+1
GFP-_+1
GFP+_-1
GFP-_-1
GFP+_+2
GFP-_+2
GFP+_-2
GFP-_+2
GFP+_+3
GFP-_+3
GFP+_-3
GFP-_-3
3594.38
0
short-library_called_tADs
MTF-1_+3
MTF-1_+1
short-library_enrichment
GFP+_+1.enr
GFP+_-1.enr
GFP+_+2.enr
GFP+_-2.enr
GFP+_+3.enr
GFP+_-3.enr
3
0

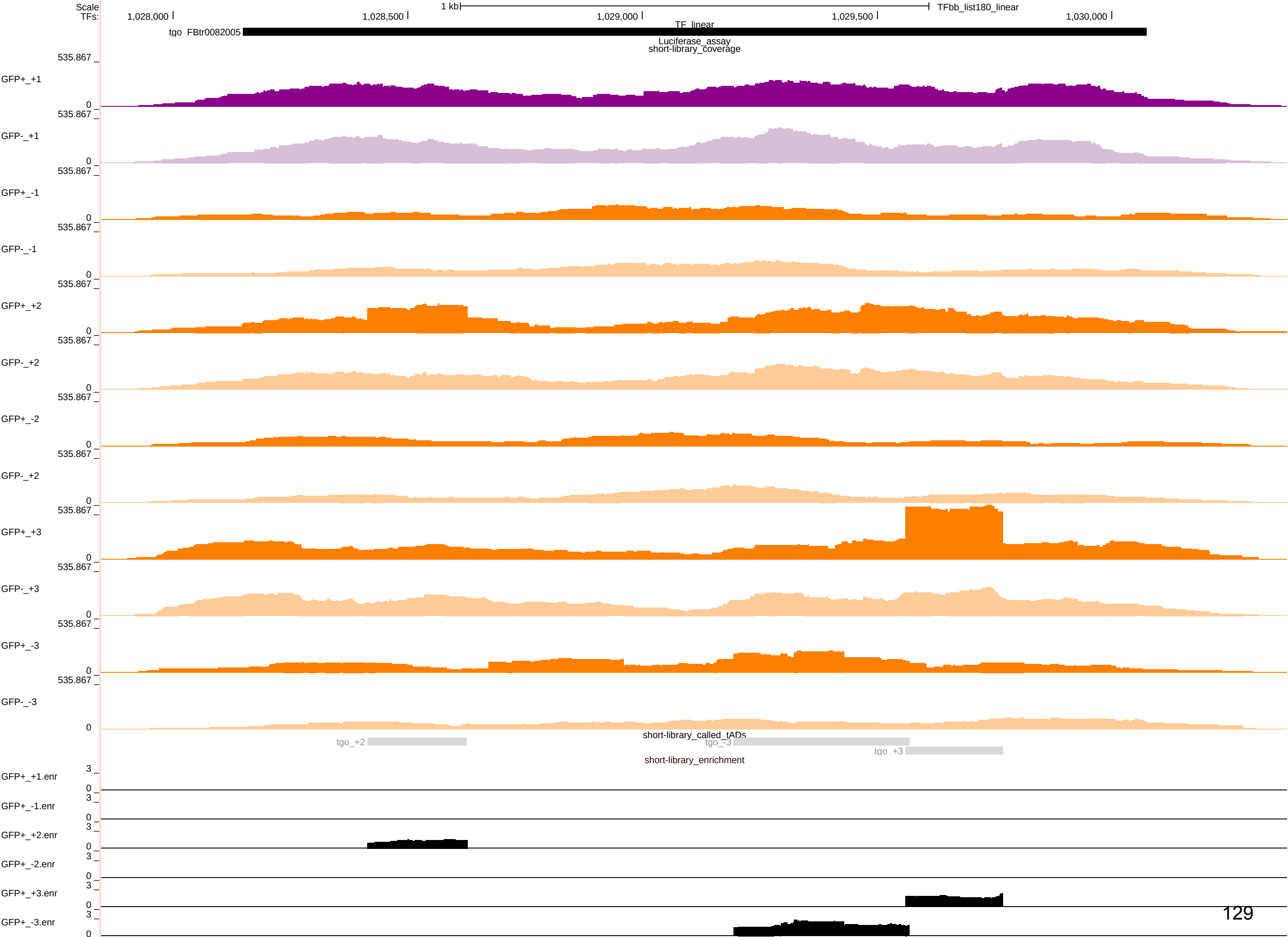
Scale
TFs:
1 kb
TFbb_list180_linear
1,028,000
1,028,500
1,029,000
1,029,500
1,030,000
TF_linear
tgo_FBtr0082005
Luciferase_assay
short-library_coverage
535.867
GFP+_+1
0
GFP-_+1
GFP+_-1
GFP-_-1
GFP+_+2
GFP-_+2
GFP+_-2
GFP-_+2
GFP+_+3
GFP-_+3
GFP+_-3
GFP-_-3
short-library_called_tADs
tgo_+2
tgo_-3
tgo_+3
short-library_enrichment
GFP+_+1.enr
3
GFP+_-1.enr
GFP+_+2.enr
GFP+_-2.enr
GFP+_+3.enr
GFP+_-3.enr

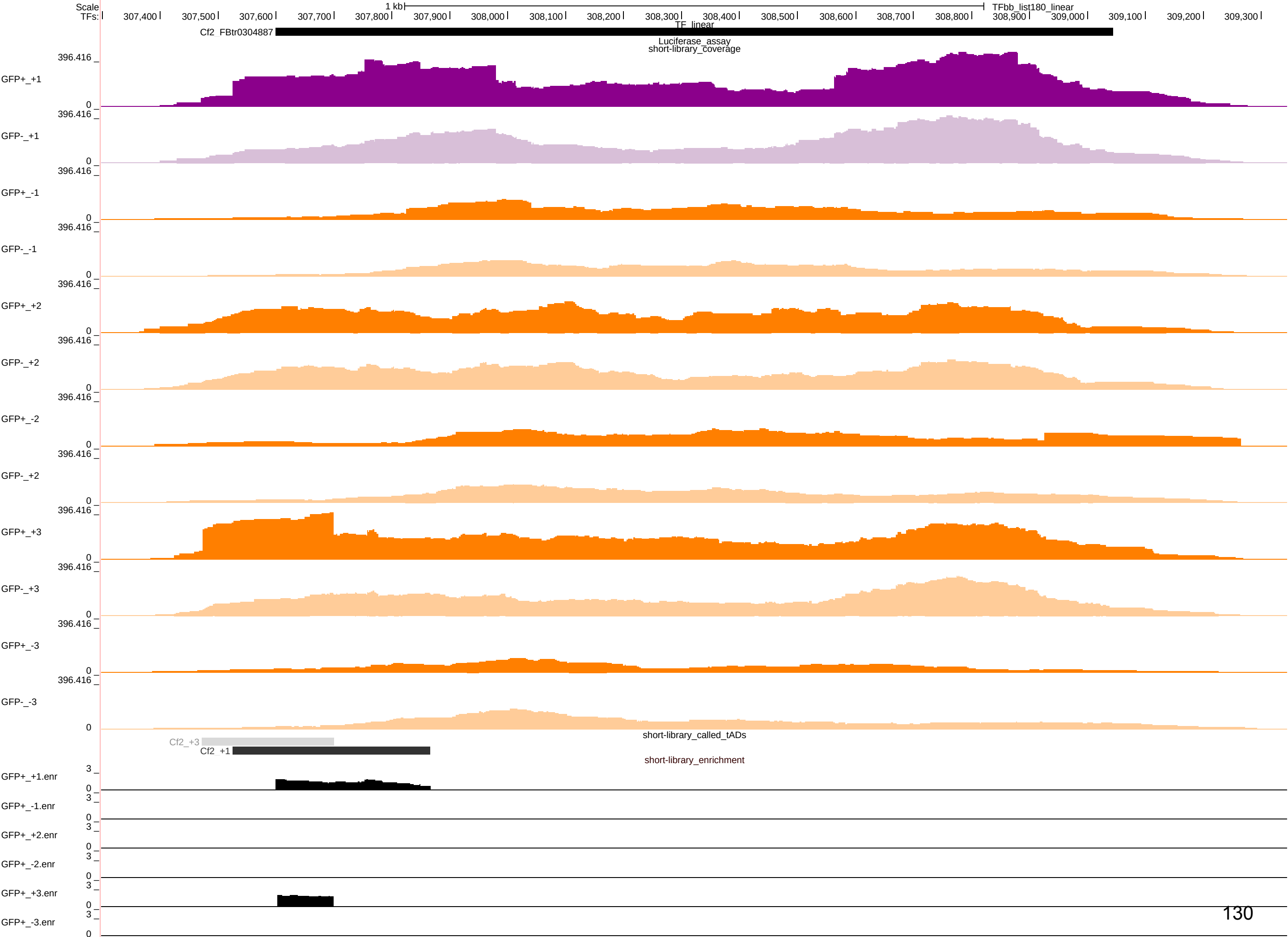
Scale
TFs:
1 kb
TFbb_list180_linear
307,400
307,500
307,600
307,700
307,800
307,900
308,000
308,100
308,200
308,300
308,400
308,500
308,600
308,700
308,800
308,900
309,000
309,100
309,200
309,300
TF_linear
Cf2_FBtr0304887
Luciferase_assay
short-library_coverage
GFP+_+1
GFP-_+1
GFP+_-1
GFP-_-1
GFP+_+2
GFP-_+2
GFP+_-2
GFP-_+2
GFP+_+3
GFP-_+3
GFP+_-3
GFP-_-3
396.416
0
short-library_called_tADs
Cf2_+3
Cf2_+1
short-library_enrichment
GFP+_+1.enr
GFP+_-1.enr
GFP+_+2.enr
GFP+_-2.enr
GFP+_+3.enr
GFP+_-3.enr
3
0

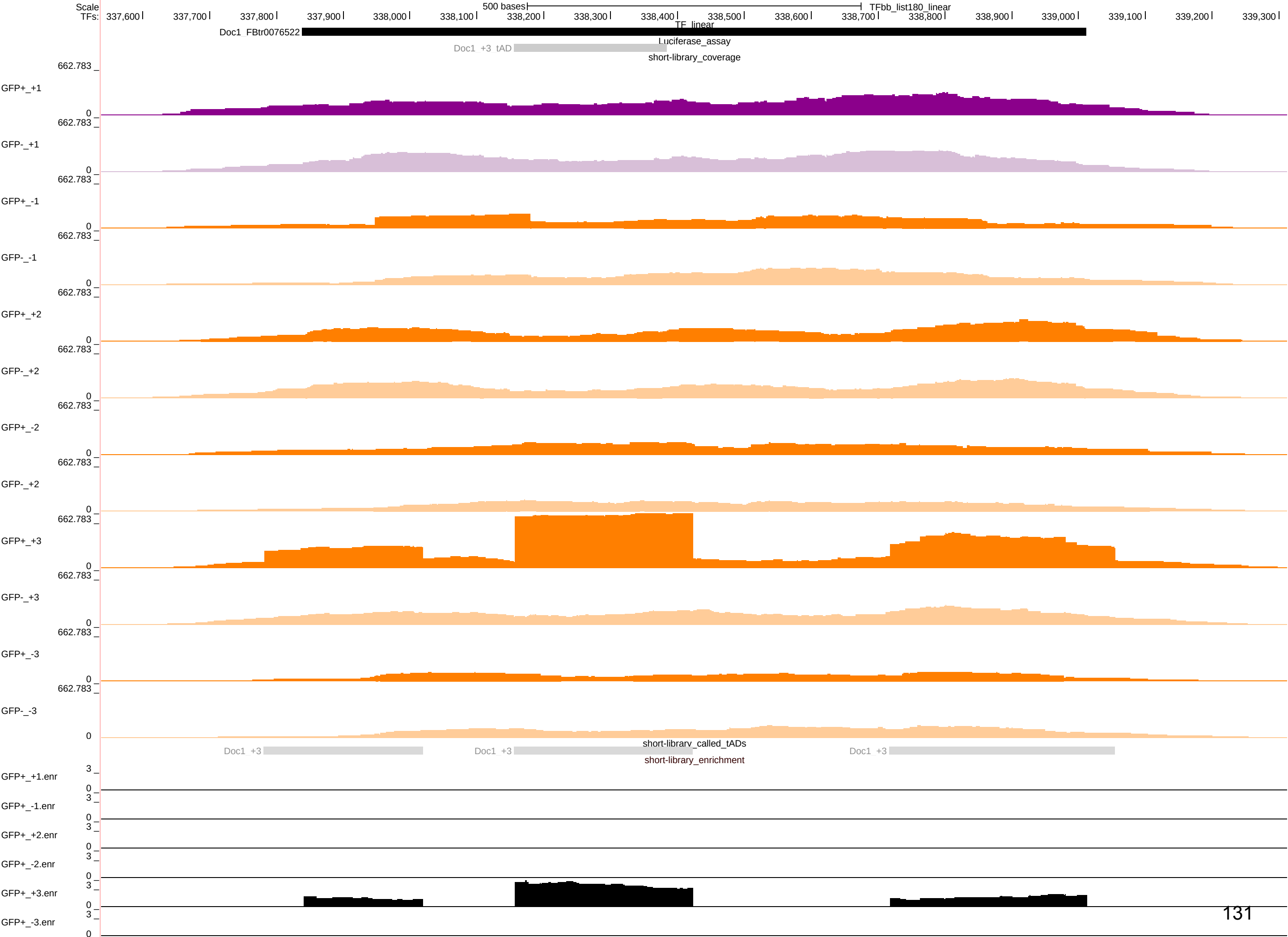

Scale
500 bases
TFs: 337,600 337,700 337,800 337,900 338,000 338,100 338,200 338,300 338,400 338,500 338,600 338,700 338,800 338,900 339,000 339,100 339,200 339,300
TFbb_list180_linear
TF_linear
Doc1_FBtr0076522
Luciferase_assay
Doc1_+3_tAD
short-library_coverage
662.783
0
GFP+_+1
GFP-_+1
GFP+_-1
GFP-_-1
GFP+_+2
GFP-_+2
GFP+_-2
GFP-_+2
GFP+_+3
GFP-_+3
GFP+_-3
GFP-_-3
short-library_called_tADs
Doc1_+3
Doc1_+3
Doc1_+3
short-library_enrichment
3
GFP+_+1.enr
GFP+_-1.enr
GFP+_+2.enr
GFP+_-2.enr
GFP+_+3.enr
GFP+_-3.enr

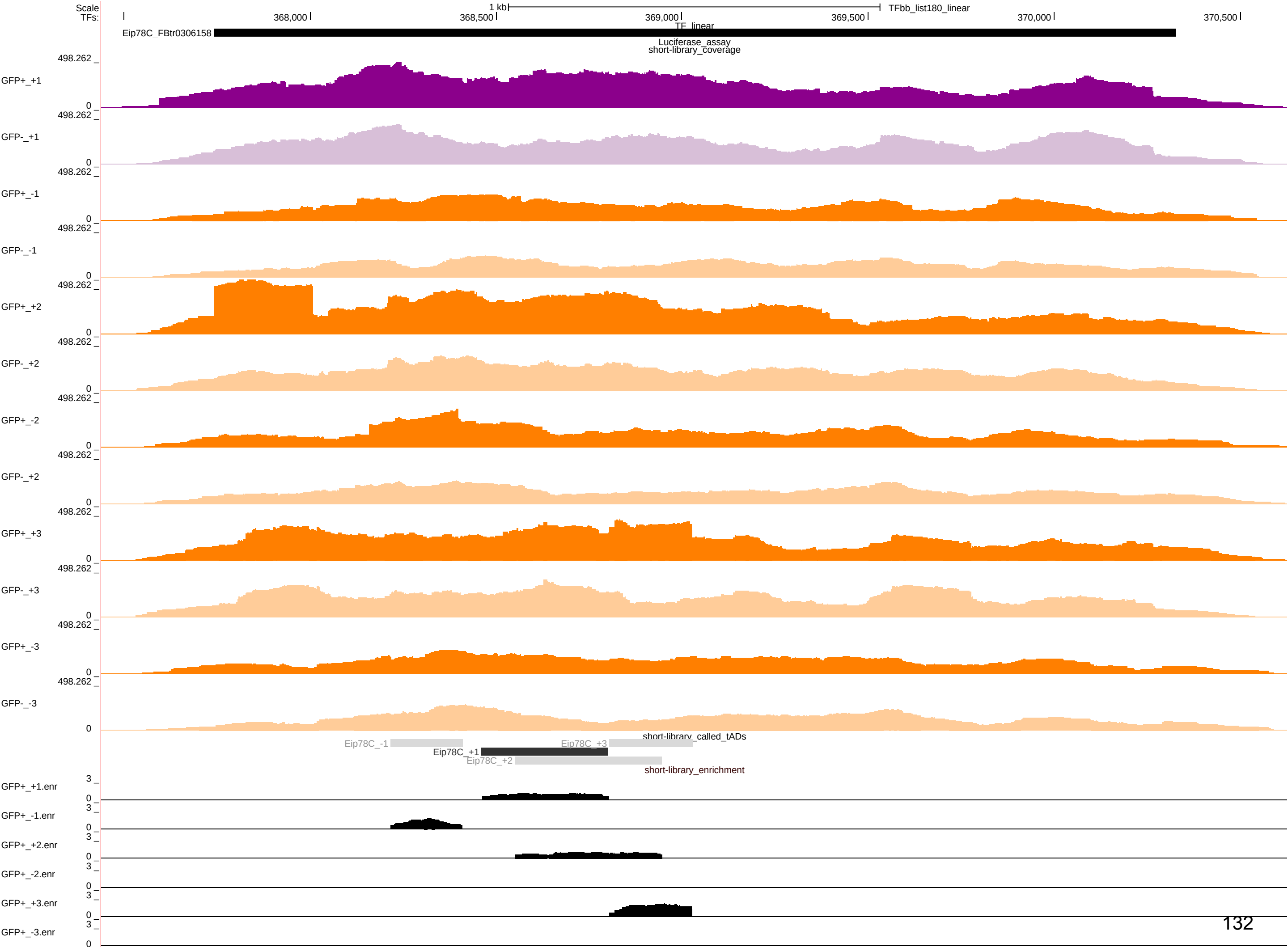
Scale
TFs:
1 kb
TFbb_list180_linear
368,000
368,500
369,000
369,500
370,000
370,500
TF_linear
Eip78C FBtr0306158
Luciferase_assay
short-library_coverage
498.262
0
GFP+_+1
GFP-_+1
GFP+_-1
GFP-_-1
GFP+_+2
GFP-_+2
GFP+_-2
GFP-_+2
GFP+_+3
GFP-_+3
GFP+_-3
GFP-_-3
short-library_called_tADs
Eip78C_-1
Eip78C_+3
Eip78C_+1
Eip78C_+2
short-library_enrichment
3
GFP+_+1.enr
GFP+_-1.enr
GFP+_+2.enr
GFP+_-2.enr
GFP+_+3.enr
GFP+_-3.enr

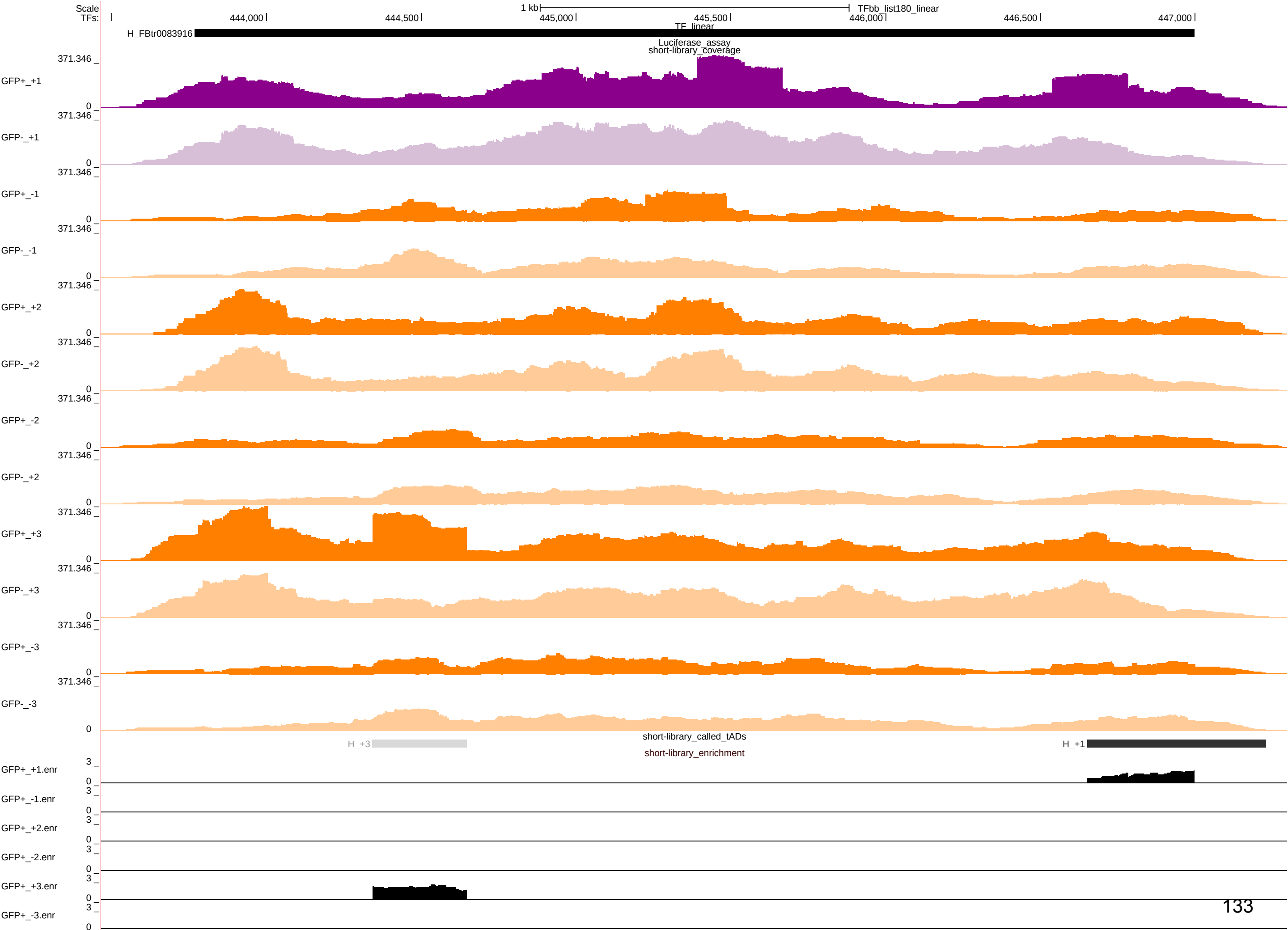

Scale
TFs:
1 kb
TFbb_list180_linear
444,000
444,500
445,000
445,500
446,000
446,500
447,000
H_FBtr0083916
TF_linear
Luciferase_assay
short-library_coverage
GFP+_+1
GFP-_+1
GFP+_-1
GFP-_-1
GFP+_+2
GFP-_+2
GFP+_-2
GFP-_+2
GFP+_+3
GFP-_+3
GFP+_-3
GFP-_-3
371.346
0
short-library_called_tADs
H_+3
H_+1
short-library_enrichment
GFP+_+1.enr
GFP+_-1.enr
GFP+_+2.enr
GFP+_-2.enr
GFP+_+3.enr
GFP+_-3.enr
3
0

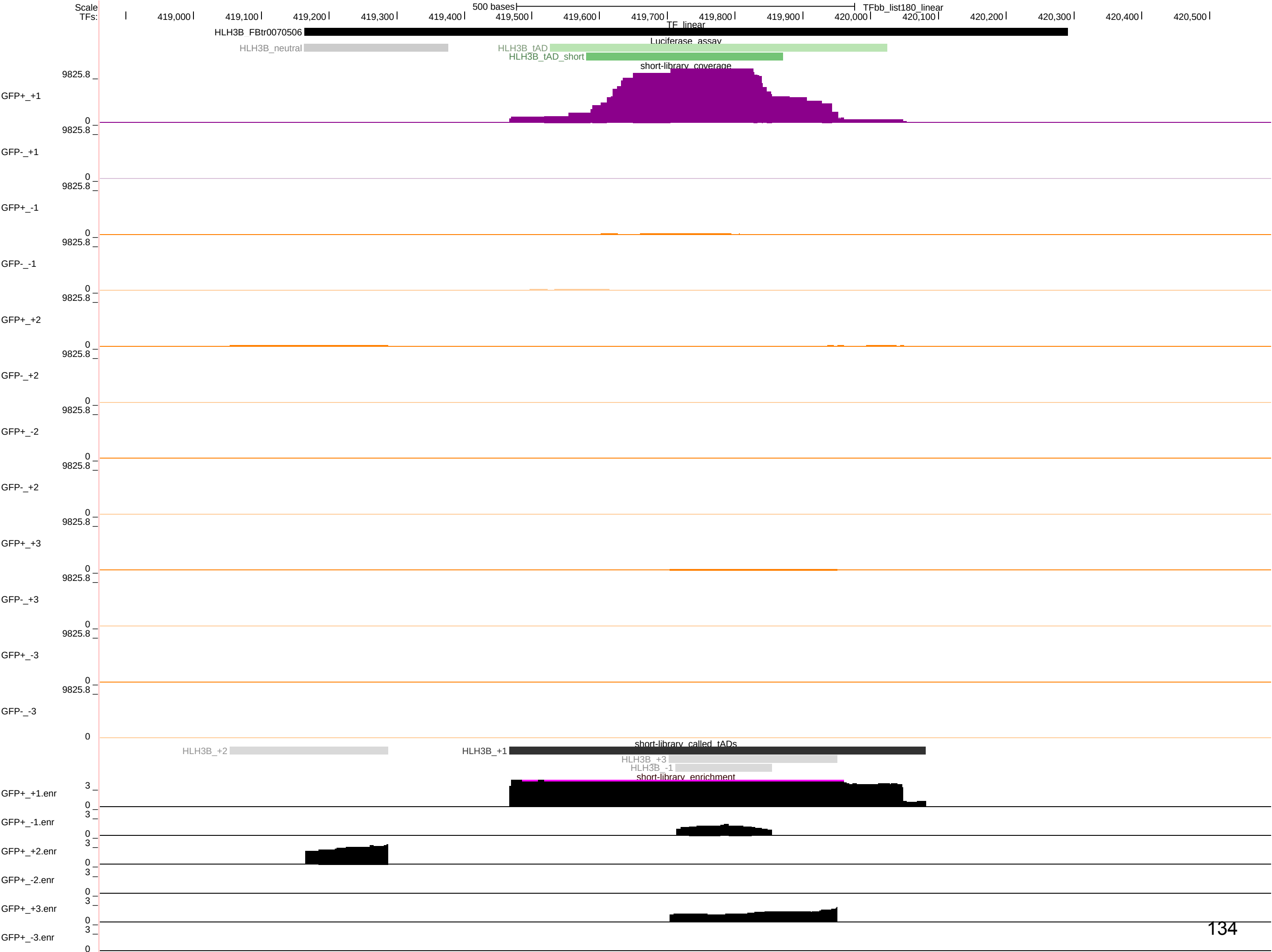

Scale
TFs:
500 bases
TFbb_list180_linear
419,000
419,100
419,200
419,300
419,400
419,500
419,600
419,700
419,800
419,900
420,000
420,100
420,200
420,300
420,400
420,500
TF_linear
HLH3B_FBtr0070506
Luciferase_assay
HLH3B_neutral
HLH3B_tAD
HLH3B_tAD_short
short-library_coverage
9825.8 _
GFP+_+1
0 _
GFP-_+1
GFP+_-1
GFP-_-1
GFP+_+2
GFP-_+2
GFP+_-2
GFP-_+2
GFP+_+3
GFP-_+3
GFP+_-3
GFP-_-3
short-library_called_tADs
HLH3B_+2
HLH3B_+1
HLH3B_+3
HLH3B_-1
short-library_enrichment
GFP+_+1.enr
GFP+_-1.enr
GFP+_+2.enr
GFP+_-2.enr
GFP+_+3.enr
GFP+_-3.enr
3 _
0 _

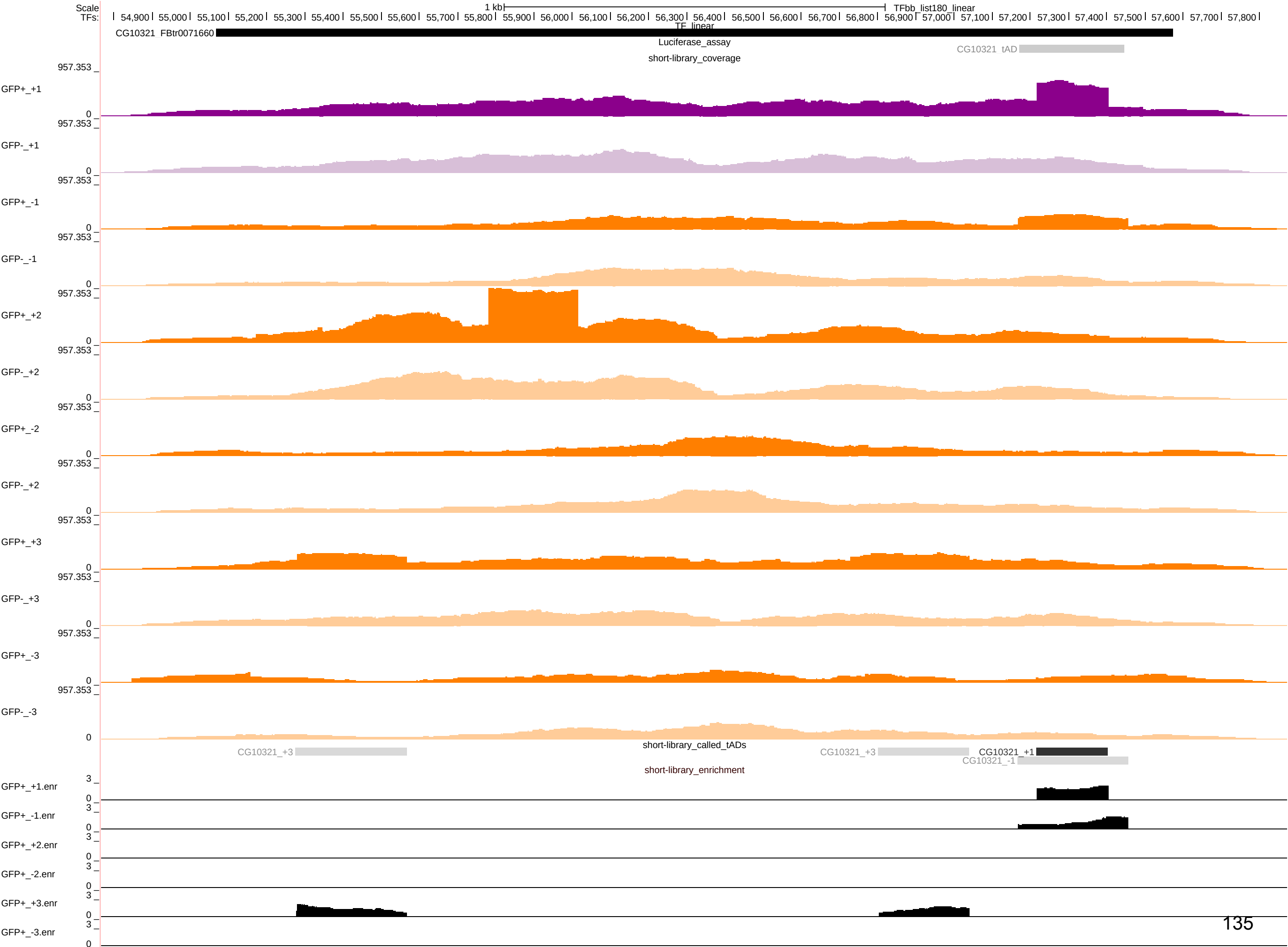

Scale
1 kb
TFs:
54,900
55,000
55,100
55,200
55,300
55,400
55,500
55,600
55,700
55,800
55,900
56,000
56,100
56,200
56,300
56,400
56,500
56,600
56,700
56,800
56,900
57,000
57,100
57,200
57,300
57,400
57,500
57,600
57,700
57,800
TFbb_list180_linear
TF_linear
CG10321 FBtr0071660
Luciferase_assay
CG10321 tAD
short-library_coverage
GFP+_+1
GFP-_+1
GFP+_-1
GFP-_-1
GFP+_+2
GFP-_+2
GFP+_-2
GFP-_+2
GFP+_+3
GFP-_+3
GFP+_-3
GFP-_-3
957.353
0
short-library_called_tADs
CG10321_+3
CG10321_+3
CG10321_+1
CG10321_-1
short-library_enrichment
GFP+_+1.enr
GFP+_-1.enr
GFP+_+2.enr
GFP+_-2.enr
GFP+_+3.enr
GFP+_-3.enr
3
0

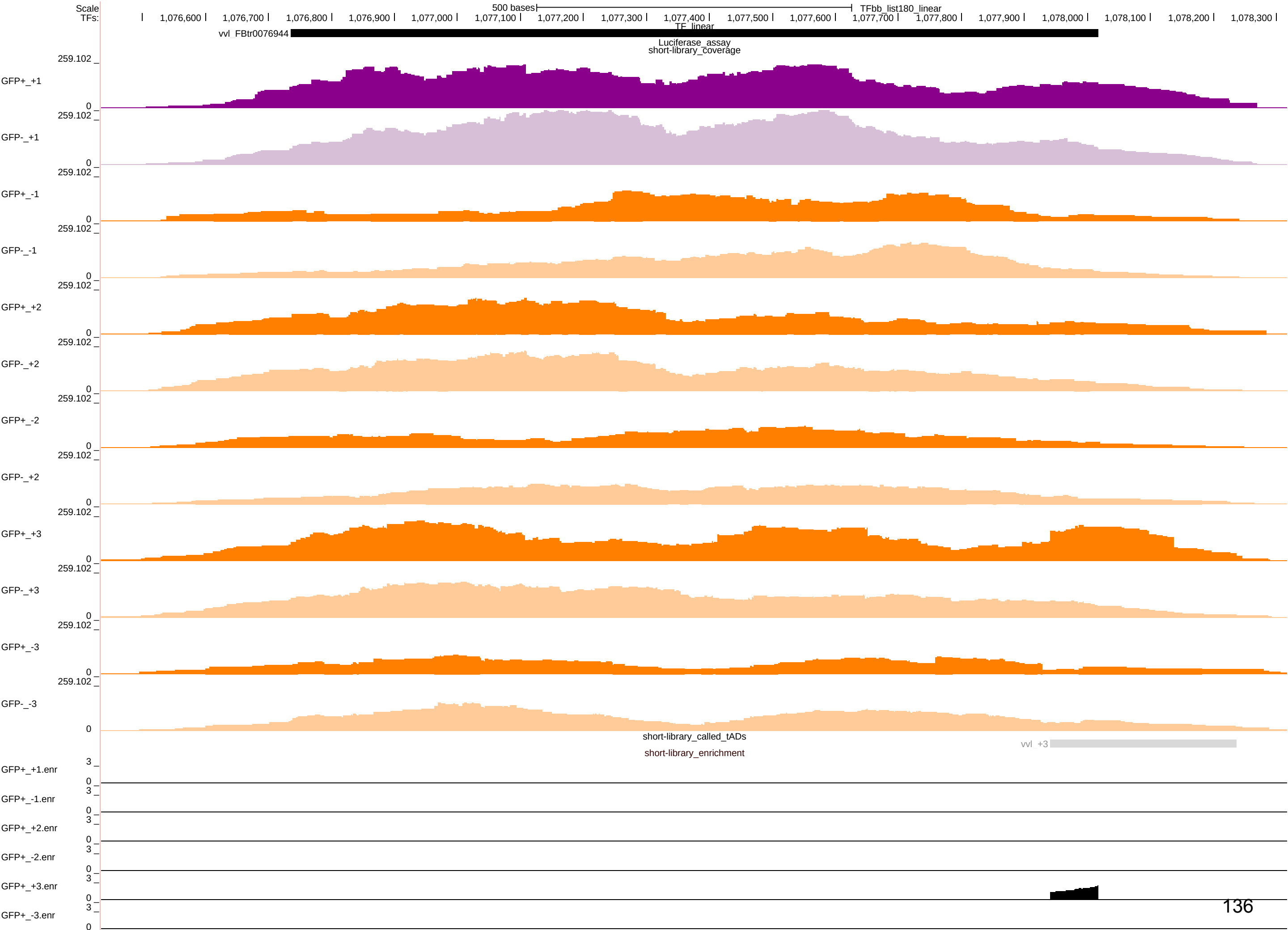

Scale
TFs:
500 bases
TFbb_list180_linear
1,076,600
1,076,700
1,076,800
1,076,900
1,077,000
1,077,100
1,077,200
1,077,300
1,077,400
1,077,500
1,077,600
1,077,700
1,077,800
1,077,900
1,078,000
1,078,100
1,078,200
1,078,300
TF_linear
vvl FBtr0076944
Luciferase_assay
short-library_coverage
259.102
0
GFP+_+1
GFP-_+1
GFP+_-1
GFP-_-1
GFP+_+2
GFP-_+2
GFP+_-2
GFP-_+2
GFP+_+3
GFP-_+3
GFP+_-3
GFP-_-3
short-library_called_tADs
vvl +3
short-library_enrichment
3
GFP+_+1.enr
GFP+_-1.enr
GFP+_+2.enr
GFP+_-2.enr
GFP+_+3.enr
GFP+_-3.enr

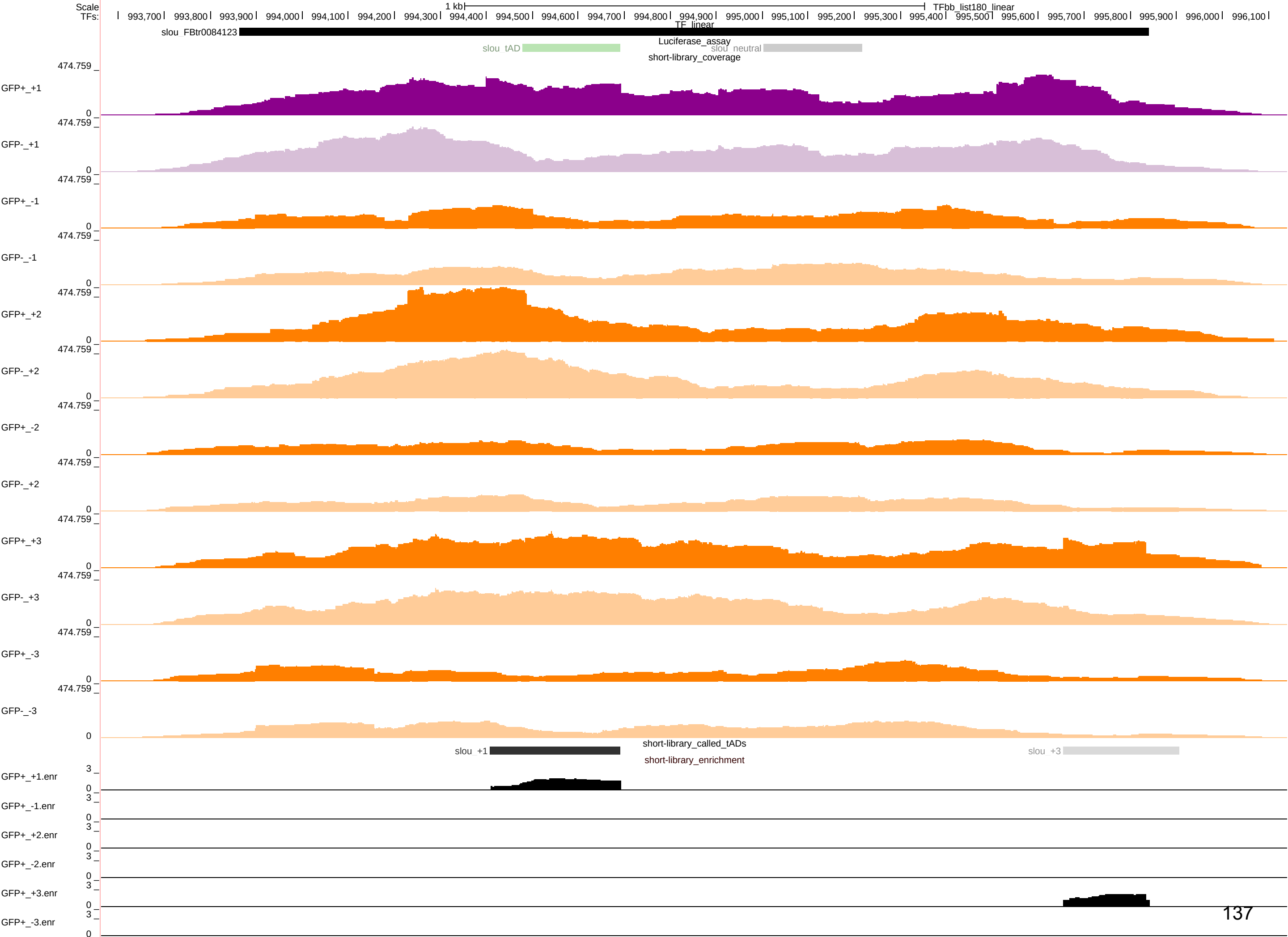

Scale
TFs:
1 kb
993,700
993,800
993,900
994,000
994,100
994,200
994,300
994,400
994,500
994,600
994,700
994,800
994,900
995,000
995,100
995,200
995,300
995,400
995,500
995,600
995,700
995,800
995,900
996,000
996,100
TFbb_list180_linear
TF_linear
slou_FBtr0084123
Luciferase_assay
slou_tAD
slou_neutral
short-library_coverage
GFP+_+1
GFP-_+1
GFP+_-1
GFP-_-1
GFP+_+2
GFP-_+2
GFP+_-2
GFP-_+2
GFP+_+3
GFP-_+3
GFP+_-3
GFP-_-3
474.759
0
short-library_called_tADs
slou_+1
slou_+3
short-library_enrichment
GFP+_+1.enr
GFP+_-1.enr
GFP+_+2.enr
GFP+_-2.enr
GFP+_+3.enr
GFP+_-3.enr
3
0

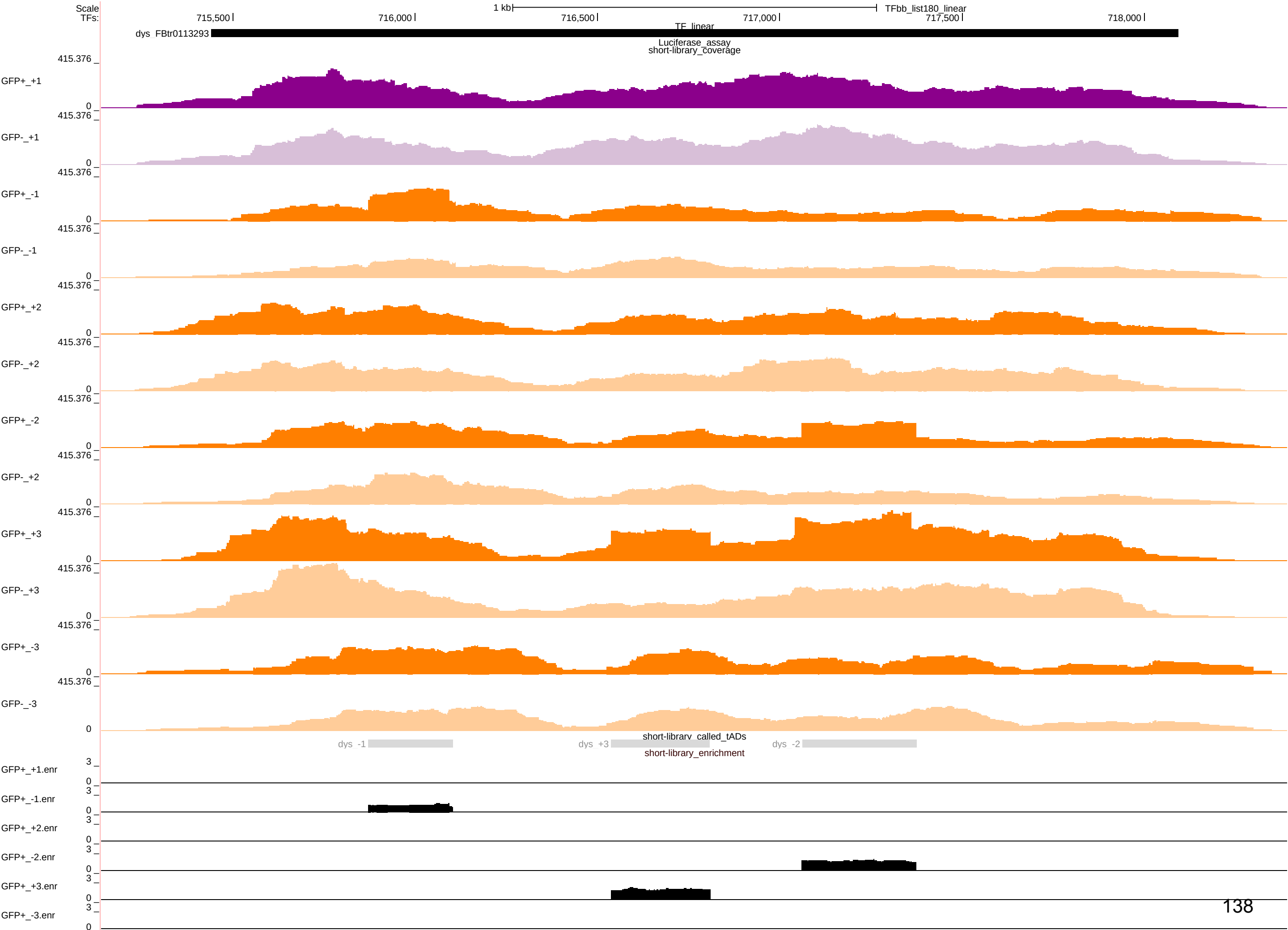

Scale
TFs:
1 kb
TFbb_list180_linear
715,500
716,000
716,500
717,000
717,500
718,000
TF_linear
dys_FBtr0113293
Luciferase_assay
short-library_coverage
415.376 _
GFP+_+1
0 _
GFP-_+1
GFP+_-1
GFP-_-1
GFP+_+2
GFP-_+2
GFP+_-2
GFP-_+2
GFP+_+3
GFP-_+3
GFP+_-3
GFP-_-3
short-library_called_tADs
dys_-1
dys_+3
dys_-2
short-library_enrichment
3 _
GFP+_+1.enr
GFP+_-1.enr
GFP+_+2.enr
GFP+_-2.enr
GFP+_+3.enr
GFP+_-3.enr

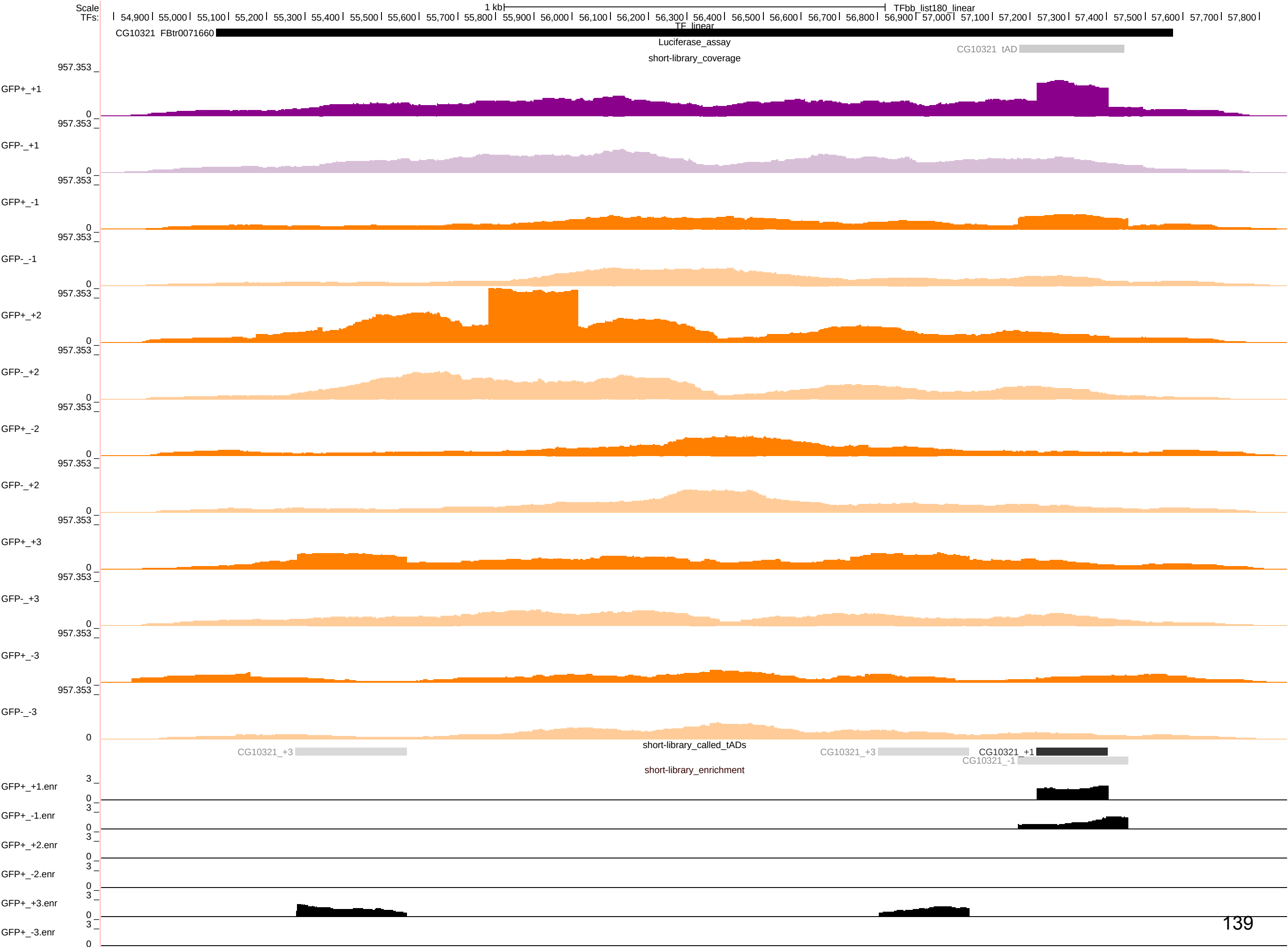

Scale
1 kb
TFs:
54,900
55,000
55,100
55,200
55,300
55,400
55,500
55,600
55,700
55,800
55,900
56,000
56,100
56,200
56,300
56,400
56,500
56,600
56,700
56,800
56,900
57,000
57,100
57,200
57,300
57,400
57,500
57,600
57,700
57,800
TFbb_list180_linear
TF_linear
CG10321 FBtr0071660
Luciferase_assay
CG10321 tAD
short-library_coverage
GFP+_+1
GFP-_+1
GFP+_-1
GFP-_-1
GFP+_+2
GFP-_+2
GFP+_-2
GFP-_+2
GFP+_+3
GFP-_+3
GFP+_-3
GFP-_-3
957.353
0
short-library_called_tADs
CG10321_+3
CG10321_+3
CG10321_+1
CG10321_-1
short-library_enrichment
GFP+_+1.enr
GFP+_-1.enr
GFP+_+2.enr
GFP+_-2.enr
GFP+_+3.enr
GFP+_-3.enr
3
0

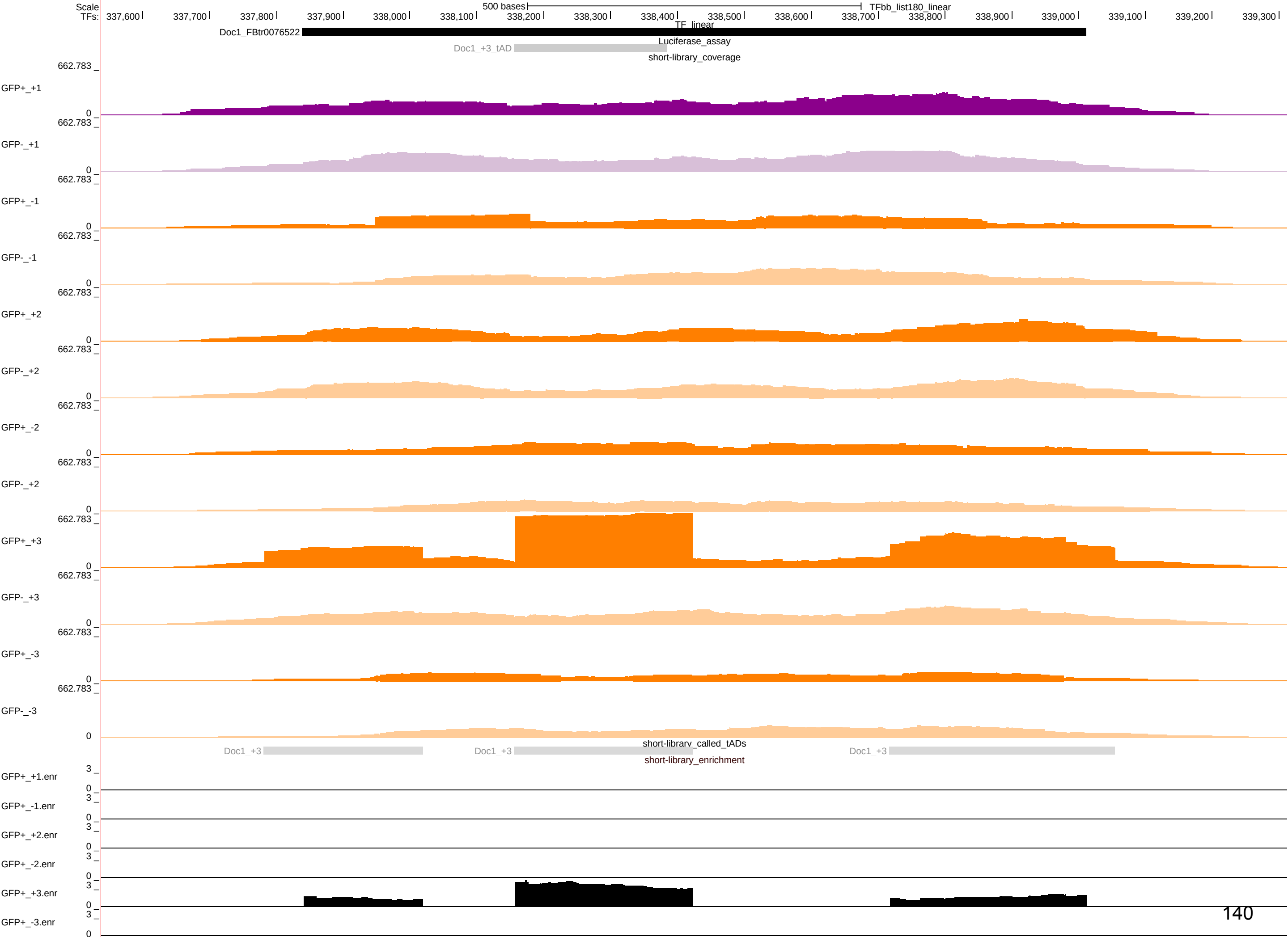

Scale
500 bases
TFbb_list180_linear
TFs:
337,600
337,700
337,800
337,900
338,000
338,100
338,200
338,300
338,400
338,500
338,600
338,700
338,800
338,900
339,000
339,100
339,200
339,300
TF_linear
Doc1_FBtr0076522
Luciferase_assay
Doc1_+3_tAD
short-library_coverage
GFP+_+1
GFP-_+1
GFP+_-1
GFP-_-1
GFP+_+2
GFP-_+2
GFP+_-2
GFP-_+2
GFP+_+3
GFP-_+3
GFP+_-3
GFP-_-3
662.783
0
short-library_called_tADs
Doc1_+3
short-library_enrichment
GFP+_+1.enr
GFP+_-1.enr
GFP+_+2.enr
GFP+_-2.enr
GFP+_+3.enr
GFP+_-3.enr
3
0

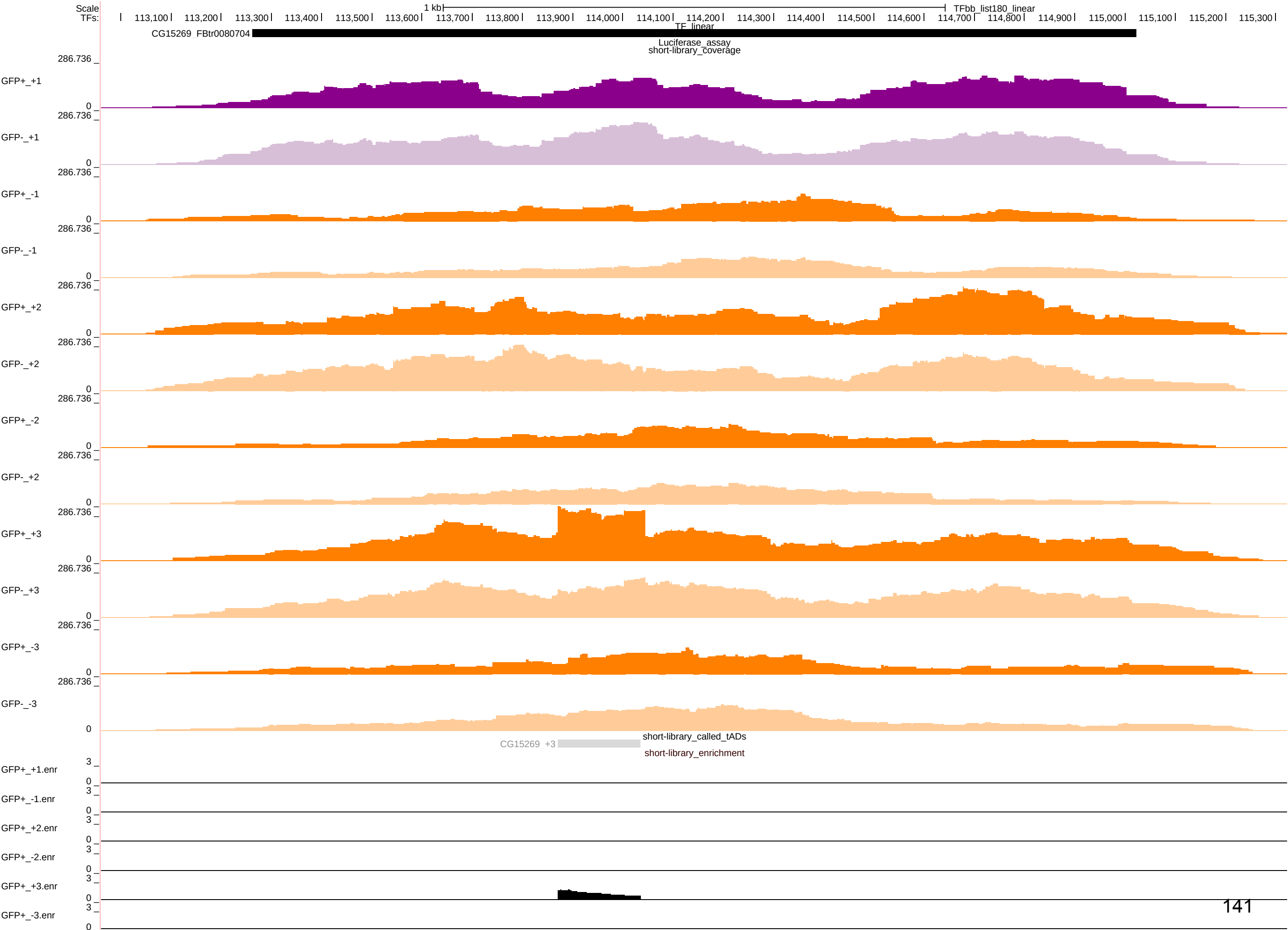
Scale
TFs:
1 kb
TFbb_list180_linear
113,100 113,200 113,300 113,400 113,500 113,600 113,700 113,800 113,900 114,000 114,100 114,200 114,300 114,400 114,500 114,600 114,700 114,800 114,900 115,000 115,100 115,200 115,300
TF_linear
CG15269 FBtr0080704
Luciferase_assay
short-library_coverage
GFP+_+1
GFP-_+1
GFP+_-1
GFP-_-1
GFP+_+2
GFP-_+2
GFP+_-2
GFP-_+2
GFP+_+3
GFP-_+3
GFP+_-3
GFP-_-3
286.736
0
short-library_called_tADs
CG15269_+3
short-library_enrichment
GFP+_+1.enr
GFP+_-1.enr
GFP+_+2.enr
GFP+_-2.enr
GFP+_+3.enr
GFP+_-3.enr
3
0

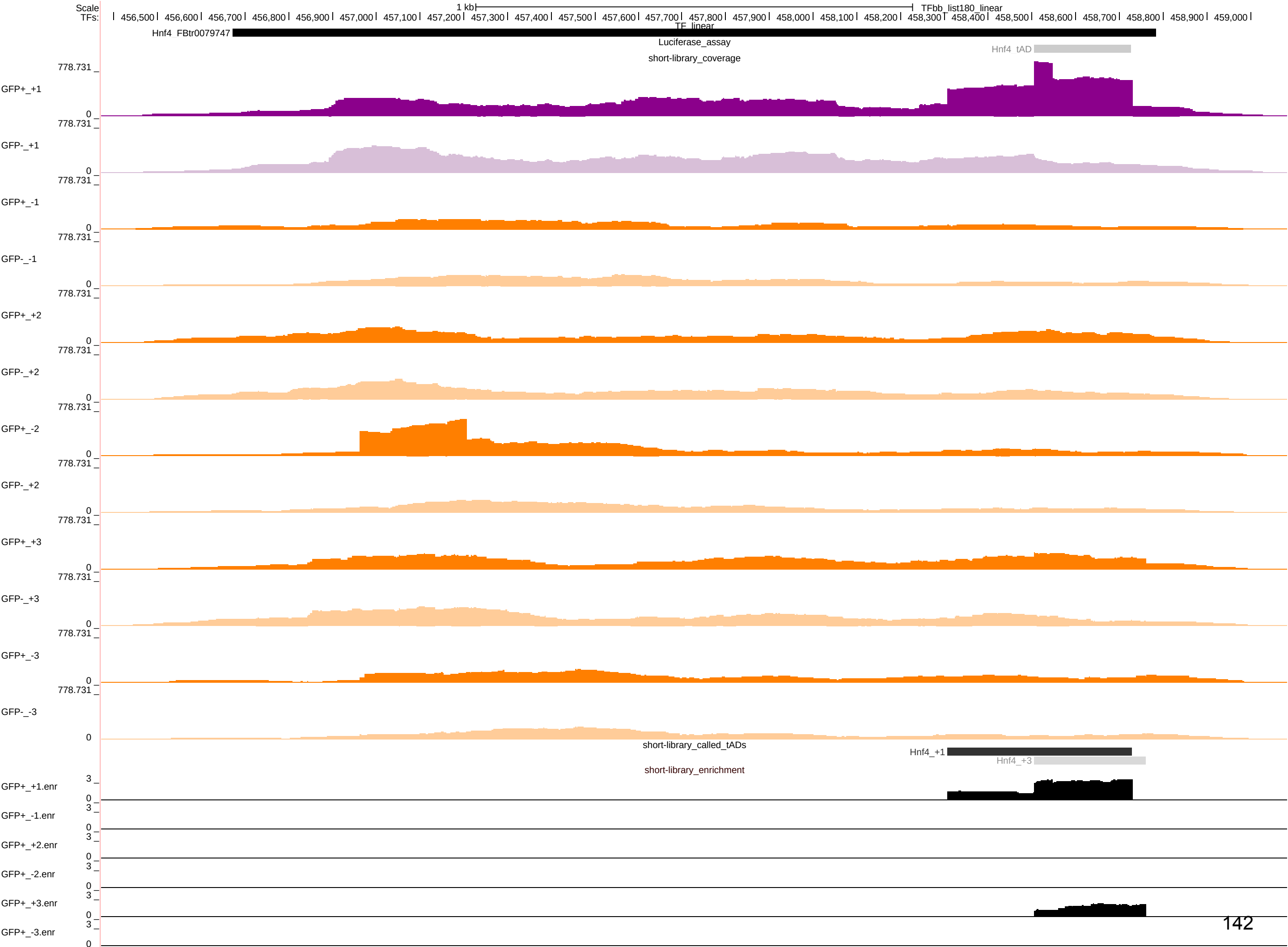

Scale
TFs:
1 kb
TFbb_list180_linear
456,500 456,600 456,700 456,800 456,900 457,000 457,100 457,200 457,300 457,400 457,500 457,600 457,700 457,800 457,900 458,000 458,100 458,200 458,300 458,400 458,500 458,600 458,700 458,800 458,900 459,000
TF_linear
Hnf4 FBtr0079747
Luciferase_assay
Hnf4 tAD
short-library_coverage
778.731
0
GFP+_+1
GFP-_+1
GFP+_-1
GFP-_-1
GFP+_+2
GFP-_+2
GFP+_-2
GFP-_+2
GFP+_+3
GFP-_+3
GFP+_-3
GFP-_-3
short-library_called_tADs
Hnf4_+1
Hnf4_+3
short-library_enrichment
3
GFP+_+1.enr
GFP+_-1.enr
GFP+_+2.enr
GFP+_-2.enr
GFP+_+3.enr
GFP+_-3.enr

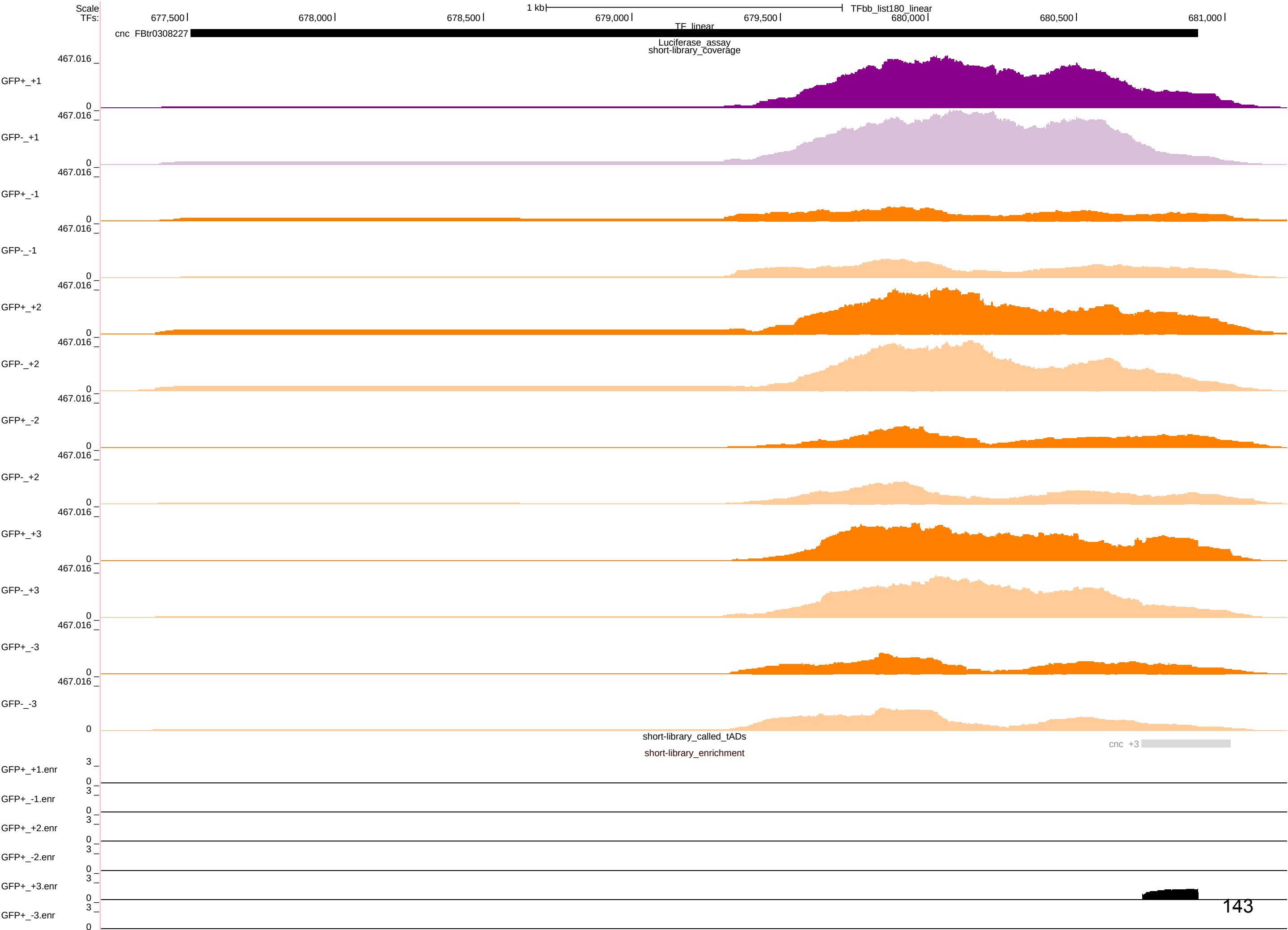
Scale
TFs:
1 kb
TFbb_list180_linear
677,500
678,000
678,500
679,000
679,500
680,000
680,500
681,000
TF_linear
cnc FBtr0308227
Luciferase_assay
short-library_coverage
GFP+_+1
GFP-_+1
GFP+_-1
GFP-_-1
GFP+_+2
GFP-_+2
GFP+_-2
GFP-_+2
GFP+_+3
GFP-_+3
GFP+_-3
GFP-_-3
467.016
0
short-library_called_tADs
cnc_+3
short-library_enrichment
GFP+_+1.enr
GFP+_-1.enr
GFP+_+2.enr
GFP+_-2.enr
GFP+_+3.enr
GFP+_-3.enr
3
0

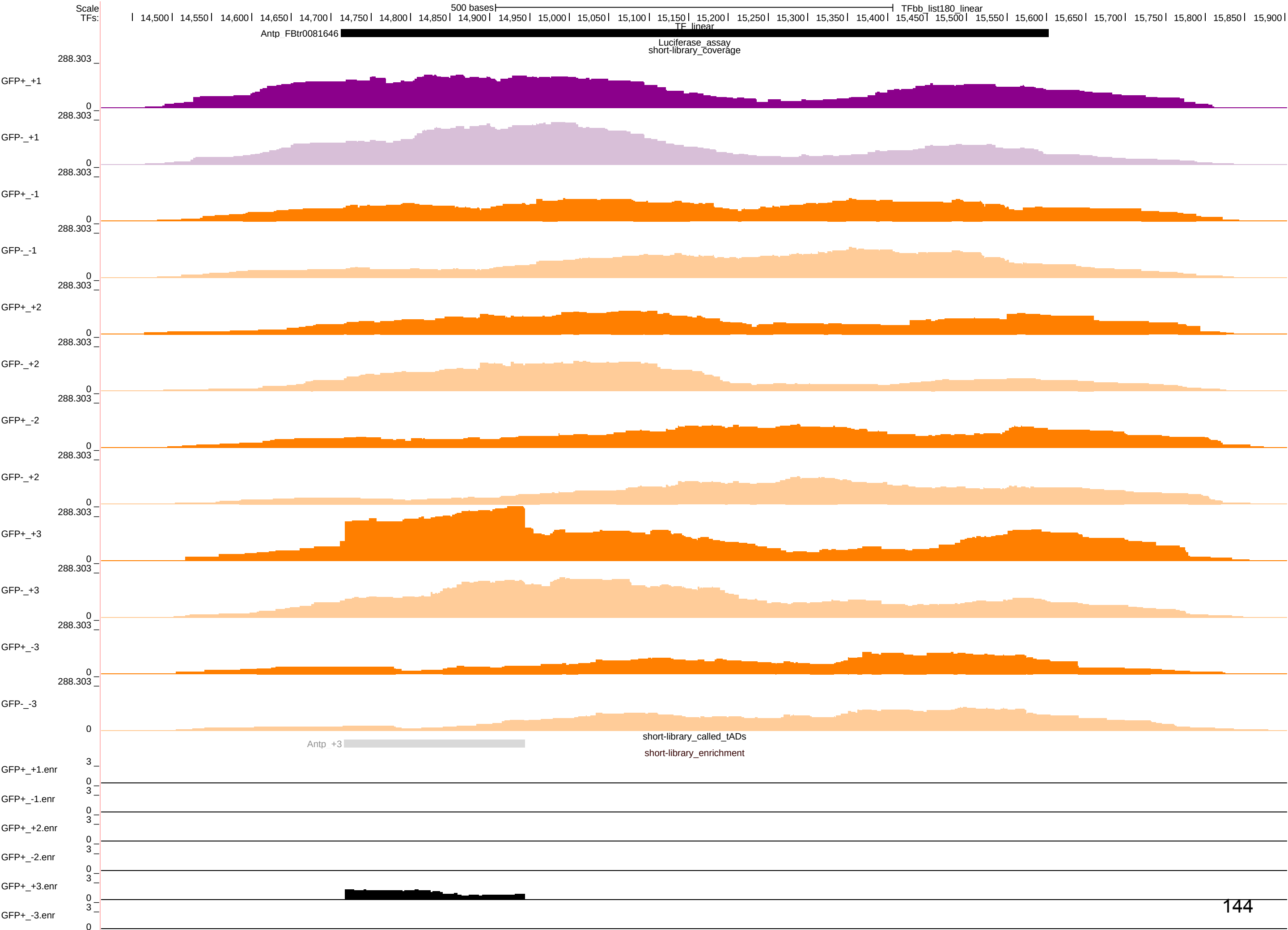

Scale
TFs:
500 bases
TFbb_list180_linear
TF_linear
Antp_FBtr0081646
Luciferase_assay
short-library_coverage
GFP+_+1
GFP-_+1
GFP+_-1
GFP-_-1
GFP+_+2
GFP-_+2
GFP+_-2
GFP-_+2
GFP+_+3
GFP-_+3
GFP+_-3
GFP-_-3
short-library_called_tADs
Antp_+3
short-library_enrichment
GFP+_+1.enr
GFP+_-1.enr
GFP+_+2.enr
GFP+_-2.enr
GFP+_+3.enr
GFP+_-3.enr

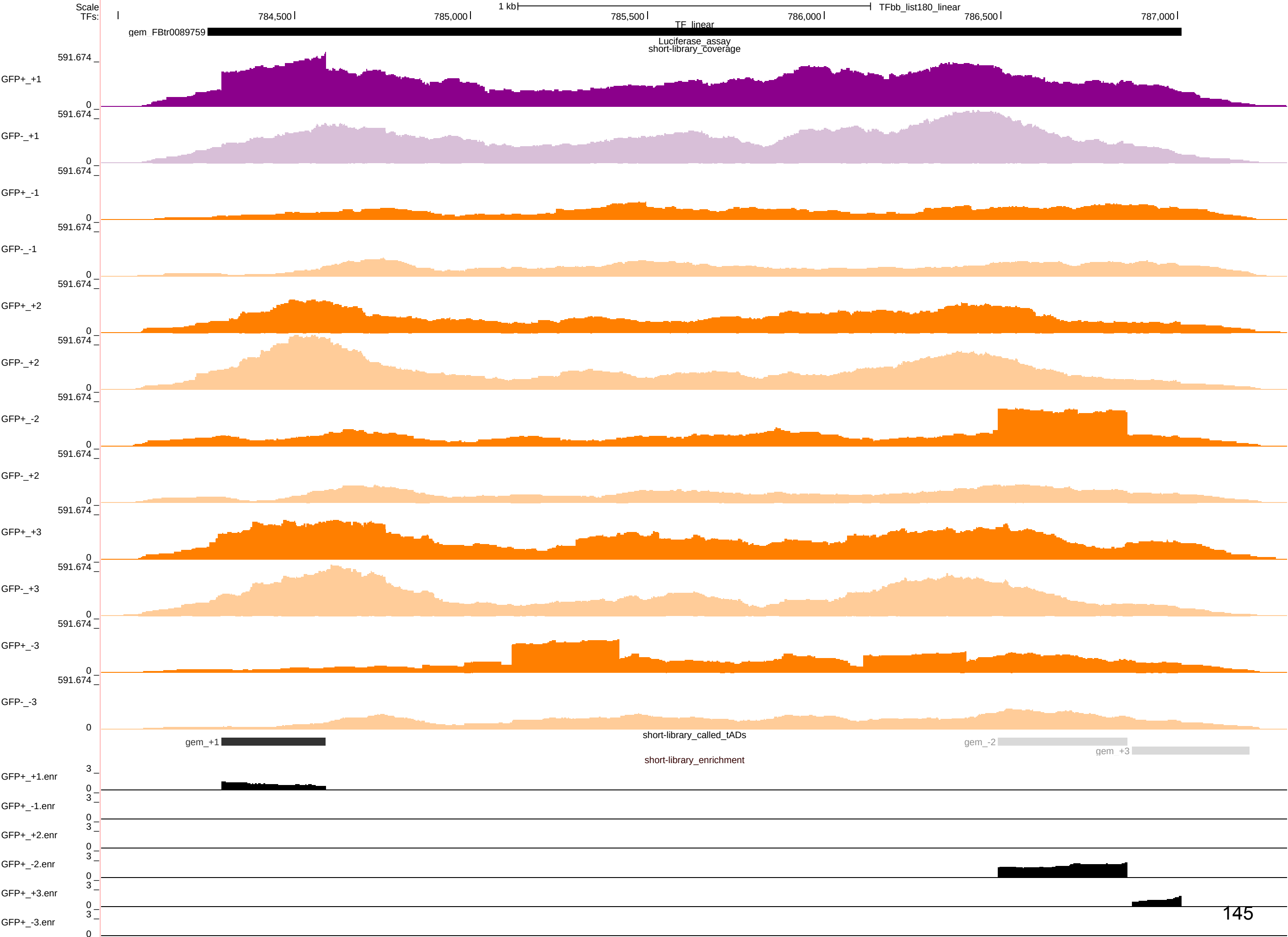
Scale
TFs:
1 kb
TFbb_list180_linear
784,500
785,000
785,500
786,000
786,500
787,000
TF_linear
gem_FBtr0089759
Luciferase_assay
short-library_coverage
591.674
0
GFP+_+1
GFP-_+1
GFP+_-1
GFP-_-1
GFP+_+2
GFP-_+2
GFP+_-2
GFP-_+2
GFP+_+3
GFP-_+3
GFP+_-3
GFP-_-3
short-library_called_tADs
gem_+1
gem_-2
gem_+3
short-library_enrichment
3
GFP+_+1.enr
GFP+_-1.enr
GFP+_+2.enr
GFP+_-2.enr
GFP+_+3.enr
GFP+_-3.enr

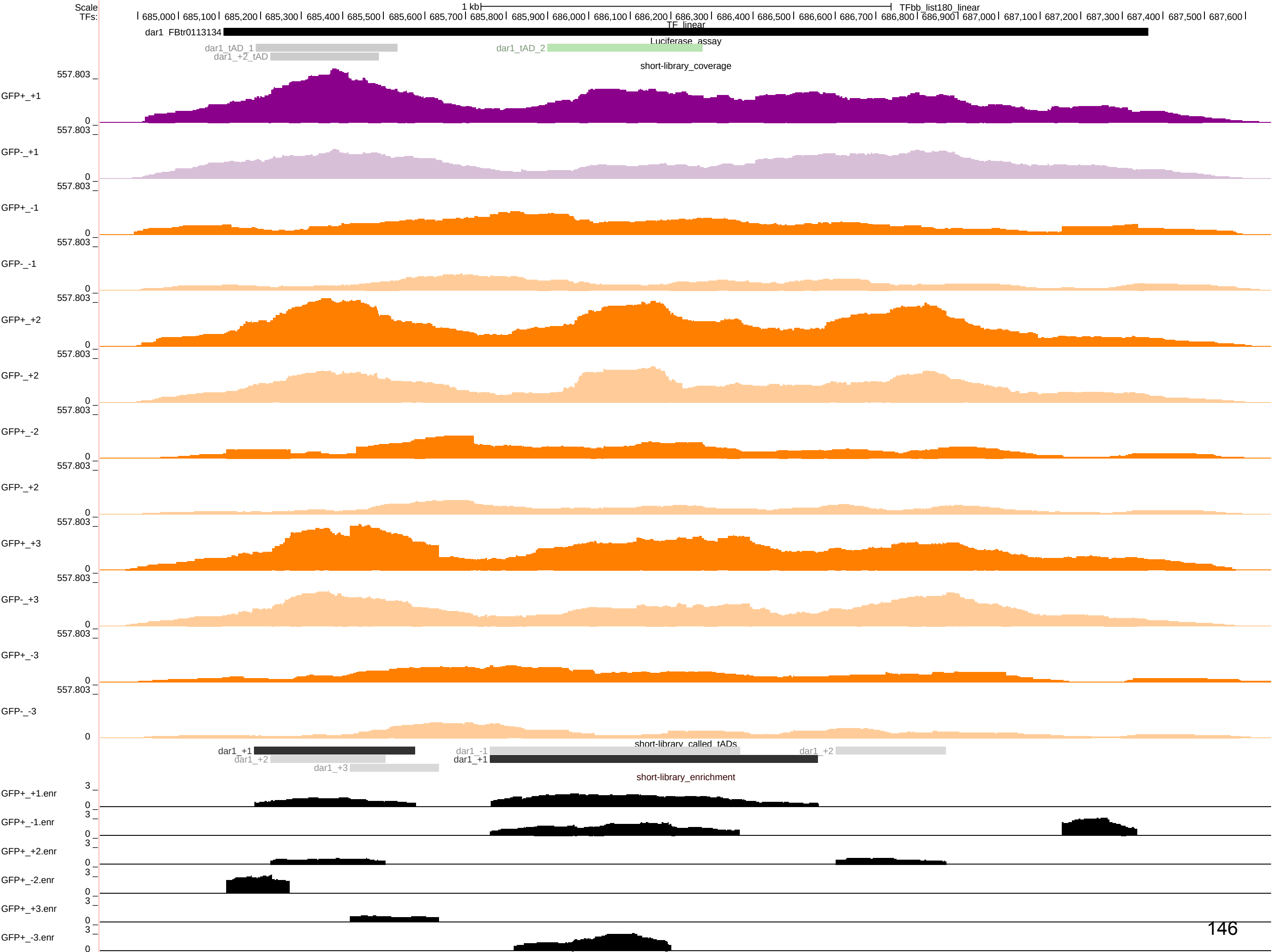
Scale
TFs:
1 kb
TFbb_list180_linear
685,000 685,100 685,200 685,300 685,400 685,500 685,600 685,700 685,800 685,900 686,000 686,100 686,200 686,300 686,400 686,500 686,600 686,700 686,800 686,900 687,000 687,100 687,200 687,300 687,400 687,500 687,600
TF_linear
dar1 FBtr0113134
Luciferase_assay
dar1_tAD_1
dar1_+2_tAD
dar1_tAD_2
short-library_coverage
GFP+_+1
GFP-_+1
GFP+_-1
GFP-_-1
GFP+_+2
GFP-_+2
GFP+_-2
GFP-_+2
GFP+_+3
GFP-_+3
GFP+_-3
GFP-_-3
557.803
0
short-library_called_tADs
dar1_+1
dar1_+2
dar1_+3
dar1_-1
dar1_+1
dar1_+2
short-library_enrichment
GFP+_+1.enr
GFP+_-1.enr
GFP+_+2.enr
GFP+_-2.enr
GFP+_+3.enr
GFP+_-3.enr
3
0

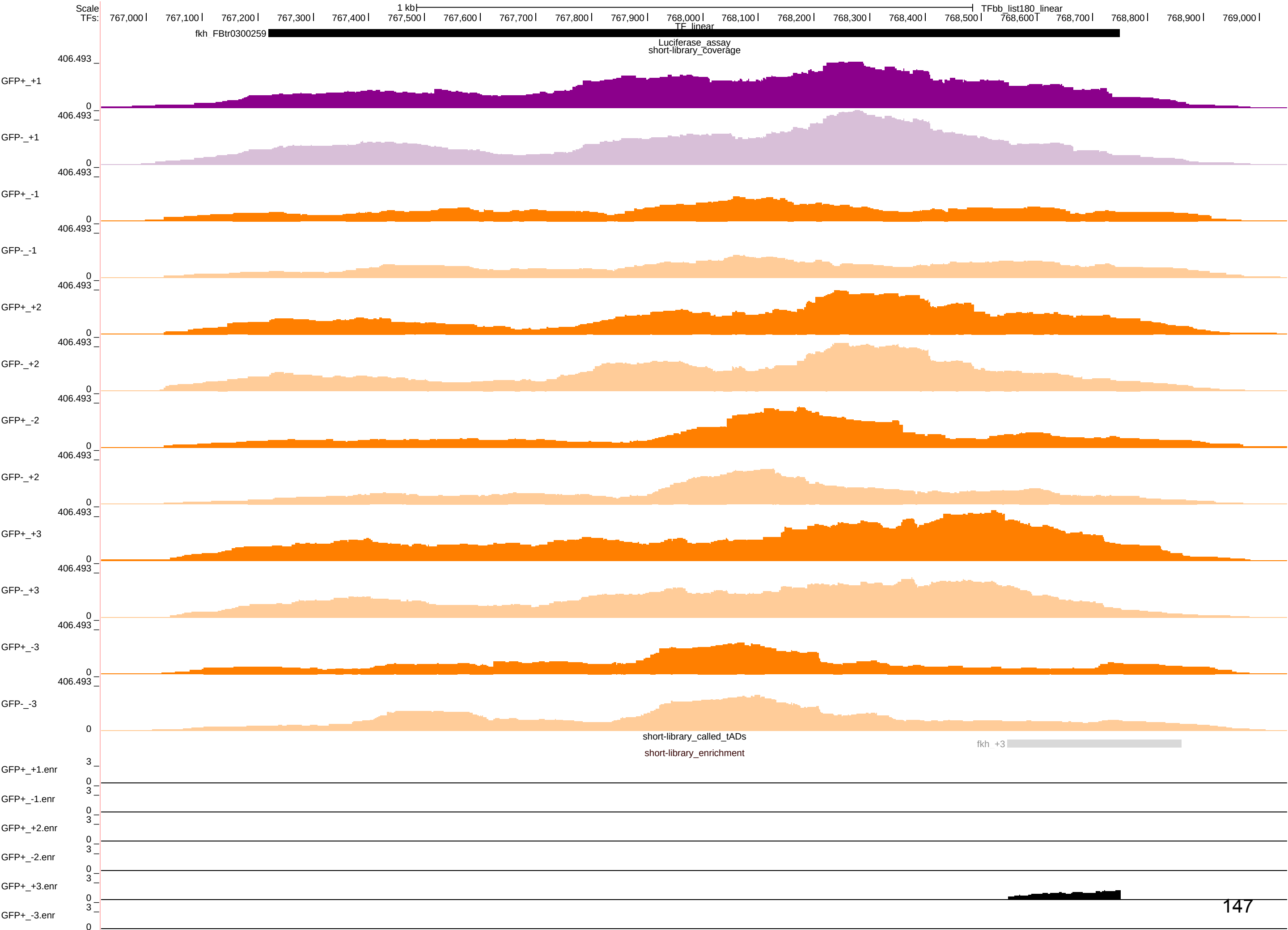
Scale
TFs:
1 kb
TFbb_list180_linear
767,000
767,100
767,200
767,300
767,400
767,500
767,600
767,700
767,800
767,900
768,000
768,100
768,200
768,300
768,400
768,500
768,600
768,700
768,800
768,900
769,000
TF_linear
fkh_FBtr0300259
Luciferase_assay
short-library_coverage
GFP+_+1
GFP-_+1
GFP+_-1
GFP-_-1
GFP+_+2
GFP-_+2
GFP+_-2
GFP-_+2
GFP+_+3
GFP-_+3
GFP+_-3
GFP-_-3
406.493
0
short-library_called_tADs
fkh_+3
short-library_enrichment
GFP+_+1.enr
GFP+_-1.enr
GFP+_+2.enr
GFP+_-2.enr
GFP+_+3.enr
GFP+_-3.enr
3
0

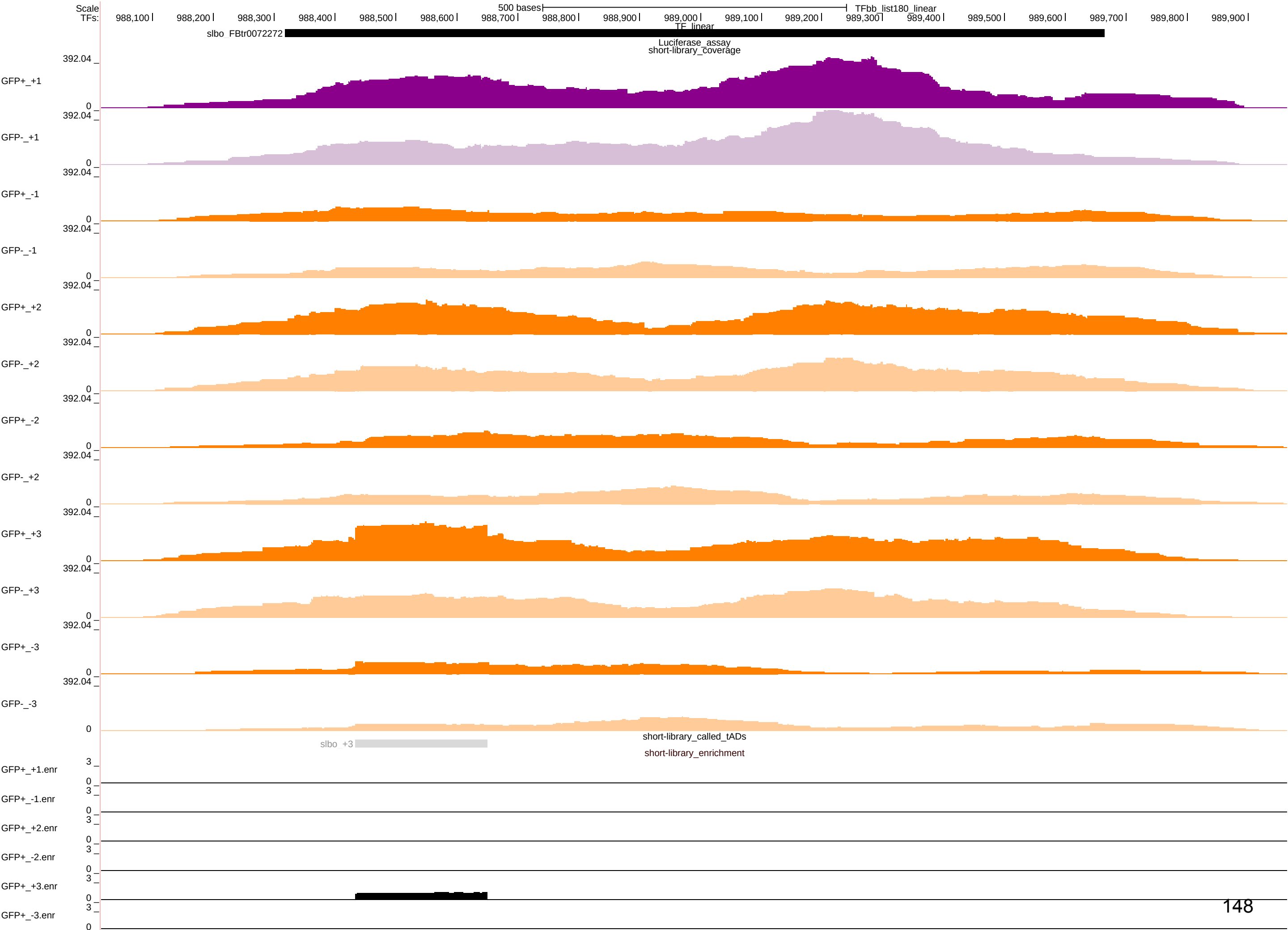
Scale
500 bases
TFbb_list180_linear
TFs:
988,100
988,200
988,300
988,400
988,500
988,600
988,700
988,800
988,900
989,000
989,100
989,200
989,300
989,400
989,500
989,600
989,700
989,800
989,900
TF_linear
slbo_FBtr0072272
Luciferase_assay
short-library_coverage
392.04 _
GFP+_+1
0 _
GFP-_+1
GFP+_-1
GFP-_-1
GFP+_+2
GFP-_+2
GFP+_-2
GFP-_+2
GFP+_+3
GFP-_+3
GFP+_-3
GFP-_-3
short-library_called_tADs
slbo_+3
short-library_enrichment
3 _
GFP+_+1.enr
GFP+_-1.enr
GFP+_+2.enr
GFP+_-2.enr
GFP+_+3.enr
GFP+_-3.enr

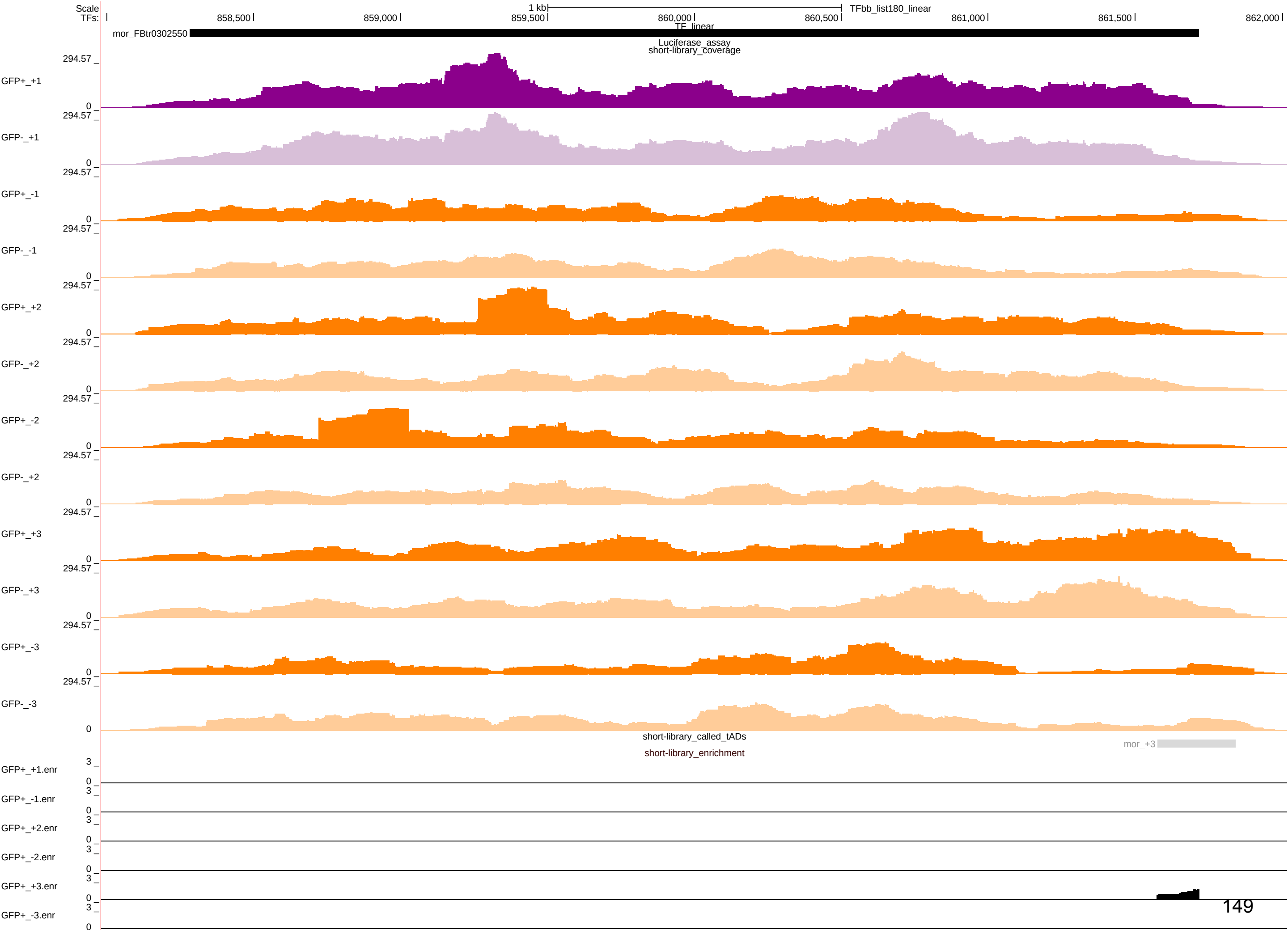
Scale
TFs:
1 kb
858,500
859,000
859,500
860,000
860,500
861,000
861,500
862,000
TFbb_list180_linear
TF_linear
mor_FBtr0302550
Luciferase_assay
short-library_coverage
294.57 _
0 _
GFP+_+1
GFP-_+1
GFP+_-1
GFP-_-1
GFP+_+2
GFP-_+2
GFP+_-2
GFP-_+2
GFP+_+3
GFP-_+3
GFP+_-3
GFP-_-3
short-library_called_tADs
mor_+3
short-library_enrichment
3 _
GFP+_+1.enr
GFP+_-1.enr
GFP+_+2.enr
GFP+_-2.enr
GFP+_+3.enr
GFP+_-3.enr

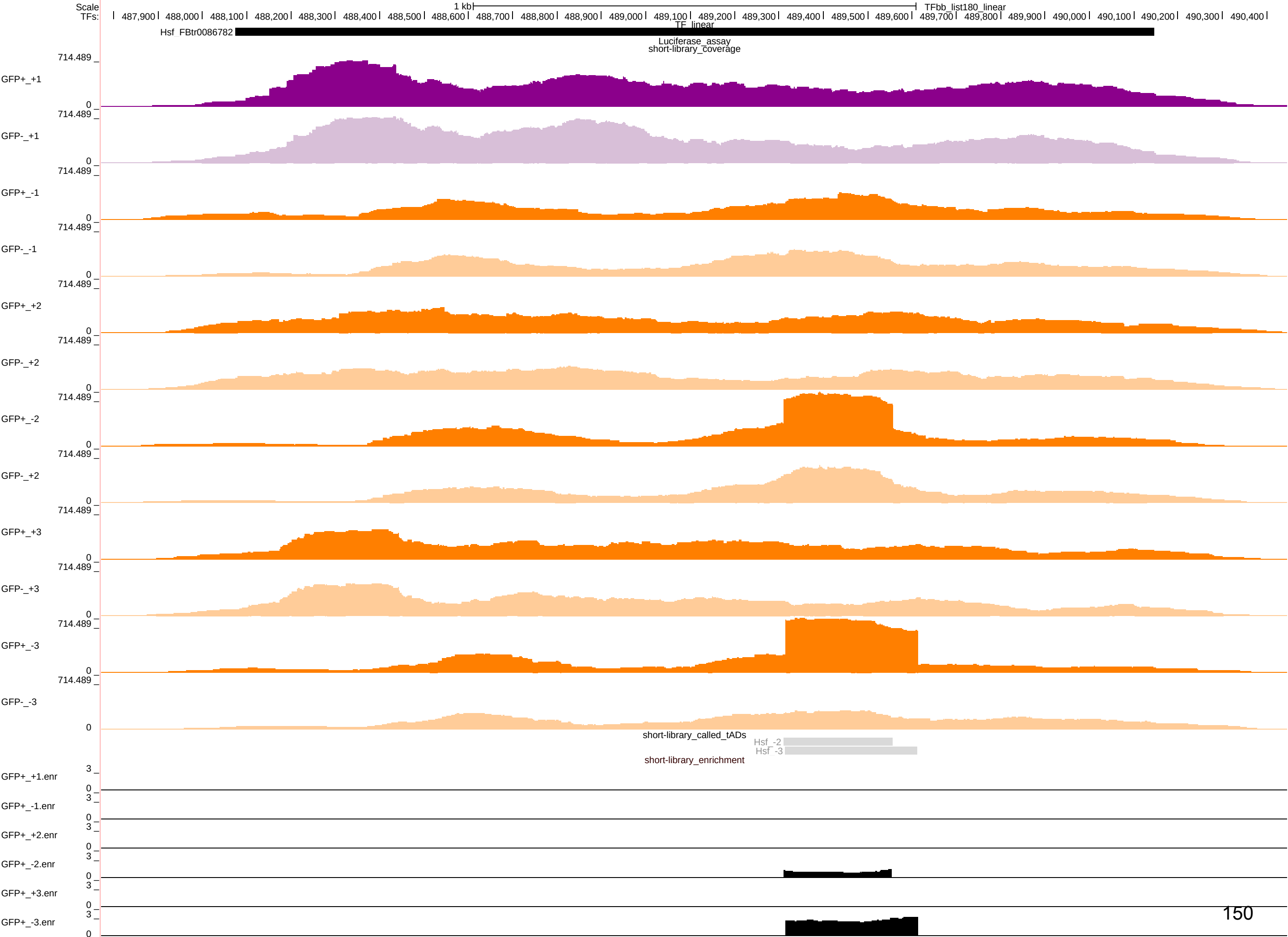

Scale
1 kb
TFbb_list180_linear
TFs:
487,900
488,000
488,100
488,200
488,300
488,400
488,500
488,600
488,700
488,800
488,900
489,000
489,100
489,200
489,300
489,400
489,500
489,600
489,700
489,800
489,900
490,000
490,100
490,200
490,300
490,400
TF_linear
Hsf_FBtr0086782
Luciferase_assay
short-library_coverage
714.489
0
GFP+_+1
GFP-_+1
GFP+_-1
GFP-_-1
GFP+_+2
GFP-_+2
GFP+_-2
GFP-_+2
GFP+_+3
GFP-_+3
GFP+_-3
GFP-_-3
short-library_called_tADs
Hsf_-2
Hsf_-3
short-library_enrichment
3
0
GFP+_+1.enr
GFP+_-1.enr
GFP+_+2.enr
GFP+_-2.enr
GFP+_+3.enr
GFP+_-3.enr

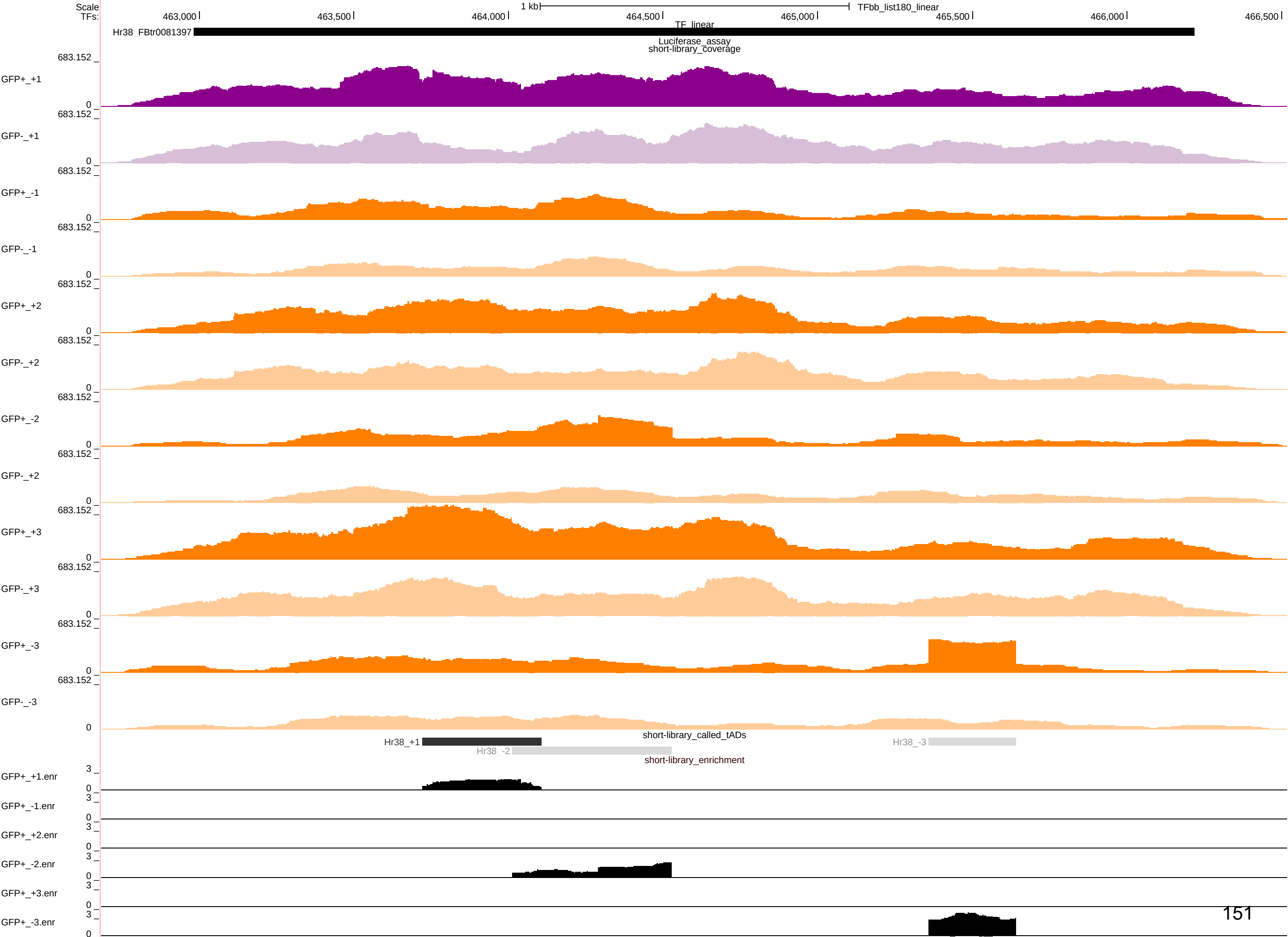
Scale
TFs:
1 kb
TFbb_list180_linear
463,000
463,500
464,000
464,500
465,000
465,500
466,000
466,500
TF_linear
Hr38_FBtr0081397
Luciferase_assay
short-library_coverage
GFP+_+1
GFP-_+1
GFP+_-1
GFP-_-1
GFP+_+2
GFP-_+2
GFP+_-2
GFP-_+2
GFP+_+3
GFP-_+3
GFP+_-3
GFP-_-3
683.152
0
short-library_called_tADs
Hr38_+1
Hr38_-2
Hr38_-3
short-library_enrichment
GFP+_+1.enr
GFP+_-1.enr
GFP+_+2.enr
GFP+_-2.enr
GFP+_+3.enr
GFP+_-3.enr
3
0

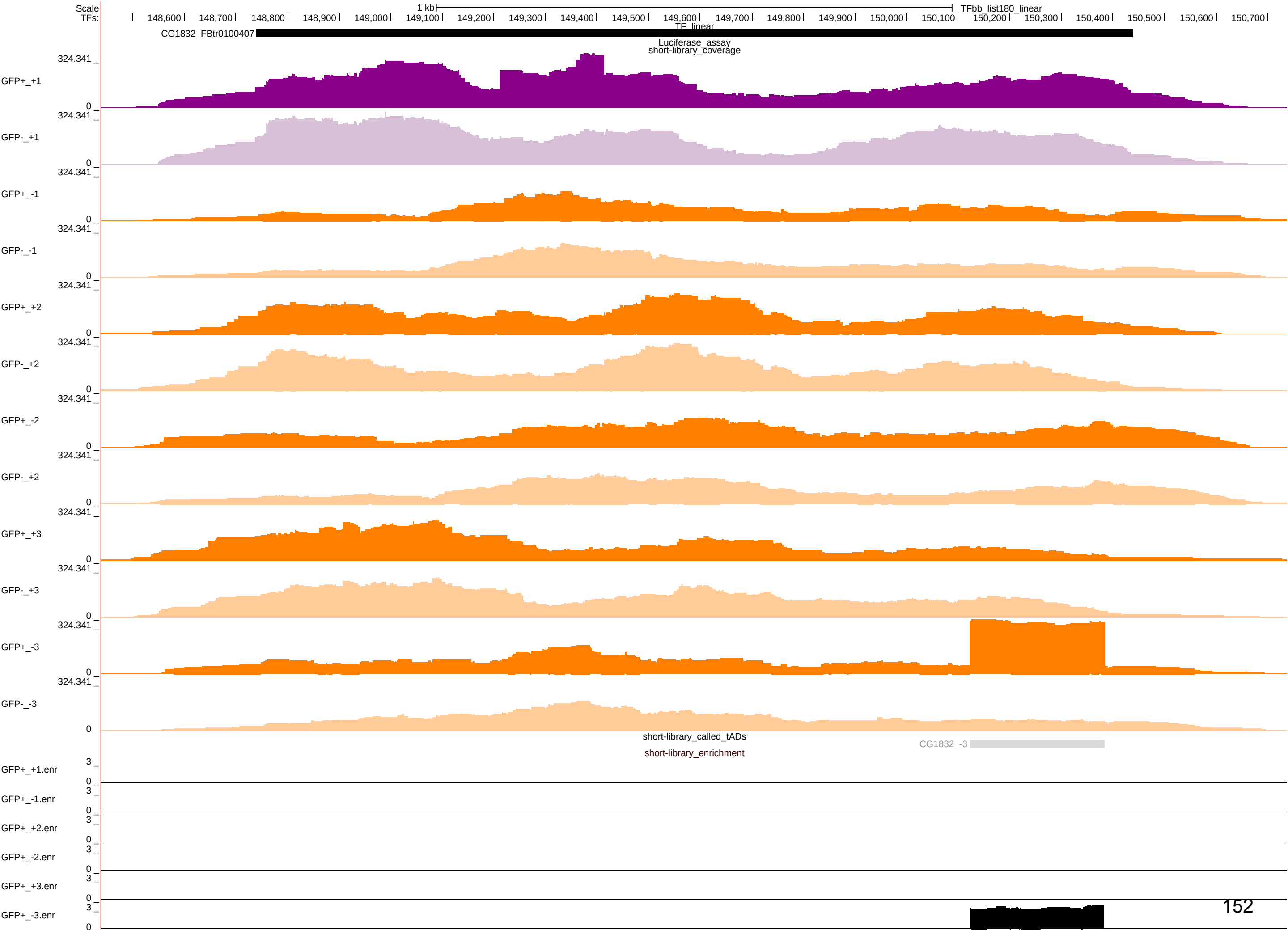
Scale
TFs:
1 kb
TFbb_list180_linear
148,600
148,700
148,800
148,900
149,000
149,100
149,200
149,300
149,400
149,500
149,600
149,700
149,800
149,900
150,000
150,100
150,200
150,300
150,400
150,500
150,600
150,700
TF_linear
CG1832 FBtr0100407
Luciferase_assay
short-library_coverage
GFP+_+1
GFP-_+1
GFP+_-1
GFP-_-1
GFP+_+2
GFP-_+2
GFP+_-2
GFP-_+2
GFP+_+3
GFP-_+3
GFP+_-3
GFP-_-3
324.341
0
short-library_called_tADs
CG1832_-3
short-library_enrichment
GFP+_+1.enr
GFP+_-1.enr
GFP+_+2.enr
GFP+_-2.enr
GFP+_+3.enr
GFP+_-3.enr
3
0

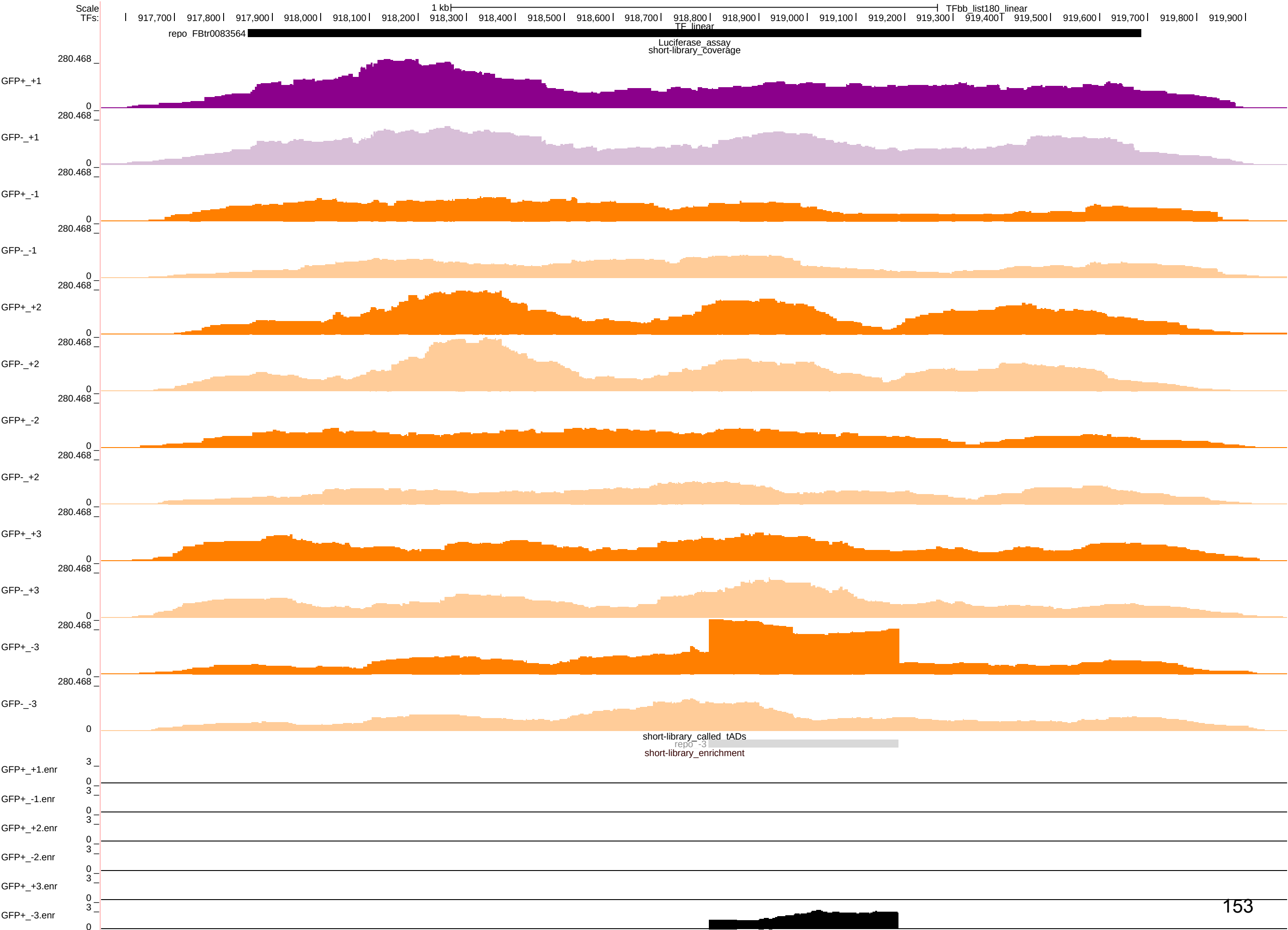

Scale
1 kb
TFbb_list180_linear
TFs:
917,700
917,800
917,900
918,000
918,100
918,200
918,300
918,400
918,500
918,600
918,700
918,800
918,900
919,000
919,100
919,200
919,300
919,400
919,500
919,600
919,700
919,800
919,900
TF_linear
repo FBtr0083564
Luciferase_assay
short-library_coverage
280.468
0
GFP+_+1
GFP-_+1
GFP+_-1
GFP-_-1
GFP+_+2
GFP-_+2
GFP+_-2
GFP-_+2
GFP+_+3
GFP-_+3
GFP+_-3
GFP-_-3
short-library_called_tADs
repo_-3
short-library_enrichment
3
GFP+_+1.enr
GFP+_-1.enr
GFP+_+2.enr
GFP+_-2.enr
GFP+_+3.enr
GFP+_-3.enr

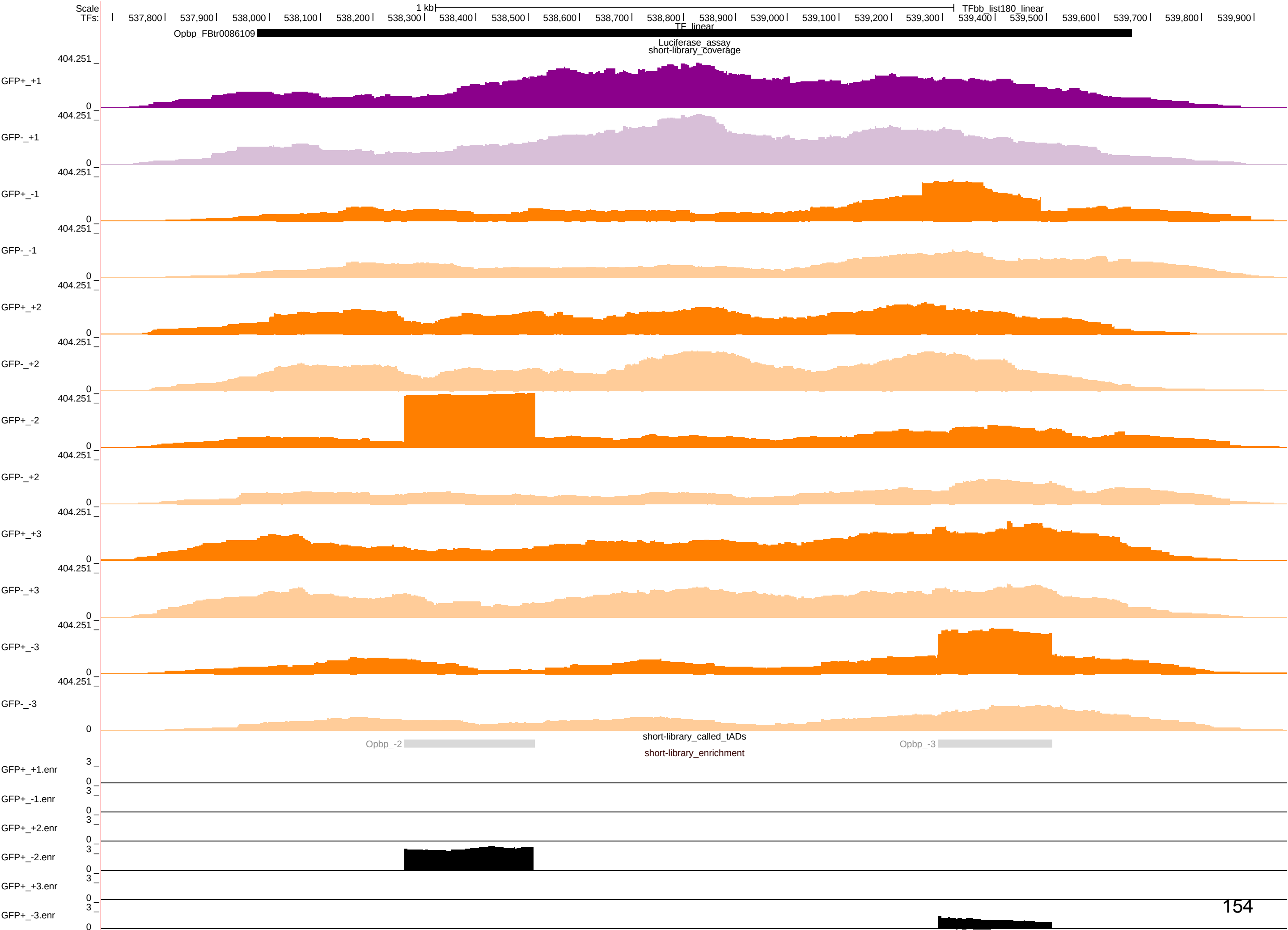
Scale
TFs:
1 kb
TFbb_list180_linear
537,800
537,900
538,000
538,100
538,200
538,300
538,400
538,500
538,600
538,700
538,800
538,900
539,000
539,100
539,200
539,300
539,400
539,500
539,600
539,700
539,800
539,900
TF_linear
Opbp FBtr0086109
Luciferase_assay
short-library_coverage
GFP+_+1
GFP-_+1
GFP+_-1
GFP-_-1
GFP+_+2
GFP-_+2
GFP+_-2
GFP-_+2
GFP+_+3
GFP-_+3
GFP+_-3
GFP-_-3
404.251
0
short-library_called_tADs
Opbp -2
Opbp -3
short-library_enrichment
GFP+_+1.enr
GFP+_-1.enr
GFP+_+2.enr
GFP+_-2.enr
GFP+_+3.enr
GFP+_-3.enr
3
0

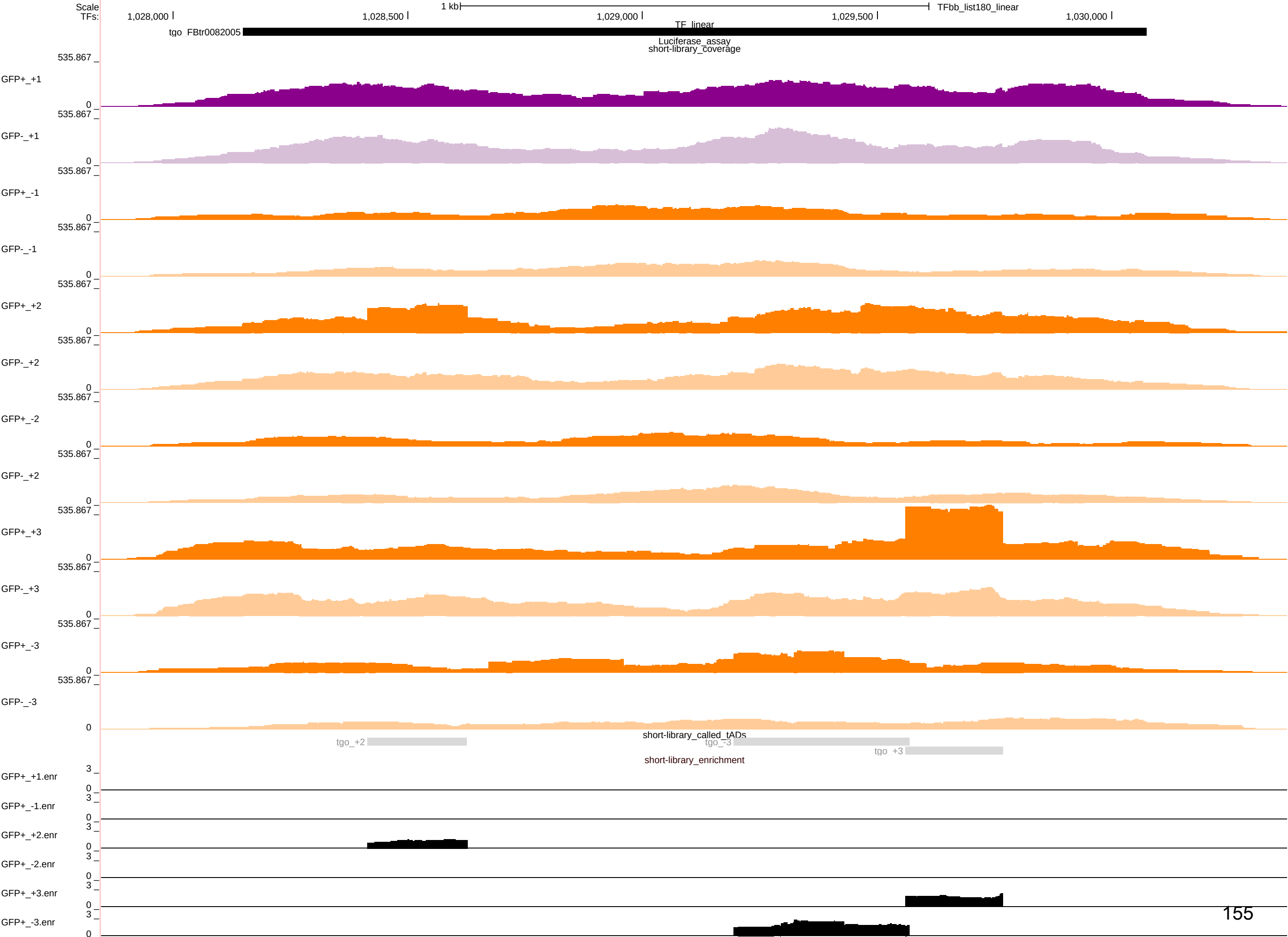

Scale
TFs:
1 kb
TFbb_list180_linear
1,028,000
1,028,500
1,029,000
1,029,500
1,030,000
TF_linear
tgo_FBtr0082005
Luciferase_assay
short-library_coverage
535.867
GFP+_+1
0
GFP-_+1
GFP+_-1
GFP-_-1
GFP+_+2
GFP-_+2
GFP+_-2
GFP-_+2
GFP+_+3
GFP-_+3
GFP+_-3
GFP-_-3
short-library_called_tADs
tgo_+2
tgo_-3
tgo_+3
short-library_enrichment
3
GFP+_+1.enr
GFP+_-1.enr
GFP+_+2.enr
GFP+_-2.enr
GFP+_+3.enr
GFP+_-3.enr

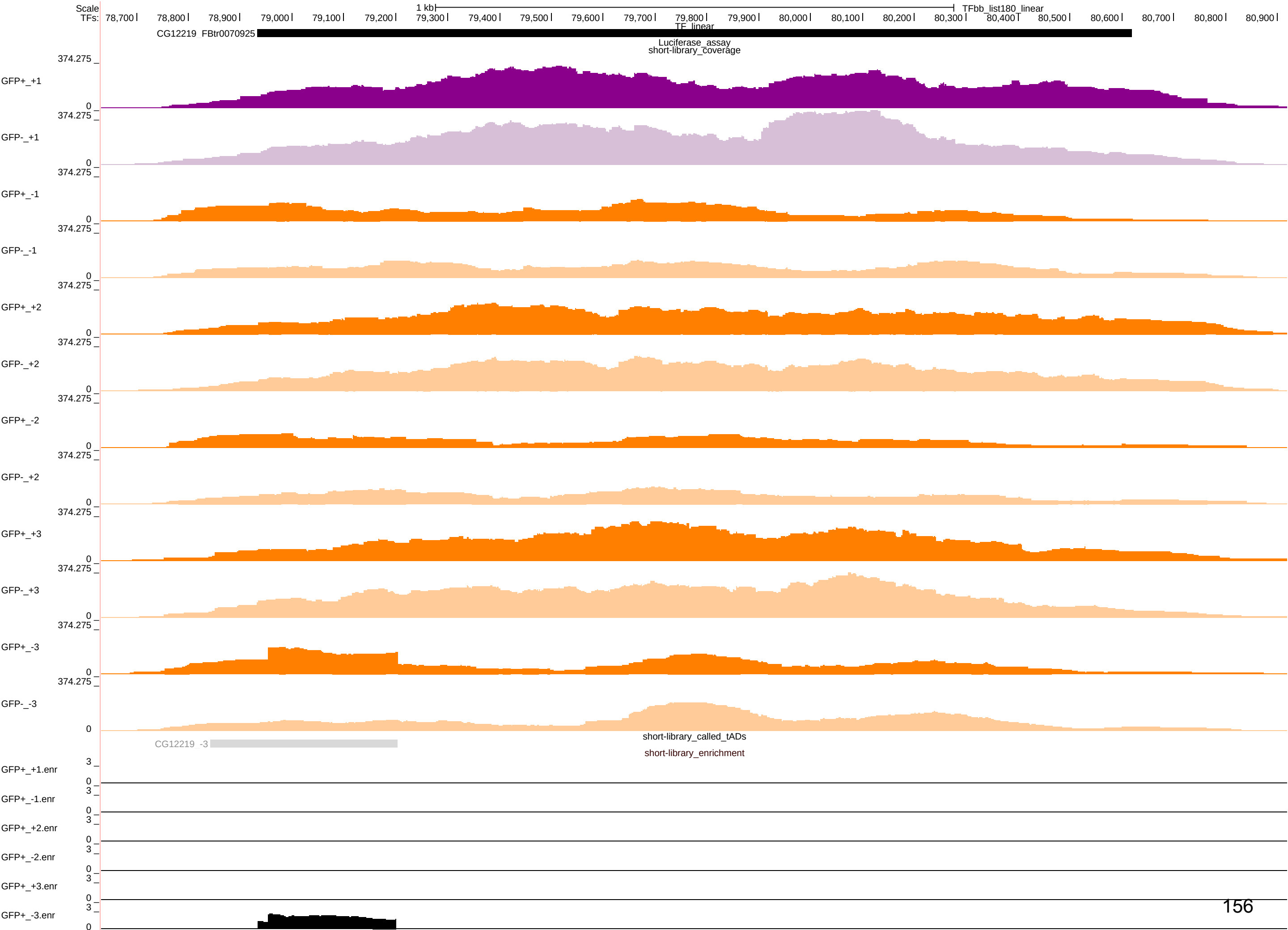

Scale
1 kb
TFbb_list180_linear
TFs:
78,700
78,800
78,900
79,000
79,100
79,200
79,300
79,400
79,500
79,600
79,700
79,800
79,900
80,000
80,100
80,200
80,300
80,400
80,500
80,600
80,700
80,800
80,900
TF_linear
CG12219 FBtr0070925
Luciferase_assay
short-library_coverage
374.275
0
GFP+_+1
GFP-_+1
GFP+_-1
GFP-_-1
GFP+_+2
GFP-_+2
GFP+_-2
GFP-_+2
GFP+_+3
GFP-_+3
GFP+_-3
GFP-_-3
short-library_called_tADs
CG12219 -3
short-library_enrichment
3
GFP+_+1.enr
GFP+_-1.enr
GFP+_+2.enr
GFP+_-2.enr
GFP+_+3.enr
GFP+_-3.enr

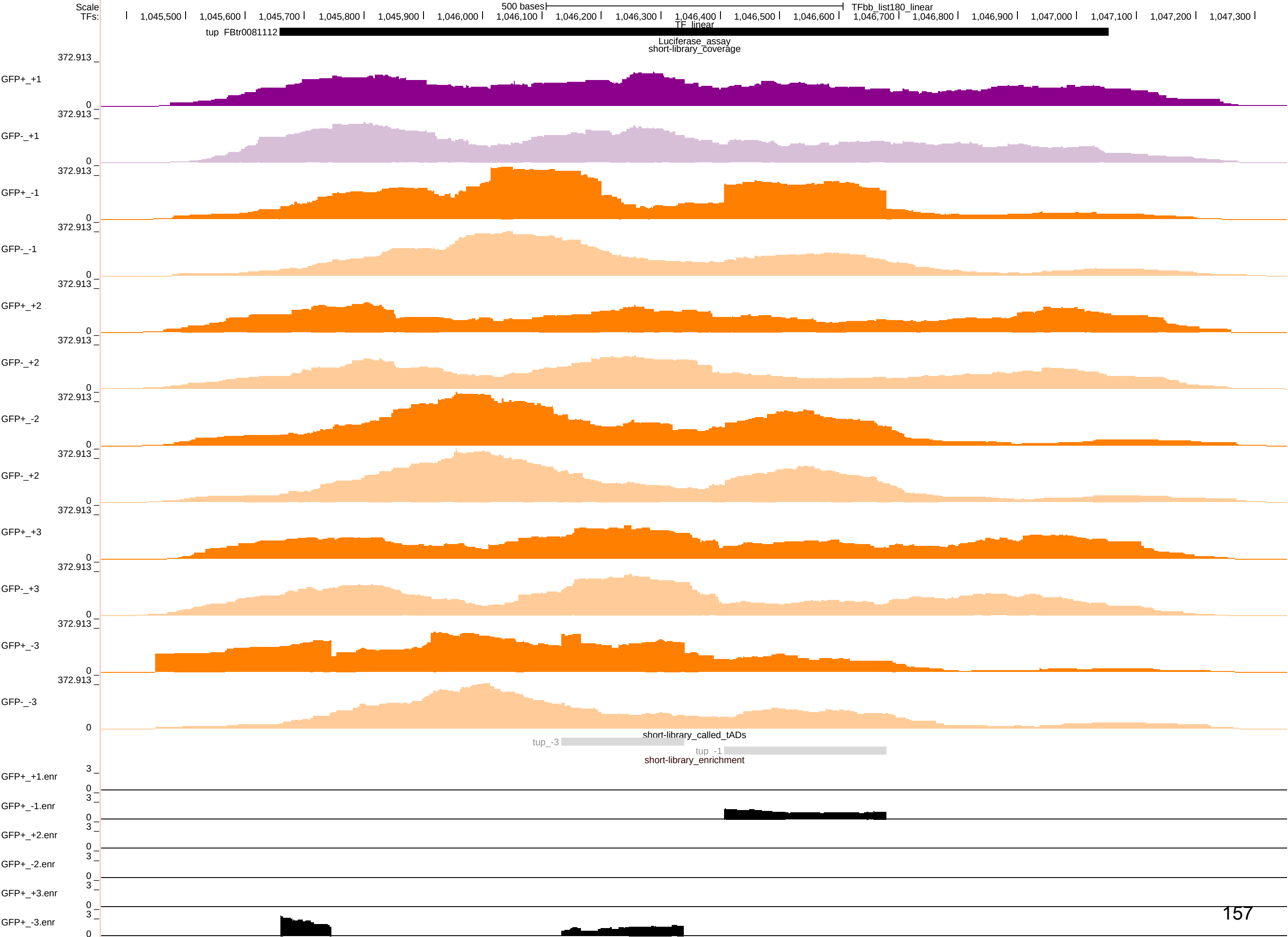
Scale
TFs:
500 bases
TFbb_list180_linear
1,045,500
1,045,600
1,045,700
1,045,800
1,045,900
1,046,000
1,046,100
1,046,200
1,046,300
1,046,400
1,046,500
1,046,600
1,046,700
1,046,800
1,046,900
1,047,000
1,047,100
1,047,200
1,047,300
TF_linear
tup FBtr0081112
Luciferase_assay
short-library_coverage
372.913
0
GFP+_+1
GFP-_+1
GFP+_-1
GFP-_-1
GFP+_+2
GFP-_+2
GFP+_-2
GFP-_+2
GFP+_+3
GFP-_+3
GFP+_-3
GFP-_-3
short-library_called_tADs
tup_-3
tup_-1
short-library_enrichment
3
GFP+_+1.enr
GFP+_-1.enr
GFP+_+2.enr
GFP+_-2.enr
GFP+_+3.enr
GFP+_-3.enr

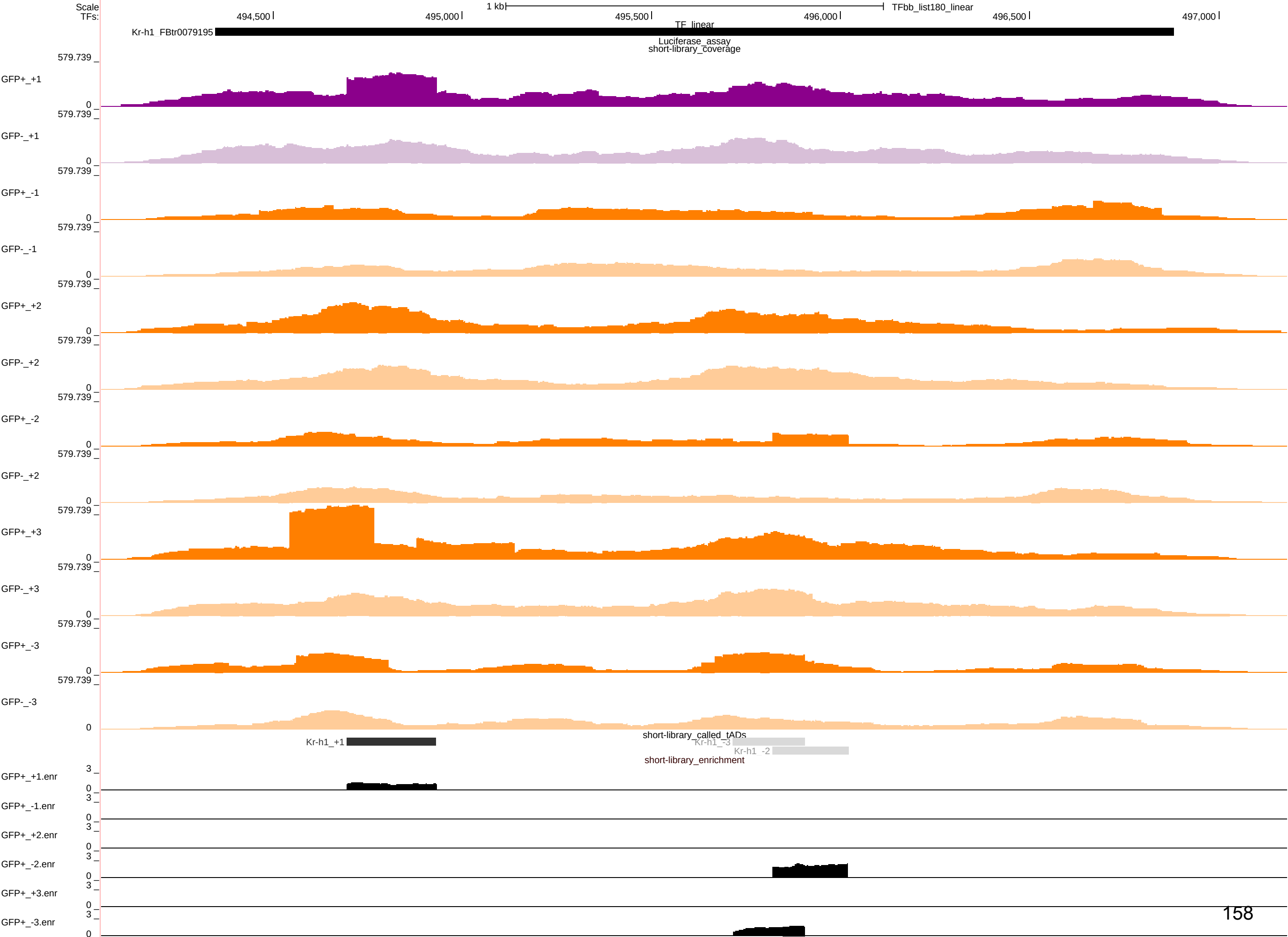
Scale
TFs:
1 kb
TFbb_list180_linear
494,500
495,000
495,500
496,000
496,500
497,000
TF_linear
Kr-h1 FBtr0079195
Luciferase_assay
short-library_coverage
579.739 _
0 _
GFP+_+1
GFP-_+1
GFP+_-1
GFP-_-1
GFP+_+2
GFP-_+2
GFP+_-2
GFP-_+2
GFP+_+3
GFP-_+3
GFP+_-3
GFP-_-3
short-library_called_tADs
Kr-h1_+1
Kr-h1_-3
Kr-h1_-2
short-library_enrichment
3 _
GFP+_+1.enr
GFP+_-1.enr
GFP+_+2.enr
GFP+_-2.enr
GFP+_+3.enr
GFP+_-3.enr

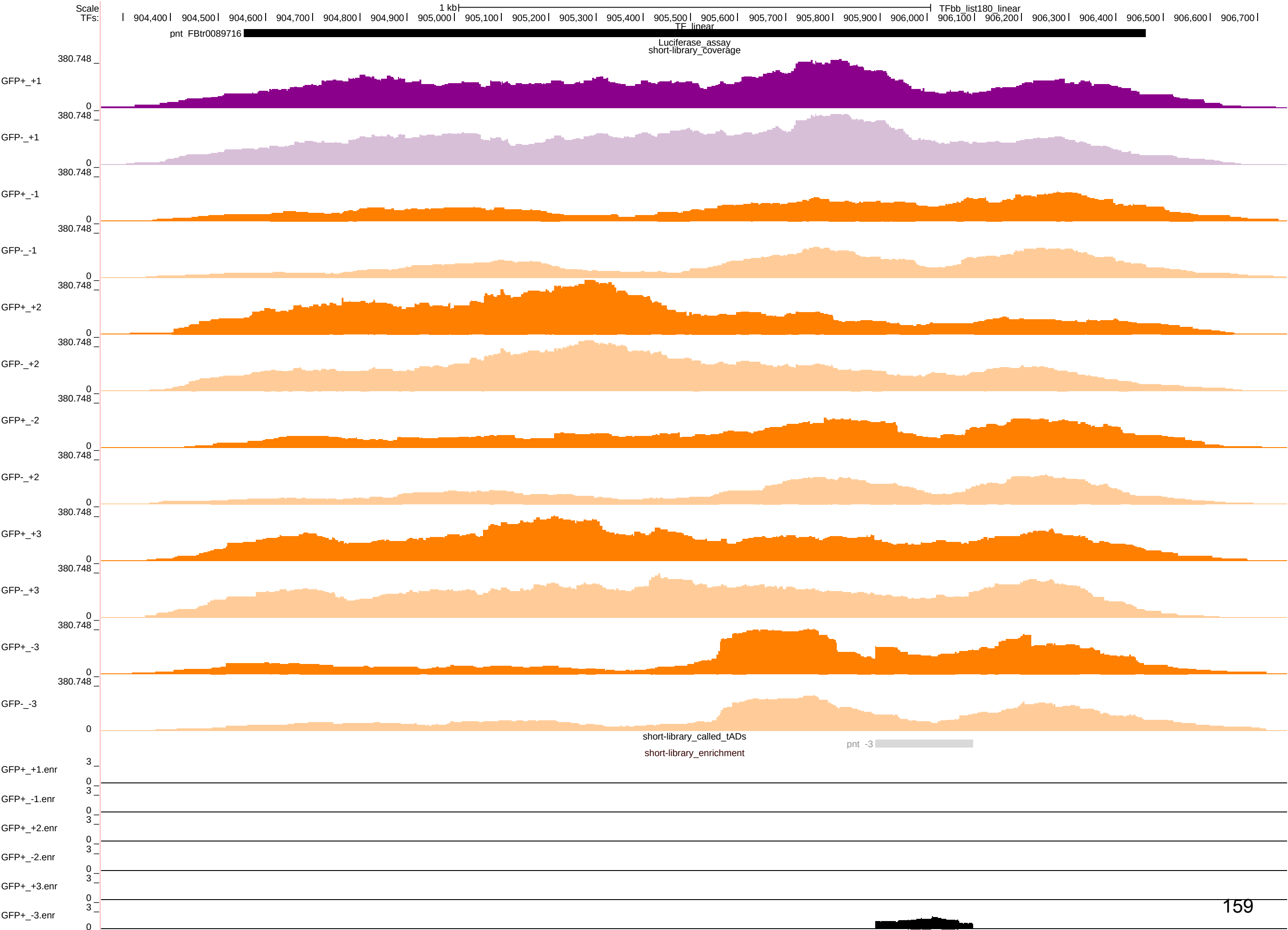

Scale
1 kb
TFs:
904,400
904,500
904,600
904,700
904,800
904,900
905,000
905,100
905,200
905,300
905,400
905,500
905,600
905,700
905,800
905,900
906,000
906,100
906,200
906,300
906,400
906,500
906,600
906,700
TFbb_list180_linear
TF_linear
pnt FBtr0089716
Luciferase_assay
short-library_coverage
380.748
0
GFP+_+1
GFP-_+1
GFP+_-1
GFP-_-1
GFP+_+2
GFP-_+2
GFP+_-2
GFP-_+2
GFP+_+3
GFP-_+3
GFP+_-3
GFP-_-3
short-library_called_tADs
pnt -3
short-library_enrichment
3
GFP+_+1.enr
GFP+_-1.enr
GFP+_+2.enr
GFP+_-2.enr
GFP+_+3.enr
GFP+_-3.enr

Appendix Figure S3

18 long-fragment library native (+1) reading-frame tADs

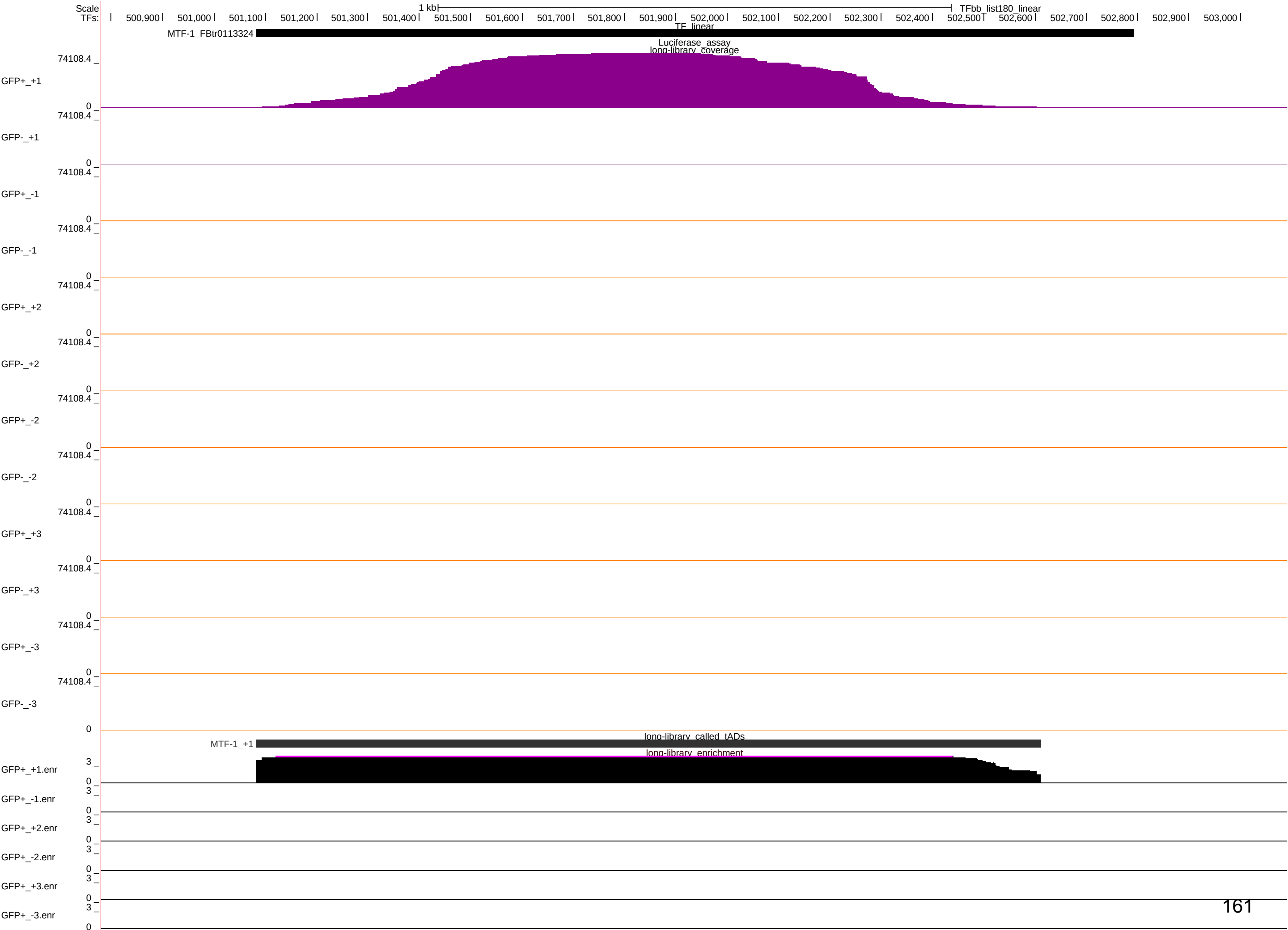
Scale
TFs:
1 kb
TFbb_list180_linear
500,900
501,000
501,100
501,200
501,300
501,400
501,500
501,600
501,700
501,800
501,900
502,000
502,100
502,200
502,300
502,400
502,500
502,600
502,700
502,800
502,900
503,000
TF_linear
MTF-1_FBtr0113324
Luciferase_assay
long-library_coverage
74108.4
0
GFP+_+1
GFP-_+1
GFP+_-1
GFP-_-1
GFP+_+2
GFP-_+2
GFP+_-2
GFP-_-2
GFP+_+3
GFP-_+3
GFP+_-3
GFP-_-3
long-library_called_tADs
MTF-1_+1
long-library_enrichment
3
GFP+_+1.enr
GFP+_-1.enr
GFP+_+2.enr
GFP+_-2.enr
GFP+_+3.enr
GFP+_-3.enr

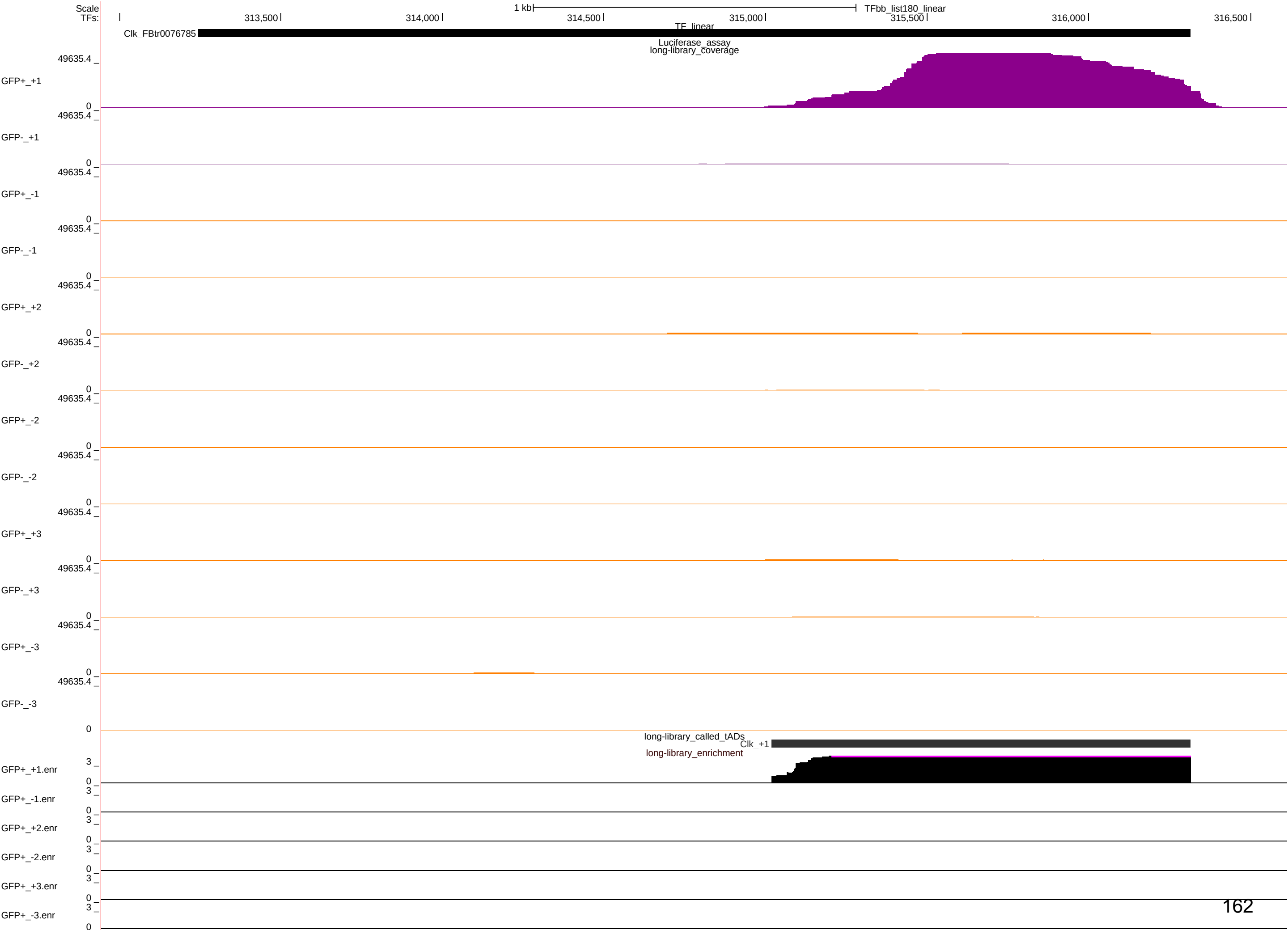

Scale
TFs:
1 kb
TFbb_list180_linear
313,500
314,000
314,500
315,000
315,500
316,000
316,500
Clk FBtr0076785
TF_linear
Luciferase_assay
long-library_coverage
49635.4
0
GFP+_+1
GFP-_+1
GFP+_-1
GFP-_-1
GFP+_+2
GFP-_+2
GFP+_-2
GFP-_-2
GFP+_+3
GFP-_+3
GFP+_-3
GFP-_-3
long-library_called_tADs
Clk_+1
long-library_enrichment
3
GFP+_+1.enr
GFP+_-1.enr
GFP+_+2.enr
GFP+_-2.enr
GFP+_+3.enr
GFP+_-3.enr

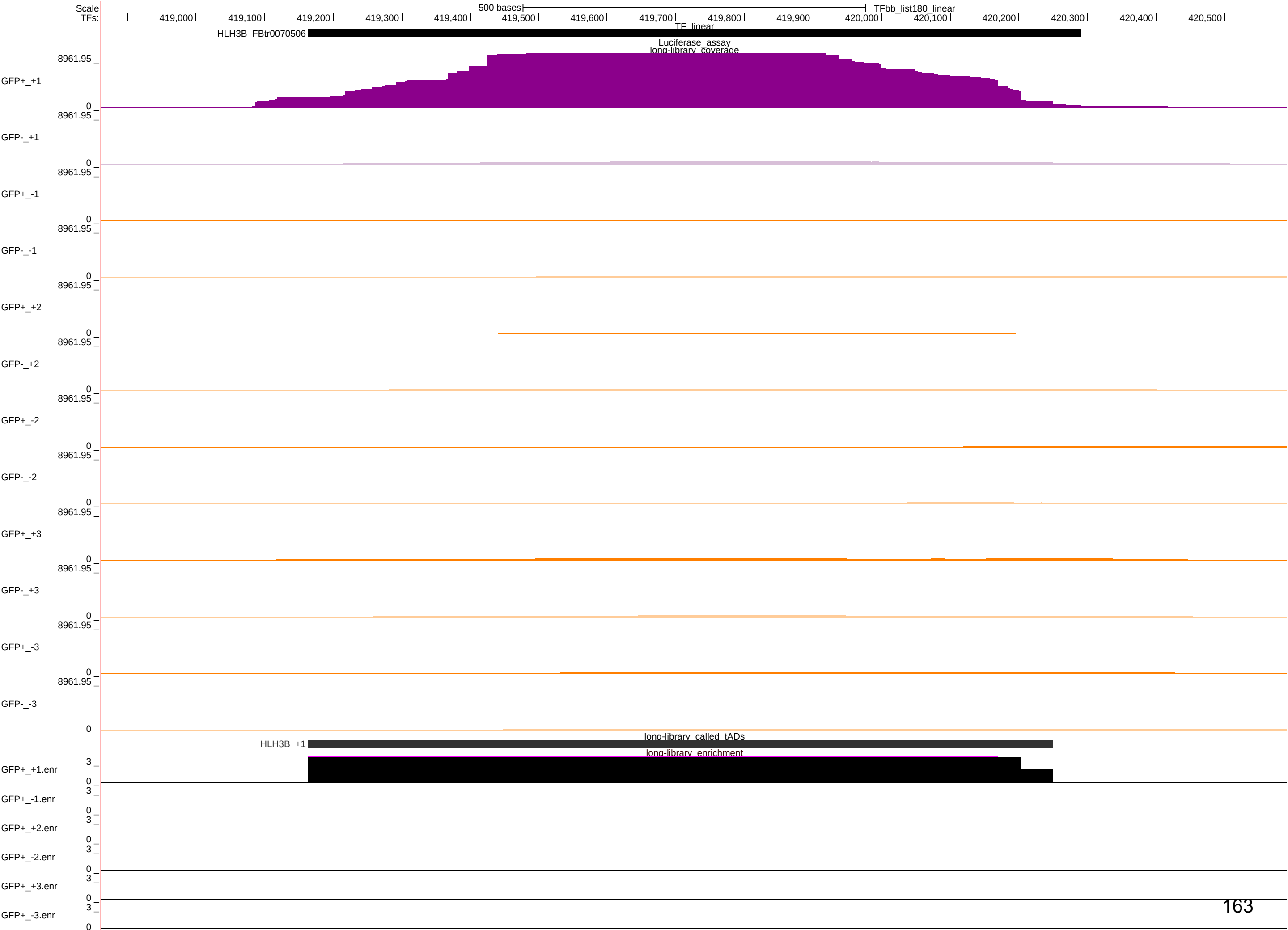
Scale
TFs:
500 bases
TFbb_list180_linear
419,000
419,100
419,200
419,300
419,400
419,500
419,600
419,700
419,800
419,900
420,000
420,100
420,200
420,300
420,400
420,500
TF_linear
HLH3B_FBtr0070506
Luciferase_assay
long-library_coverage
8961.95
0
GFP+_+1
GFP-_+1
GFP+_-1
GFP-_-1
GFP+_+2
GFP-_+2
GFP+_-2
GFP-_-2
GFP+_+3
GFP-_+3
GFP+_-3
GFP-_-3
long-library_called_tADs
HLH3B_+1
long-library_enrichment
3
GFP+_+1.enr
GFP+_-1.enr
GFP+_+2.enr
GFP+_-2.enr
GFP+_+3.enr
GFP+_-3.enr

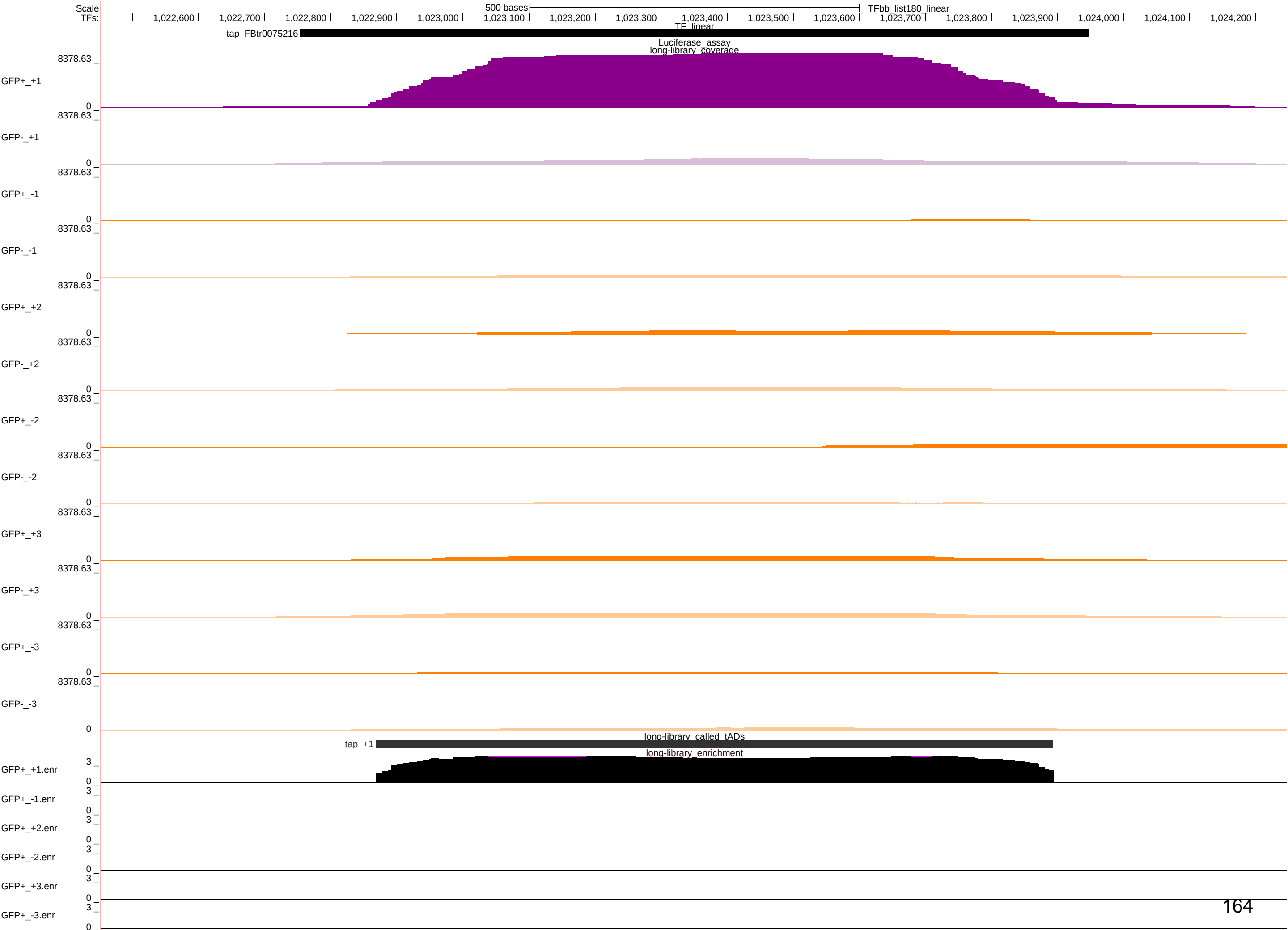
Scale
TFs:
500 bases
TFbb_list180_linear
1,022,600
1,022,700
1,022,800
1,022,900
1,023,000
1,023,100
1,023,200
1,023,300
1,023,400
1,023,500
1,023,600
1,023,700
1,023,800
1,023,900
1,024,000
1,024,100
1,024,200
TF_linear
tap_FBtr0075216
Luciferase_assay
long-library_coverage
GFP+_+1
GFP-_+1
GFP+_-1
GFP-_-1
GFP+_+2
GFP-_+2
GFP+_-2
GFP-_-2
GFP+_+3
GFP-_+3
GFP+_-3
GFP-_-3
8378.63
0
long-library_called_tADs
tap_+1
long-library_enrichment
GFP+_+1.enr
GFP+_-1.enr
GFP+_+2.enr
GFP+_-2.enr
GFP+_+3.enr
GFP+_-3.enr
3
0

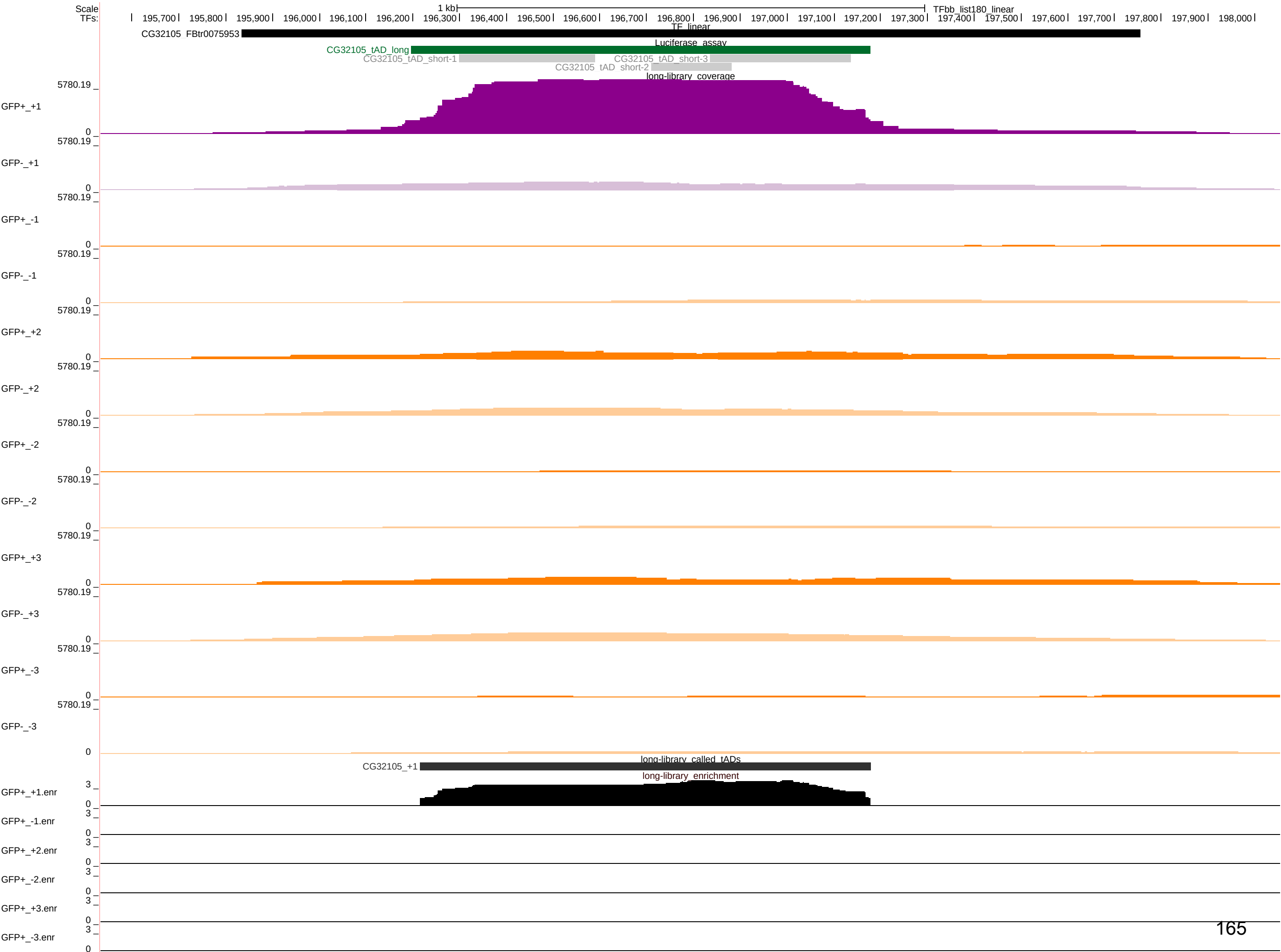
Scale
1 kb
TFs:
195,700
195,800
195,900
196,000
196,100
196,200
196,300
196,400
196,500
196,600
196,700
196,800
196,900
197,000
197,100
197,200
197,300
197,400
197,500
197,600
197,700
197,800
197,900
198,000
TFbb_list180_linear
TF_linear
CG32105_FBtr0075953
Luciferase_assay
CG32105_tAD_long
CG32105_tAD_short-1
CG32105_tAD_short-3
CG32105_tAD_short-2
long-library_coverage
5780.19
0
GFP+_+1
GFP-_+1
GFP+_-1
GFP-_-1
GFP+_+2
GFP-_+2
GFP+_-2
GFP-_-2
GFP+_+3
GFP-_+3
GFP+_-3
GFP-_-3
long-library_called_tADs
CG32105_+1
long-library_enrichment
3
GFP+_+1.enr
GFP+_-1.enr
GFP+_+2.enr
GFP+_-2.enr
GFP+_+3.enr
GFP+_-3.enr

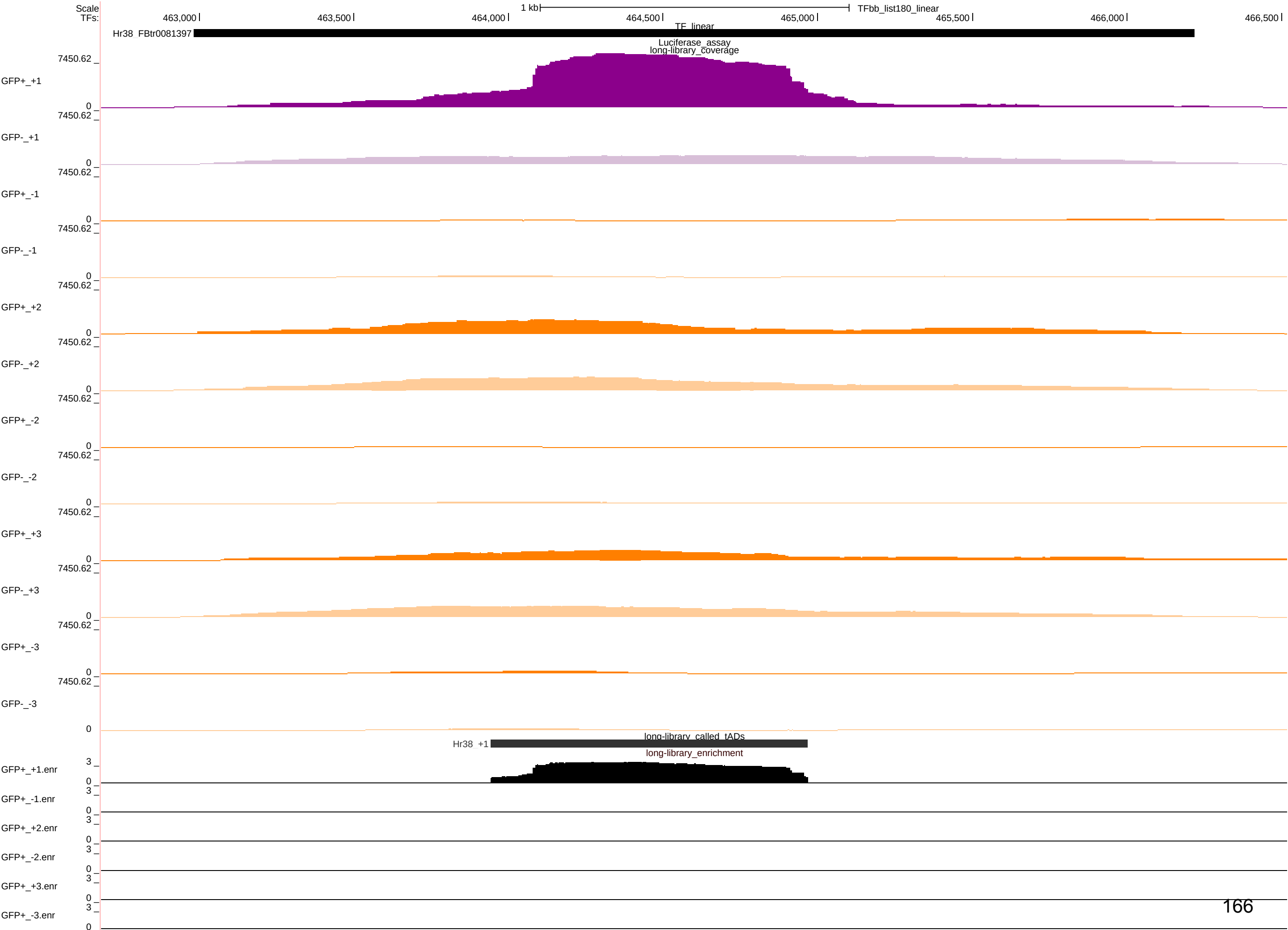
Scale
TFs:
1 kb
TFbb_list180_linear
463,000
463,500
464,000
464,500
465,000
465,500
466,000
466,500
TF_linear
Hr38_FBtr0081397
Luciferase_assay
long-library_coverage
7450.62
0
GFP+_+1
GFP-_+1
GFP+_-1
GFP-_-1
GFP+_+2
GFP-_+2
GFP+_-2
GFP-_-2
GFP+_+3
GFP-_+3
GFP+_-3
GFP-_-3
long-library_called_tADs
Hr38_+1
long-library_enrichment
3
GFP+_+1.enr
GFP+_-1.enr
GFP+_+2.enr
GFP+_-2.enr
GFP+_+3.enr
GFP+_-3.enr

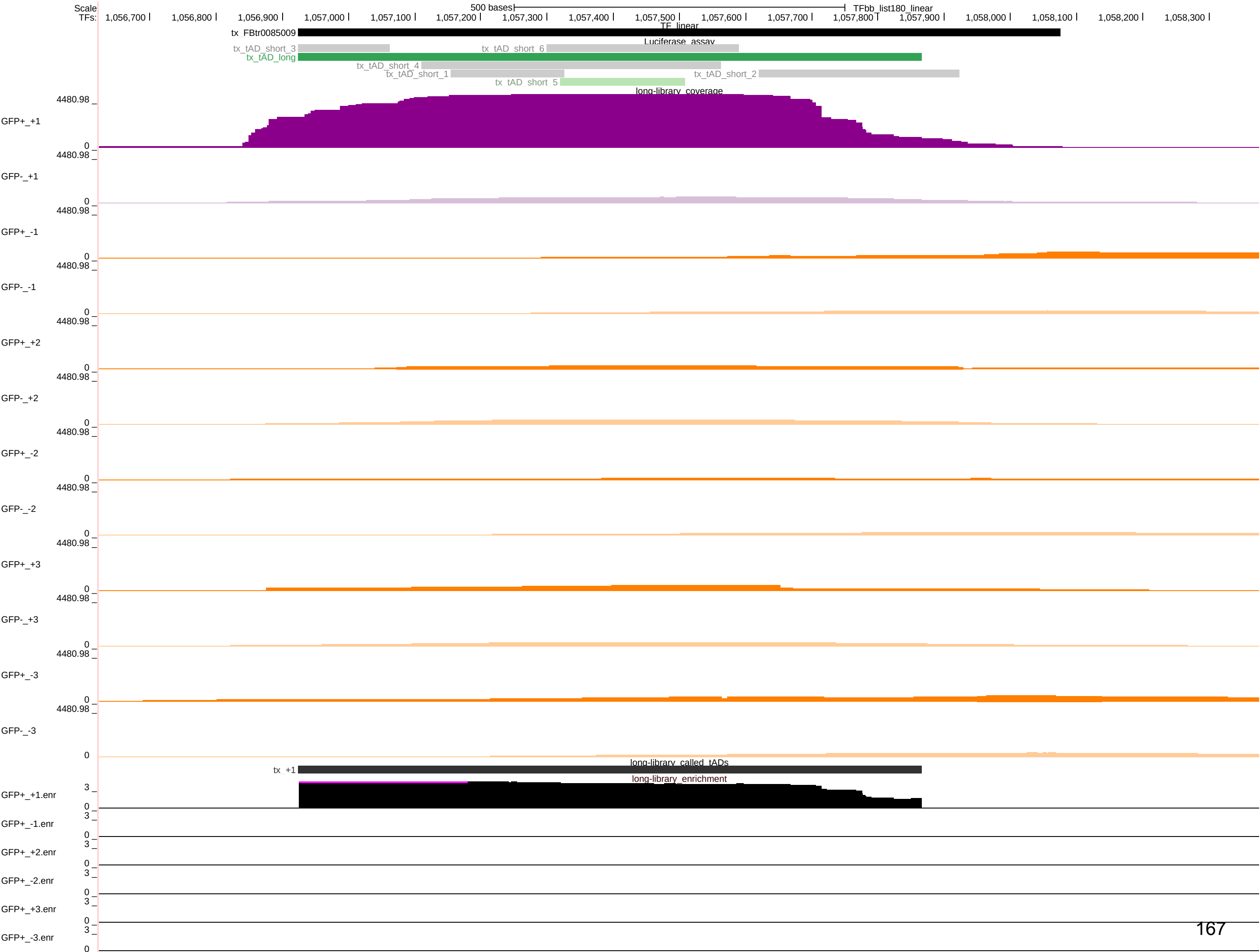
Scale
TFs:
500 bases
TFbb_list180_linear
1,056,700
1,056,800
1,056,900
1,057,000
1,057,100
1,057,200
1,057,300
1,057,400
1,057,500
1,057,600
1,057,700
1,057,800
1,057,900
1,058,000
1,058,100
1,058,200
1,058,300
TF_linear
tx_FBtr0085009
Luciferase_assay
tx_tAD_short_3
tx_tAD_short_6
tx_tAD_long
tx_tAD_short_4
tx_tAD_short_1
tx_tAD_short_2
tx_tAD_short_5
long-library_coverage
GFP+_+1
GFP-_+1
GFP+_-1
GFP-_-1
GFP+_+2
GFP-_+2
GFP+_-2
GFP-_-2
GFP+_+3
GFP-_+3
GFP+_-3
GFP-_-3
4480.98
0
long-library_called_tADs
tx_+1
long-library_enrichment
GFP+_+1.enr
GFP+_-1.enr
GFP+_+2.enr
GFP+_-2.enr
GFP+_+3.enr
GFP+_-3.enr
3
0

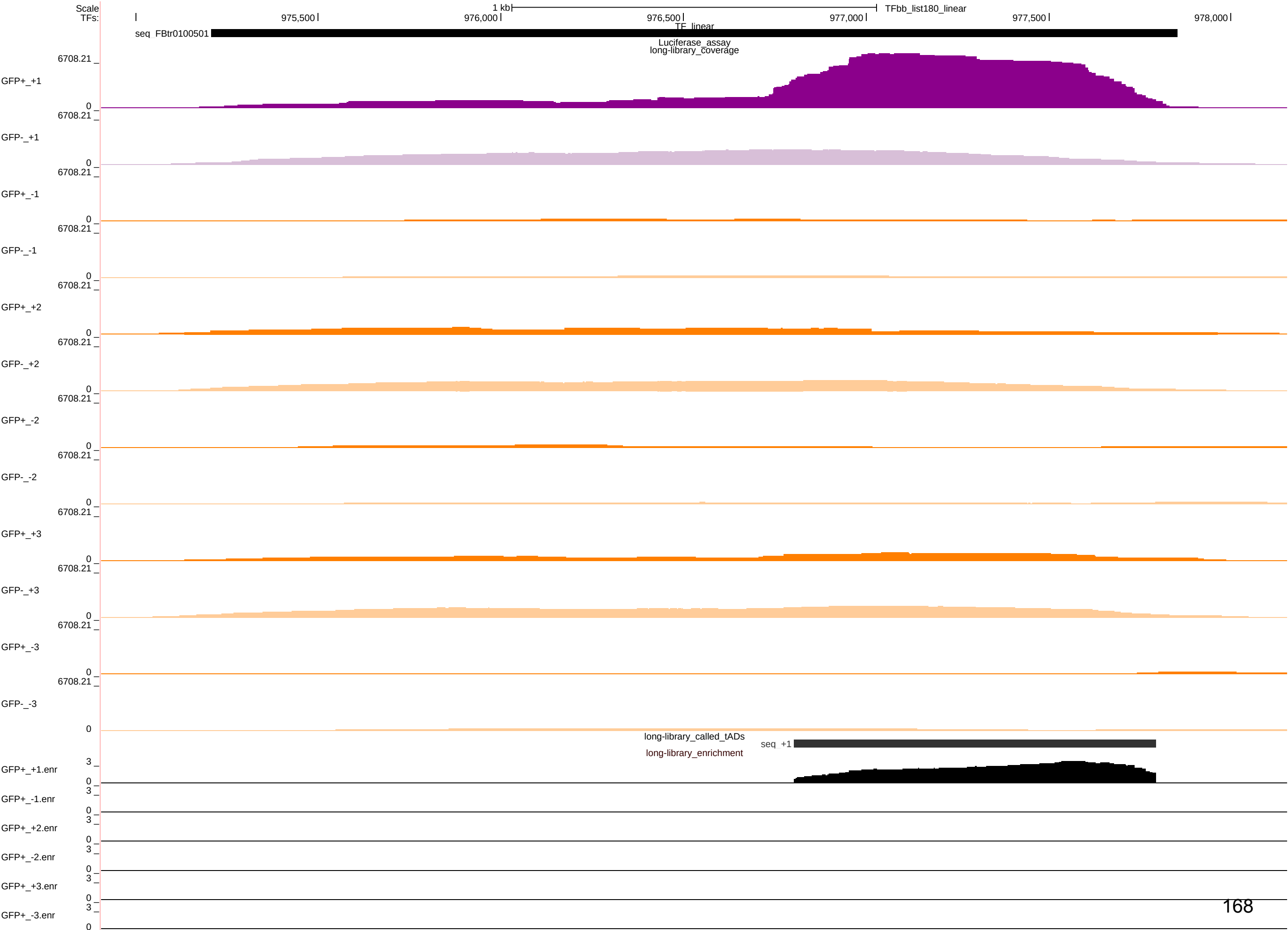
Scale
TFs:
1 kb
TFbb_list180_linear
975,500
976,000
976,500
977,000
977,500
978,000
TF_linear
seq_FBtr0100501
Luciferase_assay
long-library_coverage
GFP+_+1
GFP-_+1
GFP+_-1
GFP-_-1
GFP+_+2
GFP-_+2
GFP+_-2
GFP-_-2
GFP+_+3
GFP-_+3
GFP+_-3
GFP-_-3
6708.21
0
long-library_called_tADs
seq_+1
long-library_enrichment
GFP+_+1.enr
GFP+_-1.enr
GFP+_+2.enr
GFP+_-2.enr
GFP+_+3.enr
GFP+_-3.enr
3
0

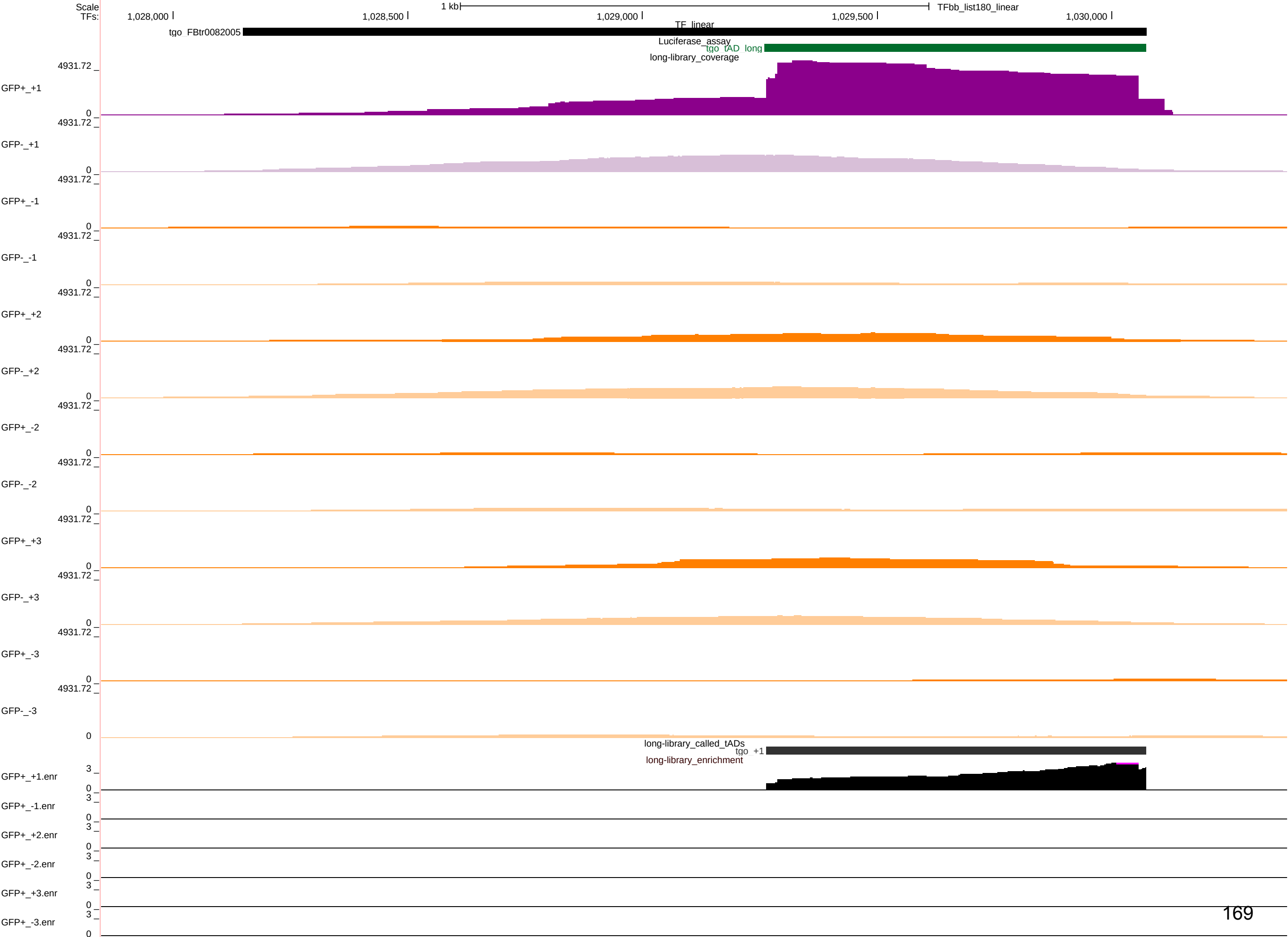
Scale
TFs:
1 kb
TFbb_list180_linear
1,028,000
1,028,500
1,029,000
1,029,500
1,030,000
TF_linear
tgo_FBtr0082005
Luciferase_assay
tgo_tAD_long
long-library_coverage
4931.72
0
GFP+_+1
GFP-_+1
GFP+_-1
GFP-_-1
GFP+_+2
GFP-_+2
GFP+_-2
GFP-_-2
GFP+_+3
GFP-_+3
GFP+_-3
GFP-_-3
long-library_called_tADs
tgo_+1
long-library_enrichment
3
GFP+_+1.enr
GFP+_-1.enr
GFP+_+2.enr
GFP+_-2.enr
GFP+_+3.enr
GFP+_-3.enr

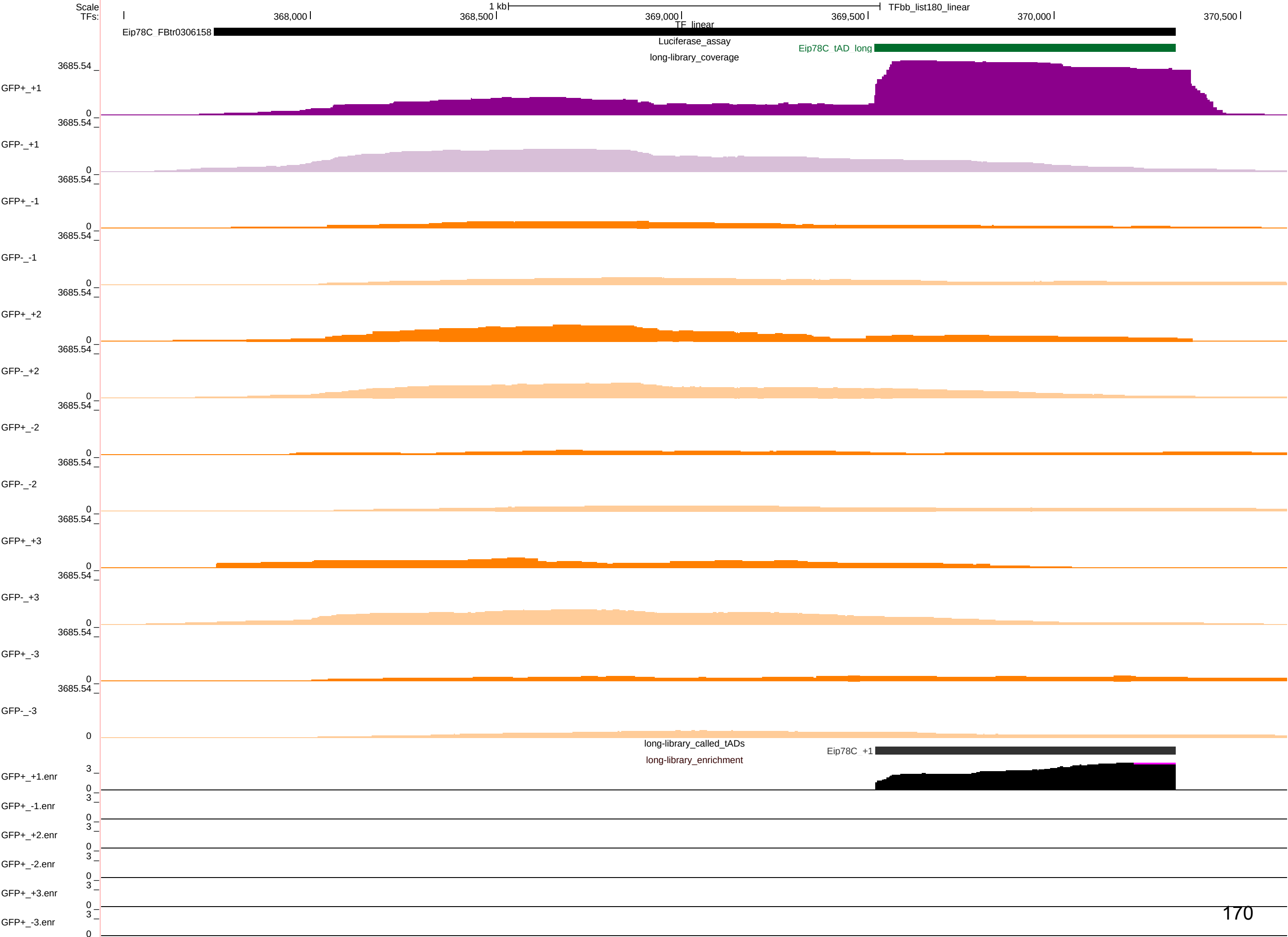
Scale
TFs:
1 kb
TFbb_list180_linear
368,000
368,500
369,000
369,500
370,000
370,500
TF_linear
Eip78C_FBtr0306158
Luciferase_assay
Eip78C_tAD_long
long-library_coverage
3685.54
0
GFP+_+1
GFP-_+1
GFP+_-1
GFP-_-1
GFP+_+2
GFP-_+2
GFP+_-2
GFP-_-2
GFP+_+3
GFP-_+3
GFP+_-3
GFP-_-3
long-library_called_tADs
Eip78C_+1
long-library_enrichment
3
GFP+_+1.enr
GFP+_-1.enr
GFP+_+2.enr
GFP+_-2.enr
GFP+_+3.enr
GFP+_-3.enr

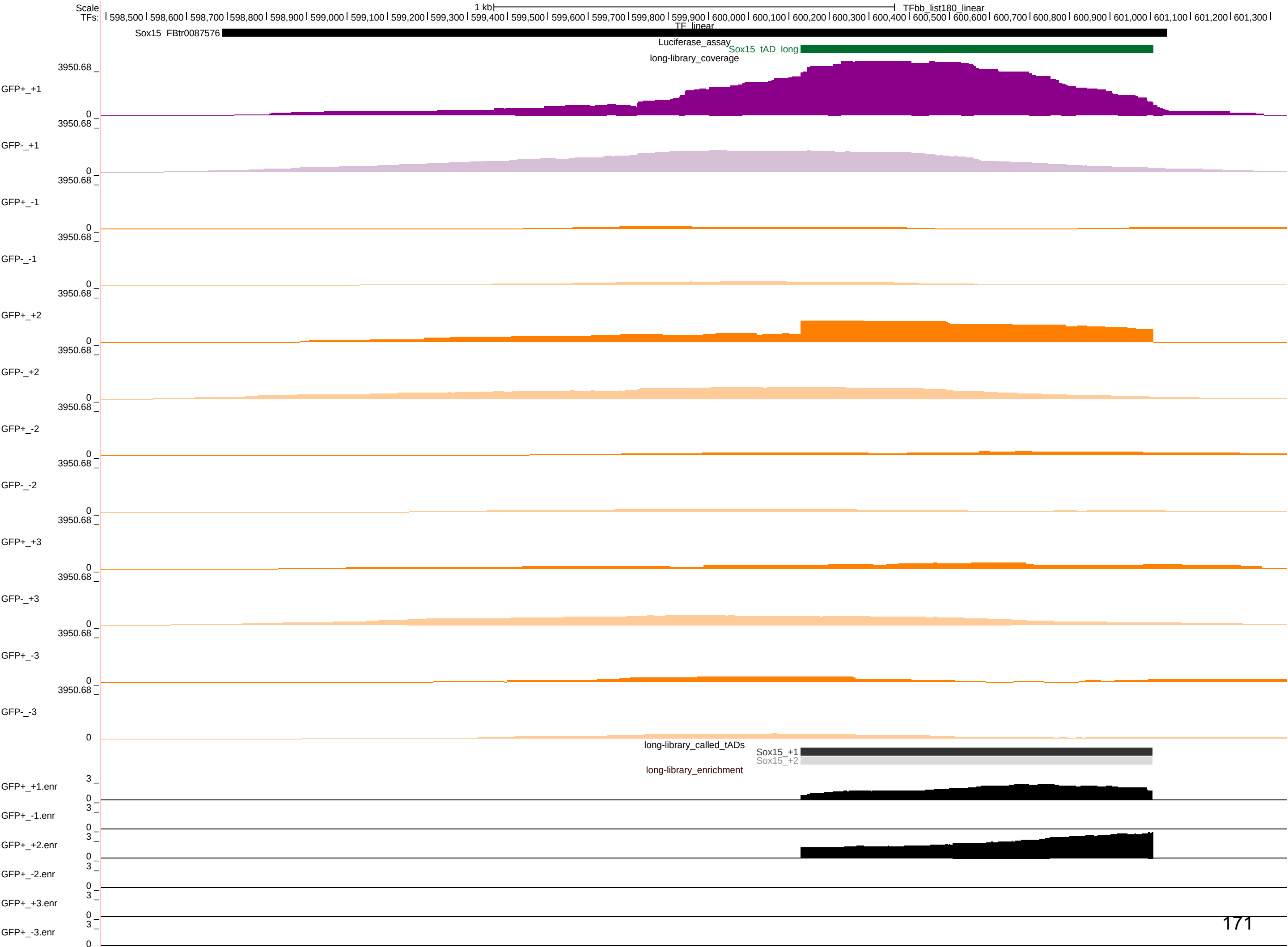
Scale
1 kb
TFbb_list180_linear
TFs:
598,500 598,600 598,700 598,800 598,900 599,000 599,100 599,200 599,300 599,400 599,500 599,600 599,700 599,800 599,900 600,000 600,100 600,200 600,300 600,400 600,500 600,600 600,700 600,800 600,900 601,000 601,100 601,200 601,300
TF_linear
Sox15_FBtr0087576
Luciferase_assay
Sox15_tAD_long
long-library_coverage
GFP+_+1
GFP-_+1
GFP+_-1
GFP-_-1
GFP+_+2
GFP-_+2
GFP+_-2
GFP-_-2
GFP+_+3
GFP-_+3
GFP+_-3
GFP-_-3
3950.68
0
long-library_called_tADs
Sox15_+1
Sox15_+2
long-library_enrichment
GFP+_+1.enr
GFP+_-1.enr
GFP+_+2.enr
GFP+_-2.enr
GFP+_+3.enr
GFP+_-3.enr
3
0

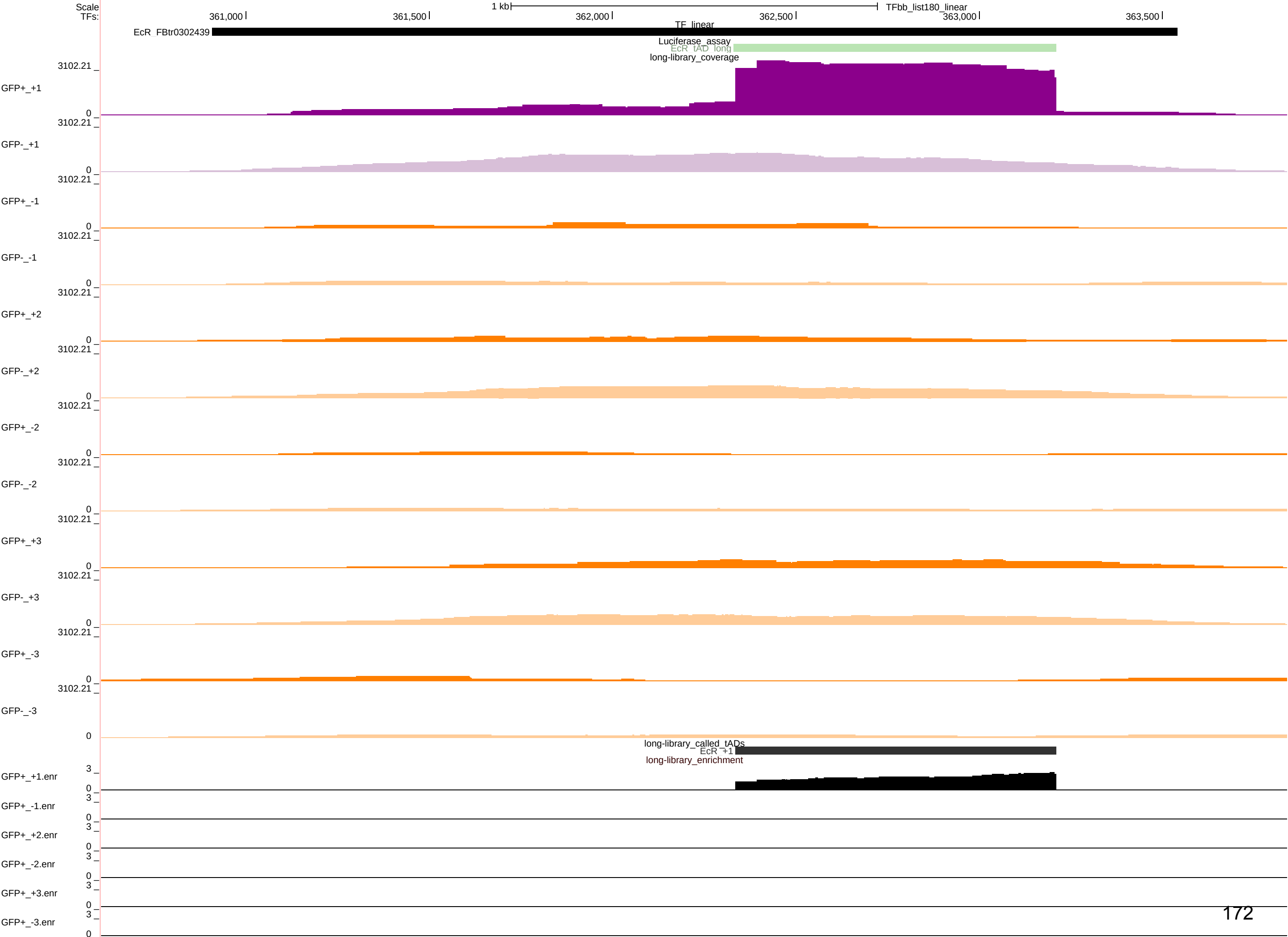
Scale
TFs:
1 kb
TFbb_list180_linear
361,000
361,500
362,000
362,500
363,000
363,500
TF_linear
EcR_FBtr0302439
Luciferase_assay
EcR_tAD_long
long-library_coverage
3102.21
GFP+_+1
0
GFP-_+1
GFP+_-1
GFP-_-1
GFP+_+2
GFP-_+2
GFP+_-2
GFP-_-2
GFP+_+3
GFP-_+3
GFP+_-3
GFP-_-3
long-library_called_tADs
EcR_+1
long-library_enrichment
3
GFP+_+1.enr
GFP+_-1.enr
GFP+_+2.enr
GFP+_-2.enr
GFP+_+3.enr
GFP+_-3.enr

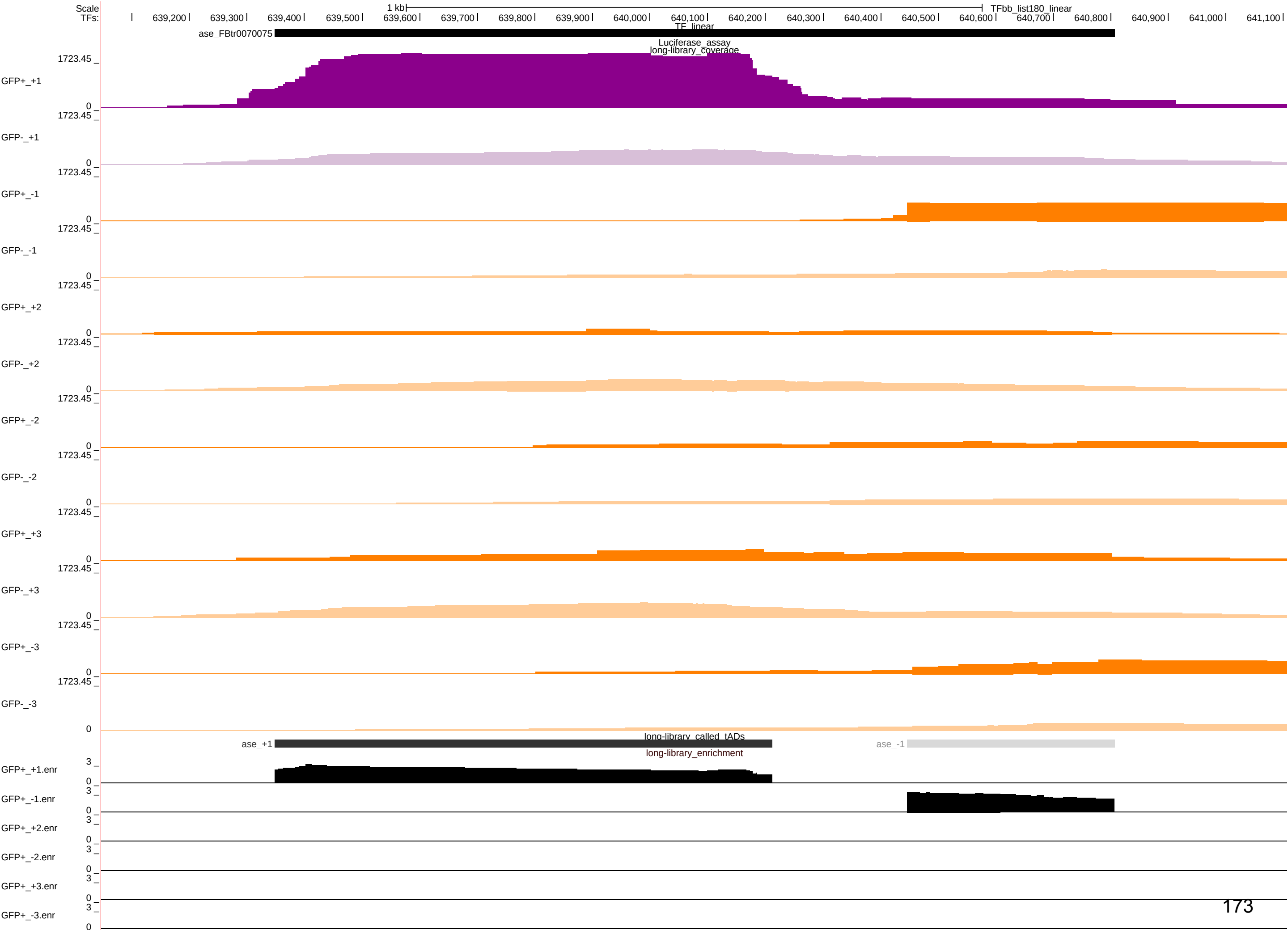

Scale
1 kb
TFbb_list180_linear
TFs:
639,200
639,300
639,400
639,500
639,600
639,700
639,800
639,900
640,000
640,100
640,200
640,300
640,400
640,500
640,600
640,700
640,800
640,900
641,000
641,100
TF_linear
ase_FBtr0070075
Luciferase_assay
long-library_coverage
1723.45
0
GFP+_+1
GFP-_+1
GFP+_-1
GFP-_-1
GFP+_+2
GFP-_+2
GFP+_-2
GFP-_-2
GFP+_+3
GFP-_+3
GFP+_-3
GFP-_-3
long-library_called_tADs
ase_+1
ase_-1
long-library_enrichment
3
GFP+_+1.enr
GFP+_-1.enr
GFP+_+2.enr
GFP+_-2.enr
GFP+_+3.enr
GFP+_-3.enr

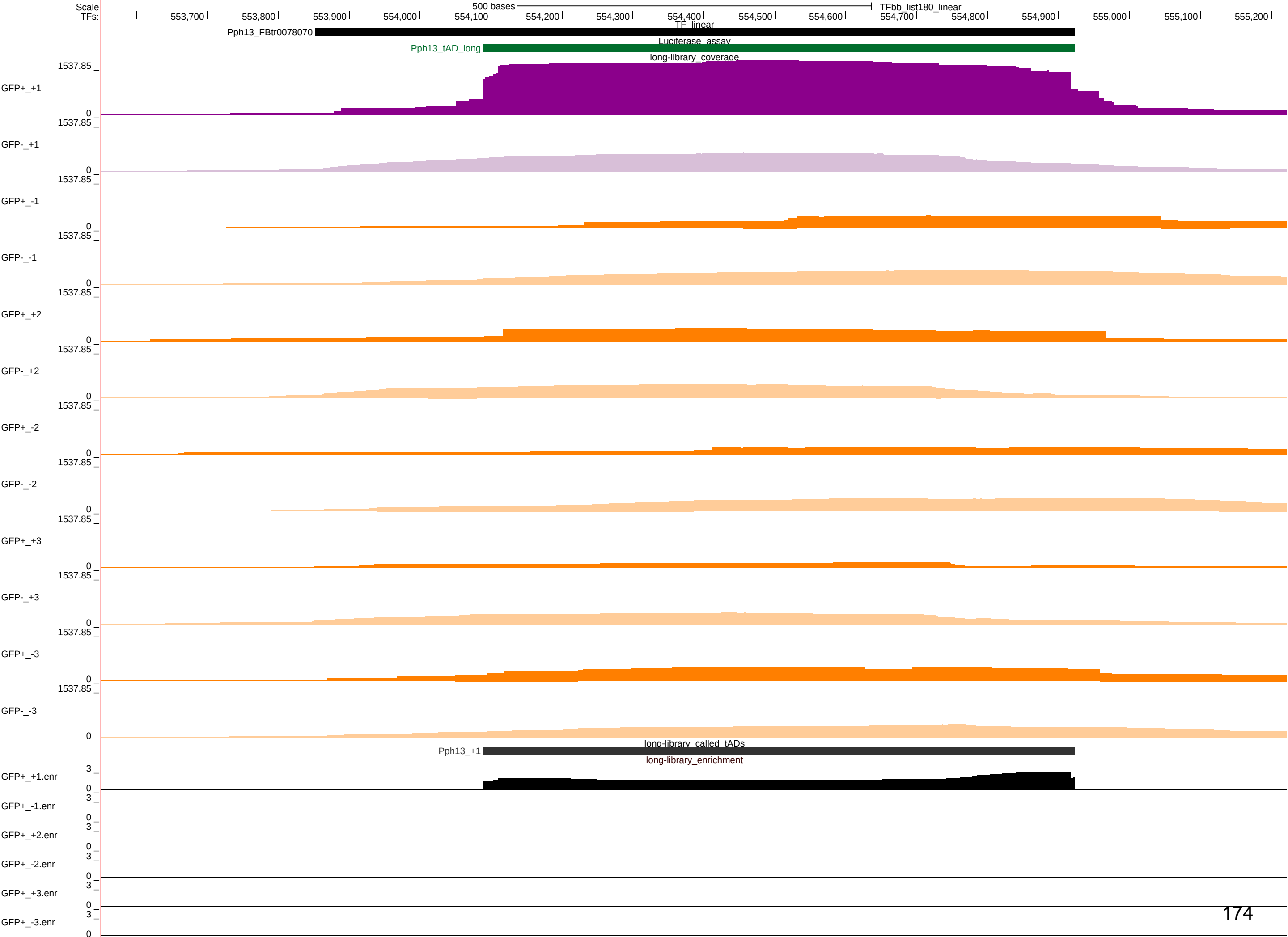
Scale
TFs:
500 bases
TFbb_list180_linear
553,700
553,800
553,900
554,000
554,100
554,200
554,300
554,400
554,500
554,600
554,700
554,800
554,900
555,000
555,100
555,200
TF_linear
Pph13_FBtr0078070
Luciferase_assay
Pph13_tAD_long
long-library_coverage
1537.85 _
GFP+_+1
0 _
GFP-_+1
GFP+_-1
GFP-_-1
GFP+_+2
GFP-_+2
GFP+_-2
GFP-_-2
GFP+_+3
GFP-_+3
GFP+_-3
GFP-_-3
long-library_called_tADs
Pph13_+1
long-library_enrichment
3 _
GFP+_+1.enr
GFP+_-1.enr
GFP+_+2.enr
GFP+_-2.enr
GFP+_+3.enr
GFP+_-3.enr

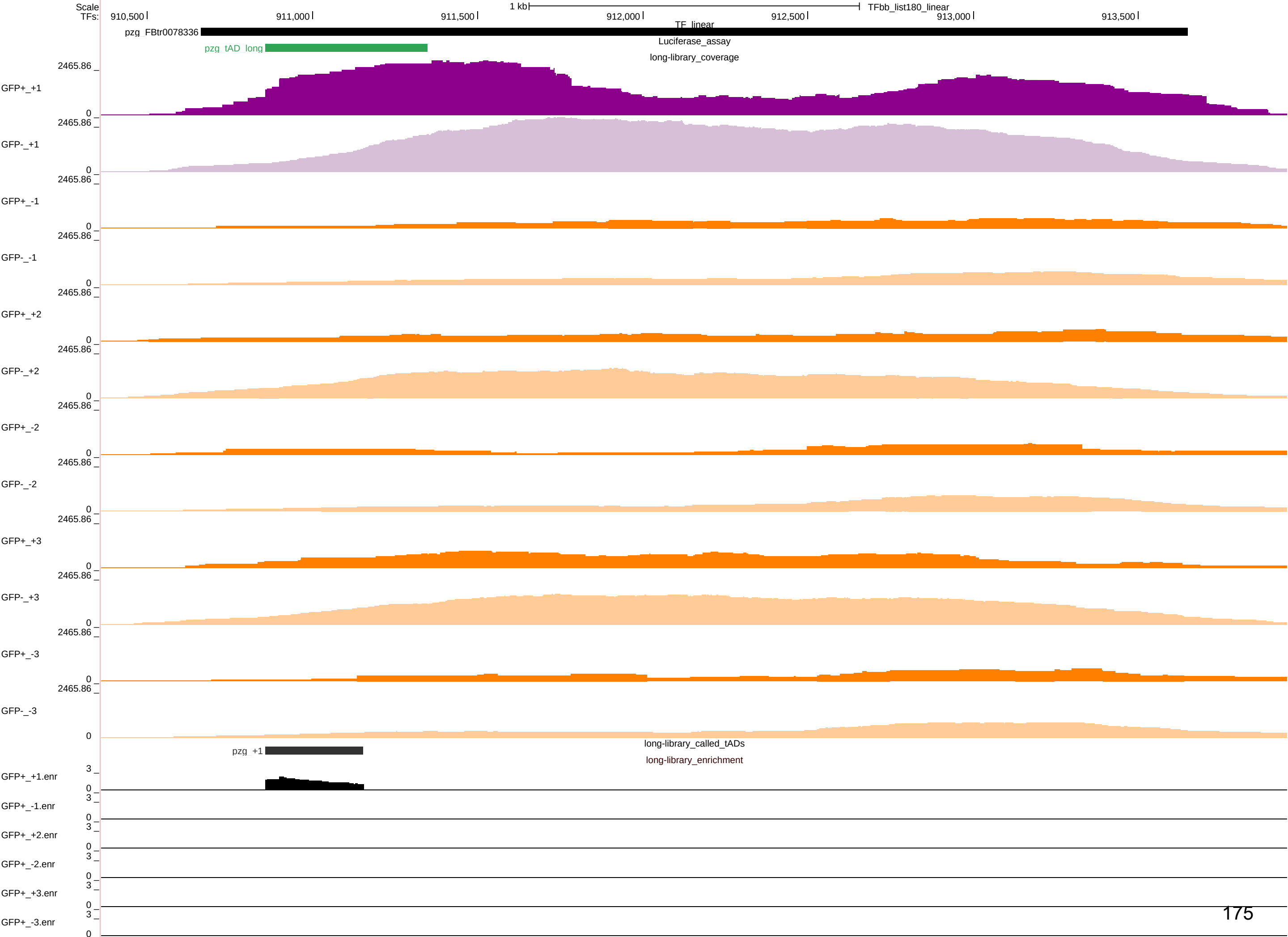
Scale
TFs:
910,500
911,000
911,500
912,000
912,500
913,000
913,500
1 kb
TFbb_list180_linear
TF_linear
pzg_FBtr0078336
Luciferase_assay
pzg_tAD_long
long-library_coverage
GFP+_+1
GFP-_+1
GFP+_-1
GFP-_-1
GFP+_+2
GFP-_+2
GFP+_-2
GFP-_-2
GFP+_+3
GFP-_+3
GFP+_-3
GFP-_-3
2465.86
0
long-library_called_tADs
pzg_+1
long-library_enrichment
GFP+_+1.enr
GFP+_-1.enr
GFP+_+2.enr
GFP+_-2.enr
GFP+_+3.enr
GFP+_-3.enr
3
0

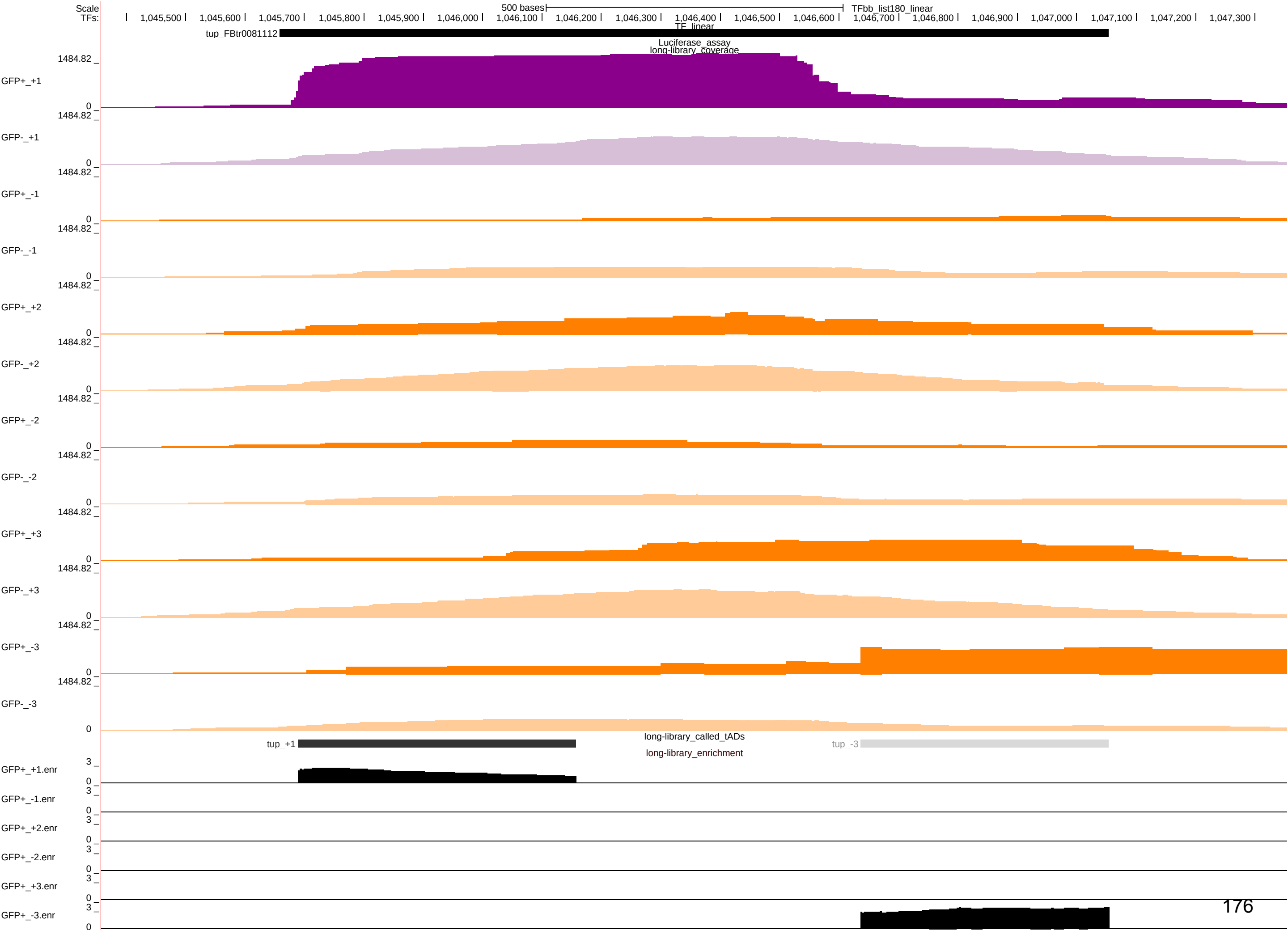
Scale
TFs:
500 bases
TFbb_list180_linear
1,045,500
1,045,600
1,045,700
1,045,800
1,045,900
1,046,000
1,046,100
1,046,200
1,046,300
1,046,400
1,046,500
1,046,600
1,046,700
1,046,800
1,046,900
1,047,000
1,047,100
1,047,200
1,047,300
TF_linear
tup_FBtr0081112
Luciferase_assay
long-library_coverage
GFP+_+1
GFP-_+1
GFP+_-1
GFP-_-1
GFP+_+2
GFP-_+2
GFP+_-2
GFP-_-2
GFP+_+3
GFP-_+3
GFP+_-3
GFP-_-3
1484.82
0
long-library_called_tADs
tup_+1
tup_-3
long-library_enrichment
GFP+_+1.enr
GFP+_-1.enr
GFP+_+2.enr
GFP+_-2.enr
GFP+_+3.enr
GFP+_-3.enr
3
0

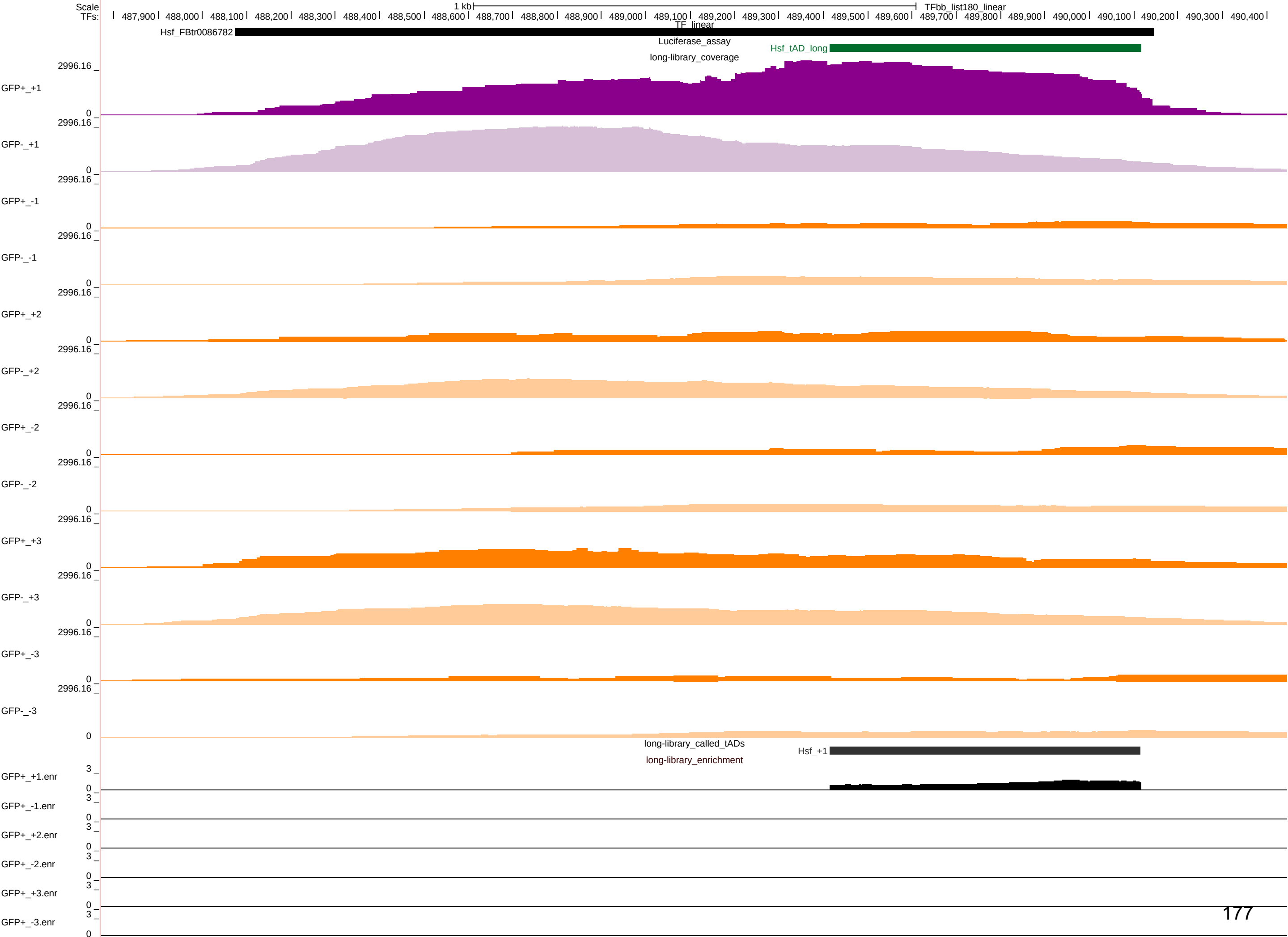
Scale
1 kb
TFbb_list180_linear
TFs:
487,900
488,000
488,100
488,200
488,300
488,400
488,500
488,600
488,700
488,800
488,900
489,000
489,100
489,200
489,300
489,400
489,500
489,600
489,700
489,800
489,900
490,000
490,100
490,200
490,300
490,400
TF_linear
Hsf_FBtr0086782
Luciferase_assay
Hsf_tAD_long
long-library_coverage
GFP+_+1
GFP-_+1
GFP+_-1
GFP-_-1
GFP+_+2
GFP-_+2
GFP+_-2
GFP-_-2
GFP+_+3
GFP-_+3
GFP+_-3
GFP-_-3
2996.16
0
long-library_called_tADs
Hsf_+1
long-library_enrichment
GFP+_+1.enr
GFP+_-1.enr
GFP+_+2.enr
GFP+_-2.enr
GFP+_+3.enr
GFP+_-3.enr
3
0

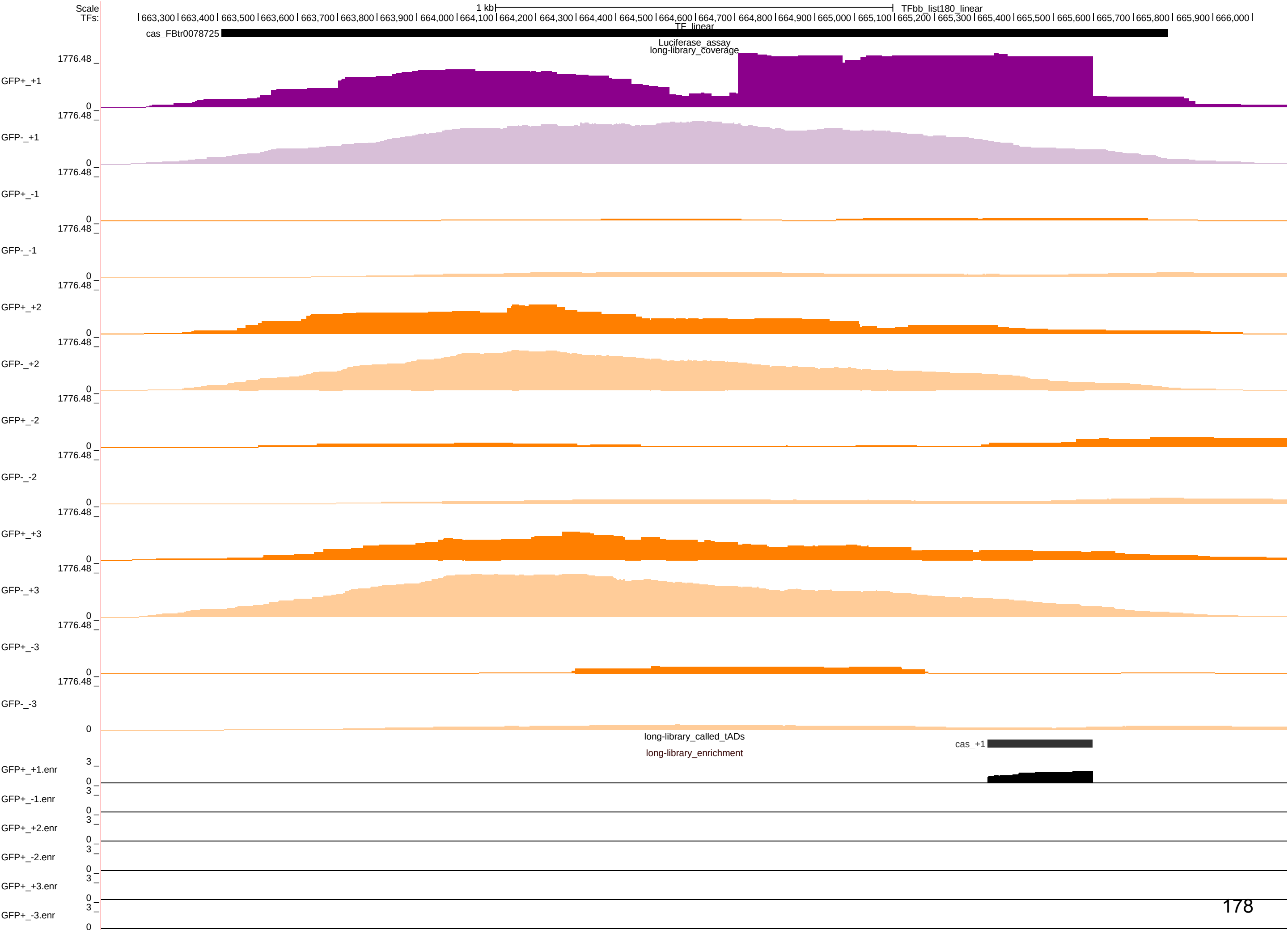
Scale
TFs:
1 kb
TFbb_list180_linear
663,300 663,400 663,500 663,600 663,700 663,800 663,900 664,000 664,100 664,200 664,300 664,400 664,500 664,600 664,700 664,800 664,900 665,000 665,100 665,200 665,300 665,400 665,500 665,600 665,700 665,800 665,900 666,000
TF_linear
cas_FBtr0078725
Luciferase_assay
long-library_coverage
1776.48
0
GFP+_+1
GFP-_+1
GFP+_-1
GFP-_-1
GFP+_+2
GFP-_+2
GFP+_-2
GFP-_-2
GFP+_+3
GFP-_+3
GFP+_-3
GFP-_-3
long-library_called_tADs
cas_+1
long-library_enrichment
3
GFP+_+1.enr
GFP+_-1.enr
GFP+_+2.enr
GFP+_-2.enr
GFP+_+3.enr
GFP+_-3.enr

Appendix Figure S4

13 long-fragment library non-native frame tADs

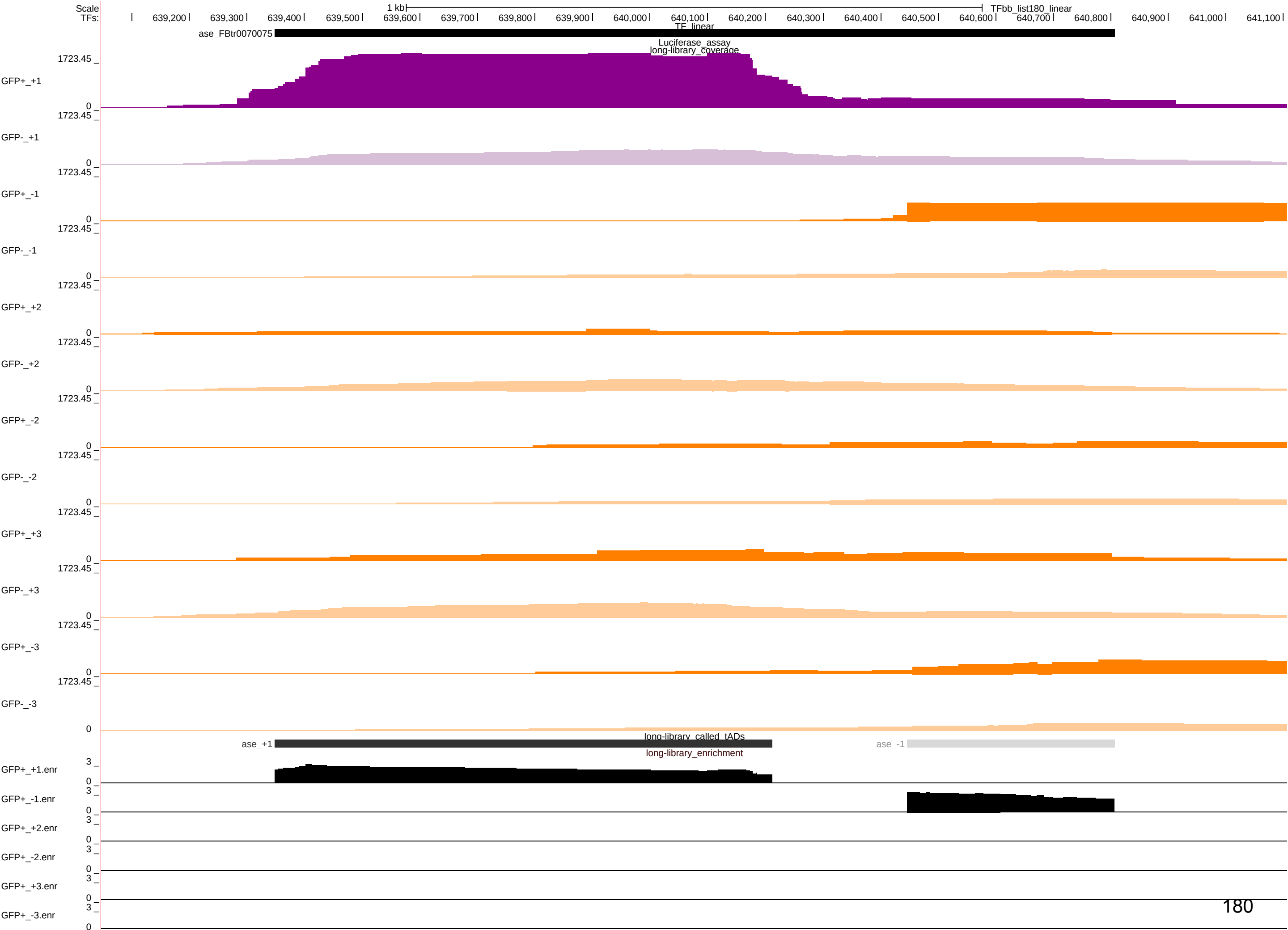

Scale
1 kb
TFbb_list180_linear
TFs:
639,200
639,300
639,400
639,500
639,600
639,700
639,800
639,900
640,000
640,100
640,200
640,300
640,400
640,500
640,600
640,700
640,800
640,900
641,000
641,100
TF_linear
ase_FBtr0070075
Luciferase_assay
long-library_coverage
1723.45
0
GFP+_+1
GFP-_+1
GFP+_-1
GFP-_-1
GFP+_+2
GFP-_+2
GFP+_-2
GFP-_-2
GFP+_+3
GFP-_+3
GFP+_-3
GFP-_-3
long-library_called_tADs
ase_+1
ase_-1
long-library_enrichment
3
GFP+_+1.enr
GFP+_-1.enr
GFP+_+2.enr
GFP+_-2.enr
GFP+_+3.enr
GFP+_-3.enr

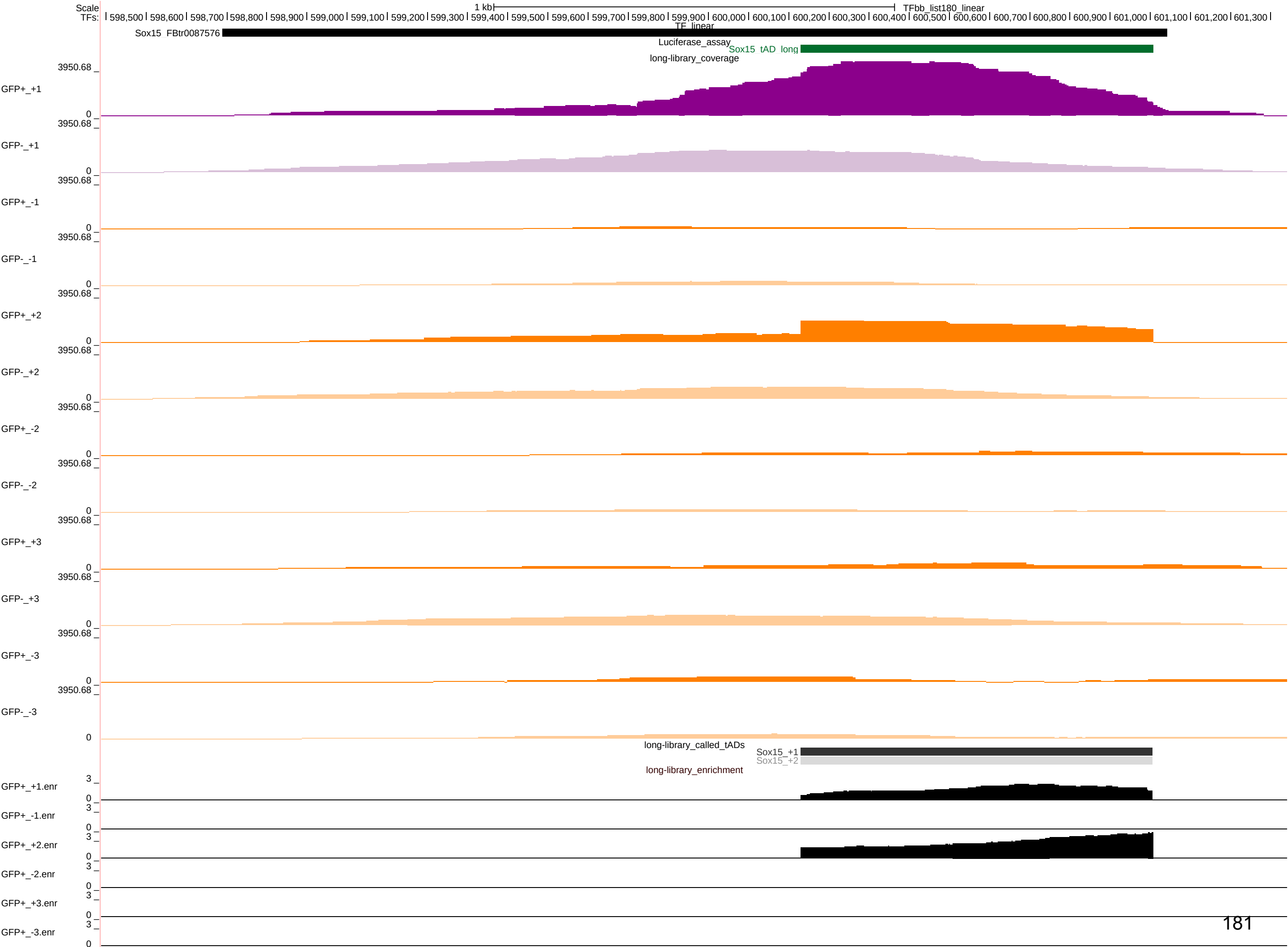
Scale
1 kb
TFbb_list180_linear
TFs:
598,500
598,600
598,700
598,800
598,900
599,000
599,100
599,200
599,300
599,400
599,500
599,600
599,700
599,800
599,900
600,000
600,100
600,200
600,300
600,400
600,500
600,600
600,700
600,800
600,900
601,000
601,100
601,200
601,300
TF_linear
Sox15_FBtr0087576
Luciferase_assay
Sox15_tAD_long
long-library_coverage
3950.68
0
GFP+_+1
GFP-_+1
GFP+_-1
GFP-_-1
GFP+_+2
GFP-_+2
GFP+_-2
GFP-_-2
GFP+_+3
GFP-_+3
GFP+_-3
GFP-_-3
long-library_called_tADs
Sox15_+1
Sox15_+2
long-library_enrichment
3
GFP+_+1.enr
GFP+_-1.enr
GFP+_+2.enr
GFP+_-2.enr
GFP+_+3.enr
GFP+_-3.enr

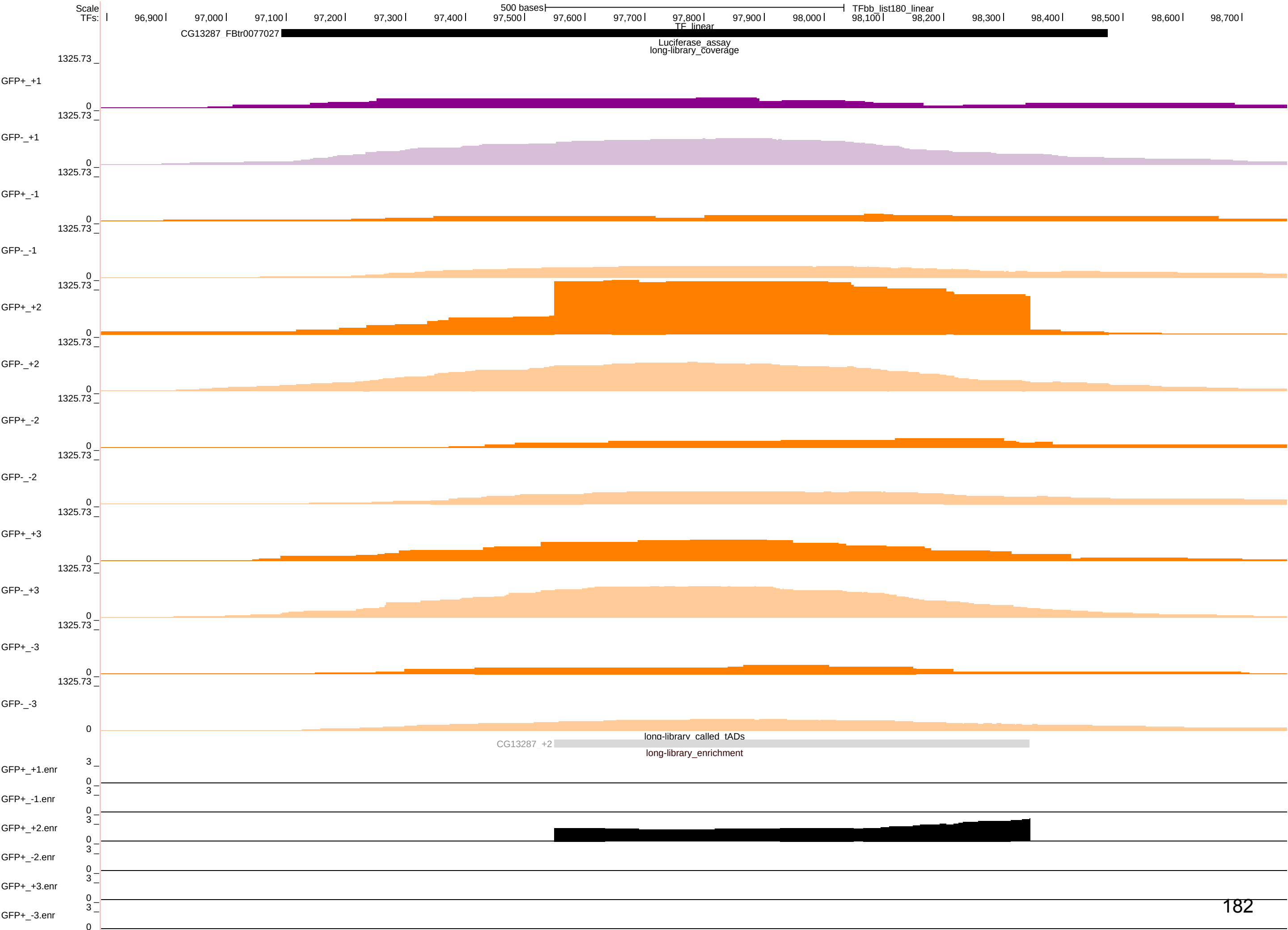
Scale
TFs:
500 bases
TFbb_list180_linear
96,900
97,000
97,100
97,200
97,300
97,400
97,500
97,600
97,700
97,800
97,900
98,000
98,100
98,200
98,300
98,400
98,500
98,600
98,700
TF_linear
CG13287 FBtr0077027
Luciferase_assay
long-library_coverage
1325.73
0
GFP+_+1
GFP-_+1
GFP+_-1
GFP-_-1
GFP+_+2
GFP-_+2
GFP+_-2
GFP-_-2
GFP+_+3
GFP-_+3
GFP+_-3
GFP-_-3
long-library_called_tADs
CG13287_+2
long-library_enrichment
3
GFP+_+1.enr
GFP+_-1.enr
GFP+_+2.enr
GFP+_-2.enr
GFP+_+3.enr
GFP+_-3.enr

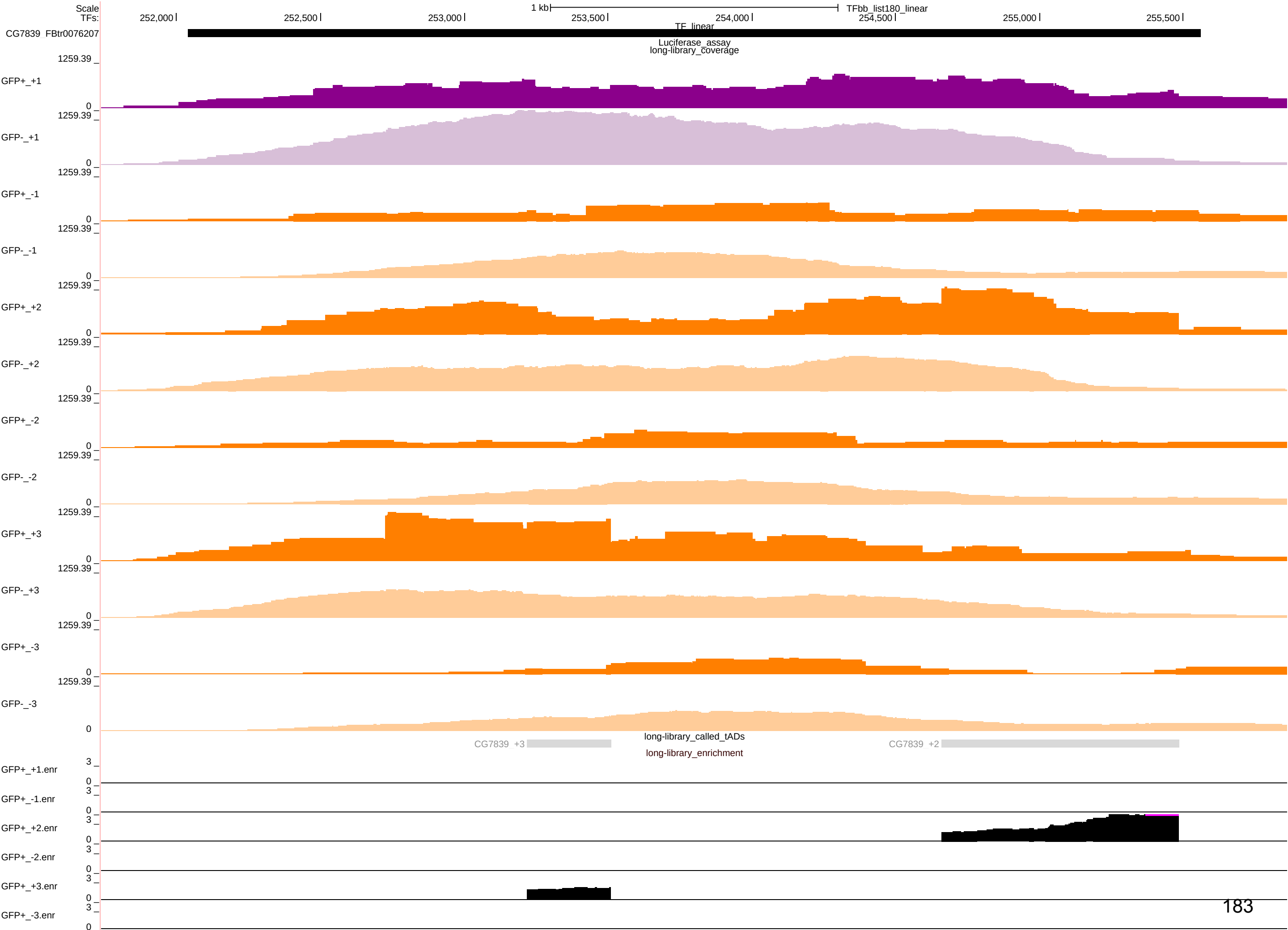
Scale
1 kb
TFs:
TFbb_list180_linear
252,000
252,500
253,000
253,500
254,000
254,500
255,000
255,500
TF_linear
CG7839_FBtr0076207
Luciferase_assay
long-library_coverage
1259.39
0
GFP+_+1
GFP-_+1
GFP+_-1
GFP-_-1
GFP+_+2
GFP-_+2
GFP+_-2
GFP-_-2
GFP+_+3
GFP-_+3
GFP+_-3
GFP-_-3
long-library_called_tADs
CG7839_+3
CG7839_+2
long-library_enrichment
3
GFP+_+1.enr
GFP+_-1.enr
GFP+_+2.enr
GFP+_-2.enr
GFP+_+3.enr
GFP+_-3.enr

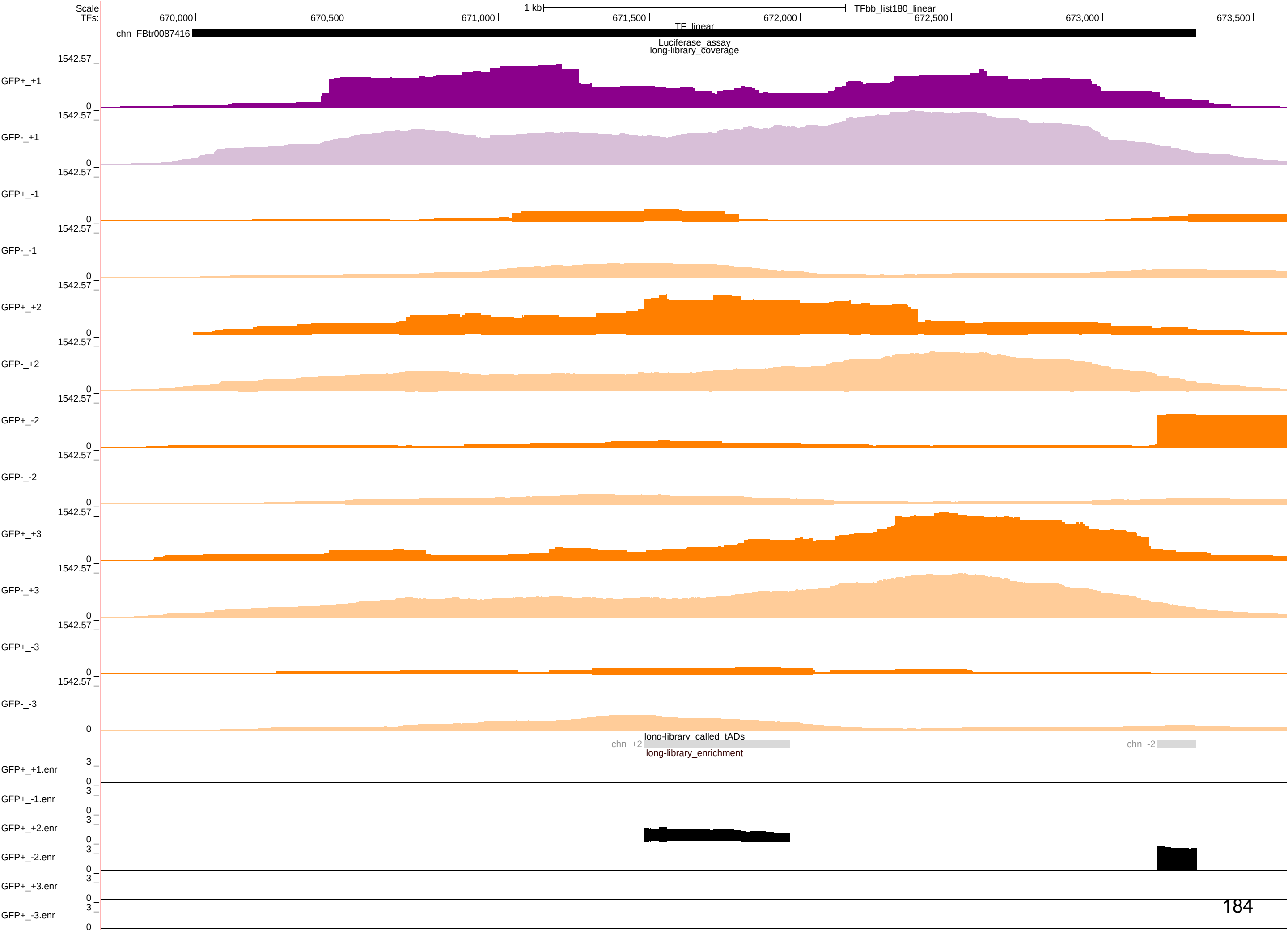

Scale
TFs:
1 kb
TFbb_list180_linear
670,000
670,500
671,000
671,500
672,000
672,500
673,000
673,500
TF_linear
chn FBtr0087416
Luciferase_assay
long-library_coverage
GFP+_+1
GFP-_+1
GFP+_-1
GFP-_-1
GFP+_+2
GFP-_+2
GFP+_-2
GFP-_-2
GFP+_+3
GFP-_+3
GFP+_-3
GFP-_-3
1542.57
0
long-library_called_tADs
chn_+2
chn_-2
long-library_enrichment
GFP+_+1.enr
GFP+_-1.enr
GFP+_+2.enr
GFP+_-2.enr
GFP+_+3.enr
GFP+_-3.enr
3
0

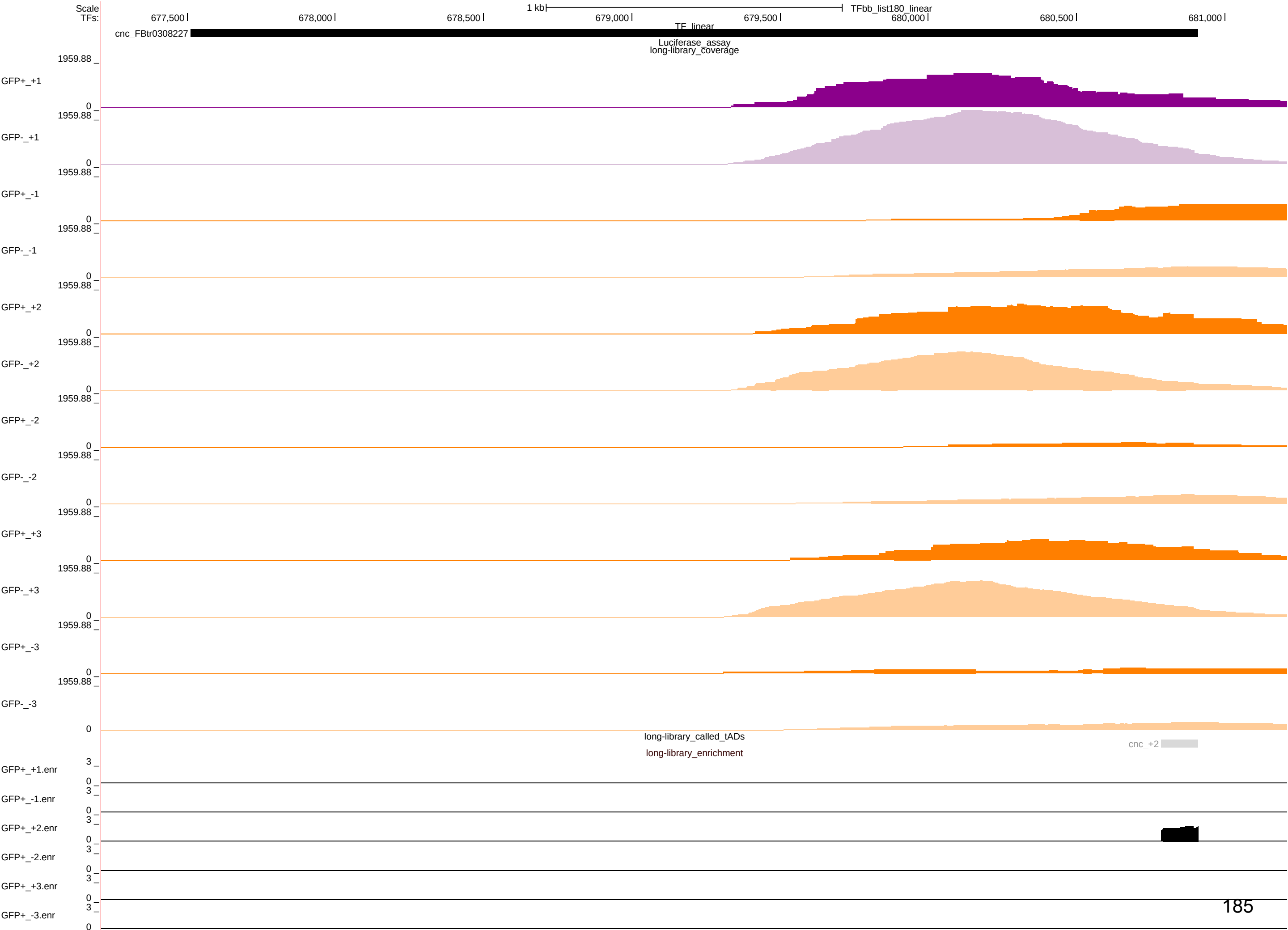

Scale
TFs:
1 kb
TFbb_list180_linear
677,500
678,000
678,500
679,000
679,500
680,000
680,500
681,000
TF_linear
cnc FBtr0308227
Luciferase_assay
long-library_coverage
1959.88
GFP+_+1
0
GFP-_+1
GFP+_-1
GFP-_-1
GFP+_+2
GFP-_+2
GFP+_-2
GFP-_-2
GFP+_+3
GFP-_+3
GFP+_-3
GFP-_-3
long-library_called_tADs
cnc +2
long-library_enrichment
3
GFP+_+1.enr
GFP+_-1.enr
GFP+_+2.enr
GFP+_-2.enr
GFP+_+3.enr
GFP+_-3.enr

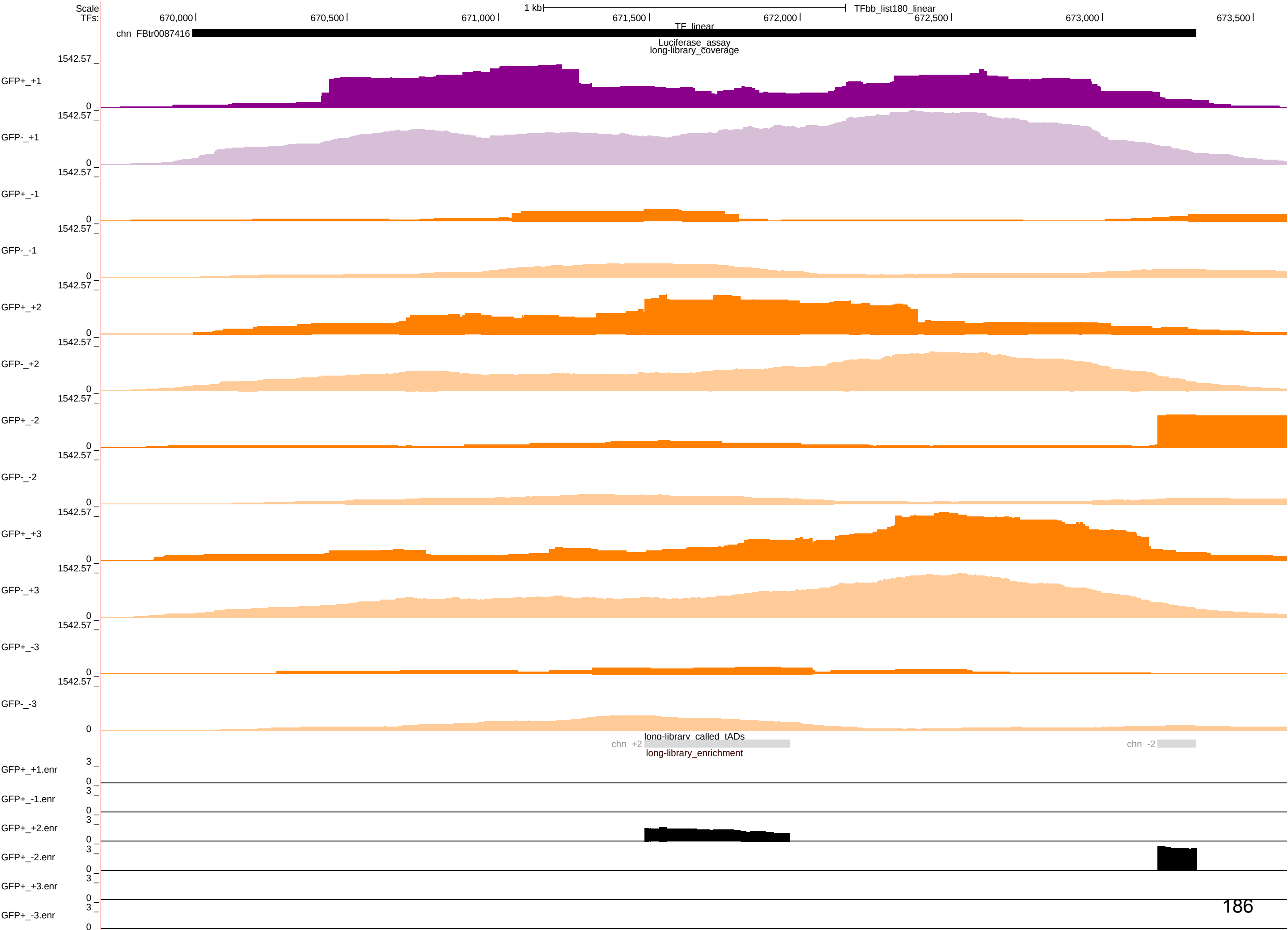
Scale
TFs:
1 kb
TFbb_list180_linear
670,000
670,500
671,000
671,500
672,000
672,500
673,000
673,500
TF_linear
chn_FBtr0087416
Luciferase_assay
long-library_coverage
GFP+_+1
GFP-_+1
GFP+_-1
GFP-_-1
GFP+_+2
GFP-_+2
GFP+_-2
GFP-_-2
GFP+_+3
GFP-_+3
GFP+_-3
GFP-_-3
1542.57
0
long-library_called_tADs
chn_+2
chn_-2
long-library_enrichment
GFP+_+1.enr
GFP+_-1.enr
GFP+_+2.enr
GFP+_-2.enr
GFP+_+3.enr
GFP+_-3.enr
3
0

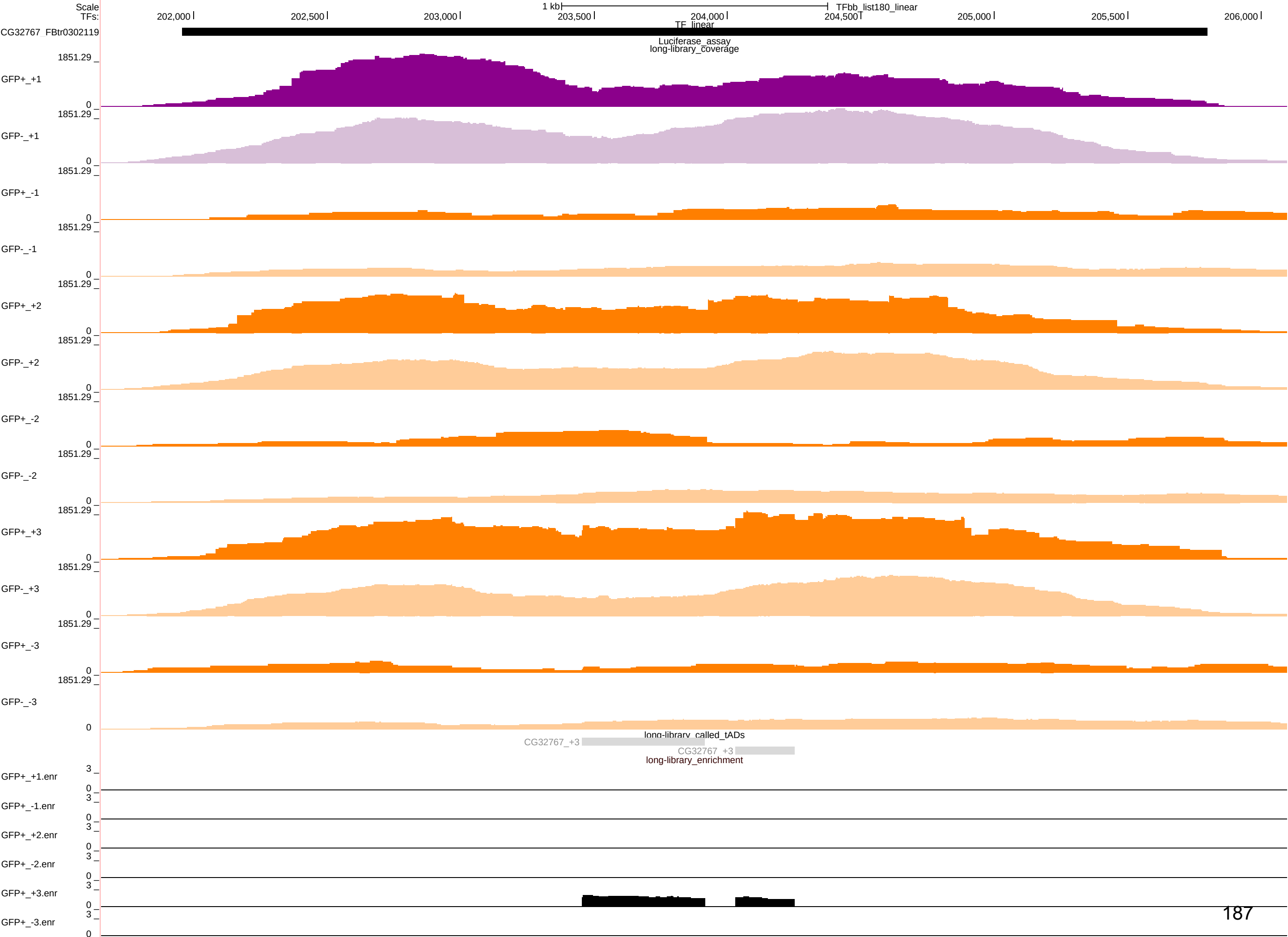
Scale
TFs:
1 kb
TFbb_list180_linear
202,000
202,500
203,000
203,500
204,000
204,500
205,000
205,500
206,000
TF_linear
CG32767_FBtr0302119
Luciferase_assay
long-library_coverage
1851.29 _
GFP+_+1
0 _
1851.29 _
GFP-_+1
0 _
1851.29 _
GFP+_-1
0 _
1851.29 _
GFP-_-1
0 _
1851.29 _
GFP+_+2
0 _
1851.29 _
GFP-_+2
0 _
1851.29 _
GFP+_-2
0 _
1851.29 _
GFP-_-2
0 _
1851.29 _
GFP+_+3
0 _
1851.29 _
GFP-_+3
0 _
1851.29 _
GFP+_-3
0 _
1851.29 _
GFP-_-3
0
long-library_called_tADs
CG32767_+3
CG32767_+3
long-library_enrichment
GFP+_+1.enr
3 _
0 _
GFP+_-1.enr
3 _
0 _
GFP+_+2.enr
3 _
0 _
GFP+_-2.enr
3 _
0 _
GFP+_+3.enr
3 _
0 _
GFP+_-3.enr
3 _
0

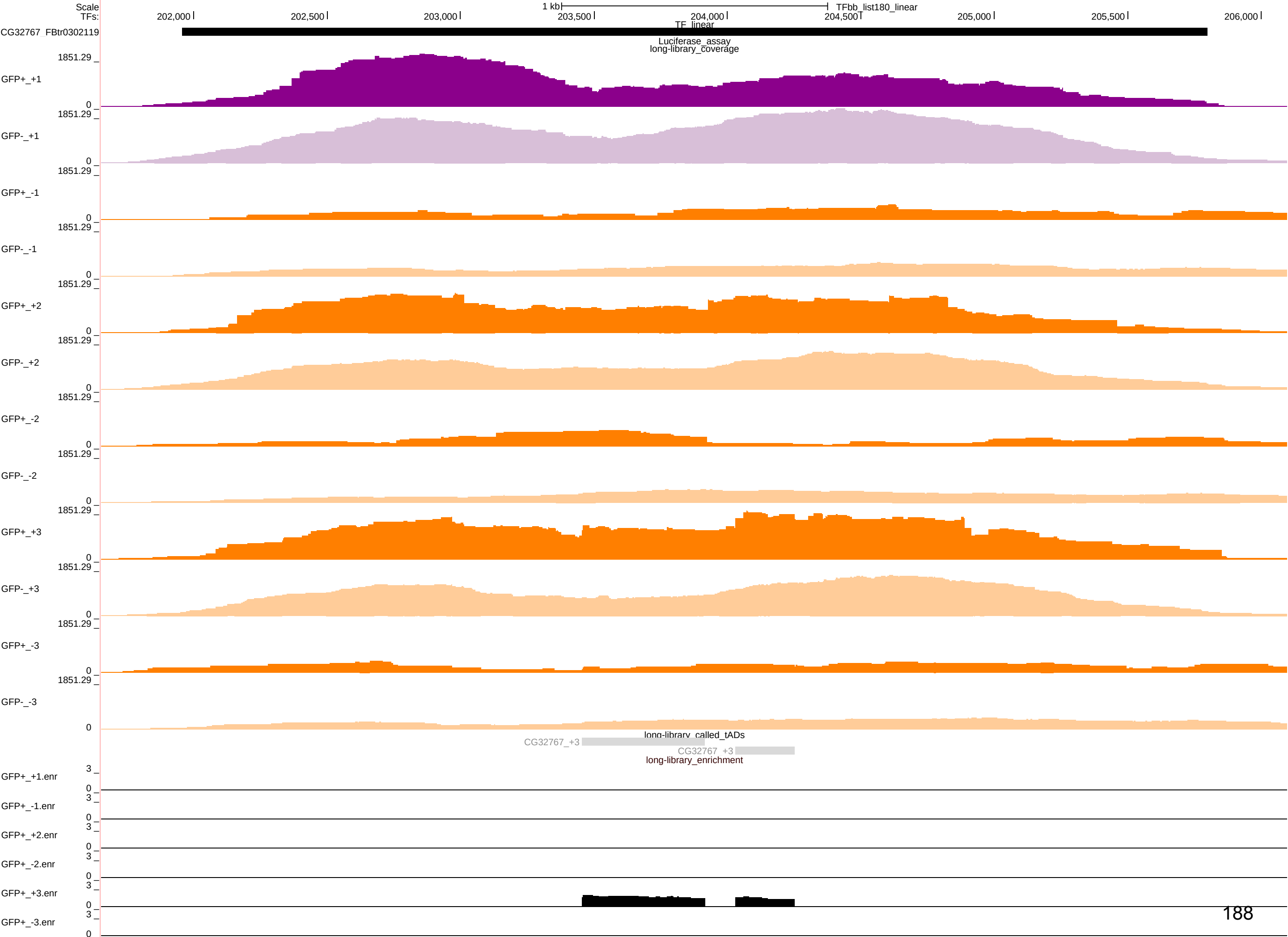
Scale
TFs:
1 kb
TFbb_list180_linear
202,000
202,500
203,000
203,500
204,000
204,500
205,000
205,500
206,000
TF_linear
CG32767_FBtr0302119
Luciferase_assay
long-library_coverage
GFP+_+1
GFP-_+1
GFP+_-1
GFP-_-1
GFP+_+2
GFP-_+2
GFP+_-2
GFP-_-2
GFP+_+3
GFP-_+3
GFP+_-3
GFP-_-3
1851.29
0
long-library_called_tADs
CG32767_+3
CG32767_+3
long-library_enrichment
GFP+_+1.enr
GFP+_-1.enr
GFP+_+2.enr
GFP+_-2.enr
GFP+_+3.enr
GFP+_-3.enr
3
0

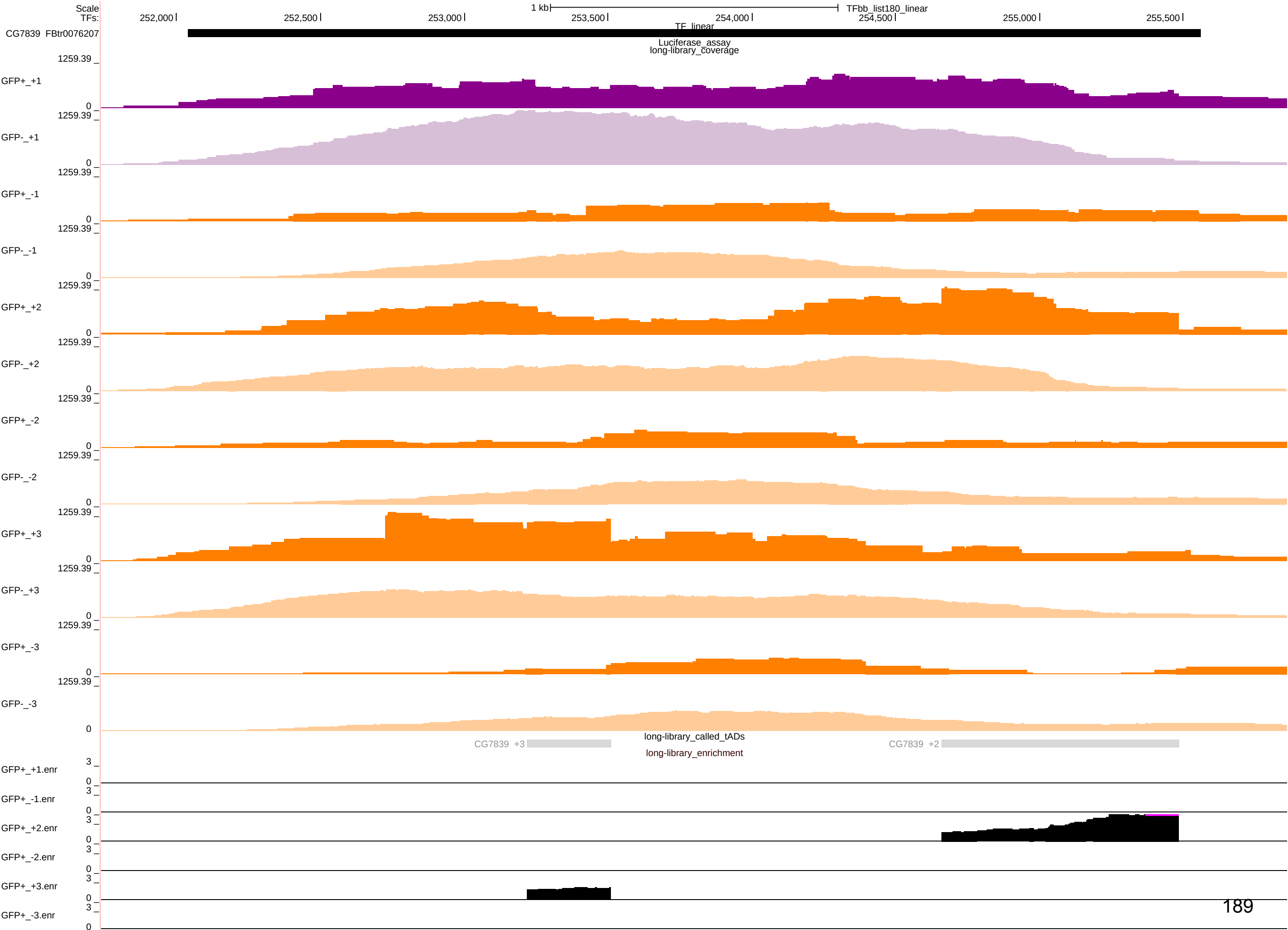
Scale
1 kb
TFbb_list180_linear
TFs:
252,000
252,500
253,000
253,500
254,000
254,500
255,000
255,500
TF_linear
CG7839_FBtr0076207
Luciferase_assay
long-library_coverage
GFP+_+1
GFP-_+1
GFP+_-1
GFP-_-1
GFP+_+2
GFP-_+2
GFP+_-2
GFP-_-2
GFP+_+3
GFP-_+3
GFP+_-3
GFP-_-3
1259.39
0
long-library_called_tADs
CG7839_+3
CG7839_+2
long-library_enrichment
GFP+_+1.enr
GFP+_-1.enr
GFP+_+2.enr
GFP+_-2.enr
GFP+_+3.enr
GFP+_-3.enr
3
0

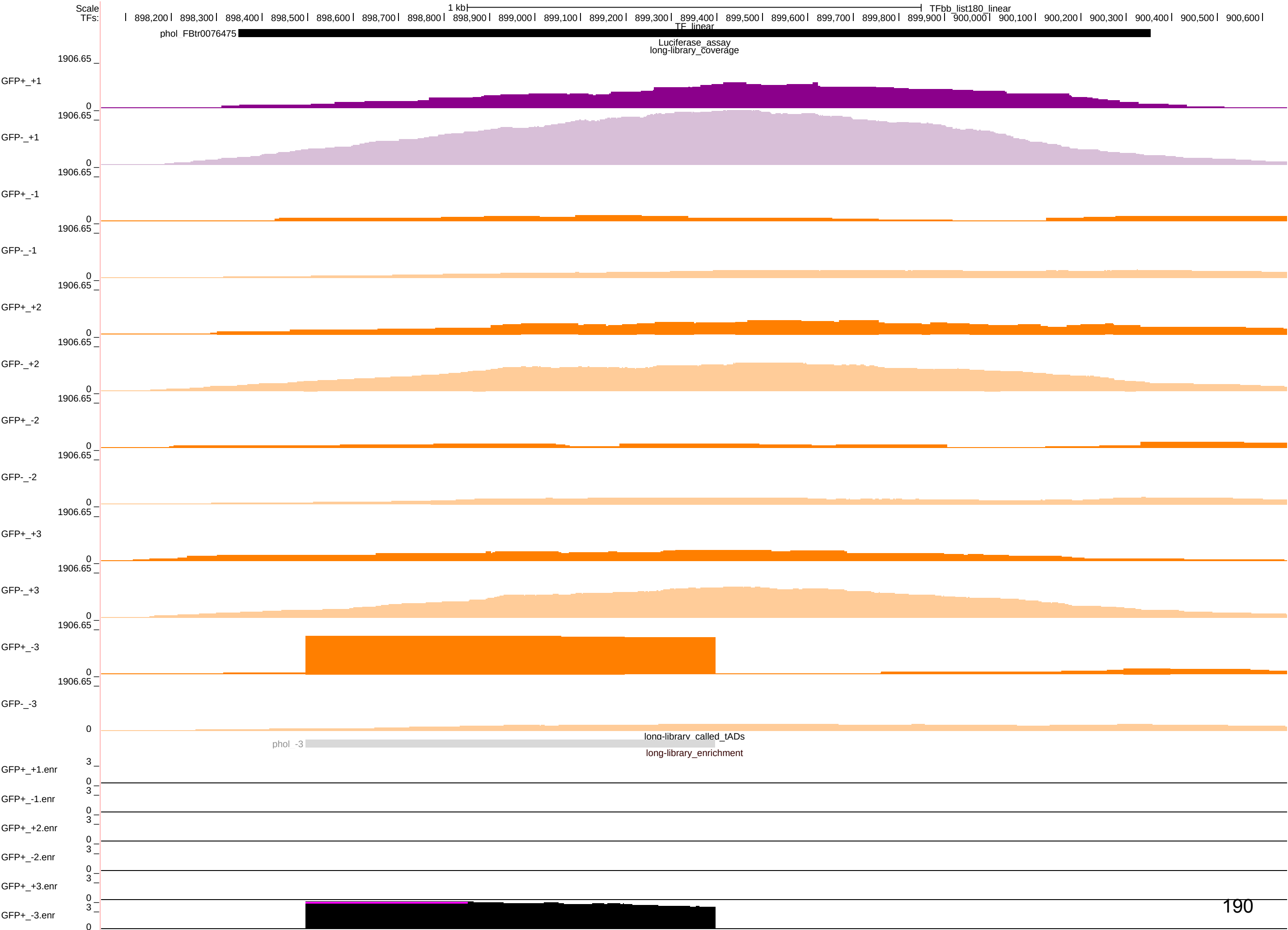
Scale
1 kb
TFbb_list180_linear
TFs:
898,200 898,300 898,400 898,500 898,600 898,700 898,800 898,900 899,000 899,100 899,200 899,300 899,400 899,500 899,600 899,700 899,800 899,900 900,000 900,100 900,200 900,300 900,400 900,500 900,600
TF_linear
phol_FBtr0076475
Luciferase_assay
long-library_coverage
1906.65
0
GFP+_+1
GFP-_+1
GFP+_-1
GFP-_-1
GFP+_+2
GFP-_+2
GFP+_-2
GFP-_-2
GFP+_+3
GFP-_+3
GFP+_-3
GFP-_-3
long-library_called_tADs
phol_-3
long-library_enrichment
3
GFP+_+1.enr
GFP+_-1.enr
GFP+_+2.enr
GFP+_-2.enr
GFP+_+3.enr
GFP+_-3.enr

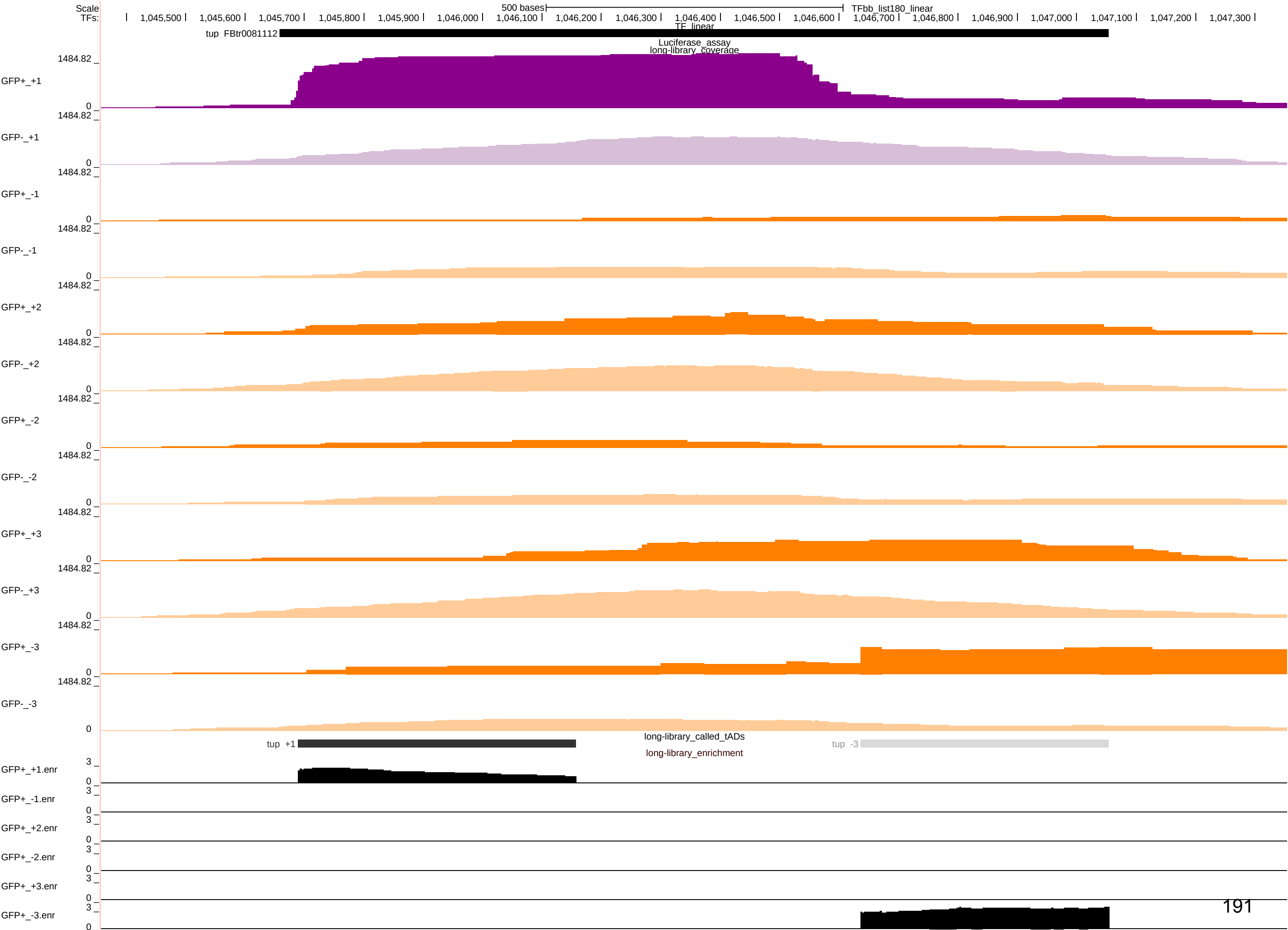
Scale
500 bases
TFs:
TFbb_list180_linear
1,045,500
1,045,600
1,045,700
1,045,800
1,045,900
1,046,000
1,046,100
1,046,200
1,046,300
1,046,400
1,046,500
1,046,600
1,046,700
1,046,800
1,046,900
1,047,000
1,047,100
1,047,200
1,047,300
TF_linear
tup_FBtr0081112
Luciferase_assay
long-library_coverage
1484.82 _
0 _
GFP+_+1
GFP-_+1
GFP+_-1
GFP-_-1
GFP+_+2
GFP-_+2
GFP+_-2
GFP-_-2
GFP+_+3
GFP-_+3
GFP+_-3
GFP-_-3
long-library_called_tADs
tup_+1
tup_-3
long-library_enrichment
3 _
0 _
GFP+_+1.enr
GFP+_-1.enr
GFP+_+2.enr
GFP+_-2.enr
GFP+_+3.enr
GFP+_-3.enr

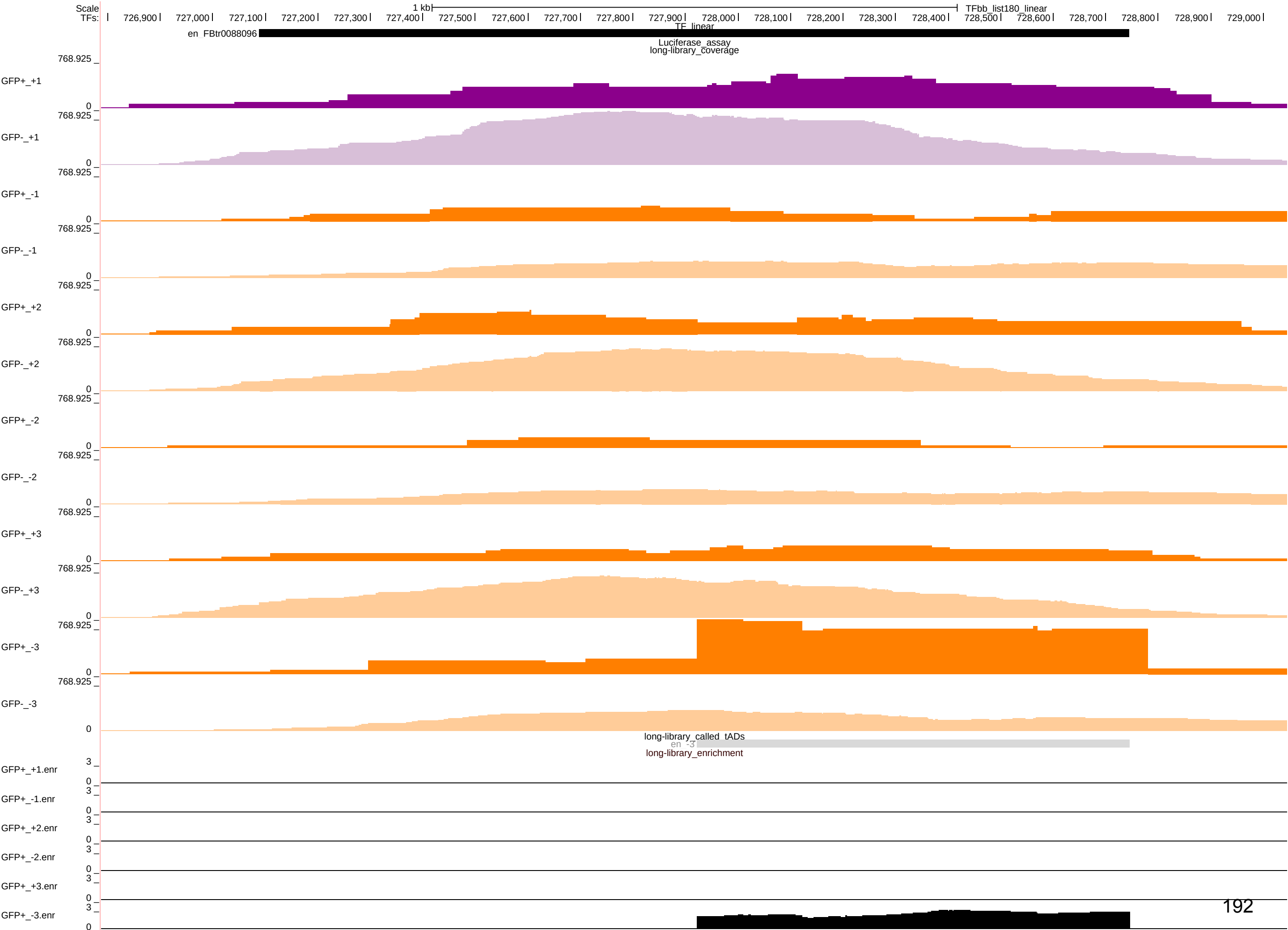
Scale
1 kb
TFbb_list180_linear
TFs:
726,900
727,000
727,100
727,200
727,300
727,400
727,500
727,600
727,700
727,800
727,900
728,000
728,100
728,200
728,300
728,400
728,500
728,600
728,700
728,800
728,900
729,000
TF_linear
en FBtr0088096
Luciferase_assay
long-library_coverage
768.925
GFP+_+1
0
GFP-_+1
GFP+_-1
GFP-_-1
GFP+_+2
GFP-_+2
GFP+_-2
GFP-_-2
GFP+_+3
GFP-_+3
GFP+_-3
GFP-_-3
long-library_called_tADs
en_-3
long-library_enrichment
3
GFP+_+1.enr
GFP+_-1.enr
GFP+_+2.enr
GFP+_-2.enr
GFP+_+3.enr
GFP+_-3.enr
