# Supplementary material for: A high‐throughput method to identify trans‐activation domains within transcription factor sequences
Source: EMBO J. 2018 Jul 13;37(16):e98896. doi: 10.15252/embj.201798896 (PMC6092621; doi:10.15252/embj.201798896)
Supplement: Supplementary file 2 — Expanded View Figures PDF [file EMBJ-37-e98896-s002.pdf]

Expanded View Figures

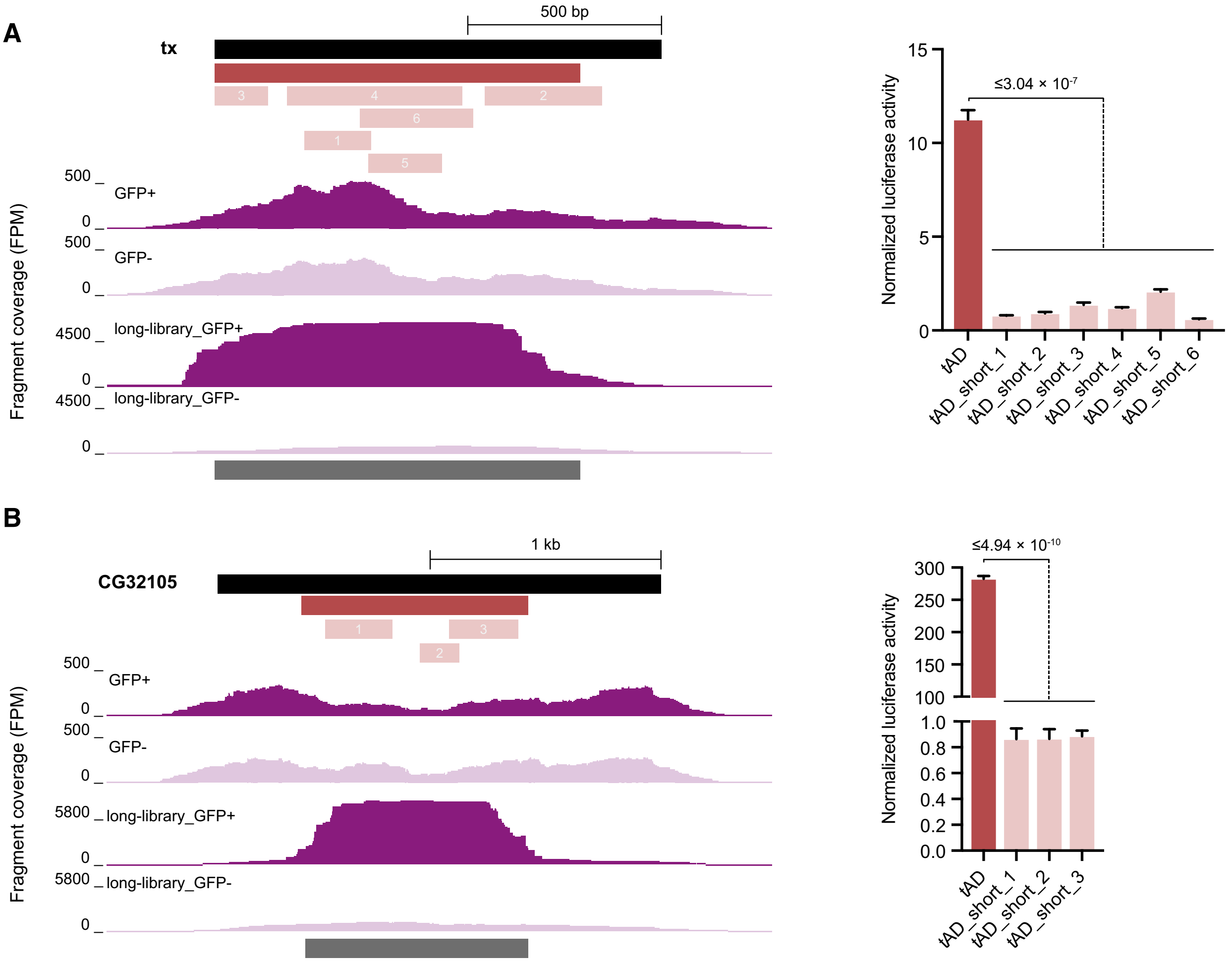

Figure EV1. tADs can have minimal sizes below which they do not function.

A, B UCSC GB screenshots displaying candidate fragment coverage for taxi (tx; frame +1) (A) or CG32105 (B) of short (top)- and long (bottom)-fragment candidate libraries from GFP$^+$ cells (dark) and GFP$^-$ cells (light). The black bar on top indicates the full-length CDS, the dark gray bars below the coverage tracks the tADs called in the long-fragment tAD-seq screen (no tAD was found in the short-fragment screen). The red bar and the light red bars indicate tAD candidates tested in luciferase assays, the normalized luciferase activities (normalized to GFP control) of long and short tAD candidates are shown on the right (n = 3 long tAD candidate; n = 4 short tAD candidates, error bars: s.d., P-value: two-sided Student's t-test vs. short tAD candidates, reported is the highest P-value).

© 2018 The Authors

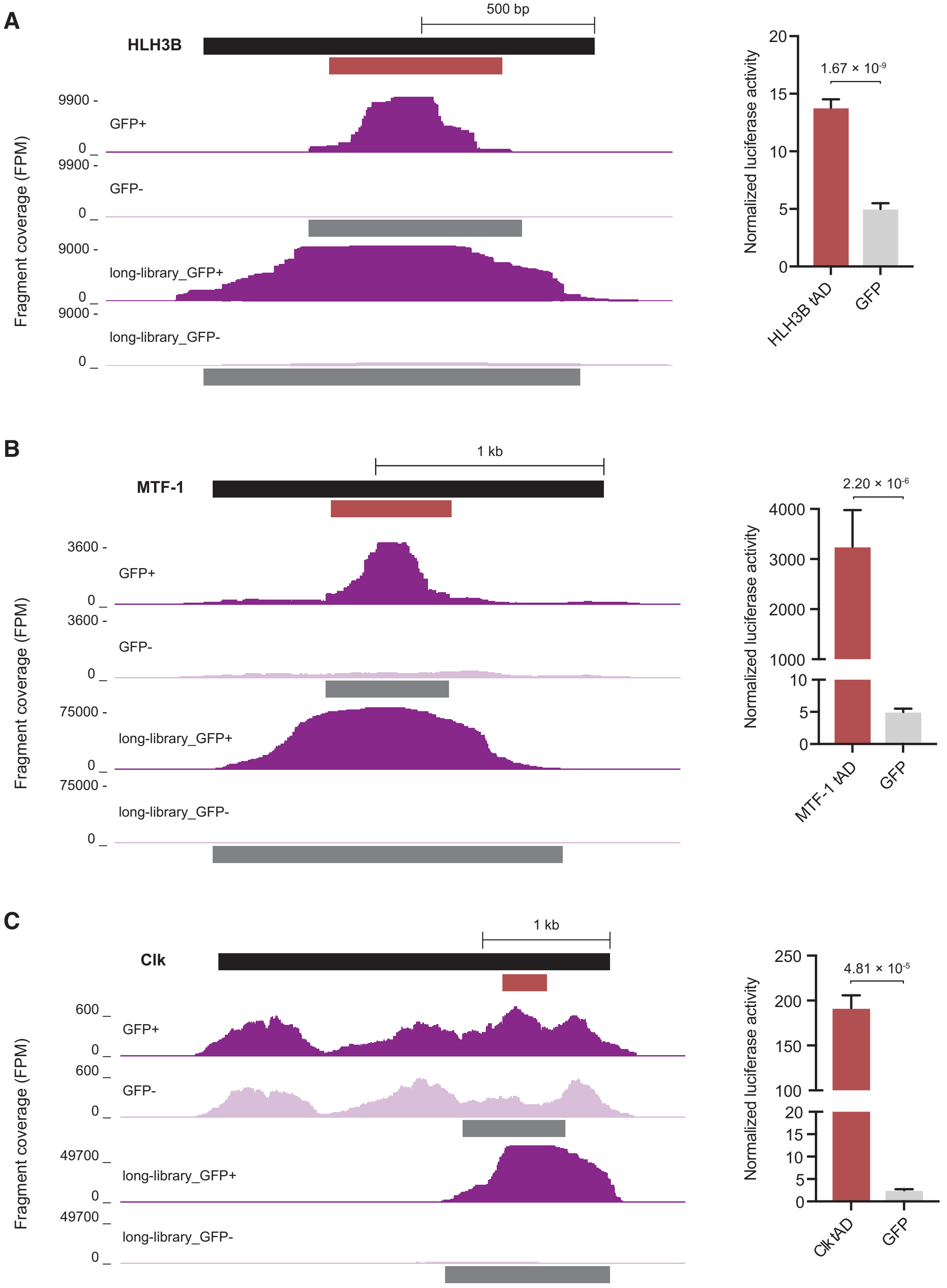

Figure EV2. Fine mapping of tAD locations.

A–C Candidate fragment coverage and tAD calls (dark gray bars below coverage tracks) for HLH3B (frame +1) (A), MTF-1 (B) or Clk (C) of short (top)- and long (bottom)-fragment candidate libraries from GFP^+ cells (dark) and GFP^- cells (light) are shown left. Normalized luciferase activities (firefly/Renilla) of the tAD candidates and the respective GFP control are shown (n = 4 for HLH3B and MTF-1; n = 3 for Clk, error bars: s.d., P-value: two-sided Student's t-test vs. GFP control) are shown right; for HLH3B, see Fig 3B). Short-fragment coverage tracks and luciferase measurements for HLH3B and MTF-1 are repeated from Figs 3B and 2, respectively, for comparison.

© 2018 The Authors

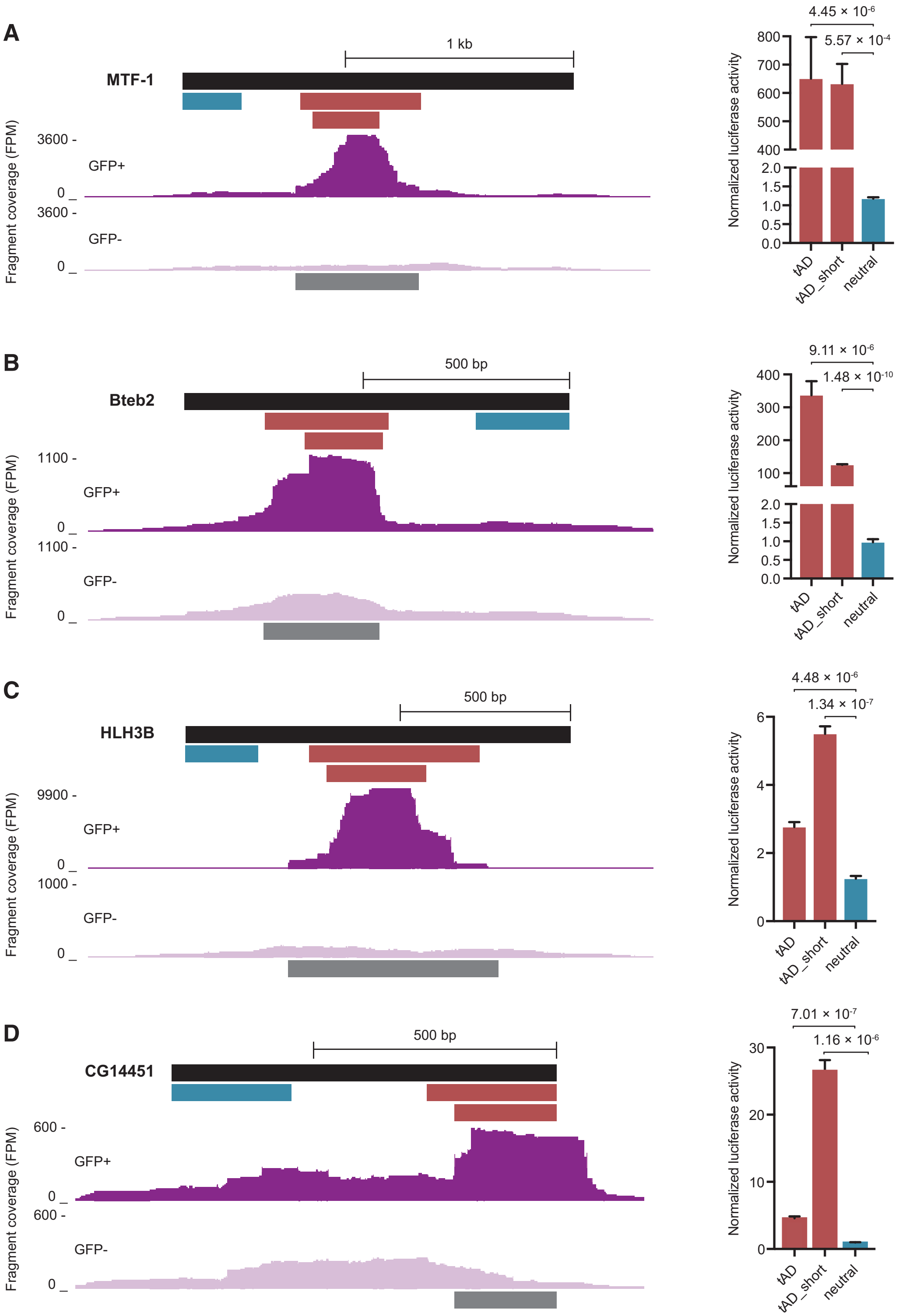

Figure EV3. Mapping of tAD boundaries.

A–D Short-fragment library and candidate fragment coverage for the native (+1) frames for GFP$^+$ and GFP$^-$ cells (dark and light purple tracks), called tADs (dark gray bars), and two luciferase assay tested tAD candidates (red) and neutral (blue) fragments are shown. Normalized luciferase activities (normalized to GFP control) for tAD candidates and neutral regions (n = 4, error bars: s.d., P-value: two-sided Student's t-test vs. neutral region; n = 3 CG14451-tAD_short). Data for coverage tracks and luciferase assays of tAD candidates (labeled: tAD) and neutral regions repeated from Figs 2 and 3 for comparison.

 © 2018 The Authors
