# Supplementary material for: A high‐throughput method to identify trans‐activation domains within transcription factor sequences
Source: EMBO J. 2018 Jul 13;37(16):e98896. doi: 10.15252/embj.201798896 (PMC6092621; doi:10.15252/embj.201798896)
Supplement: Supplementary file 3 — Table EV1 [file EMBJ-37-e98896-s003.zip › Table_EV1/Table_EV1_Legend.docx]

**Expanded View Table Legend**

Table EV1: Candidate library composition

List of 180 TF coding sequences flanked by 510bp of the upstream and downstream plasmid backbone sequence, that are contained in the short- and long-fragment library
